# Stability of bicyclic guanidine superbases and their salts in water†

**DOI:** 10.1039/d4ra08751h

**Published:** 2025-02-13

**Authors:** Eva Gazagnaire, Jussi Helminen, Thomas Golin Almeida, Petri Heinonen, Markus Metsälä, Theo Kurten, Ilkka Kilpeläinen

**Affiliations:** a Department of Chemistry, Faculty of Science, University of Helsinki FI-00560 Helsinki Finland ilkka.kilpelainen@helsinki.fi; b Institute for Atmospheric and Earth System Research/Chemistry, Faculty of Science FI-00560 Helsinki Finland

## Abstract

Bicyclic guanidines are widely used in organic chemistry as strong basic reagents and catalysts. They are also utilized in CO_2_ chemistry, ring opening polymerization reactions and in cellulose dissolution or processing as ionic liquids. Despite their wide applicability, they are usually sensitive to water, especially under alkaline conditions. The salts of guanidines usually exhibit slower degradation rates but are still somewhat prone to hydrolysis. We studied the hydrolysis rates of a series of different bicyclic guanidines and their carboxylate salts under different aqueous conditions. It appears that bicyclic guanidines and their salts are in principle stable in water but undergo hydrolysis in the presence of hydroxide ions (OH^−^). Therefore, it is feasible to design bicyclic guanidine bases that are highly tolerant to water, but can still simultaneously possess different desirable chemical characteristics, like hydrophobicity to allow compatibility under varying conditions.

## Abstract 2

Bicyclic guanidines are widely used in organic chemistry as strong basic reagents and catalysts.

## Graphical abstract

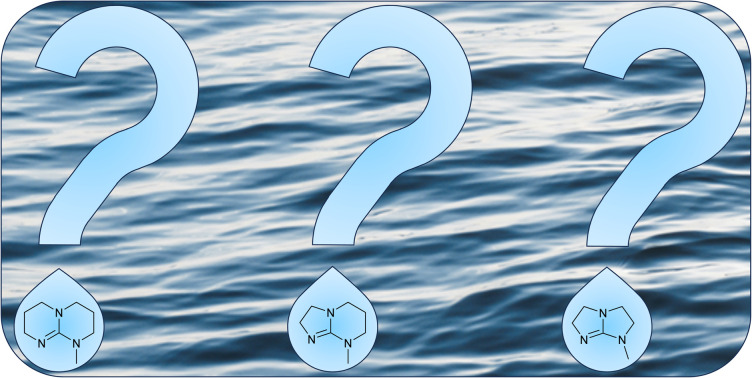

## Introduction

Guanidines are strong organic bases that are widely used in chemistry as basic reagents or catalysts. Their solubility in organic solvents makes them advantageous compared to inorganic bases.^1^ Acyclic structures such as 1,1,3,3-tetramethylguanidine (TMG) have found various applications but suffer from poor thermal and water stability.^2,3^ Bicyclic structures show various advantages compared to their acyclic analogues. Their rigid skeleton stabilizes the protonated form which directly increases the basicity of the molecule.^4,5^ For example, the commercially available bicyclic organic bases, 1,5,7-triazabicyclo[4.4.0]dec-5-ene (TBD) and its methylated derivative 7-methyl-1,5,7-triazabicyclo[4.4.0]dec-5-ene (mTBD) are approximately 100 times more basic than TMG.^6^

The general hydrolysis of acyclic guanidines begins with an attack of a nucleophile on the central carbon atom of the guanidine, followed by the cleavage of one of the C–N bonds. This then leads to the formation of an amine (or ammonia) and urea as the hydrolysis products.^2,7,8^

**Fig. 1:**
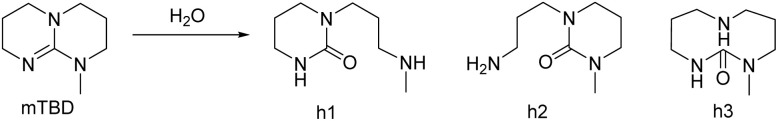
The hydrolysis of mTBD can yield hydrolysis products h1 (favoured), h2 (minor) and h3 (not observed).^10^

The hydrolysis of bicyclic guanidines follows the same pathway as acyclic guanidines. Due to the delocalization of the imine double bond in guanidines, the hydrolysis can yield three different products depending on which one of the C–N bonds breaks (Fig. 1).^7–9^ For mTBD, the main hydrolysis product is the ring opening product h1 (Fig. 1), while the opening leading to a macrocycle formation is highly unfavourable (h3 in Fig. 1).^9,10^ If the contact with water cannot be avoided, the hydrolytic instability of guanidines can jeopardize their usability. However, this propensity for hydrolysis has been used as an advantage in some rare examples, for example, using the hydrolysis product as an *in situ* formed reagent.^3,9^

The hydrolysis rate of guanidine superbases increases with increasing water amount, which is typical for chemical reactions involving two species.^11,12^ It has also been reported that the hydrolysis rate decreases as a function of decreasing pH.^13,14^ This is in line with the postulated stabilization of the protonated form, *i.e.* salt formation.

Several types of ionic liquids have shown good efficiency in dissolution of cellulose and biomass.^15–18^ Also the protonated salts of organic superbases (SB-ILs) are efficient for dissolution of cellulose but have also shown their potential generally in organic chemistry.^19–22^ Superbase salts can be readily prepared by reacting the superbase with organic (*e.g.* formic acid, acetic acid) or mineral acids (H_2_SO_4_, HNO_3_). Our interest has been mainly focused in the carboxylate salts of bicyclic guanidine superbases, which are excellent solvents for cellulose and enable regeneration of cellulose for example to high-quality textile fibers, while being relatively stable against hydrolysis.^12,23–28^ For example, [mTBDH][OAc] can be utilized in the IONCELL process to produce man-made textile fibers.^12,18^ In this process, [mTBDH][OAc] is used to dissolve cellulose, which is then regenerated as thin filaments into a water bath. Being water soluble, the SB-IL remains in the aqueous phase.^10,29^ For recycling, the [mTBDH][OAc] is regenerated from the spinning bath by evaporation of water under reduced pressure at elevated temperature. This repeated contact with water leads to slow hydrolysis of [mTBDH][OAc].^10^ Schlapp-Hackl *et al.* pointed out that the hydrolysis of [mTBDH][OAc] is dependent on the amount of water – a low concentration of water (around 10 wt%) resulted in a higher hydrolysis rate.^12^

**Fig. 2:**
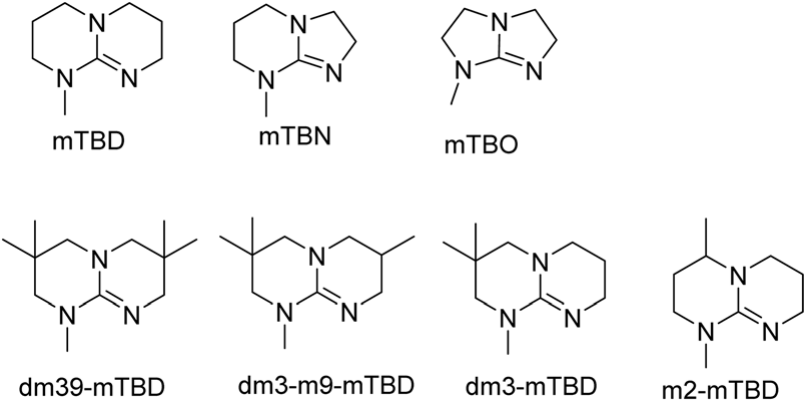
Structure of bicyclic guanidine superbases studied in this work. mTBN, dm3-m9-mTBD, dm3-mTBD and m2-MTBD were used as mixtures of positional isomers.^26^

We have recently shown that the hydrolytic stability of bicyclic guanidine superbases can be significantly improved by varying the ring sizes, and especially by introducing suitable sidechains.^26^ In other words, these novel structures are able to resist hydrolysis at variable conditions but retain their ability to dissolve cellulose as the acetate salts. In the current work, we studied the relative hydrolytic stabilities of these bicyclic guanidine superbases, and their acetate salts (Fig. 2) in more detail.

## Results and discussion

Bicyclic guanidine superbase carboxylate salts offer possibilities for dissolution and regeneration of cellulose to produce man-made textile fibers.^25^ While the bicyclic structures show improved hydrolytic stability of the acetate salts, they still undergo hydrolysis under aqueous conditions. We have earlier reported a series of novel structures (Fig. 2) with considerable hydrolytic stability as acetate salts.^26^ It was shown that in general methyl substituents on the bicyclic core helps to stabilize the superbase salt in aqueous media. However, among the bicyclic guanidines, mTBO and mTBN showed rather high stability in water especially at higher temperatures.

### Stability of bicyclic guanidine superbases in water

The hydrolytic stability of the superbases was studied by varying the water concentrations (0.2, 0.5, 1, 5 and 10 molar equivalents according to the superbase) and following the hydrolysis of each superbase for 48 h at 90 °C using ^1^H NMR. The series of the spectra was used to obtain the half-life time of the hydrolysis reaction, *i.e.* the time needed to convert 50 mol% of a superbase to its hydrolysed products (see details Table S136 in ESI†). As expected, there is a tendency for all of the superbases to hydrolyse faster when the water concentration increases. However, the difference of half-life time between 1 eq. and 10 eq. of water is in many cases larger than expected on basis of stoichiometric concentrations. Furthermore, the number of methyl substituents on the bicyclic core of the superbase did not affect the stability as much as we assumed initially. It appears that at low water concentrations (≤1 molar equivalent) the superbases are rather stable with half-life time of several hours (Table 1). Somewhat surprisingly, the hydrolytic stabilities of the mTBD analogues (dm39-mTBD, dm3-m0-mTBD, dm3-mTBD and m2-mTBD) as free bases are rather close to each other, while mTBO and mTBN appear to be more stable regardless of the water concentration. The apparent stability of mTBO is due to its lower basicity, *i.e.* the hydroxide ion concentration in mTBO/water mixtures is lower resulting in slower hydrolysis rate. However, the relatively good stability of mTBN is likely due to lower reactivity of the protonated base due to steric reasons.

**Table 1:** Comparison of the half-life times and amount of hydrolysis of the bicyclic guanidines after 48 hours in varying amounts of water^c^

Structure	Half-life time (hours^a^) in increasing eq. of water at 90 °C					Hydrolyzed % after 48 h in increasing eq. of water^b^				
	0.2	0.5	1	5	10	0.2	0.5	1	5	10
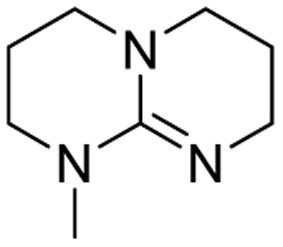	8	6	8	0.5	0.5	20.9	50.8	65.4	100	100
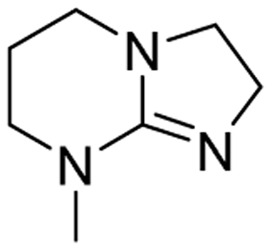	113	90	87	2.3	0.9	4.9	16.8	33.3	98.0	98.9
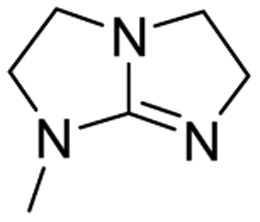	55	N.P	71	1.3	0.8	4.4	8.1	31.0	96.6	97.3
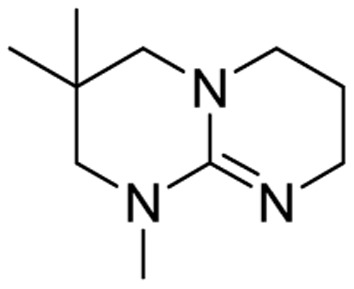	4	4	6	1.0	0.7	26.6	54.9	64.0	91.2	96.1
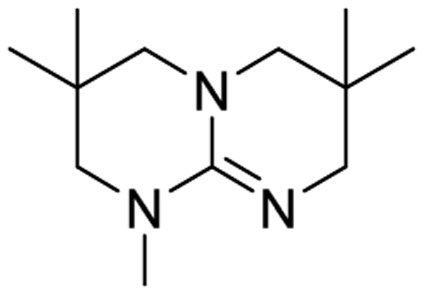	n.d.	n.d.	35	0.7	0.5	n.d.	n.d.	98.2	99.8	100
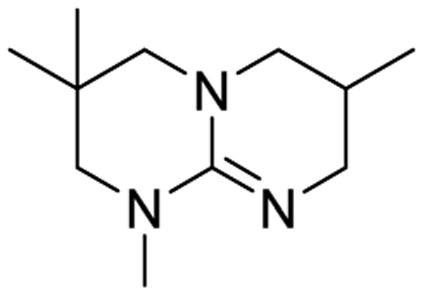	n.d.	n.d.	42	3	2	n.d.	n.d.	54.5	97.4	100
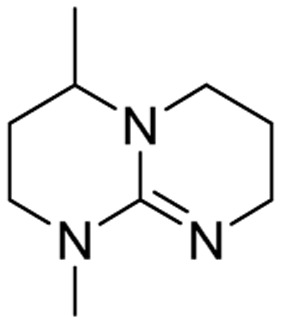	n.d.	n.d.	41	0.7	0.5	n.d.	n.d.	53.2	99.7	98.7

a For times >1 h the half-life is given in whole hours.

b When the half-life time is longer than 48 h, the half-time is noted non-predictable (N.P) (see details in Table S134). n.d. states for ‘not determined’ due to limited solubility into water.

c The presence of structural isomers (see Fig. 2) was not accounted for in the given values, *i.e*. the listed values represent overall value for the isomeric mixtures.

**Fig. 3:**
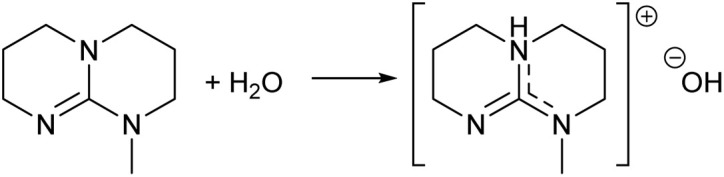
Acid–base reaction of guanidines with water yields protonated guanidine and hydroxide anion salt (formation of hydroxide salt).

Organic superbases are, by definition, compounds having a very high basicity.^30^ Therefore, water interacts with superbases as an acid forming stoichiometric superbase hydroxide salts (Fig. 3). If this was the case, the dilute aqueous superbase solutions should have properties comparable to the solutions of alkali metal hydroxide salts, like KOH. Indeed, the pH values of dilute KOH and SB solutions have an identical concentration dependence (see ESI†). Also, the titration curve of a dilute weak acid, such as acetic acid, and mTBD show remarkable similarity with a corresponding titration curve of weak acid and KOH (see ESI†). It is therefore apparent that in dilute aqueous solutions the superbase is fully protonated and exist essentially as the hydroxide salt (Fig. 3).

Direct pH measurement becomes meaningless for concentrated solutions and other approaches are required. The bicyclic guanidine salts have rather characteristic Bohlmann bands in their infrared (IR) spectra, that could in principle be used to monitor the protonation degree of the base.^31^

To follow the possible formation of the hydroxide salts of guanidines, at low concentration of water, we thus first attempted to utilize IR. It has been shown that upon protonation, the intense Bohlmann bands of bicyclic guanidine (mTBD) disappear, and new, less intense signals of the protonated form appear.^32^ This phenomenon is due to the adaptation of the guanidine moiety into a planar structure upon protonation.^33^

Hence, we carried out a titration experiment of mTBD with water. The CH_2_ vibrations at 2700–3000 cm^−1^ indeed undergo a blueshift of approximately 40 cm^−1^, but the change is a continuous phenomenon (see Fig. S168, S172, S176 and S180 in ESI†), depending on the amount of water and thus rather difficult to correlate to any theoretical model for protonation or hydration of the superbase (see ESI†). Notably, in parallel to the observed blueshift the imine stretch at 1600 cm^−1^ undergoes a redshift in concert with the disappearance of the Bohlmann bands. Similar trends were observed for all the other studied superbases. The IR titration method may be sensitive to the salt formation and provide snapshots of different species in the equilibria, but does not give, at least directly, information about the hydroxide salt formation of the guanidines that could easily linked to the mechanism of the hydrolysis reaction.

**Fig. 4:**
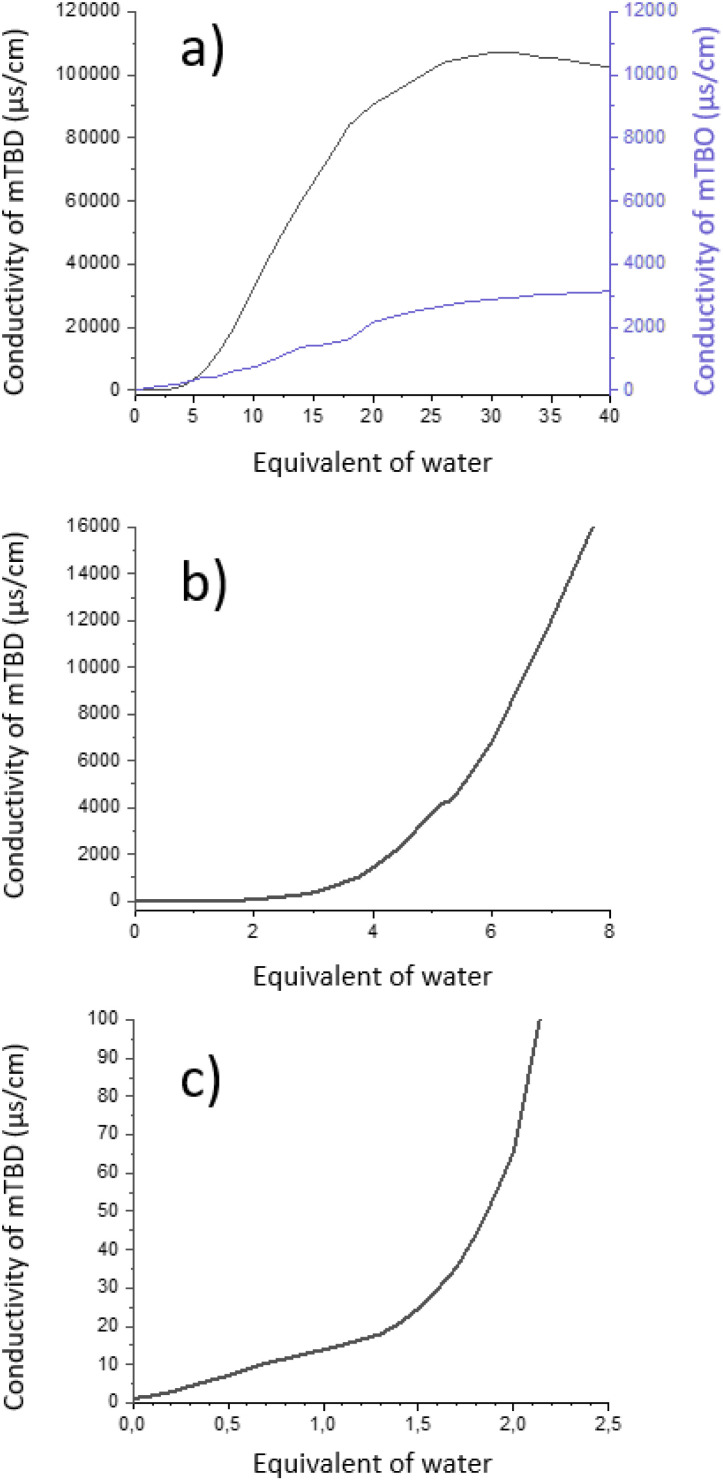
Conductivity dependence of mTBD (black) and mTBO (purple) in presence of different water amounts. (a) There is a rather large difference between conductivities of mTBD (a superbase) and mTBO (a weak guanidine base) in water. Expansion of the graph for mTBD shows two turning points. For mTBD, (b) there is one turning point around 3.5 eq. of water, and (c) another one around 1.3 water equivalents.

As the IR results were able to follow the free base–salt transition only indicatively, we turned our attention to the conductivity measurements (Fig. 4). The conductivity of a solution is caused by the movement of ions. It cannot be directly used to measure the stoichiometry of the hydroxide salt formation, but it can be an efficient tool to follow the formation and the hydration of the salt and provide information about the mobility of the ionic species in the solution. We carried out the conductivity measurements by successive additions of water to the superbase solution at RT (room temperature). It should be noted that although the superbases can undergo hydrolysis, the reaction is relatively slow at RT allowing for measurements without interference from hydrolysis.

The conductivity profiles of strong superbases (*e.g.* mTBD in Fig. 4) in water are overall similar to other conductivity profile of organic salts.^34^ However, a closer inspection reveals some unique features at low amounts of water. The conductivity of mTBD is low and increases only slightly up to 1.3 equivalents of water, where there is a clear turning point (Fig. 4c). Another turning point appears at 3.5 eq. of water (Fig. 4b). As the numerical values for the conductivity are small, these turning points remain easily unnoticed, but are critical. There exist no such turning points in conductivity of weaker bases, such as mTBO. In that case the conductivity shows only a linear increase with increasing amount of water (Fig. 4a).

Up to the first turning point of mTBD with 1.3 eq. of water, the conductivity increases slowly in a liner fashion, which indicates salt formation, but the formed salt ion pairs remain associated and are not free to move (Fig. 4c). After the first turning point at 1.3 eq., the conductivity continues to steadily increase to ∼3.5 equivalents of water, where is a second turning point (Fig. 4b). After the second turning point, the conductivity of the mTBD/water increases strongly indicating increasing free movement of ions (Fig. 4a). After a maximum value of 40 000 μS cm^−1^ at 1 : 20 mTBD : H_2_O the graph follows typical dilution behaviour of salt solutions (see ESI† p. 156–161 for the other superbases, like mTBN).

The conductivity behaviour of mTBD and other superbases between 1.3 to 3.5 eq. of water is different from a weaker base, mTBO. For example, at 2 eq. of water mTBO conductivity is higher (118) than superbases mTBD (66), mTBN (22) and dm3-mTBD (82). As the viscosity of their water solutions are quite low/similar, there likely exists also other contributing factors. For example, aggregation of superbase hydroxide salts to larger aggregates could cause lower mobility of ions resulting in lower conductivity, or possibly the strong bases still exist as associated ion pairs before complete dissolution above 3.5 eq. of water.

A similar behaviour in conductivity at low water amounts is also apparent in previously published graphs for stable organic salts/ionic liquids, even though not commented.^34^

When the conductivity measurement results and the rates of the hydrolysis reactions are considered together, the relative stability of superbases at low water contents (≤1 molar equivalent, half-time of several hours) can be linked with the limited mobility of ions of the formed hydroxide salt. Increasing the water content over 1 : 1 leads to an acceleration of the hydrolysis rate as the mobility of ions and the concentration of freely moving hydroxide ions increase (see Table 1 and ESI† for details).

The conductivity of mTBO is drastically different from the other bicyclic guanidines (Fig. 4a). mTBO is at the borderline of being a superbase^26^ which implies that its capacity to form a hydroxide salt with water is weaker. This is also reflected in its conductivity behaviour, as it does not have clear turning points, like other superbases. Even though the overall appearance of the conductivity graph for mTBO/water is similar to the superbases, its maximum conductivity is drastically lower, which is directly linked to the weaker base nature of mTBO (*c.f.* ESI†).

### Stability of bicyclic guanidine superbases salts in water

**Fig. 5:**
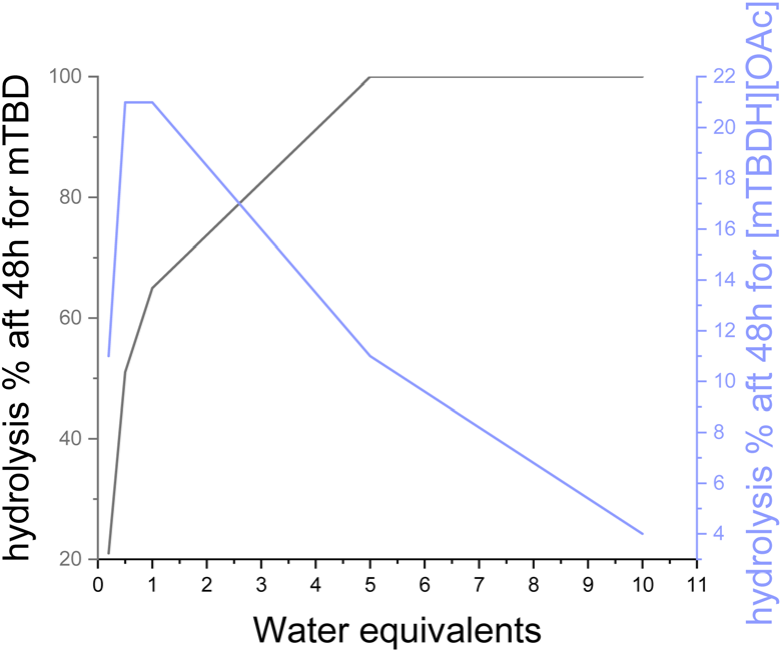
Comparison of the hydrolysis rate of mTBD and [mTBDH][OAc] in water. Clearly, the free SB hydrolysis rate increases proportionally to the amount of water. This is in strict contrast to the SB-IL which reaches a maximum hydrolysis rate at around 1 equivalent of water and slows down progressively with increasing amount of water.

To study the water stability of the superbase acetate salts, we conducted similar hydrolysis test series as for the free bases. In contrast to the aqueous solutions of free superbases, the hydrolysis rate of a SB acetate salts increase with decreasing amount of water (see Fig. 5), which is the opposite to the free bases. The acetate salt of mTBD, [mTBDH][OAc] has a half-life time of 69 h in 0.5 eq. of water, 110 h in 1 eq. of water, and is essentially stable in 10 eq. of water at 90 °C.

As the hydrolytic stability of [mTBDH][OAc] increases with increasing amount of water, the hydrolysis is likely linked to the alkalinity of the acetate anion, *i.e.* the hydrolysis is due to hydroxide anion (generated by the acetate anion) acting as a nucleophile, analogously to the hydrolysis reactions of free bases. With this in mind, we conducted similar stability test for superbase salts with acids with increasing acidity. As expected, the mTBD salts of analogous stronger acids, chloroacetic acid (p*K*_a_ = 2.87), dichloroacetic (p*K*_a_ = 1.25) and trichloroacetic acid (p*K*_a_ = 0.51) did not undergo any hydrolysis, independent from amount of water. Further, all the salts of the bicyclic guanidine superbases with other strong acids were stable against hydrolysis over a period of 48 h at 90 °C, again regardless the amount of water (see ESI†). However, as already noted for the acetate salts, the situation was markedly different for weak acid salts. As the strength of acid decreases, its conjugate base becomes stronger and the anion of the superbase salt is capable of deprotonating the surrounding water molecules, *i.e.* increasing alkalinity.

In case of acetate salts, [mTBOH][OAc] appeared to be the least stable, whereas mTBO was relatively stable as the free base (Table 1, ESI†). Being a weaker base, mTBO does not form a stoichiometric hydroxide salt with water, like the other superbases, which explains the higher stability of the free base in water. However, there are likely several reasons causing the limited hydrolytic stability of [mTBOH][OAc]. Acetic acid (*i.e.* a stronger acid than water) can protonate mTBO to a certain degree. Therefore, the acetate ion concentration of aqueous [mTBOH][OAc] solutions is relatively high causing alkalinity and thus increasing hydrolysis speed. Further, the protonated form of mTBO is rather strained, which decreases its stability against hydrolysis.

The pH of aqueous solutions of [mTBDH][OAc] increases identically to KOAc solutions with increasing salt concentration (see ESI†). In other words, the lower is the water amount, the higher is the concentration of hydroxide anions. The hydrolysis rate of superbase acetate salt [mTBDH][OAc] has its maximum at 1 : 1 SB-IL : water (10 m-%), which is in agreement with earlier observations.^12^ In this point also the hydroxide anion concentration has its maximum. Addition of more water dilutes the solution decreasing the effective hydroxide anion concentration. This behaviour agrees with the results obtained for free bicyclic guanidine superbases, as in both cases, the hydrolysis speed is dependent only on the hydroxide anion concentration, not on water itself.

For cellulose dissolution in SB-ILs, it is possible to use polar, aprotic co-solvents, like DMSO, without jeopardizing the capability of the SB-IL to dissolve cellulose. It also might be possible to utilize entertainers or additives to compete or slow down the hydrolytic reactions. Despite extensive trials with different co-solvents and/or possible entertainers (see ESI†), their effect on the hydrolysis rate was only small.

### On the hydrolysis of the bicyclic guanidines and their salts in water

The first step of hydrolysis reaction is the attack of a nucleophile to an electrophile. In general, the hydrolysis of imines is an acid catalysed reaction.

In principle, there are four possibilities for the hydrolysis reaction of guanidine superbases to take place, as the nucleophile can be water or hydroxide anion, and the electrophile can be free base or protonated base.

From the hydrolysis data of bicyclic guanidine superbase salts with strong and/or weak acids show that the reactive nucleophilic species is hydroxide anion, not water itself. This is in good agreement with the recycling tests of [mTBDH][OAc] and [mTBNH][OAc], where the progress of hydrolysis leads to decrease of alkalinity and slowing down the hydrolysis of these ILs.^10,12,35^

While the role of hydroxide anion is clear, the hydrolysis could still progress through the attack of hydroxide to a free base or to the protonated form of the base, as there is always an equilibrium between protonation and the deprotonation, regardless of the strength of the base. All our data suggests that the reacting electrophilic species is the protonated base analogously to hydrolysis of imines.^36^ Also, molecular modelling supports these findings. The reaction free energy barrier is considerably lower for OH^−^ reacting with the protonated superbase than other alternative nucleophile and electrophile combinations (see ESI†). However, a detailed mechanistic study of the hydrolysis reaction pathway and intermediates is out of the scope of the current study.

The high basicity of guanidine superbases originates from the stabilization of the protonated form by conjugation.^37^ While this is the case, this should not be understood as stabilization in general, but only to explain the high proton affinity of the guanidine superbases. In a way, the hydrolysis reaction of guanidine superbases is paradoxical: ‘acid catalysed reaction of hydroxide anion'. This behaviour results in seemingly contradicting phenomena under different conditions but is a natural consequence of the molecular properties.

## Experimental

Description of the general synthetic and hydrolysis procedures can be found in the electronic ESI† associated with this article. Additionally, the used materials, computational methods and instrumentation are depicted in the ESI.†

## Conclusions

In summary, we have studied the hydrolysis of bicyclic guanidine superbases and their corresponding salts in water. The hydrolysis reaction follows the typical hydrolysis mechanism of imines but have some unique characteristics due to the nature of these superbases.

In an aqueous environment, the bicyclic guanidine superbases form hydroxide salts. The hydroxide anion is always the reactive species (nucleophile) in the hydrolysis reaction, both for free base solutions and for their salts. Superbase salts with strong acids are stable in water, but salts with weak acids undergo hydrolysis due to the basic character of the anion.

The hydrolysis of the cellulose dissolving SB-ILs, like [mTBDH][OAc] and [mTBNH][OAc] is due to the basicity of the acetate anion. It appears to be possible to avoid the hydrolysis by changing the acid component of the ionic liquid. Unfortunately, the acetate (or carboxylate) anion plays a crucial role for breaking down hydrogen bonding of cellulose making these salts attractive for cellulose/biomass dissolution.

Bicyclic guanidine superbases are a large family of structures with varying ring sizes and possible substitution patterns. The stability of the acetate salts can be significantly improved with simple methylation patterns, as the alkalinity of these aqueous solutions is limited to the alkalinity of the acetate anion, but avoiding hydrolysis completely for the bases themselves requires more advanced approaches, as the challenge is to stabilize their hydroxide salts.

## Data availability

The data supporting this article have been included as part of the ESI.†

## Author contributions

Eva Gazagnaire: investigation, methodology, validation, writing – original daft. Jussi Helminen: writing – review & editing, formal analysis. Thomas Golin Almeida: modelling work, writing – review & editing. Petri Heinonen: formal analysis, writing – review & editing. Markus Metsälä: formal analysis, writing-review & editing. Theo Kurten: formal analysis, writing – review & editing, supervision of modelling work. Ilkka Kilpeläinen: conceptualization, investigation, supervision, resources, writing – review & editing, funding acquisition.

## Conflicts of interest

There are no conflicts to declare.

## Supplementary Material

RA-015-D4RA08751H-s001
