# Supplementary material for: Stability of bicyclic guanidine superbases and their salts in water
Source: RSC Adv. 2025 Feb 13;15(7):4945–51. doi: 10.1039/d4ra08751h (PMC11824616; doi:10.1039/d4ra08751h)
Supplement: RA-015-D4RA08751H-s001 [file RA-015-D4RA08751H-s001.pdf]

Supplementary Information (SI) for RSC Advances.
This journal is © The Royal Society of Chemistry 2025

Supporting information

Stability of Bicyclic Guanidine Superbases and Their Salts in Water

Eva Gazagnaire[a], Jussi Helminen[a], Thomas Golin Almeida[b], Petri Heinonen[a], Markus Metsälä[a], Theo Kurten[b] and Ilkka Kilpeläinen*[a]

[a] Department of Chemistry, Faculty of Science, University of Helsinki, FI-00560 Helsinki, Finland

[b] Institute for Atmospheric and Earth System Research/Chemistry, Faculty of Science, FI-00560 Helsinki, Finland

Corresponding author: ilkka.kilpelainen@helsinki.fi

1. Table of Contents

General information 5

a. General procedure for the synthesis of ionic liquid 5

b. General procedure of SB/SB-IL hydrolysis 5

I. Absolute integrals and hydrolysis calculations 5

1. mTBD 5

a. mTBD + water over 48h 5

b. mTBD + water at 70°C over 12 days 10

c. mTBD + water at 90°C over 12 days ii 13

d. mTBD + water + DMSO 16

e. mTBD + water + ethylene glycol 20

f. mTBD + water + phenol 24

g. mTBD + water + propanol 28

2. [mTBDH][OAc] 32

a. [mTBDH][OAc] + water 32

b. [mTBDH][OAc] + water + DMSO 37

c. [mTBDH][OAc] + water + ethylene glycol 41

d. [mTBDH][OAc] + water + phenol 45

e. [mTBDH][OAc] + water + propanol 49

f. [mTBDH][ClOAc] + water 53

g. [mTBDH][Cl2OAc] + water 58

h. [mTBDH][Cl3OAc] + water 63

3. mTBN 68

a. mTBN + water over 48h at 90°C. 68

b. mTBN + water over 12 days at 70°C. 73

c. mTBN + water over 12 days at 90°C. 76

4. [mTBNH][OAc] + water 79

a. [mTBNH][OAc] + water over 48h at 90°C. 79

b. [mTBNH][OAc] + water at 90°C over 12 days. 84

c. [mTBNH][OAc] + water at 70°C over 12 days. 87

d. [mTBNH][ClOAc] + water 90

e. [mTBNH][Cl2OAc] + water 95

f. [mTBNH][Cl3OAc] + water 100

5. mTBO + water 105

6. dm39-mTBD + water 110

7. [dm39-mTBDH][OAc] + water 112

8. dm3-m0-mTBD + water 115
9. [dm3-m0-mTBDH][OAc] + water 118
10. dm3-mTBD + water 121
11. [dm3-mTBDH][OAc] + water 126
12. m2-mTBD + water 131
13. [m2-mTBDH][OAc] + water 134
II. Half-time calculation 137
1. SB and their salts with pure water at 90°C 137
2. Superbase and their acetate salt in presence of water and co-solvent at 90°C 139
3. SB and their acetate salts over a three days period at different temperature 140
III. Curves of hydrolysis 141
1. mTBD with different amounts of water 141
2. mTBD with different amounts of DMSO 141
3. mTBD with different amounts of Ethylene Glycol 142
4. mTBD with different amounts of Phenol 143
5. mTBD with different amounts of Propanol 143
6. mTBN with different amounts of water 144
7. dm39-mTBD with different amounts of water 144
8. dm3-m0-mTBD with different amounts of water 145
9. dm3-mTBD with different amounts of water 145
10. m2-mTBD with different amounts of water 146
11. [mTBDH][OAc] with different amounts of water 146
12. [mTBDH][OAc] with different amounts of DMSO 147
13. [mTBDH][OAc] with different amounts of Ethylene Glycol 147
14. [mTBDH][OAc] with different amounts of Phenol 148
15. [mTBDH][OAc] with different amounts of Propanol 148
16. [mTBNH][OAc] with different amounts of water 148
17. [dm39-mTBDH][OAc] with different amount of water 149
18. [dm3-m0-mTBDH][OAc] with different amount of water 149
19. [dm3-mTBDH][OAc] with different amount of water 150
20. [m2-mTBDH][OAc] with different amount of water 150
IV. Conductivity 151
1. General procedure: 151
2. Curves and raw data. 151
a. Water titration of mTBD 151
b. water titration of mTBN 154

c. Water titration of dm3-mTBD ... 156

d. Water titration of mTBO ... 158

V. PH measurements ... 161

a. KOH or mTBD in pure distilled water ... 161

b. KOH or mTBD in distilled water with acetic acid ... 162

c. pH of KOAc and [mTBDH][OAc] solutions ... 163

VI. InfraRed measurements ... 163

1. FTIR measurements of water titration ... 163

a. mTBD ... 163

b. mTBN ... 166

c. dm3-mTBD ... 168

d. mTBO ... 170

VII. Prediction of reaction free energy barriers ... 172

VIII. References: ... 175

General information

All the used superbase ionic liquids were prepared in our research group by following the procedure published by Gazagnaire et al.[1] Liquid state ^{1}H were recorded at 27°C using a Bruker Avance Neo 600 [599.69 MHz]. ^{1}H and spectra were referenced to the residual solvent signals of DMSO-d_6 2.50.

a. General procedure for the synthesis of ionic liquid

In a 20ml vial 1 molar equivalent of superbase is mixed with 1 molar equivalent of acid and stirred for an hour. The color of the ionic liquid varies from colorless to dark orange and the melting point varies from room temperature to 60°C.

For the acetate salt the addition of the acetic acid to the superbase must be slow due to exothermicity. In the contrary, for strong acid (e.g., chloroacetic acid, dichloroacetic acid, etc.) the addition of the acid can be ffast,and the reaction mixture heated to 60°C, otherwise the acid (solid at room temperature) doesn't dissolve into the superbase.

Acetate salt liquid at room temperature: [mTBNH][OAc], [mTBOH][OAc], [dm3-m0-mTBDH][OAc], [dm3-mTBDH][OAc], [m2-mTBDH][OAc]

Acetate salt solid at room temperature: [dm39-mTBDH][OAc]

Strong acid salt liquid at room temperature: [mTBNH][ClOAc], [mTBNH][Cl_2], [mTBDH][ClOAc]

Strong acid salt solid at room temperature: [mTBNH][Cl_3OAc], [mTBDH][Cl_2], [mTBDH][Cl_3OAc]

b. General procedure of SB/SB-IL hydrolysis

In an 8ml vial 1g of SB-IL is mixed at room temperature with a defined molar equivalent of water (0.2. 0.5. 1. 5 or 10) just before the start of the kinetic studies. Then the vial is placed into an oven at 90°C. Then every hour, an NMR sample is taken from the vial and analyzed by NMR. For each analysis, the NMR tube was filled with 0.025g of sample and 0.5g of DMSO d_6 was added to allow NMR spectrometer locking and shimming. The amount of hydrolysed product was calculated according to the published formula by Sturm et. all (reference 2 in the main article). The Integration calculation was done with MesReNova software, using the Sum method, algorithm: signal picking. For the hydrolysis experiment containing a co-solvent ('entertainer', such as DMSO, phenol, ethylene glycol, etc.) the molar equivalent added is always calculated according to the amount of SB-IL or SB.

I. Absolute integrals and hydrolysis calculations

1. mTBD

a. mTBD + water over 48h

Structure	Time	Integral N-Methyl	Integral hydrolyzed product 1	Integral hydrolyzed product 2	total hydrolysis (mol %)
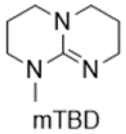 mTBD	REF	61581200	0.00	0.00	0.00
	1h	457596000	4510630	2641810	1.5
	2h	489849000	12322700	4076470	3.2
	3h	398653000	12694200	3332960	3.9
	4h	426732000	19799200	3880470	5.3
	5h	476445000	29529400	6312800	7.0
	6h	414737000	29058400	7200930	8.0
	7h	461302000	34095800	7570780	8.3

	8h	450756000	41473500	8886220	10.0
	24h	320014000	56818900	9535340	17.2
	48h	347093000	78557400	13356700	20.9

Table S1 1 molar equivalent of mTBD, 0.2 molar equivalent of water.

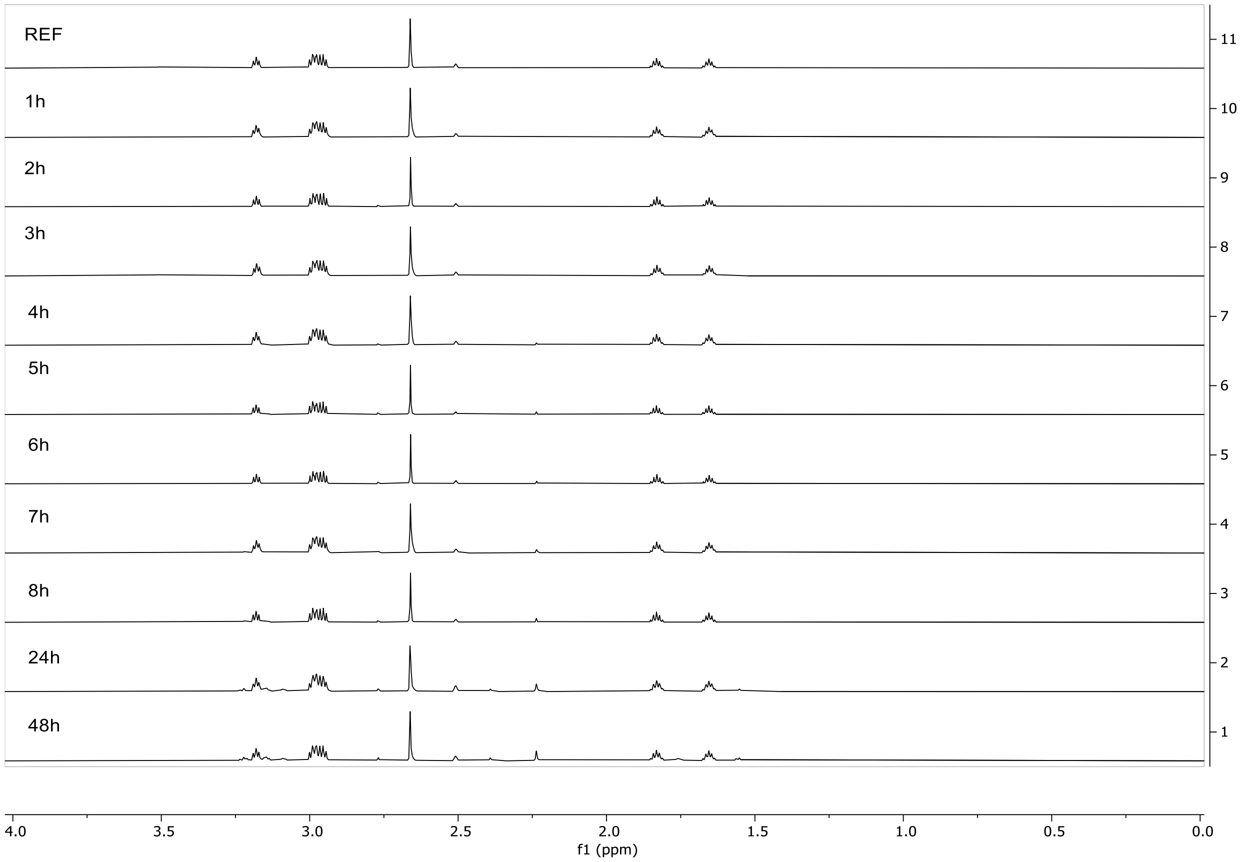

Figure S1 ^{1}H NMR of 1 molar equivalent of mTBD, 0.2 molar equivalent of water.

Structure	Time	Integral N-Methyl	Integral HP1	Integral HP2	% HP
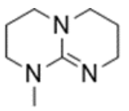 mTBD	REF	55505900	0	0	0.0
	1h	432726000	24281900	5402710	6.4
	2h	382962000	44287800	9591770	12.3
	3h	353905000	57975800	12797500	16.7
	4h	360509000	76657100	16114500	20.5
	5h	321356000	79733500	18498400	23.4
	6h	316410000	84717800	19811800	24.8
	7h	248739000	77712600	12070500	26.5
	8h	297765000	93827300	21977800	28.0
	24h	224439000	140952000	31318800	43.4
	48h	209958000	175707000	40719100	50.8

Table S2 1 molar equivalent of mTBD, 0.5 molar equivalent of water.

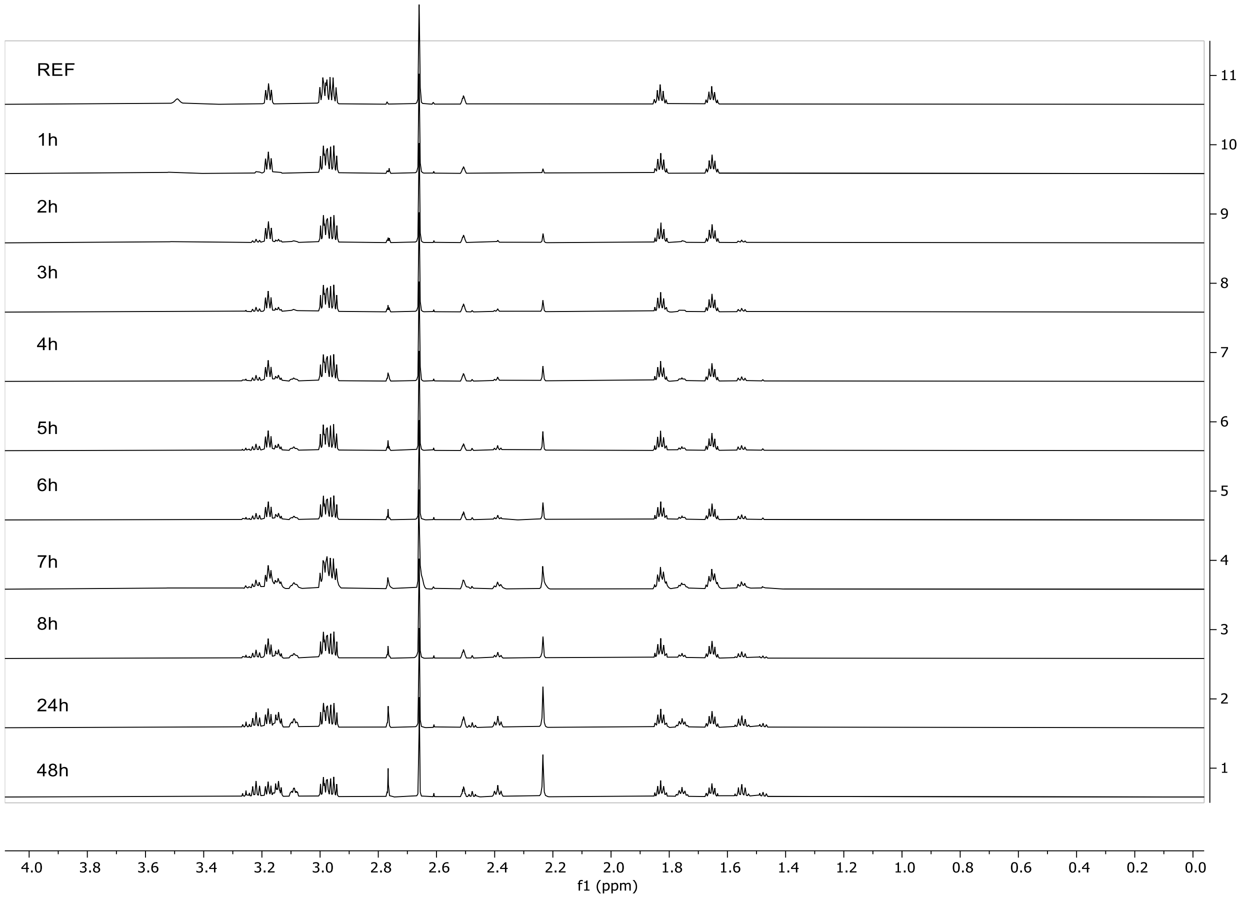

Figure S2 ^{1}H NMR of 1 molar equivalent of mTBD, 0.5 molar equivalent of water.

Structure	Time	Integral N-Methyl	Integral HP1	Integral HP2	% HP
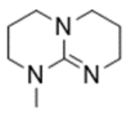 mTBD	REF	488012000.00	0.00	0.00	0.0
	1h	339497000.00	60526600.00	13630500.00	17.9
	2h	281363000.00	87612200.00	22069500.00	28.0
	3h	275186000.00	115816000.00	31145400.00	34.8
	4h	249984000.00	130420000.00	35548600.00	39.9
	5h	243647000.00	145682000.00	39494800.00	43.2
	6h	237275000.00	155514000.00	40574100.00	45.2
	7h	203880000.00	150965000.00	31341800.00	47.2
	8h	215027000.00	169739000.00	43800000.00	49.8
	24h	154476000.00	207946000.00	55822100.00	63.1
	48h	155054000.00	231181000.00	61705600.00	65.4

Table S3 1 molar equivalent of mTBD, 1 molar equivalent of water.

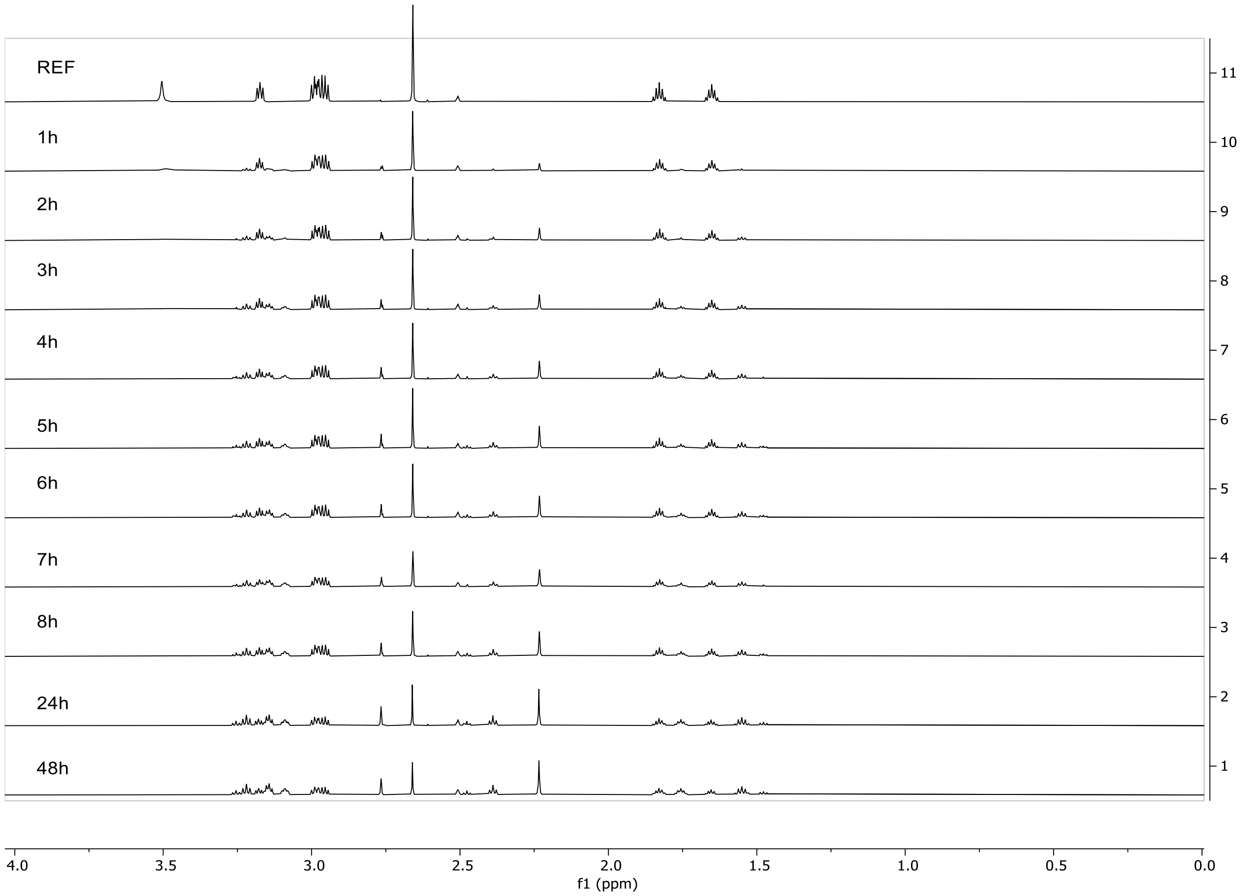

Figure S3 ¹H NMR 1 molar equivalent of mTBD, 1 molar equivalent of water.

Structure	Time	Integral N-Methyl	Integral HP1	Integral HP2	% HP
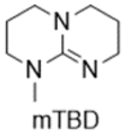 mTBD	REF	319508000.00	12907100.00	486676.00	4.0
	1h	1784990.00	286694000.00	33128300.00	99.4
	2h	1904400.00	292968000.00	34981200.00	99.4
	3h	1468770.00	303739000.00	38314700.00	99.6
	4h	922116.00	293973000.00	38033800.00	99.7
	5h	802720.00	280725000.00	36908200.00	99.7
	6h	719068.00	282919000.00	36688300.00	99.8
	7h	635588.00	282686000.00	36223300.00	99.8
	8h	721727.00	311021000.00	39786100.00	99.8
	24h	470263.00	282201000.00	36416700.00	99.9
	48h	307018.00	284222000.00	34331200.00	99.9

Table S4 1 molar equivalent of mTBD, 5 molar equivalents of water.

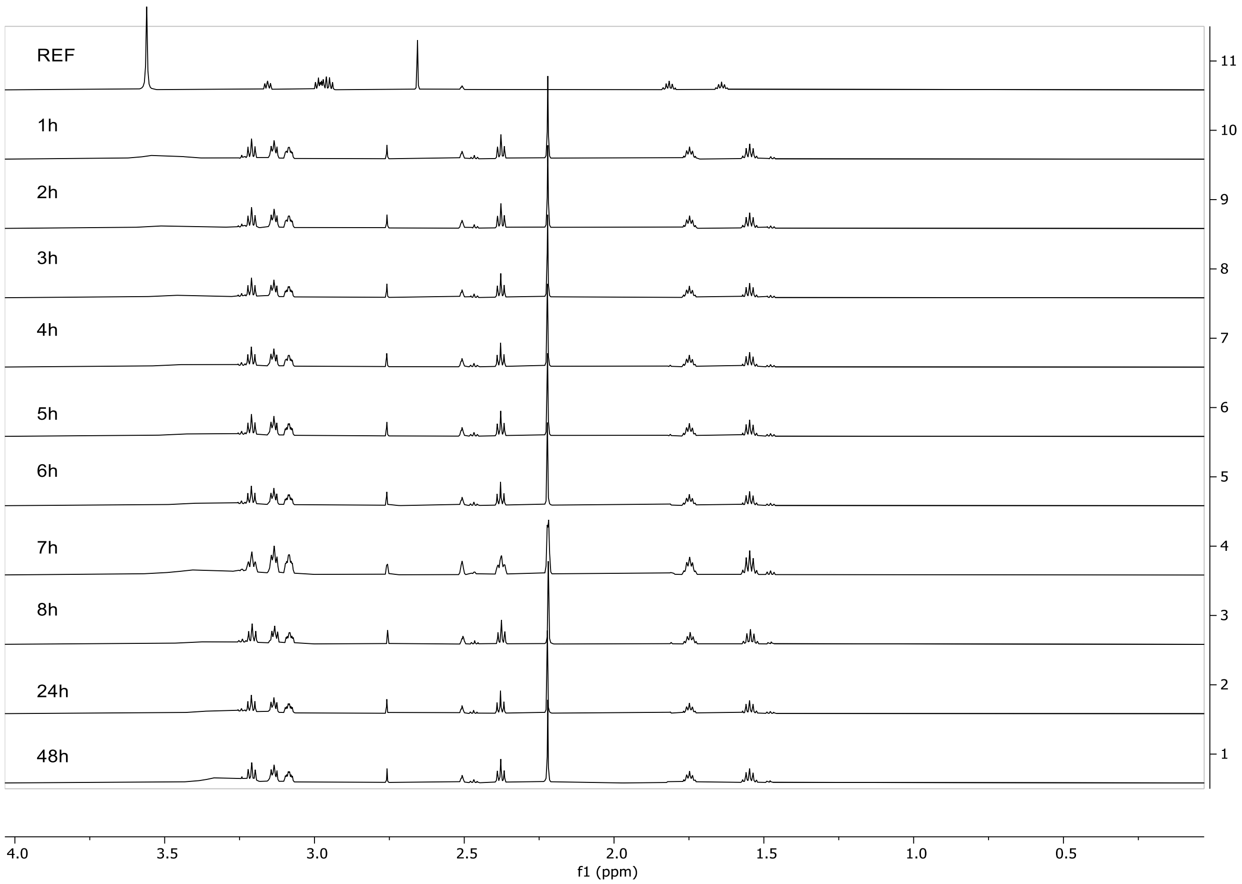

Figure S4 ^{1}H NMR 1 molar equivalent of mTBD, 5 molar equivalents of water.

Structure	Time	Integral N-Methyl	Integral HP1	Integral HP2	% HP
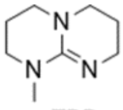 mTBD	REF	185453000.00	8978320.00	1039620.00	5.1
	1h	1041550.00	164633000.00	15298100.00	99.4
	2h	589013.00	193732000.00	20095100.00	99.7
	3h	357591.00	202954000.00	21903500.00	99.8
	4h	243435.00	203951000.00	22190000.00	99.9
	5h	184426.00	210316000.00	22924900.00	99.9
	6h	211775.00	198142000.00	19663200.00	99.9
	7h	175614.00	175746000.00	16558400.00	99.9
	8h	155521.00	202636000.00	21424400.00	99.9
	24h	415807.00	318701000.00	31550900.00	99.9
	48h	348365.00	326064000.00	31225400.00	99.9

Table S5 1 molar equivalent of mTBD, 10 molar equivalents of water.

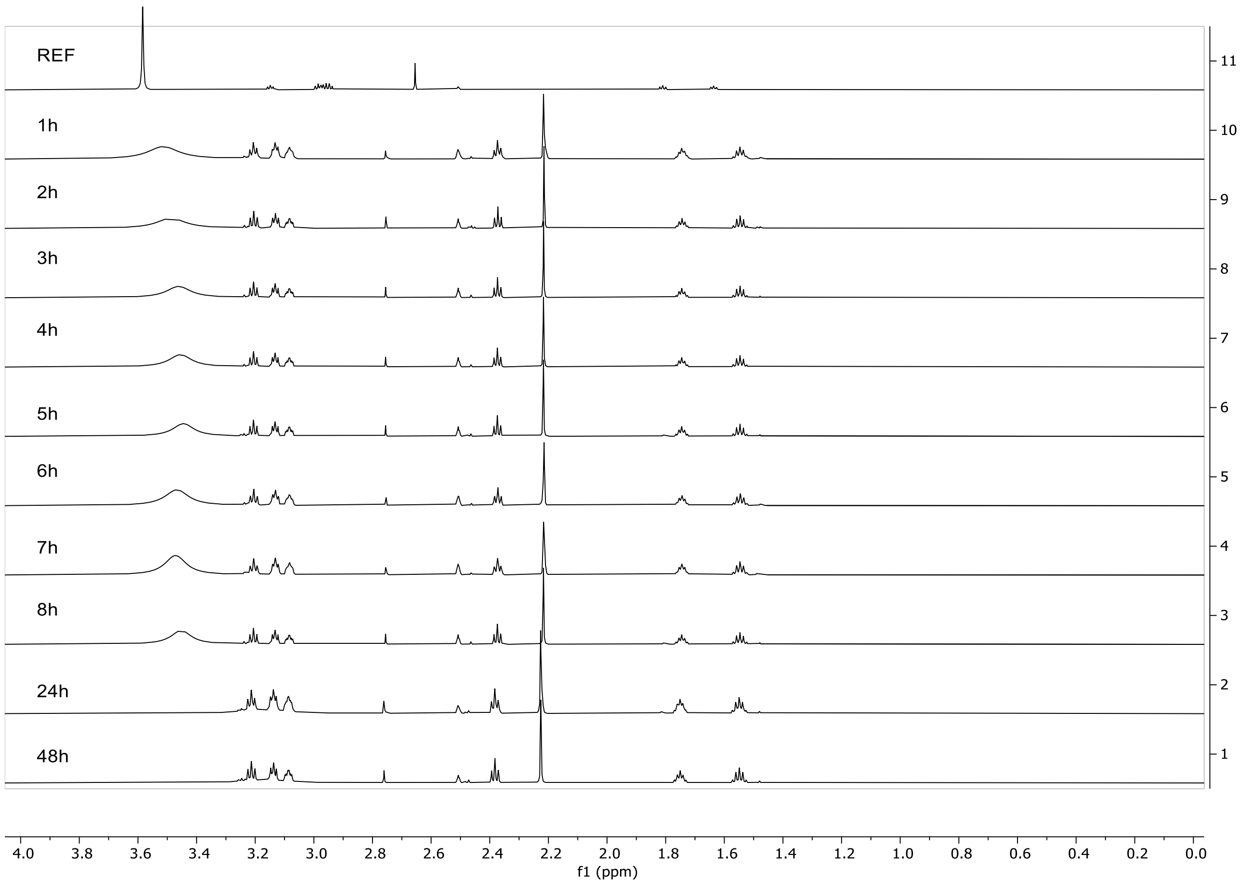

Figure S5 ^{1}H NMR 1 molar equivalent of mTBD, 10 molar equivalents of water.

b. mTBD + water at 70°C over 12 days

Structure	Time	Integral N-Methyl	Integral HP1	Integral HP2	% HP
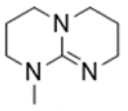 mTBD	3 days	25412900.00	51286900.00	23882800.00	74.7
	6 days	13696500.00	53197700.00	23546900.00	84.9
	9 days	9231670.00	50629300.00	22330900.00	88.8
	12 days	7484990.00	51172800.00	22413700.00	90.8

Table S6 1 molar equivalent of mTBD, 1 molar equivalent of water.

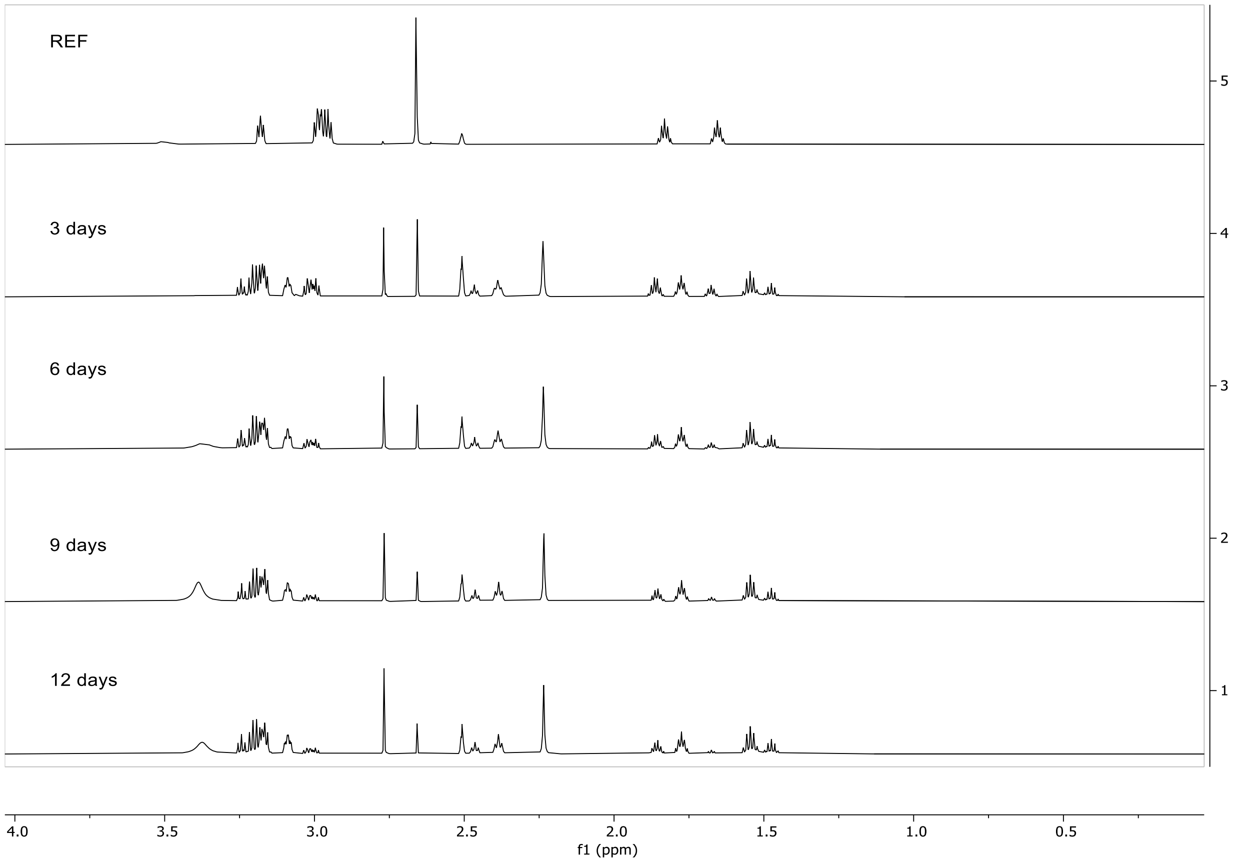

Figure S6 ^{1}H NMR 1 molar equivalent of mTBD, 1 molar equivalent of water.

Structure	Time	Integral N-Methyl	Integral HP1	Integral HP2	% HP
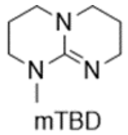 mTBD	3 days	1607800.00	66834000.00	10215700.00	98.0
	6 days	1253720.00	56638900.00	8501620.00	98.1
	9 days	1250030.00	60028500.00	9134640.00	98.2
	12 days	1090490.00	55367300.00	8371800.00	98.3

Table S7 1 molar equivalent of mTBD, 5 molar equivalents of water.

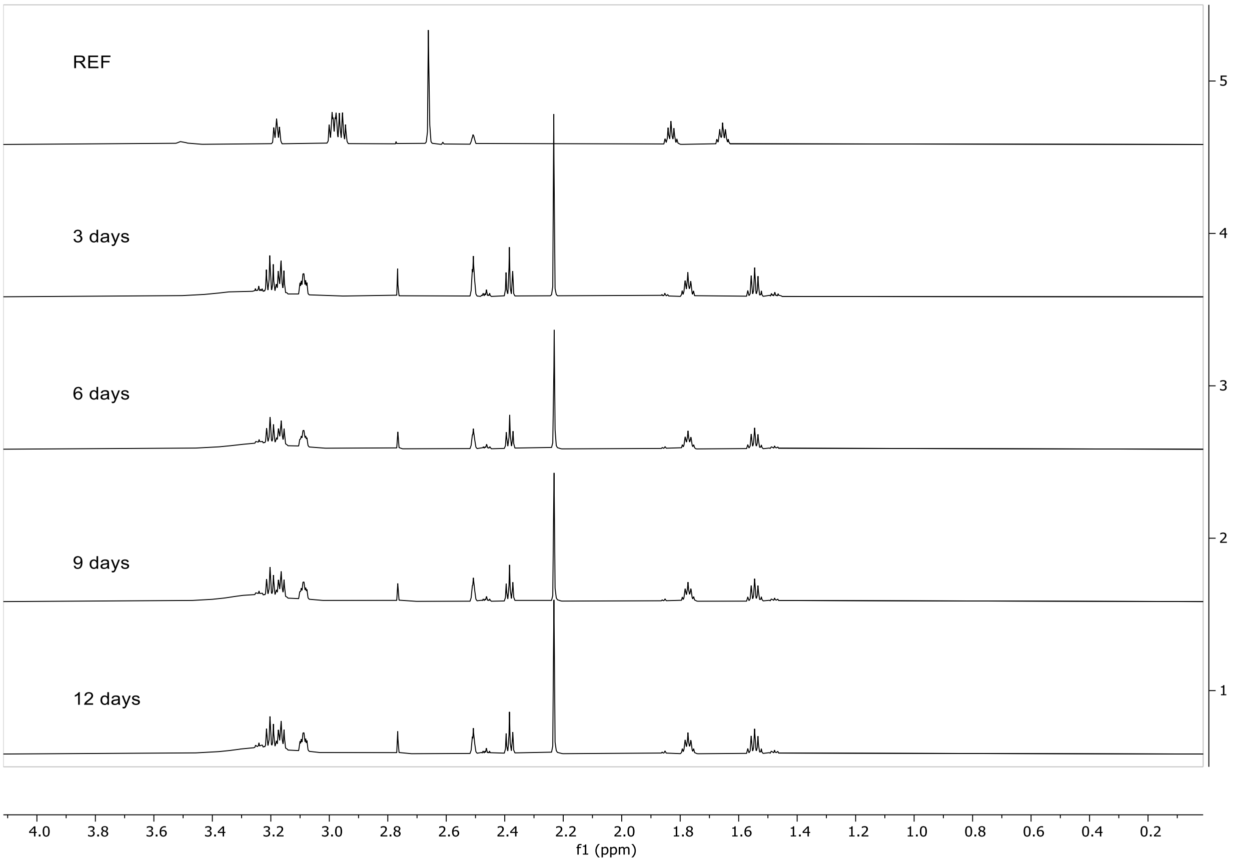

Figure S7 ^{1}H NMR 1 molar equivalent of mTBD, 5 molar equivalents of water.

Structure	Time	Integral N-Methyl	Integral HP1	Integral HP2	% HP
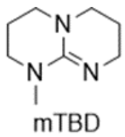 mTBD	3 days	0.00	33879300.00	3440250.00	100.0
	6 days	0.00	32486700.00	3612440.00	100.0
	9 days	0.00	43720700.00	4180940.00	100.0
	12 days	0.00	38301600.00	3354290.00	100.0

Table S8 1 molar equivalent of mTBD, 10 molar equivalents of water.

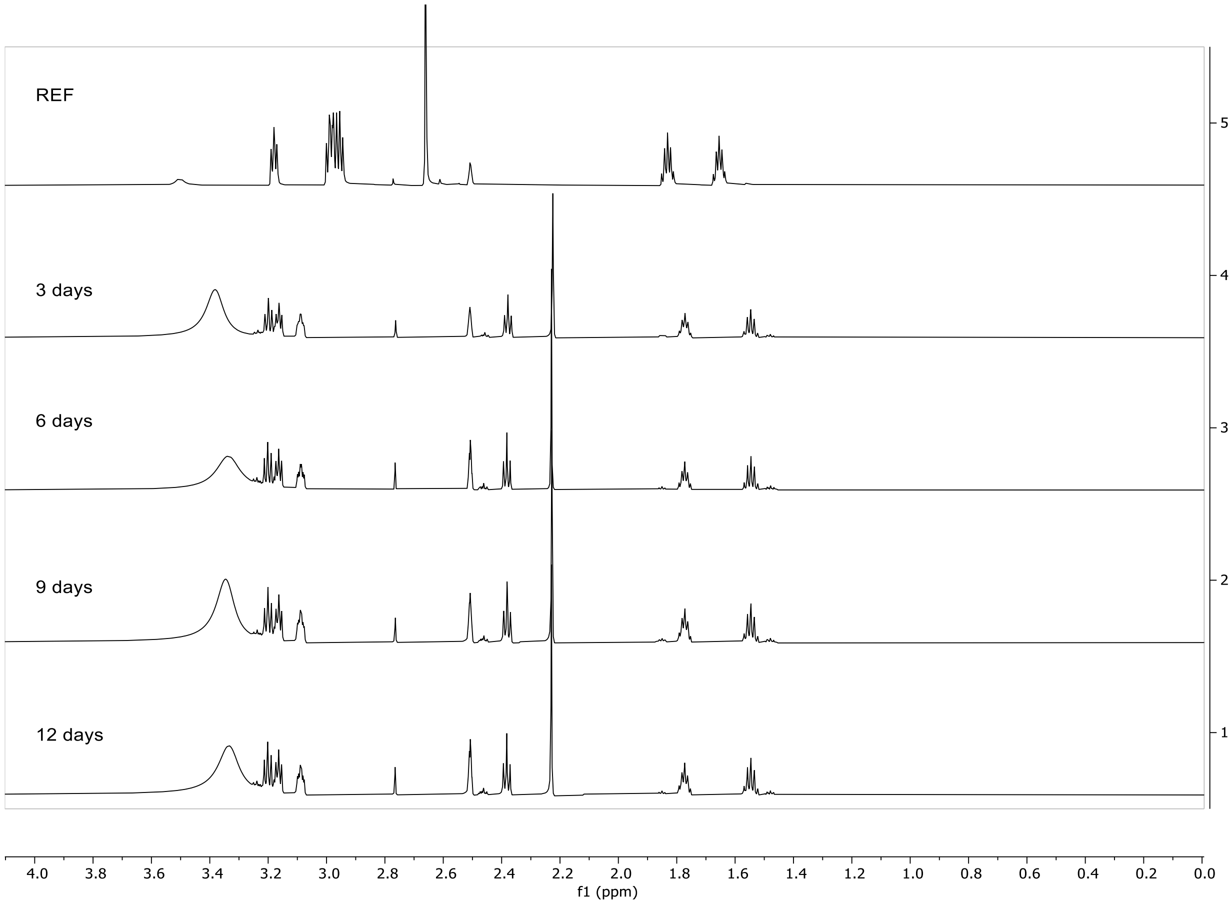

Figure S8 ^{1}H NMR 1 molar equivalent of mTBD, 10 molar equivalents of water.

c. mTBD + water at 90°C over 12 days ii

Structure	Time	Integral N-Methyl	Integral HP1	Integral HP2	% HP
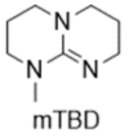	3 days	5611830.00	63214100.00	22555800.00	93.9
	6 days	2818780.00	68185600.00	24201800.00	97.0
	9 days	1429390.00	59225600.00	21150500.00	98.3
	12 days	1253480.00	74068200.00	26649700.00	98.8

Table S9 1 molar equivalent of mTBD, 1 molar equivalent of water.

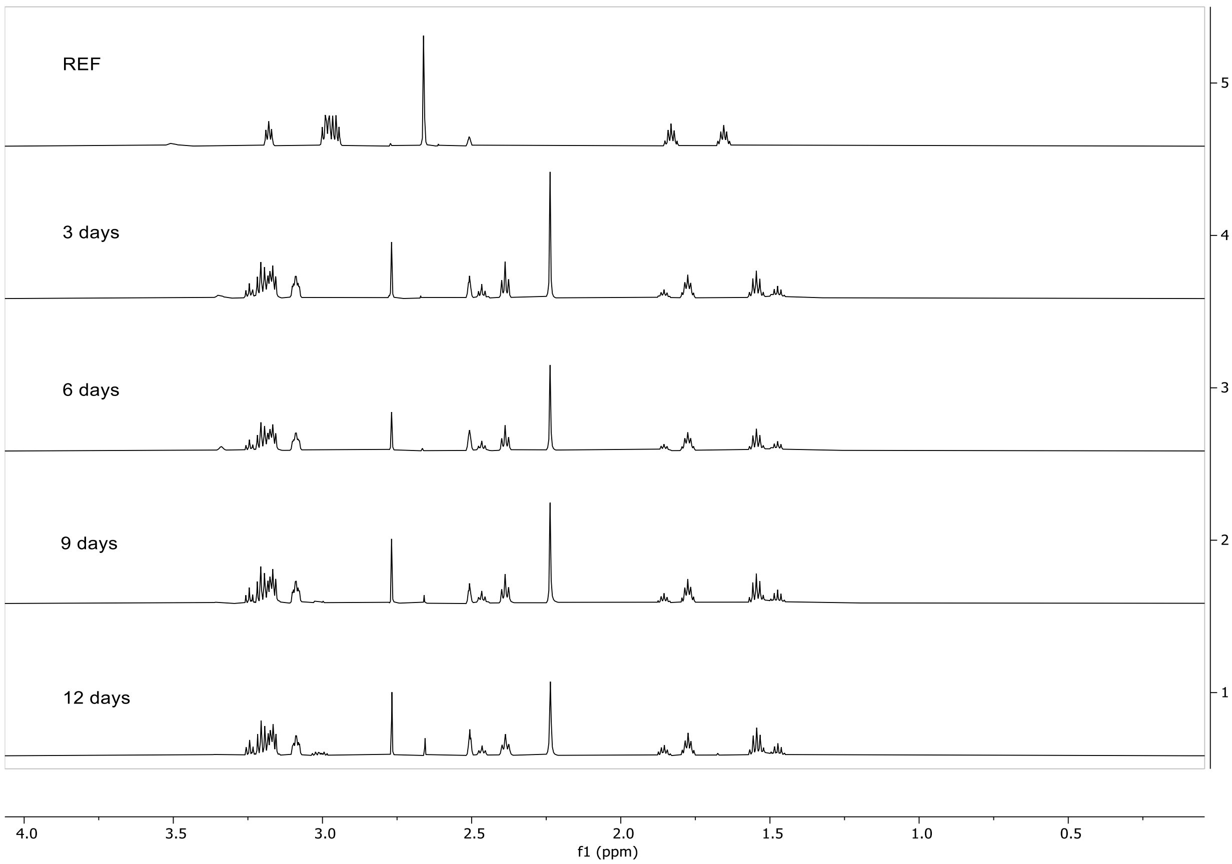

Figure S9 ^{1}H NMR 1 molar equivalent of mTBD, 1 molar equivalent of water.

Structure	Time	Integral N-Methyl	Integral HP1	Integral HP2	% HP
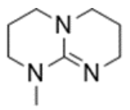 mTBD	3 days	0.00	47127000.00	8310870.00	100.0
	6 days	0.00	48657200.00	8385540.00	100.0
	9 days	0.00	52552900.00	9431320.00	100.0
	12 days	0.00	48272300.00	8594880.00	100.0

Table S10 1 molar equivalent of mTBD, 5 molar equivalents of water.

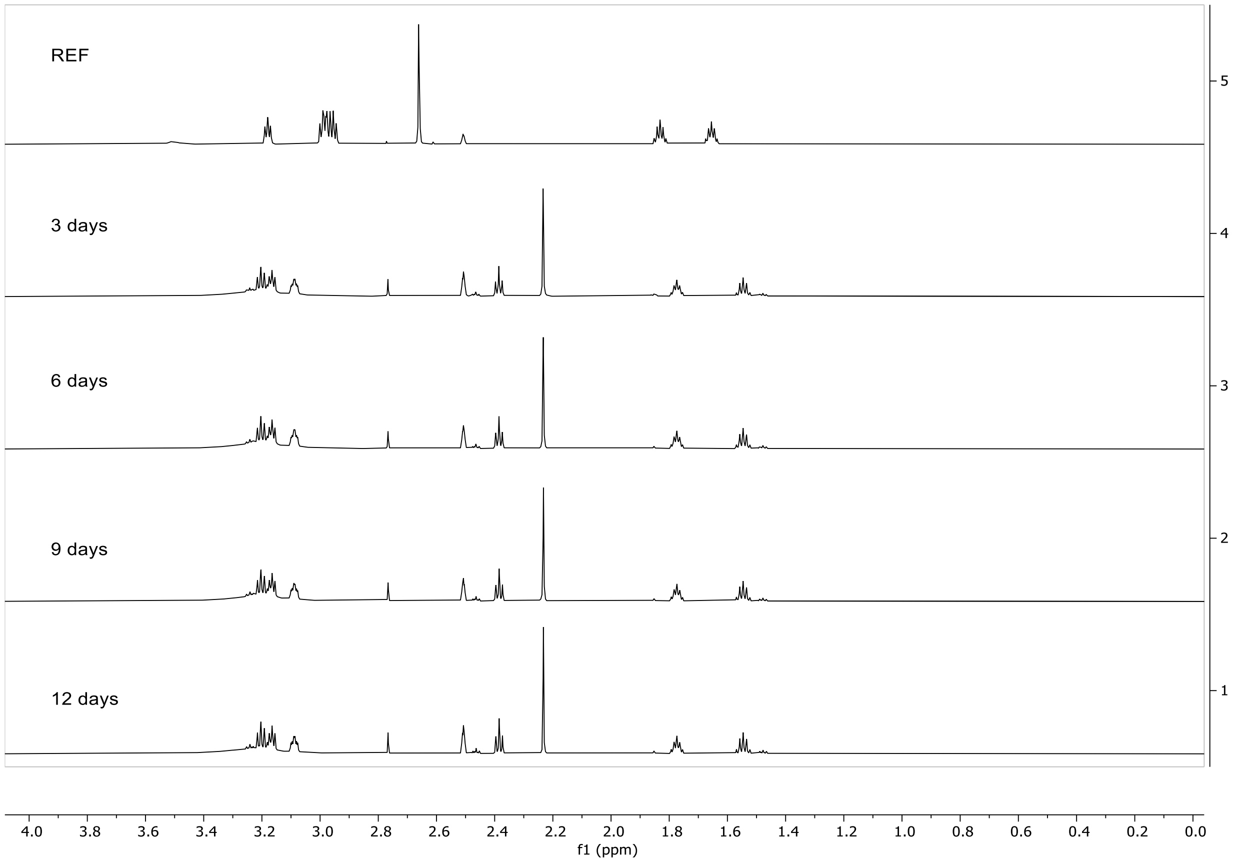

Figure S10 ^{1}H NMR 1 molar equivalent of mTBD, 5 molar equivalents of water.

Structure	Time	Integral N-Methyl	Integral HP1	Integral HP2	% HP
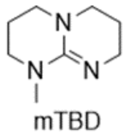	3 days	0.00	50046700.00	6751130.00	100.0
	6 days	0.00	45558200.00	6112740.00	100.0
	9 days	0.00	50808000.00	6892850.00	100.0
	12 days	0.00	47964500.00	6610730.00	100.0

Table S11 1 molar equivalent of mTBD, 10 molar equivalents of water.

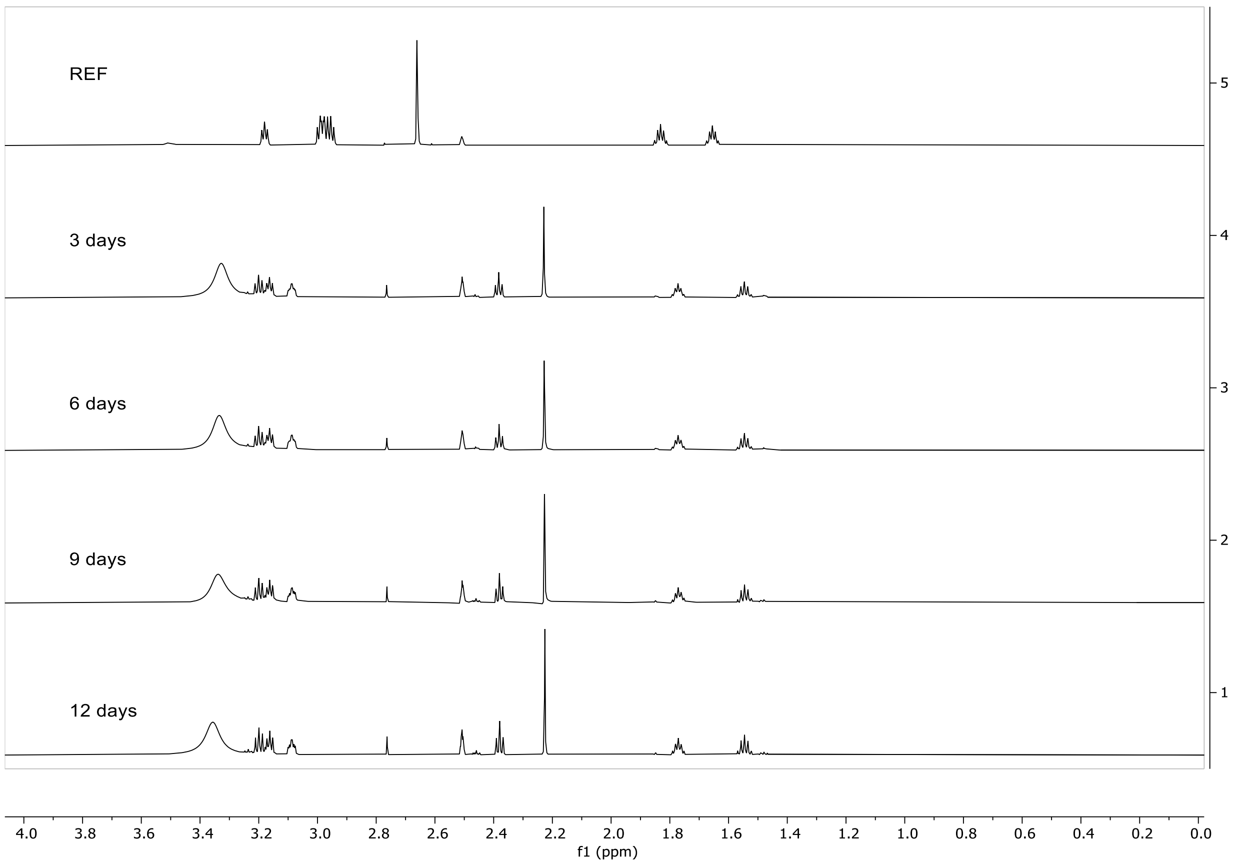

Figure S 11 ^{1}H NMR 1 molar equivalent of mTBD, 10 molar equivalents of water.

d. mTBD + water + DMSO

Structure	Time	Integral N-Methyl	Integral HP1	Integral HP2	% HP
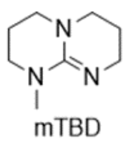	REF	80304400.00	313941.00	925844.00	0
	1h	90989700.00	13592800.00	12083500.00	22.00
	2h	80006200.00	19622800.00	12936900.00	28.92
	3h	73634200.00	23690100.00	13275700.00	33.42
	4h	68351700.00	27199100.00	14034100.00	37.62
	5h	56600700.00	26543600.00	12691600.00	40.93
	6h	41732100.00	26601800.00	11279900.00	47.58
	7h	44752400.00	30489400.00	12703900.00	49.11
	8h	45468900.00	33259900.00	13719200.00	50.81
	24h	30445800.00	47901200.00	16919300.00	68.04
	48h	23824700.00	60594500.00	20068200.00	77.19

Table S12 1 molar equivalent of mTBD, 1 molar equivalent of water and 0.2 molar equivalent of DMSO.

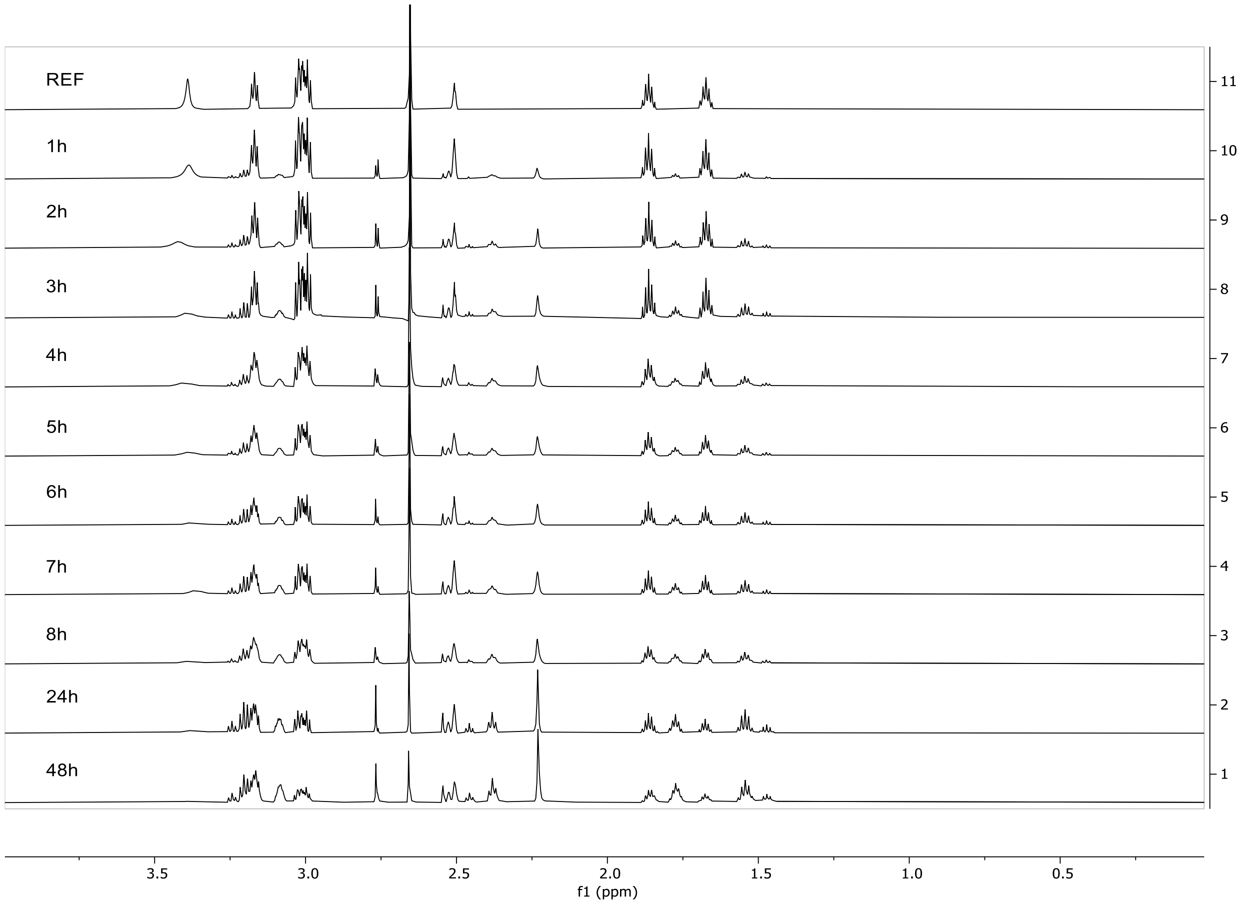

Figure S12 ¹H NMR 1 molar equivalent of mTBD, 1 molar equivalent of water and 0.2 molar equivalent of DMSO.

Structure	Time	Integral N-Methyl	Integral HP1	Integral HP2	% HP
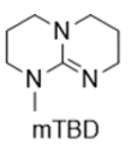	REF	74518400.00	354200.00	934315.00	0
	1h	86659600.00	7764060.00	9478300.00	16.59
	2h	69258400.00	9985840.00	8642000.00	21.19
	3h	66610600.00	13451800.00	9169030.00	25.35
	4h	67132800.00	16946200.00	10574500.00	29.07
	5h	56562900.00	17659100.00	9664520.00	32.57
	6h	51088300.00	21405000.00	10320900.00	38.30
	7h	65692200.00	29647000.00	13898000.00	39.86
	8h	44159800.00	21341500.00	9748930.00	41.31
	24h	28582000.00	32899200.00	11437500.00	60.80
	48h	25217800.00	47416400.00	15406100.00	71.36

Table S13 1 molar equivalent of mTBD, 1 molar equivalent of water and 0,5 molar equivalent of DMSO.

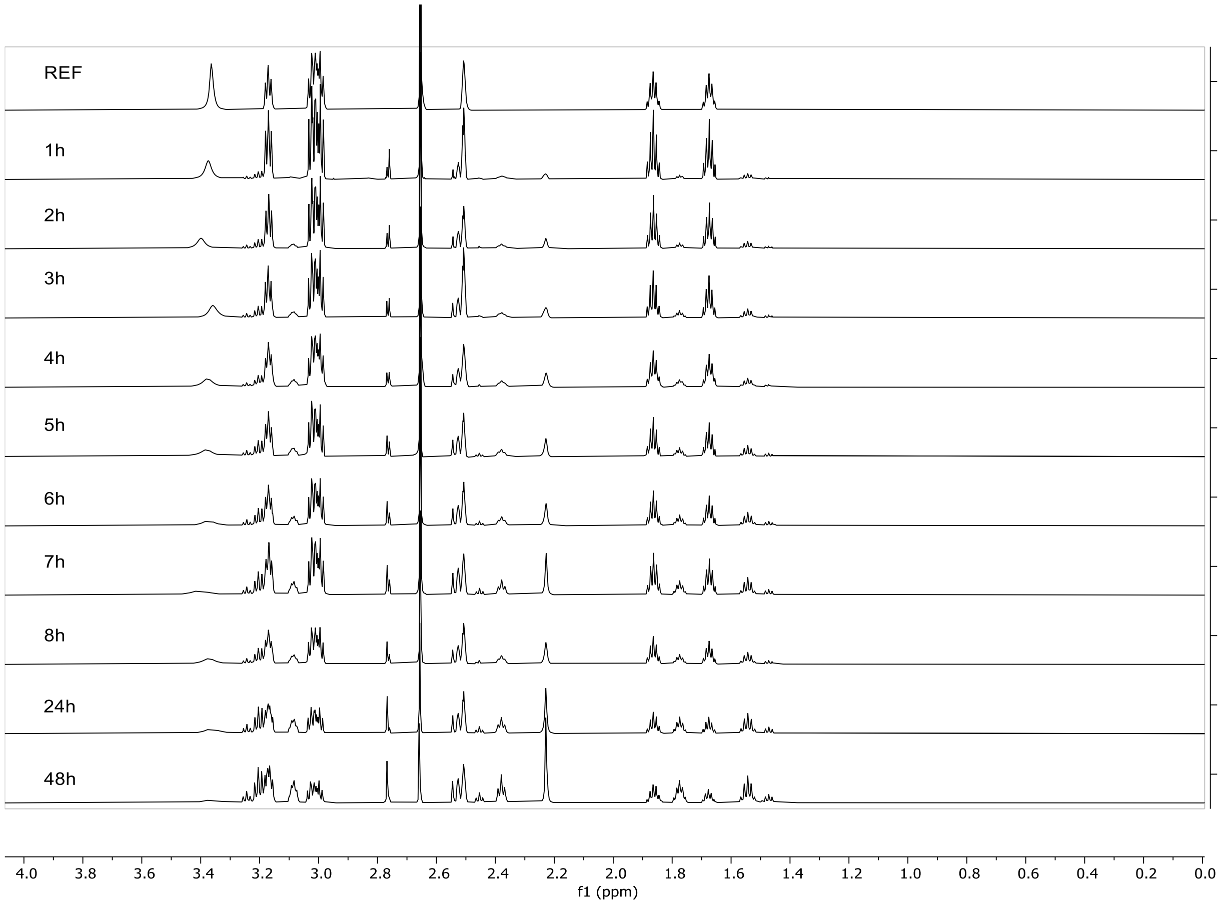

Figure S13 ¹H NMR 1 molar equivalent of mTBD, 1 molar equivalent of water and 0,5 molar equivalent of DMSO.

Structure	Time	Integral N-Methyl	Integral HP1	Integral HP2	% HP
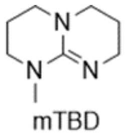 mTBD	REF	105297000.00	280451.00	1410160.00	0.00
	1h	70496500.00	3008510.00	6023000.00	11.36
	2h	83208800.00	6510630.00	8514220.00	15.30
	3h	82085300.00	8572670.00	8740020.00	17.42
	4h	61157500.00	8051330.00	7243850.00	20.01
	5h	71685800.00	11723800.00	9133510.00	22.54
	6h	67988000.00	15046500.00	9880550.00	26.83
	7h	62366100.00	15284200.00	9167420.00	28.16
	8h	54559800.00	14170400.00	8313820.00	29.18
	24h	44850900.00	31106600.00	11547800.00	48.74
	48h	37936800.00	41952100.00	13917400.00	59.56

Table S14 1 molar equivalent of mTBD, 1 molar equivalent of water and 1 molar equivalent of DMSO.

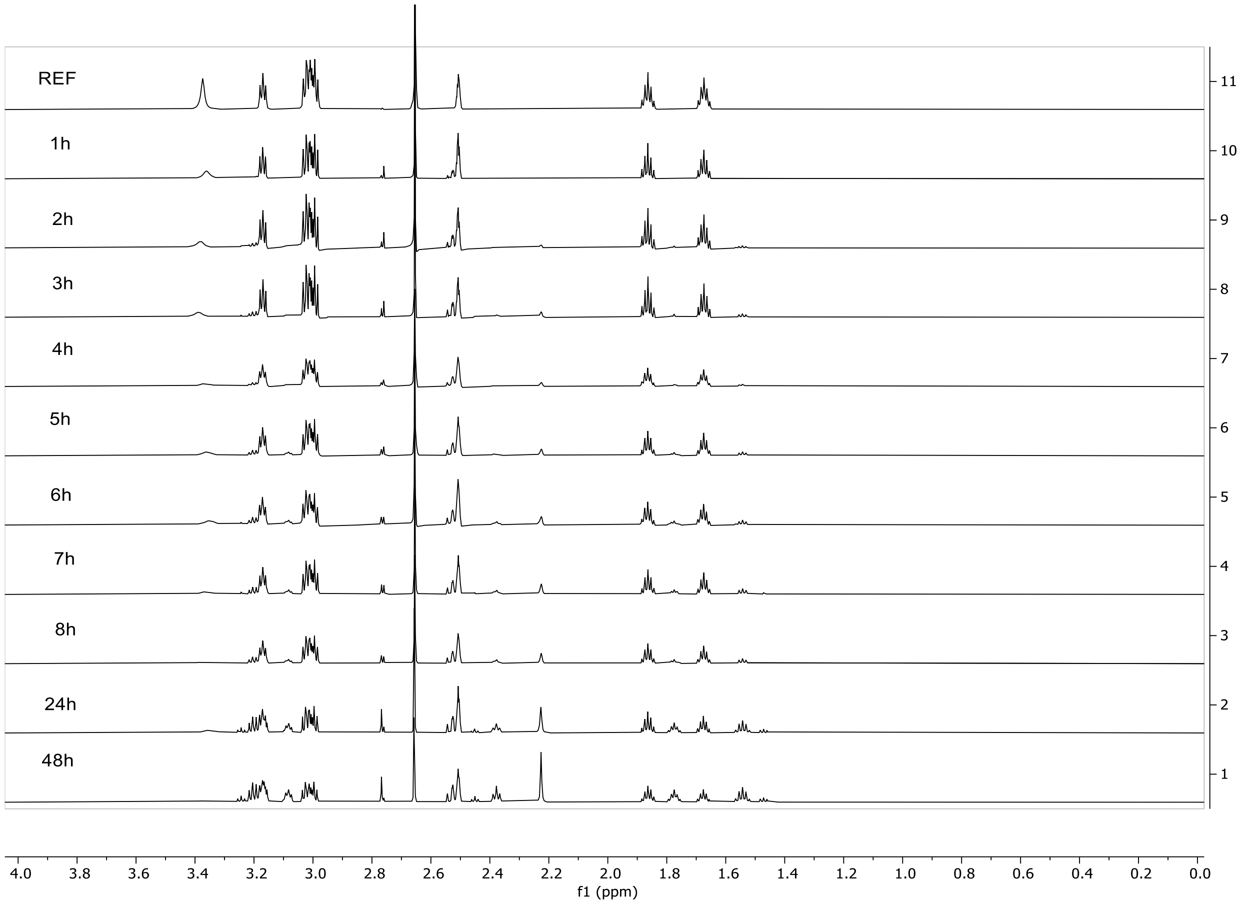

Figure S14 ^{1}H NMR 1 molar equivalent of mTBD, 1 molar equivalent of water and 1 molar equivalent of DMSO.

Structure	Time	integral N-Methyl	integral HP1	integral HP2	% HP
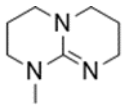 mTBD	REF	107735000.00	399917.00	1429700.00	0.00
	1h	57110400.00	1016910.00	3700660.00	7.63
	2h	69968300.00	2171010.00	5134850.00	9.45
	3h	79551300.00	3435100.00	6420420.00	11.02
	4h	73065200.00	4053290.00	6404430.00	12.52
	5h	66921800.00	4801000.00	6176480.00	14.09
	6h	66192300.00	6962910.00	6642940.00	17.05
	7h	66146900.00	7626960.00	7068510.00	18.18
	8h	69419200.00	8766100.00	7521310.00	19.00
	24h	52975100.00	19011600.00	8405880.00	34.10
	48h	47498100.00	29096300.00	10817300.00	45.66

Table S15 1 molar equivalent of mTBD, 1 molar equivalent of water and 2 molar equivalents of DMSO.

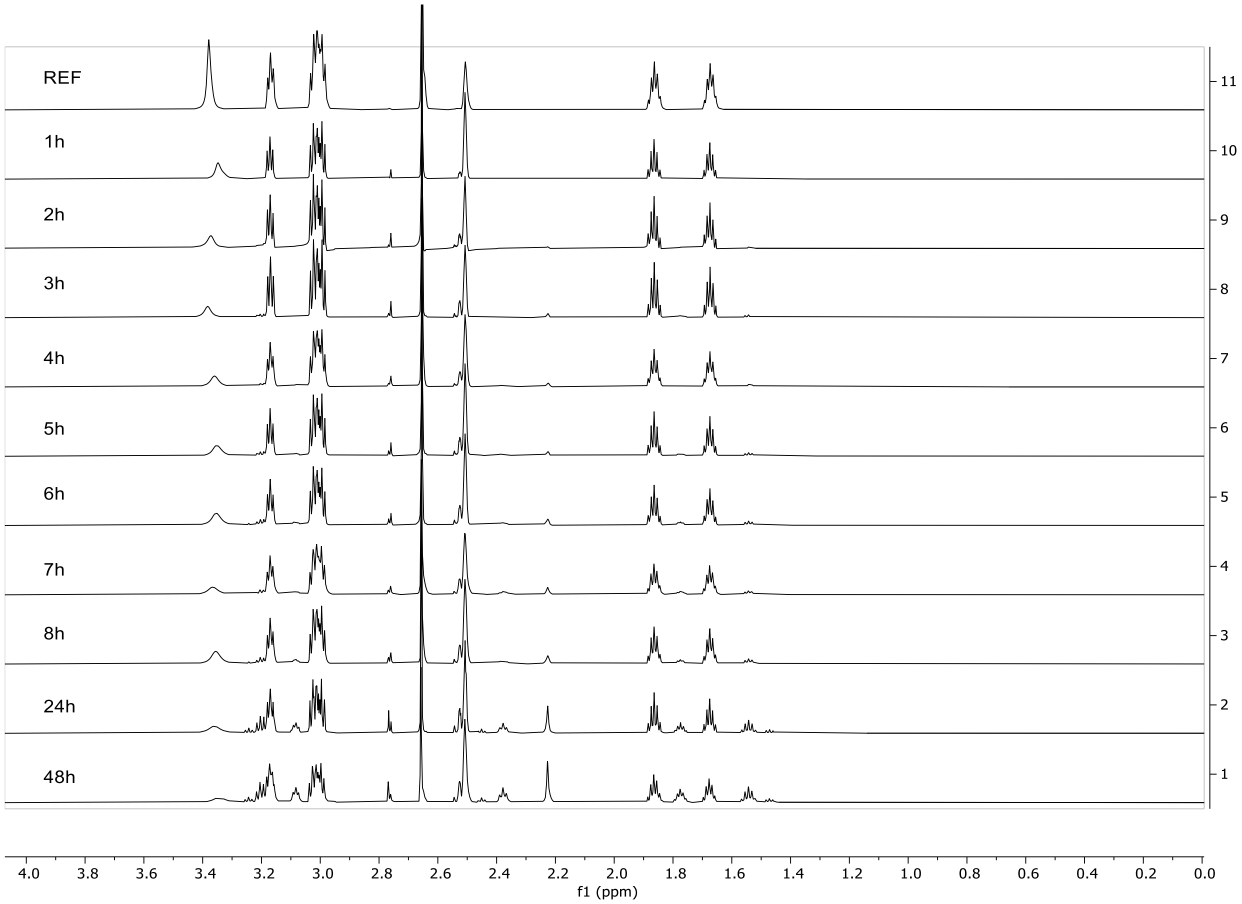

Figure S15 ^{1}H NMR 1 molar equivalent of mTBD, 1 molar equivalent of water and 2 molar equivalents of DMSO.

e. mTBD + water + ethylene glycol

Structure	Time	integral N-Methyl	integral HP1	integral HP2	% HP
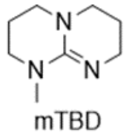	REF	101637000.00	2338860.00	1886920.00	0.00
	1h	59518200.00	19753300.00	5130870.00	29.48
	2h	55699800.00	30313100.00	7804940.00	40.63
	3h	50190100.00	36400400.00	9640300.00	47.84
	4h	44738400.00	37813300.00	10400900.00	51.87
	5h	40273100.00	38969200.00	10745700.00	55.25
	6h	35088600.00	37114800.00	9623170.00	57.12
	7h	34798900.00	41089800.00	11427000.00	60.15
	8h	31488500.00	39794000.00	10868700.00	61.67
	24h	16679200.00	58613300.00	15440500.00	81.62
	48h	10852200.00	57332500.00	16473500.00	87.18

Table S16 1 molar equivalent of mTBD, 1 molar equivalent of water and 0.2 molar equivalent of ethylene glycol.

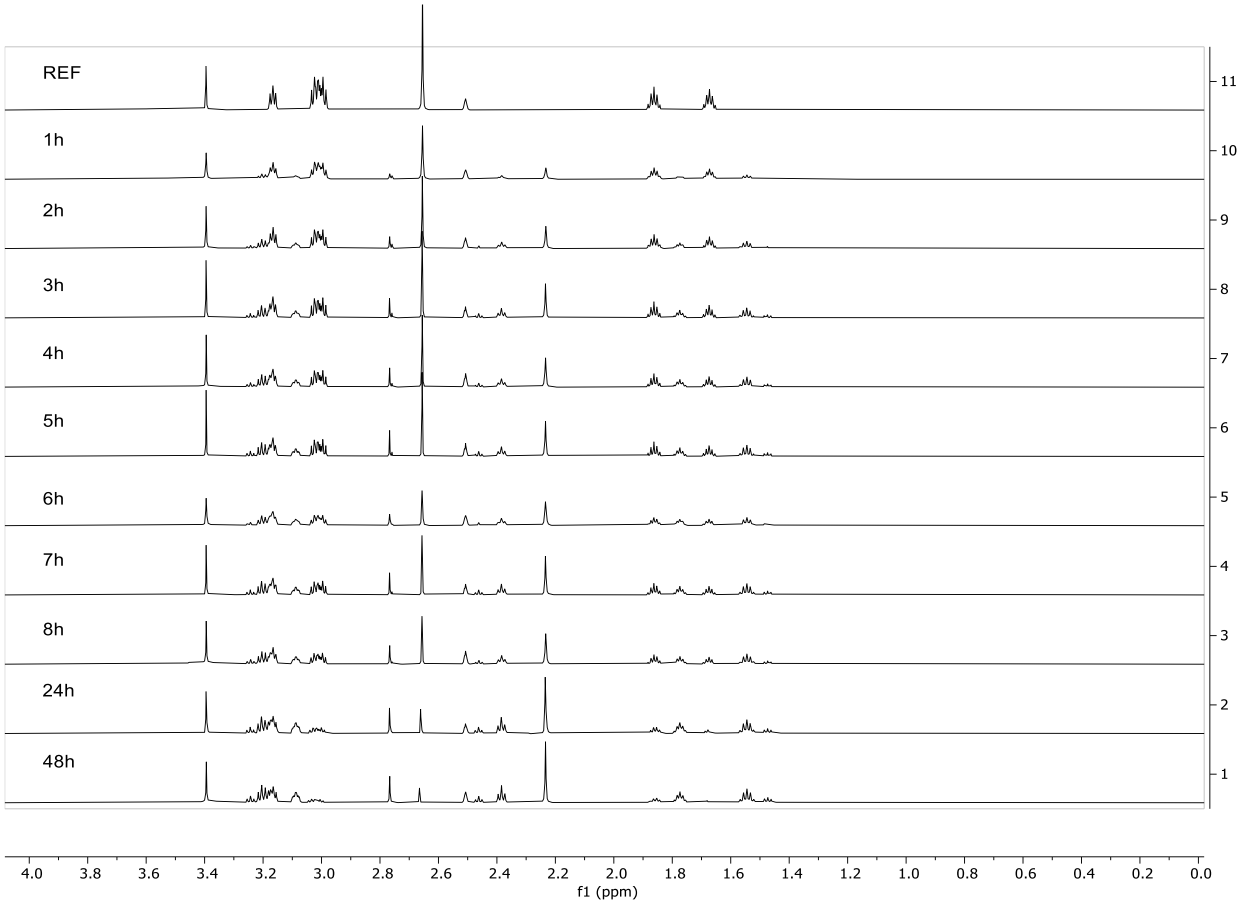

Figure S16 ^{1}H NMR 1 molar equivalent of mTBD, 1 molar equivalent of water and 0.2 molar equivalent of ethylene glycol.

Structure	Time	Integral N-Methyl	Integral HP1	Integral HP2	% HP
	REF	83272500.00	831256.00	958695.00	2.10
	1h	32020800.00	22846600.00	4938070.00	46.46
	2h	36391200.00	40042300.00	8794700.00	57.30
	3h	32608100.00	45712400.00	9875800.00	63.03
	4h	22586400.00	37223900.00	8134390.00	66.76
	5h	23820300.00	43920300.00	9878540.00	69.31
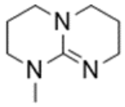 mTBD	6h	23906800.00	47765100.00	10645700.00	70.96
	7h	20459200.00	45431600.00	10438700.00	73.20
	8h	20016300.00	49185100.00	10847800.00	75.00
	24h	9530260.00	63227300.00	14880300.00	89.13
	48h	5975190.00	64834000.00	15373300.00	93.07

Table S17 1 molar equivalent of mTBD, 1 molar equivalent of water and 0.5 molar equivalent of ethylene glycol.

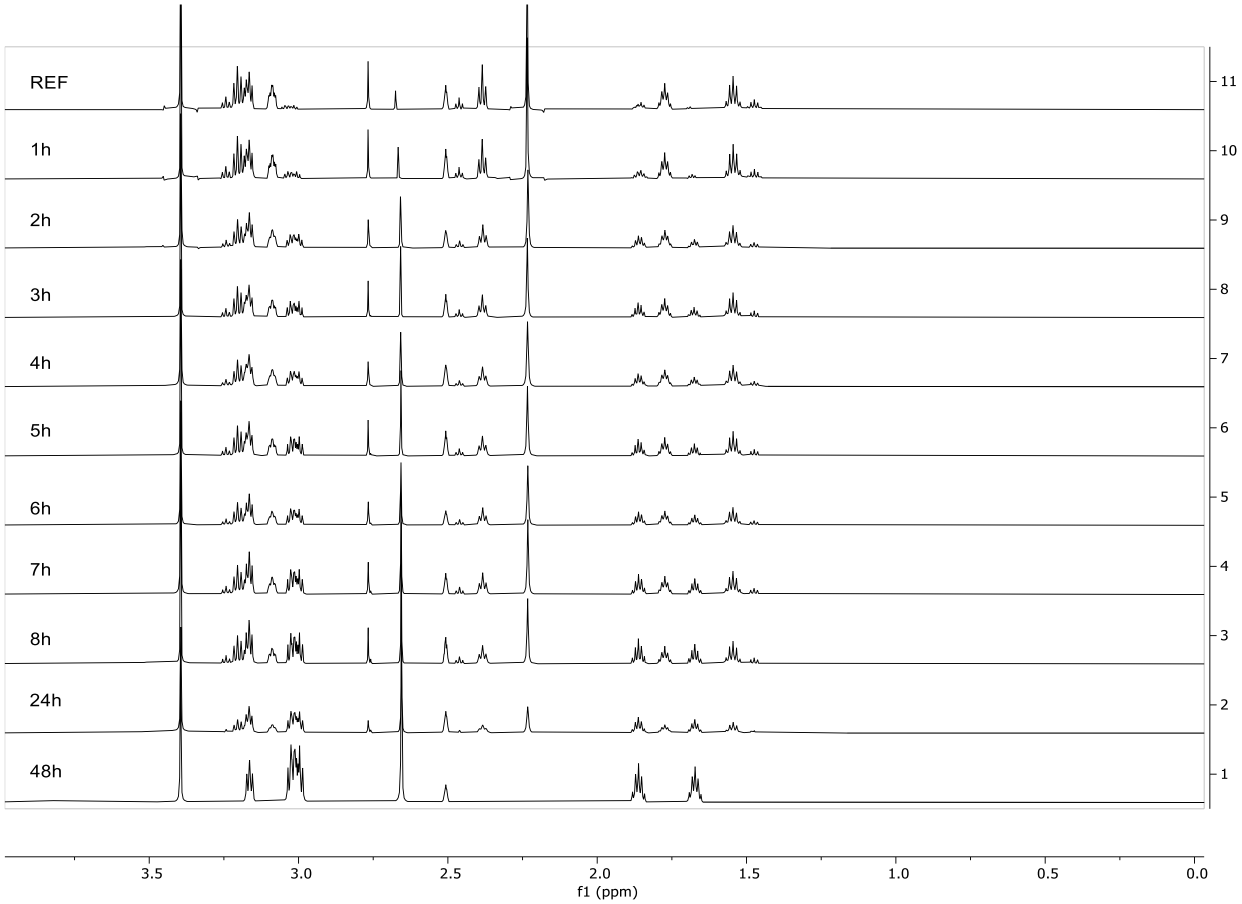

Figure S17 ^{1}H NMR 1 molar equivalent of mTBD, 1 molar equivalent of water and 0.5 molar equivalent of ethylene glycol.

Structure	Time	Integral N-Methyl	Integral HP1	Integral HP2	% HP
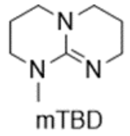	REF	160593000.00	317402.00	2463730.00	0.00
	1h	29693000.00	28232300.00	7249260.00	54.44
	2h	19119400.00	31574400.00	7153250.00	66.95
	3h	21063700.00	46420300.00	10199700.00	72.89
	4h	13086400.00	37191100.00	7781090.00	77.46
	5h	10885000.00	36031700.00	7546230.00	80.01
	6h	14245400.00	54007400.00	11095400.00	82.05
	7h	13134200.00	55408200.00	11350300.00	83.56
	8h	10997600.00	50958200.00	10384400.00	84.80
	24h	3994760.00	63598000.00	12642600.00	95.02
	48h	1957590.00	57009300.00	11296500.00	97.21

Table S18 1 molar equivalent of mTBD, 1 molar equivalent of water and 1 molar equivalent of ethylene glycol.

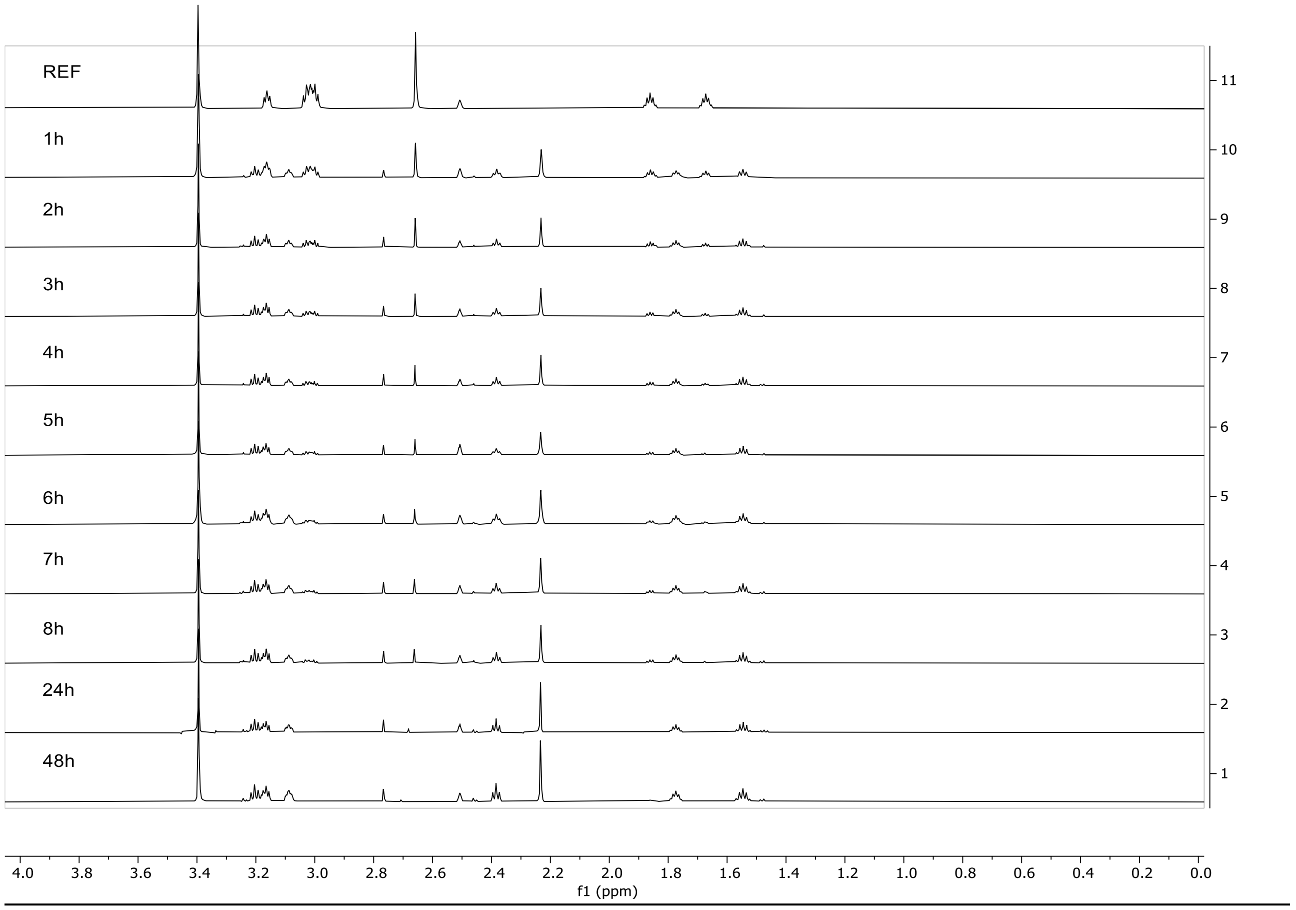

Figure S18 ^{1}H NMR 1 molar equivalent of mTBD, 1 molar equivalent of water and 1 molar equivalent of ethylene glycol.

Structure	Time	Integral N-Methyl	Integral HP1	Integral HP2	% HP
	REF	53764100.00	261020.00	587134.00	0.00
	1h	26655400.00	29669200.00	5278650.00	56.73
	2h	18458900.00	34995500.00	6127640.00	69.02
	3h	13176700.00	34909200.00	5978740.00	75.63
	4h	12591000.00	41913100.00	7239520.00	79.61
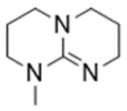	5h	8116280.00	30694500.00	5186900.00	81.55
mTBD	6h	10398700.00	44845800.00	7550200.00	83.44
	7h	10843900.00	51075500.00	8905970.00	84.69
	8h	8664980.00	42866100.00	7556850.00	85.34
	24h	2814770.00	45432000.00	7662220.00	94.97
	48h	1254010.00	40111400.00	6901790.00	97.40

Table S19 1 molar equivalent of mTBD, 1 molar equivalent of water and 2 molar equivalents of ethylene glycol.

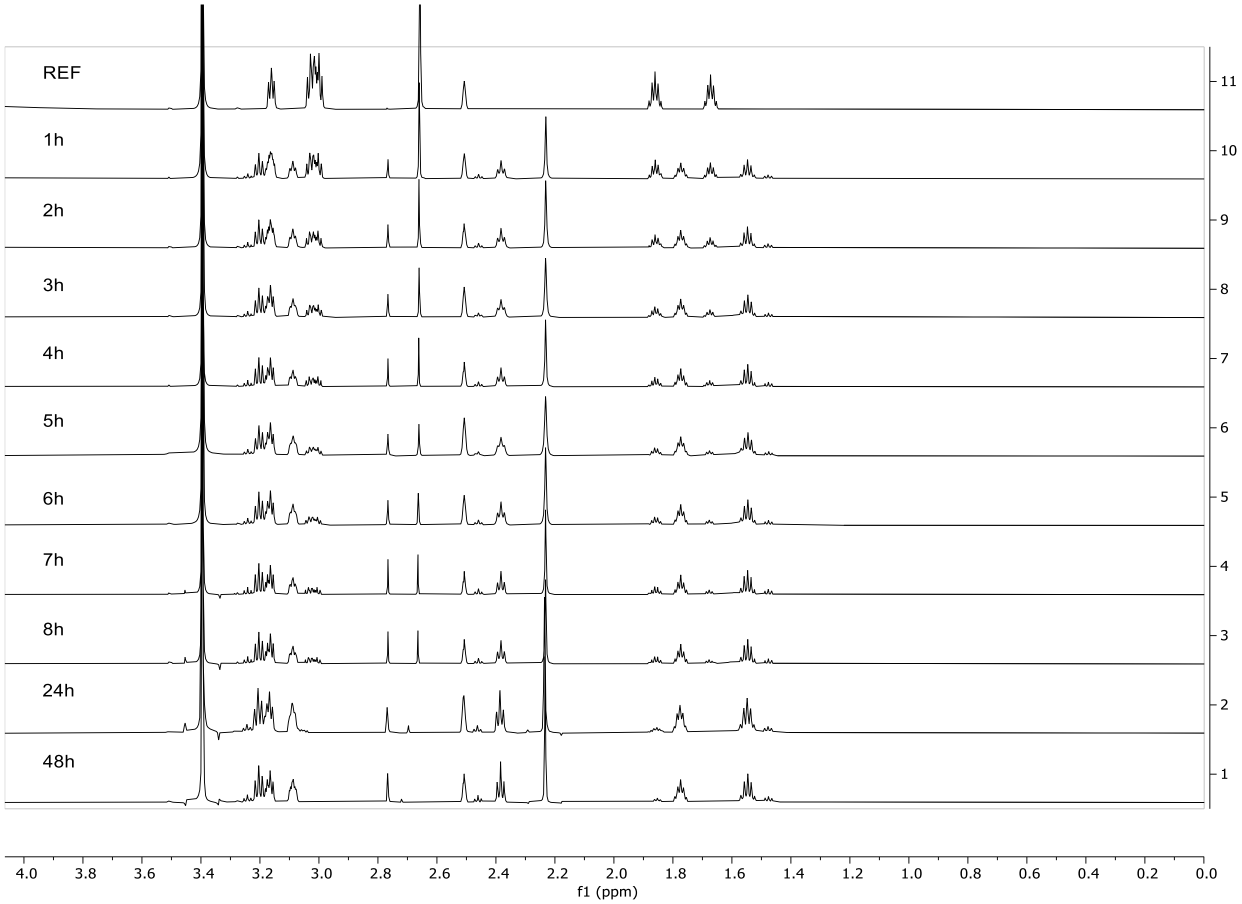

Figure S19 ^{1}H NMR 1 molar equivalent of mTBD, 1 molar equivalent of water and 2 molar equivalents of ethylene glycol.

f. mTBD + water + phenol

Structure	Time	Integral N-Methyl	Integral HP1	Integral HP2	% HP
	REF	188431000.00	255583.00	186864.00	0.00
	1h	132630000.00	38859000.00	7659510.00	25.97
	2h	51049600.00	30841400.00	5742480.00	41.75
	3h	48355200.00	40225900.00	8551800.00	50.22
	4h	33690300.00	37526700.00	8104810.00	57.53
	5h	29636600.00	38827600.00	8244820.00	61.36
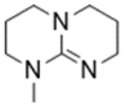 mTBD	6h	30819200.00	46108400.00	9650150.00	64.40
	7h	29817300.00	51218300.00	11240300.00	67.69
	8h	21423100.00	38784800.00	7393220.00	68.31
	24h	8100880.00	59035600.00	14278800.00	90.05
	48h	5124640.00	70387400.00	16983200.00	94.46

Table S20 1 molar equivalent of mTBD, 1 molar equivalent of water and 0.2 molar equivalents of phenol.

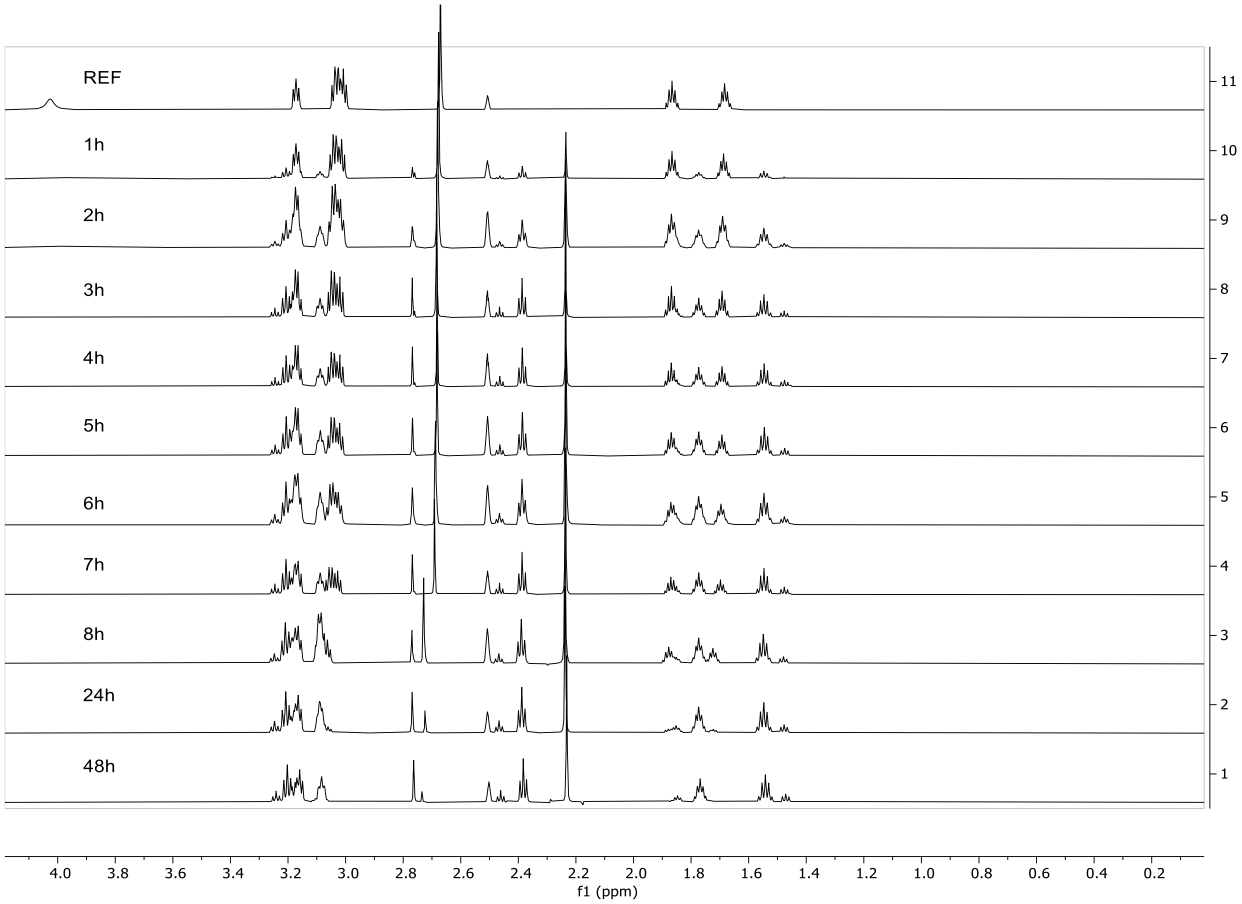

Figure S20 ^{1}H NMR 1 molar equivalent of mTBD, 1 molar equivalent of water and 0.2 molar equivalents of phenol.

Structure	Time	Integral N-Methyl	Integral HP1	Integral HP2	% HP
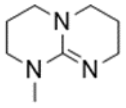 mTBD	REF	162782000.00	219307.00	182999.00	0.00
	1h	48431200.00	16193000.00	2806830.00	28.18
	2h	44145400.00	24692300.00	3722860.00	39.16
	3h	38600500.00	31492300.00	5852330.00	49.17
	4h	29971400.00	31680600.00	6185840.00	55.82
	5h	27823000.00	34598800.00	6864630.00	59.84
	6h	25372100.00	35882200.00	7311180.00	63.00
	7h	25967000.00	42331400.00	8584020.00	66.23
	8h	29051400.00	52059300.00	11628400.00	68.67
	24h	0.00	51846200.00	11324600.00	100.00
	48h	0.00	55558300.00	11922300.00	100.00

Table S21 1 molar equivalent of mTBD, 1 molar equivalent of water and 0.5 molar equivalent of phenol

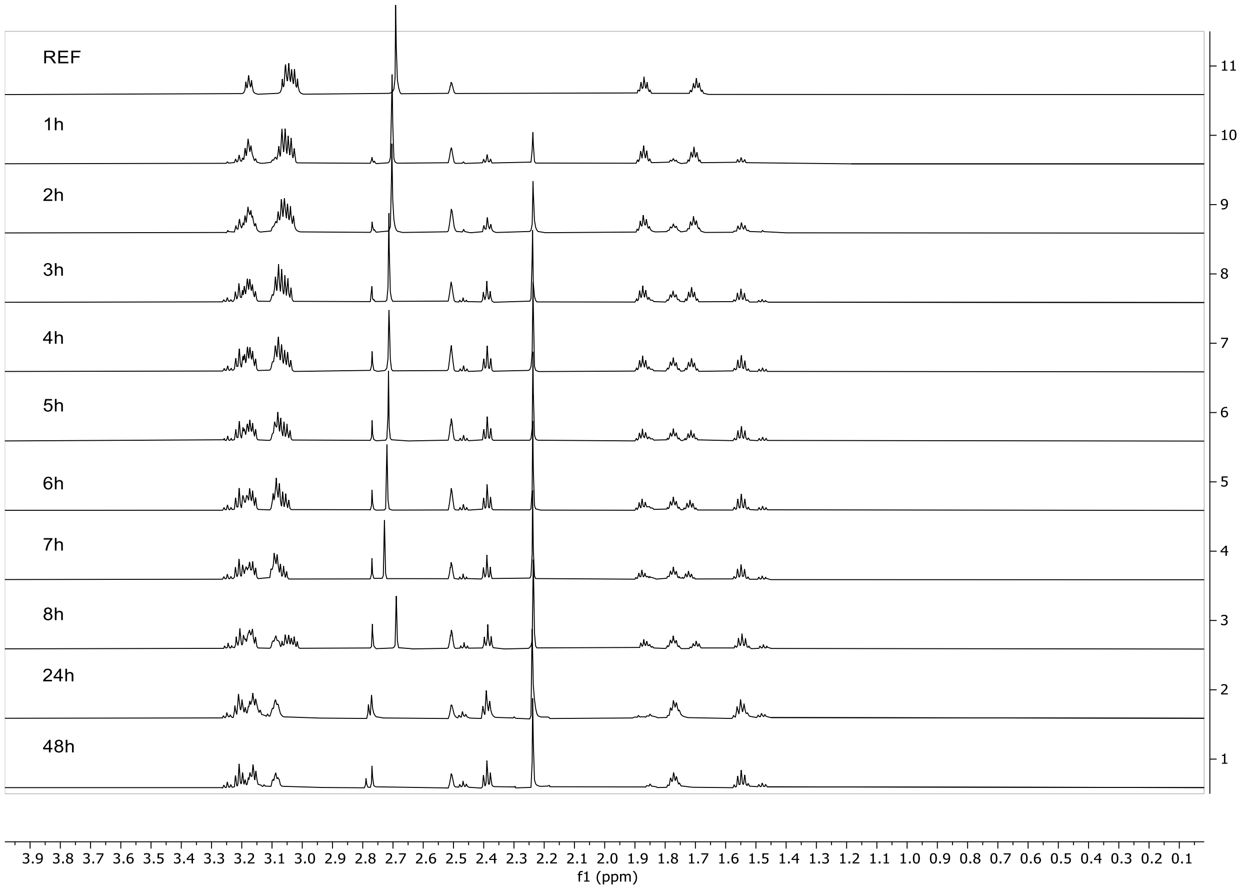

Figure S21 ^{1}H NMR 1 molar equivalent of mTBD, 1 molar equivalent of water and 0.5 molar equivalents of phenol.

.

Structure	Time	Integral N-Methyl	Integral HP1	Integral HP2	% HP
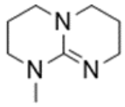 mTBD	REF	163954000.00	339939.00	85462.60	0.00
	1h	53853400.00	13723900.00	446666.00	20.83
	2h	58240400.00	24552600.00	167794.00	29.80
	3h	49454900.00	26742100.00	190070.00	35.26
	4h	35836000.00	23423800.00	212768.00	39.74
	5h	48031000.00	35764200.00	51059.50	42.72
	6h	42368200.00	34107500.00	109355.00	44.68
	7h	34769700.00	29574200.00	115970.00	46.06
	8h	31563000.00	29786700.00	220731.00	48.74
	24h	8777580.00	46883500.00	16073800.00	87.76
	48h	7675720.00	41188700.00	10633300.00	87.10

Table S22 1 molar equivalent of mTBD, 1 molar equivalent of water and 1 molar equivalents of phenol.

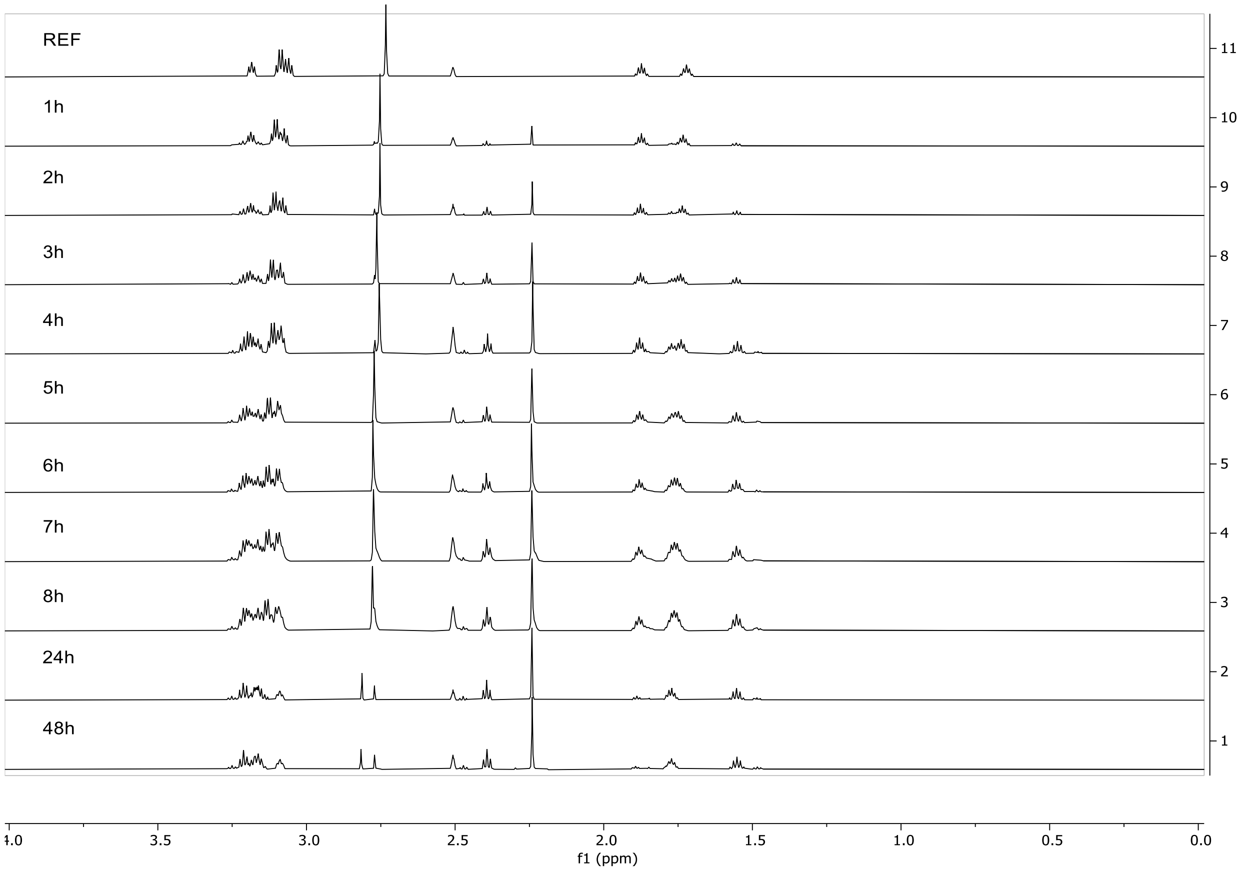

Figure S22 ^{1}H NMR 1 molar equivalent of mTBD, 1 molar equivalent of water and 1 molar equivalents of phenol.

Structure	Time	Integral N-Methyl	Integral HP1	Integral HP2	% HP
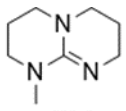 mTBD	REF	117958000.00	142888.00	137327.00	0.00
	1h	50833300.00	4570240.00	424332.00	8.95
	2h	49613900.00	7728280.00	426847.00	14.12
	3h	40225900.00	8427320.00	332492.00	17.88
	4h	44567000.00	11469700.00	402610.00	21.04
	5h	35819600.00	9947010.00	314668.00	22.27
	6h	34773700.00	10496500.00	465577.00	23.97
	7h	39862600.00	12803900.00	377254.00	24.85
	8h	29741700.00	10888400.00	9592870.00	40.78
	24h	3265000.00	23386600.00	30384800.00	94.28
	48h	1887130.00	27023400.00	26219600.00	96.58

Table S23 1 molar equivalent of mTBD, 1 molar equivalent of water and 2 molar equivalents of phenol.

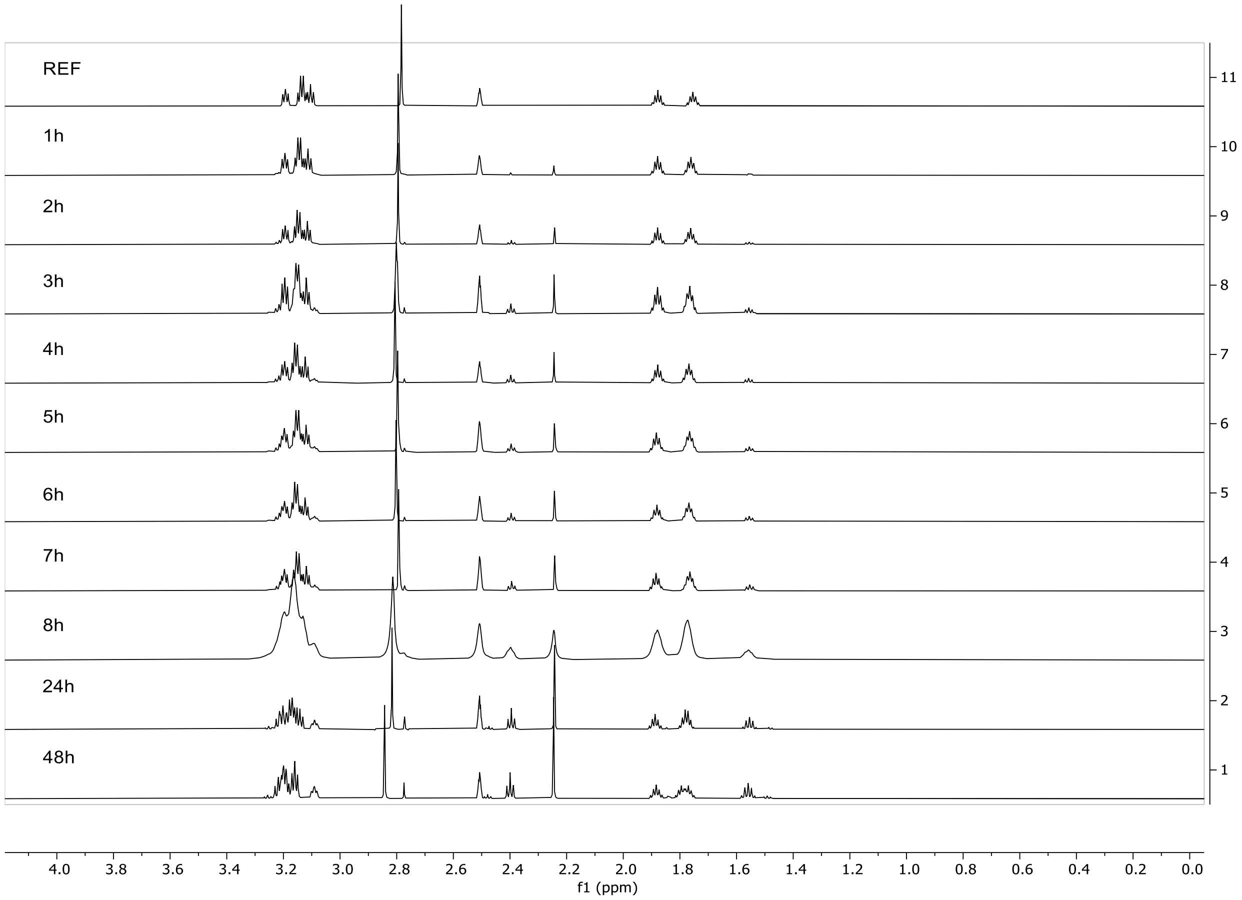

Figure S23 ^{1}H NMR 1 molar equivalent of mTBD, 1 molar equivalent of water and 2 molar equivalents of phenol.

g. mTBD + water + propanol

Structure	Time	Integral N-Methyl	Integral HP1	Integral HP2	% HP
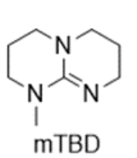	REF	85430900.00	345940.00	928759.00	0.00
	1h	75234200.00	13658100.00	10582000.00	24.37
	2h	66749700.00	18781300.00	11203600.00	31.00
	3h	64640700.00	25419700.00	12797000.00	37.16
	4h	43215600.00	21255300.00	9688740.00	41.73
	5h	51570500.00	28968600.00	12956600.00	44.84
	6h	45160700.00	34225000.00	13476800.00	51.37
	7h	43364200.00	35787200.00	14332100.00	53.61
	8h	37033400.00	33215200.00	12827900.00	55.42
	24h	26768200.00	45067300.00	16283800.00	69.62
	48h	18241900.00	53787100.00	18602300.00	79.87

Table S24 1 molar equivalent of mTBD, 1 molar equivalent of water and 0.2 molar equivalent of propanol.

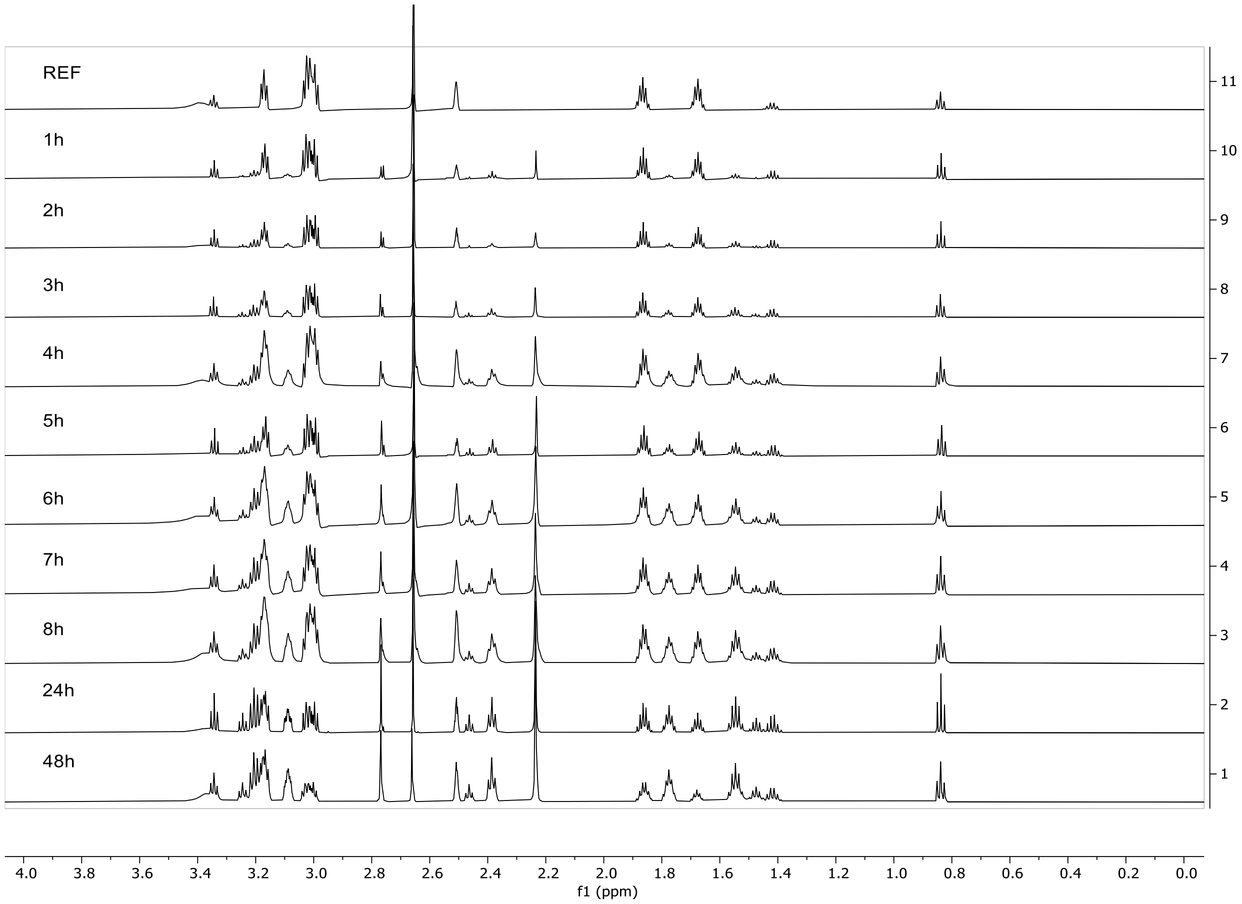

Figure S24 ^{1}H NMR 1 molar equivalent of mTBD, 1 molar equivalent of water and 0.2 molar equivalent of propanol.

Structure	Time	Integral N-Methyl	Integral HP1	integral HP2	% HP
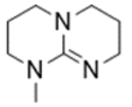 mTBD	REF	86001600.00	307232.00	1113340.00	1.62
	1h	57181800.00	12656400.00	8135950.00	26.67
	2h	57651600.00	19631500.00	10139100.00	34.05
	3h	49275700.00	22488300.00	10259800.00	39.93
	4h	40196800.00	22559100.00	9712630.00	44.53
	5h	40156900.00	25810900.00	10744100.00	47.65
	6h	41106700.00	34907100.00	13508000.00	54.08
	7h	36083500.00	33025500.00	12507300.00	55.79
	8h	27963600.00	27425500.00	10311800.00	57.44
	24h	24647000.00	47484100.00	16773200.00	72.28
	48h	17188300.00	57324600.00	19882300.00	81.79

Table S25 1 molar equivalent of mTBD, 1 molar equivalent of water and 0.5 molar equivalent of propanol.

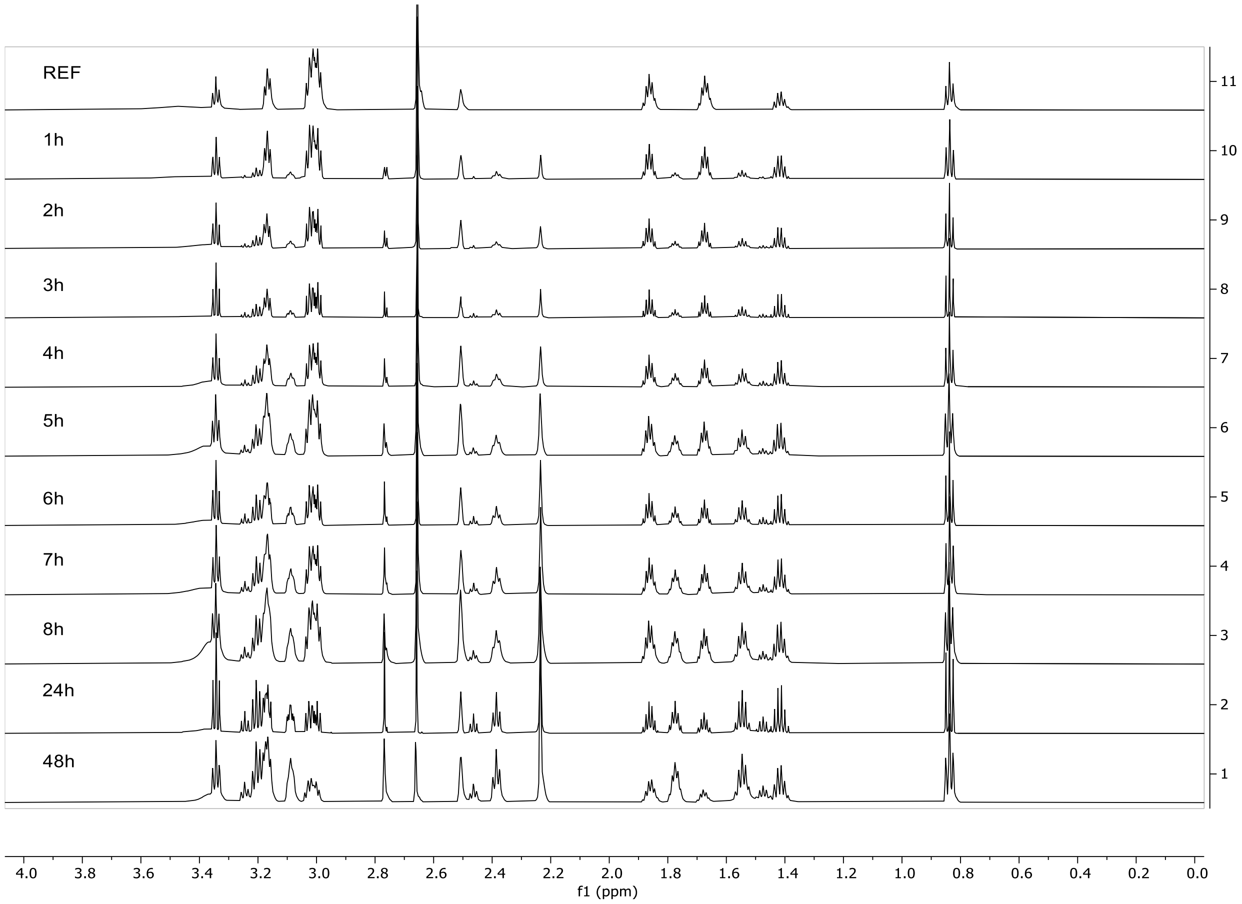

Figure S25 ^{1}H NMR 1 molar equivalent of mTBD, 1 molar equivalent of water and 0.5 molar equivalent of propanol.

Structure	Time	integral N-Methyl	integral HP1	integral HP2	% HP
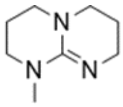 mTBD	REF	66660100.00	820591.00	1088040.00	2.78
	1h	41485800.00	11356400.00	5621920.00	29.04
	2h	40938700.00	16390600.00	6873700.00	36.24
	3h	44516900.00	23715500.00	8943150.00	42.32
	4h	29320000.00	18807400.00	6682380.00	46.51
	5h	36031200.00	26451700.00	9210580.00	49.74
	6h	31819100.00	31090000.00	10147300.00	56.45
	7h	27842900.00	29445800.00	9441930.00	58.28
	8h	24725300.00	28236500.00	8955950.00	60.07
	24h	13948200.00	31545300.00	9570610.00	74.67
	48h	11717300.00	47344500.00	14151900.00	84.00

Table S26 1 molar equivalent of mTBD, 1 molar equivalent of water and 1 molar equivalent of propanol.

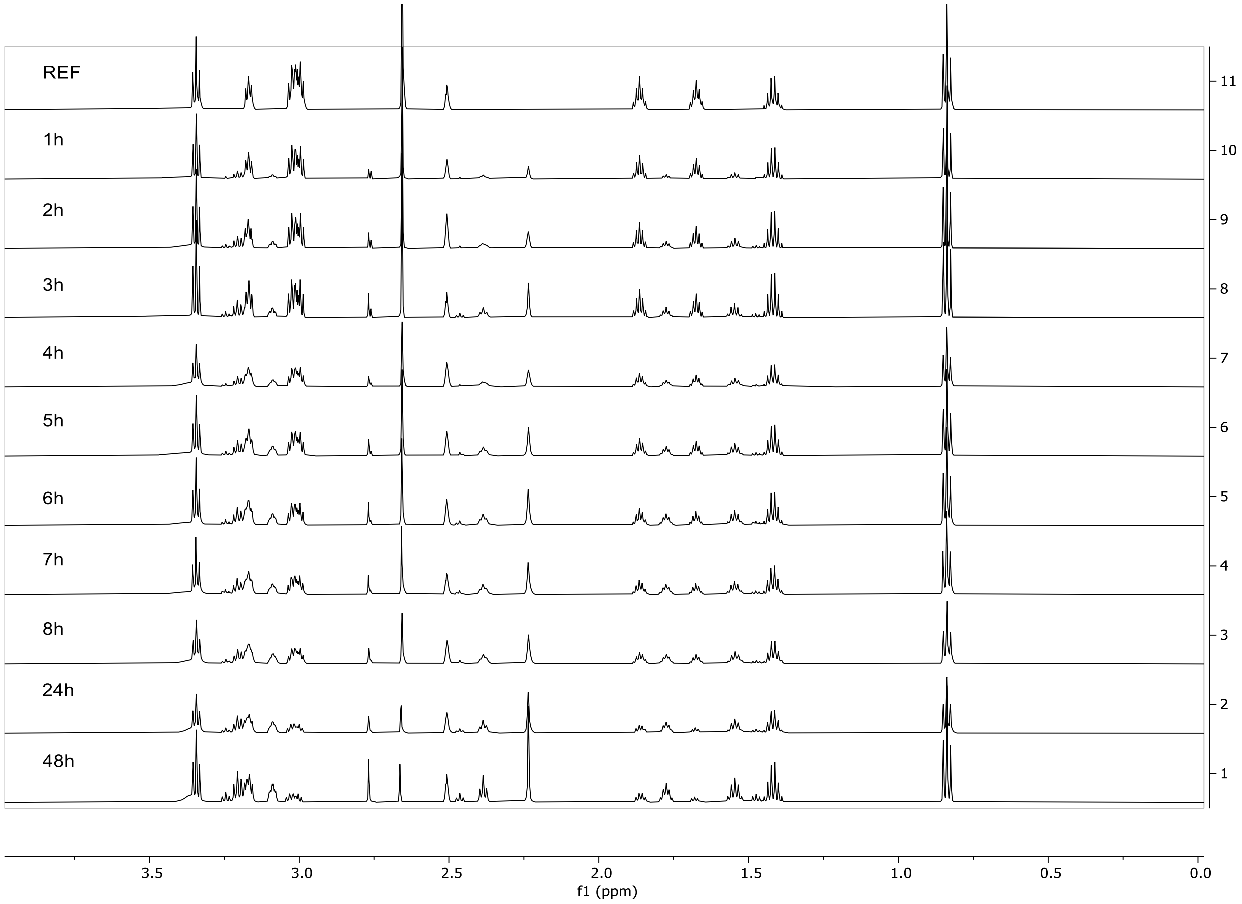

Figure S26 ^{1}H NMR 1 molar equivalent of mTBD, 1 molar equivalent of water and 1 molar equivalent of propanol.

Structure	Time	integral N-Methyl	integral HP1	integral HP2	% HP
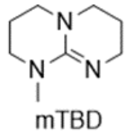	REF	56121500.00	655175.00	957258.00	0.00
	1h	33384200.00	7904250.00	3572780.00	25.58
	2h	32237100.00	11805600.00	4369280.00	33.41
	3h	34728400.00	16739200.00	5533000.00	39.07
	4h	26083500.00	15180900.00	4669490.00	43.22
	5h	27768100.00	18869500.00	5505380.00	46.75
	6h	28416500.00	24874000.00	7001100.00	52.87
	7h	19481100.00	18217900.00	5130560.00	54.51
	8h	23219900.00	23718000.00	6531270.00	56.57
	24h	13991600.00	28370400.00	7420820.00	71.89
	48h	9913060.00	35329400.00	9125150.00	81.77

Table S27 1 molar equivalent of mTBD, 1 molar equivalent of water and 2 molar equivalents of propanol.

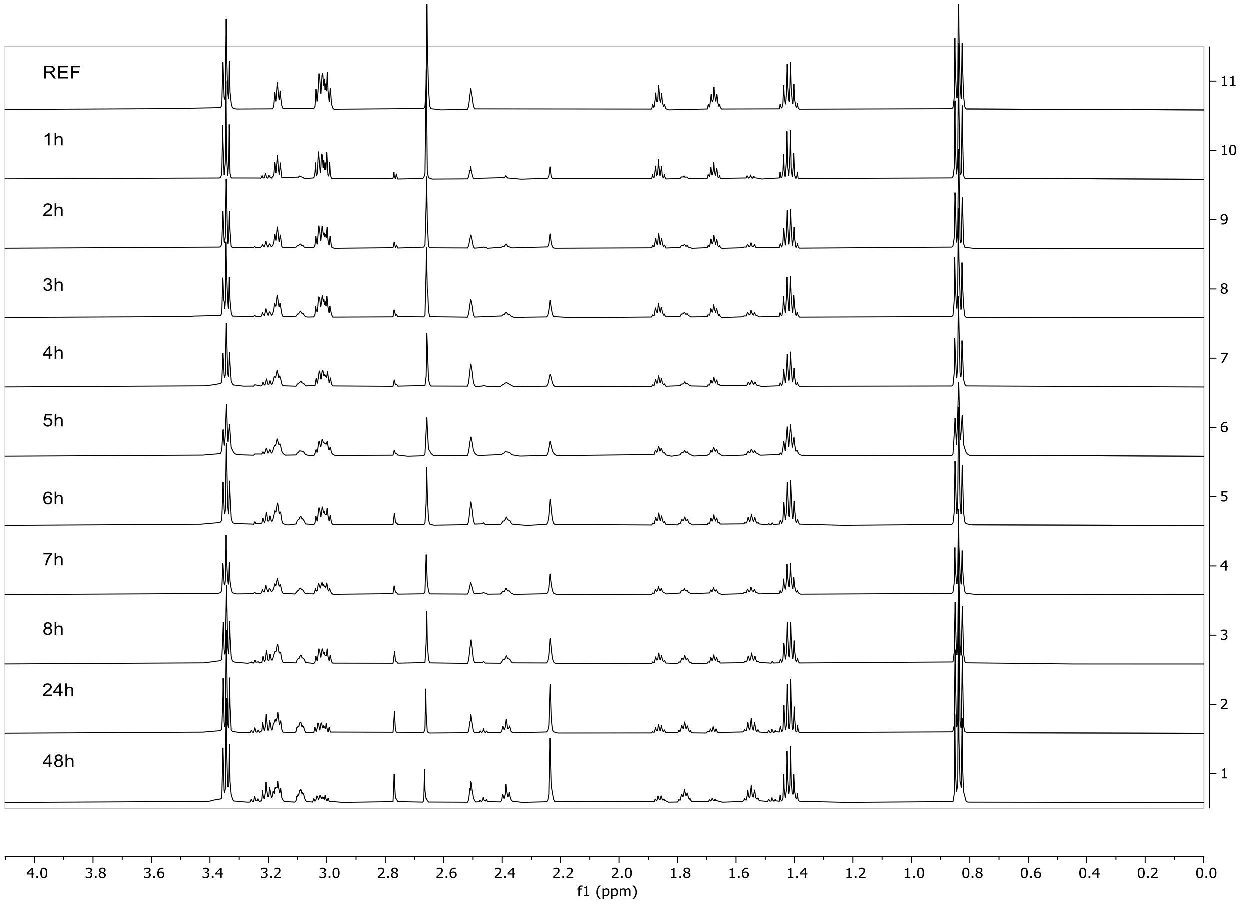

Figure S27 ^{1}H NMR 1 molar equivalent of mTBD, 1 molar equivalent of water and 2 molar equivalents of propanol.

2. <u>[mTBDH][OAc]</u>

a. [mTBDH][OAc] + water

Structure	Time	Integral N-Methyl	Integral HP	% HP
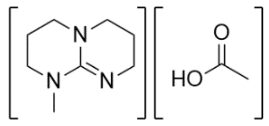	REF	267107000.00	0	0
	1h	300036000.00	538829.00	0.18
	2h	292056000.00	944885.00	0.32
	3h	224731000.00	1204880.00	0.53
	4h	163100000.00	1884370.00	1.14
	5h	329514000.00	5089200.00	1.52
	6h	324379000.00	6050250.00	1.83
	24h	262844000.00	17984900.00	6.40
	48h	249919000.00	32026600.00	11.36

Table S28 1 molar equivalent of [mTBDH][OAc] and 0.2 molar equivalent of water.

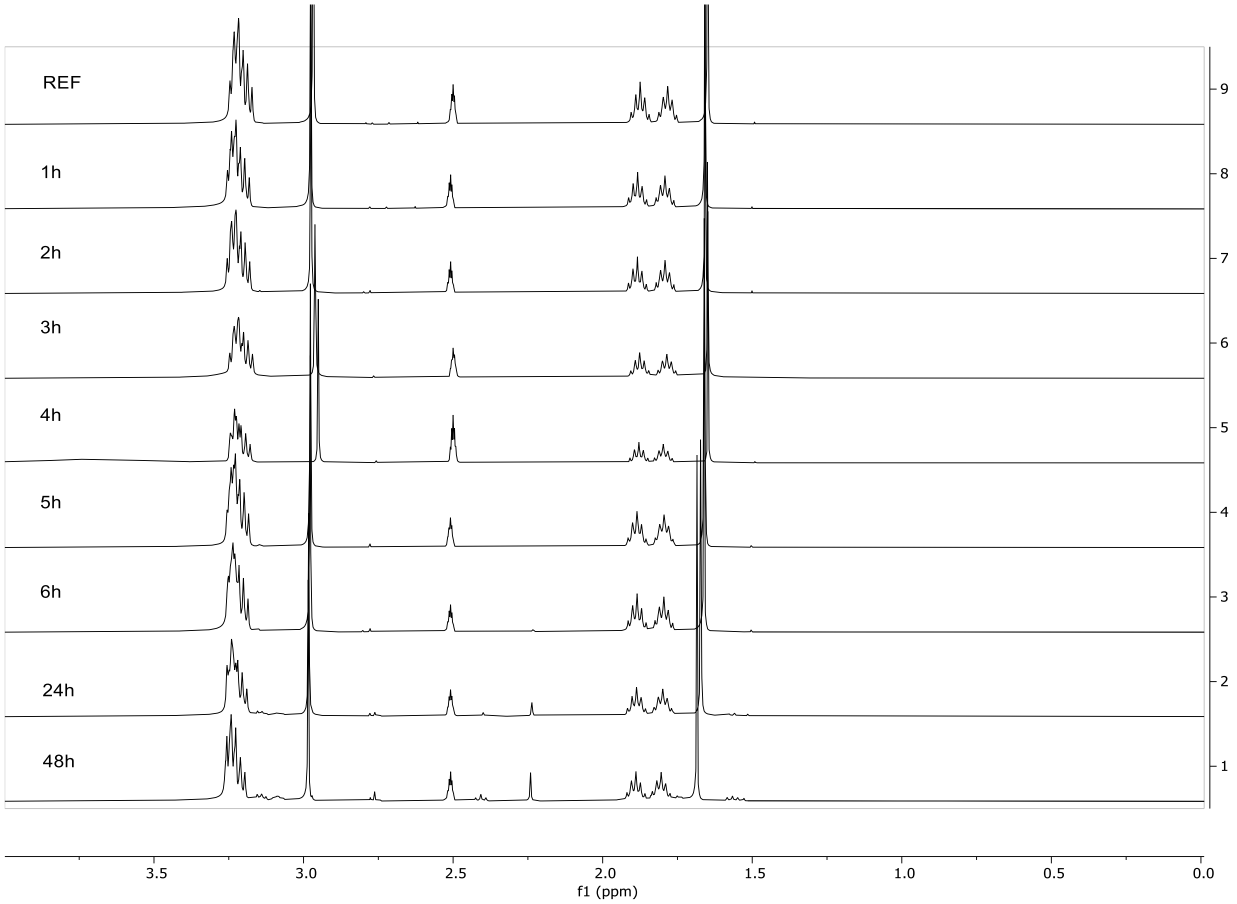

Figure S28 ^{1}H NMR 1 molar equivalent of [mTBDH][OAc] and 0.2 molar equivalent of water.

Structure	Time	Integral N-Methyl	Integral HP	% HP
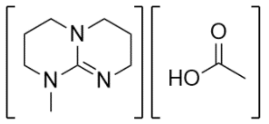	REF	241928000.00	0	0
	1h	214254000.00	2250800.00	1.04
	2h	220199000.00	3792760.00	1.69
	3h	239860000.00	6787920.00	2.75
	4h	209905000.00	6788960.00	3.13
	5h	227671000.00	7792920.00	3.31
	6h	206568000.00	9347610.00	4.33
	24h	216168000.00	29663800.00	12.07
	48h	162434000.00	42091600.00	20.58

Table S29 1 molar equivalent of [mTBDH][OAc] and 0.5 molar equivalent of water.

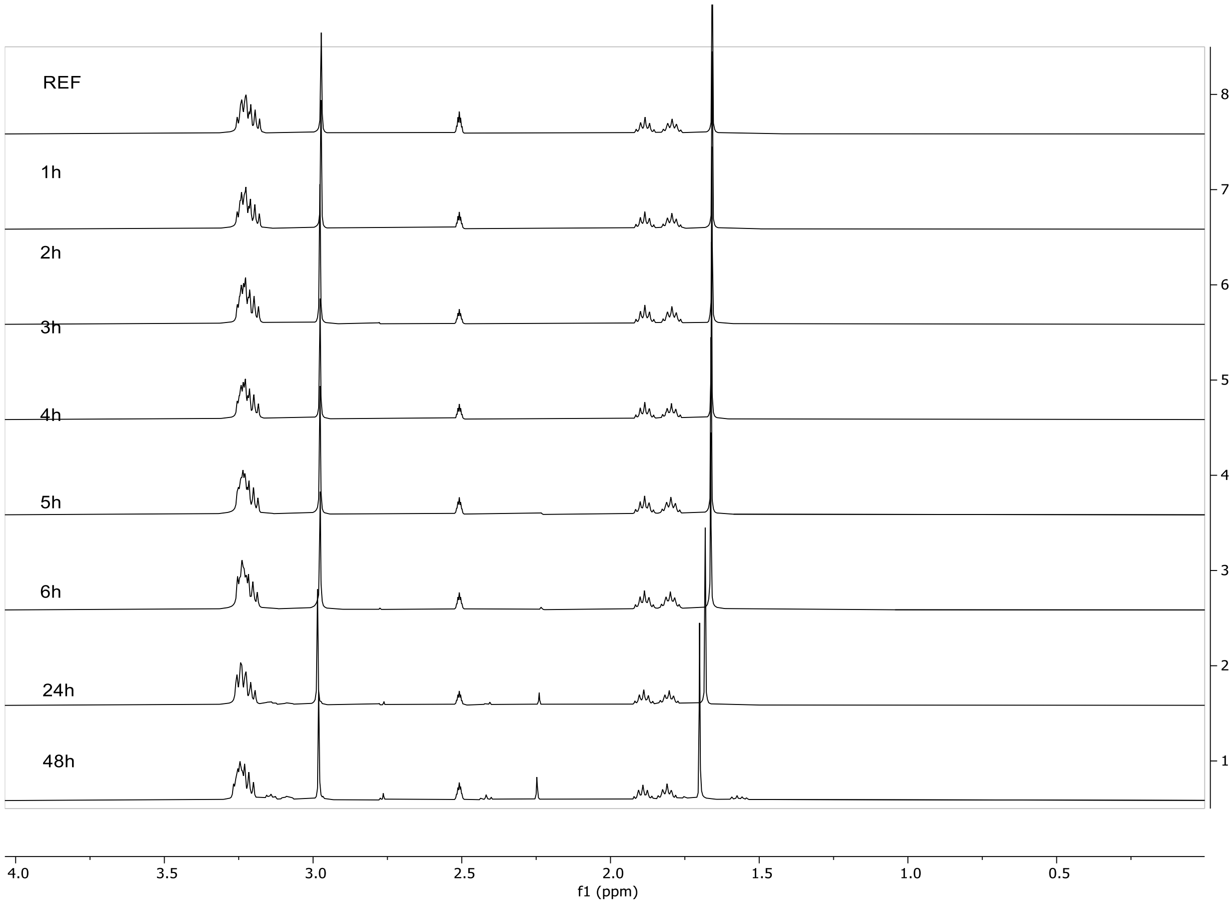

Figure S29 ^{1}H NMR 1 molar equivalent of [mTBDH][OAc] and 0.5 molar equivalent of water.

Structure	Time	Integral N-Methyl	Integral HP1	integral HP2	% HP
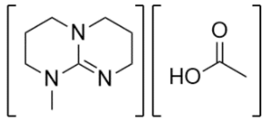	REF	224537000.00	0	0	0
	1h	224537000.00	512081.00	260546.00	0.34
	2h	219879000.00	2027920.00	217956.00	1.01
	3h	255048000.00	4052140.00	258076.00	1.66
	4h	260185000.00	6068220.00	353416.00	2.40
	5h	226600000.00	6094730.00	415538.00	2.79
	6h	223087000.00	7403650.00	496243.00	3.42
	24h	183778000.00	23603700.00	3460300.00	12.83
	48h	171300000.00	37440400.00	6313070.00	20.34

Table S30 1 molar equivalent of mTBDH][OAc] and 1 molar equivalent of water.

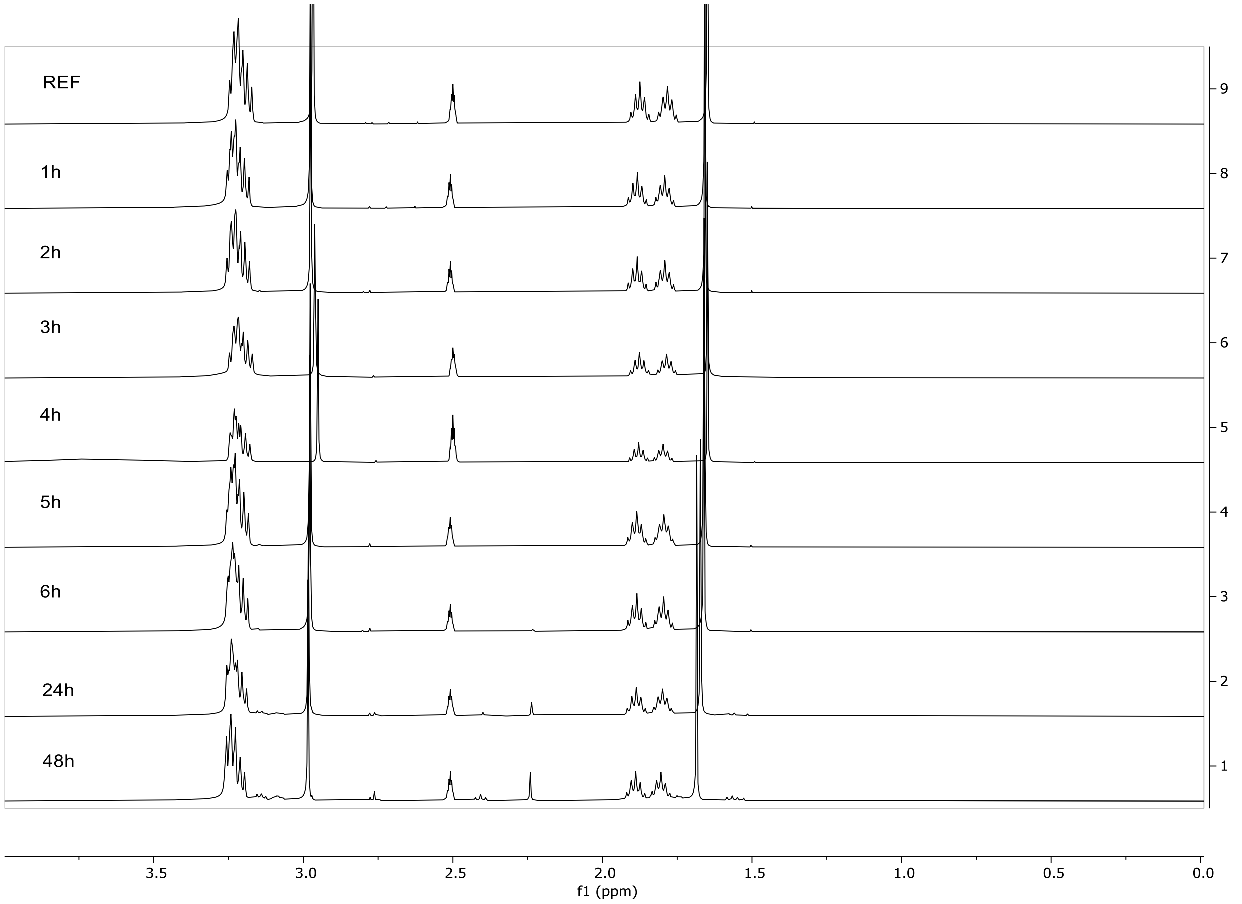

Figure S30 ^{1}H NMR 1 molar equivalent of mTBDH][OAc] and 1 molar equivalent of water.

Structure	Time	Integral N-Methyl	Integral HP1	integral HP2	% HP
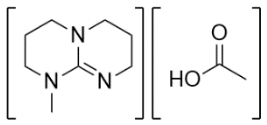	REF	197403000.00	464191.00	0.00	0.00
	1h	160249000.00	633598.00	0.00	0.39
	2h	168145000.00	1346670.00	336467.00	0.99
	3h	174234000.00	2022530.00	279829.00	1.30
	4h	145676000.00	2008340.00	237912.00	1.52
	5h	187417000.00	2747620.00	396449.00	1.65
	7h	193657000.00	4038730.00	441784.00	2.26
	24h	161754000.00	9941460.00	1364280.00	6.53
	48h	162492000.00	17148400.00	2450590.00	10.76

Table S31 1 molar equivalent of [mTBDH][OAc] and 5 molar equivalents of water.

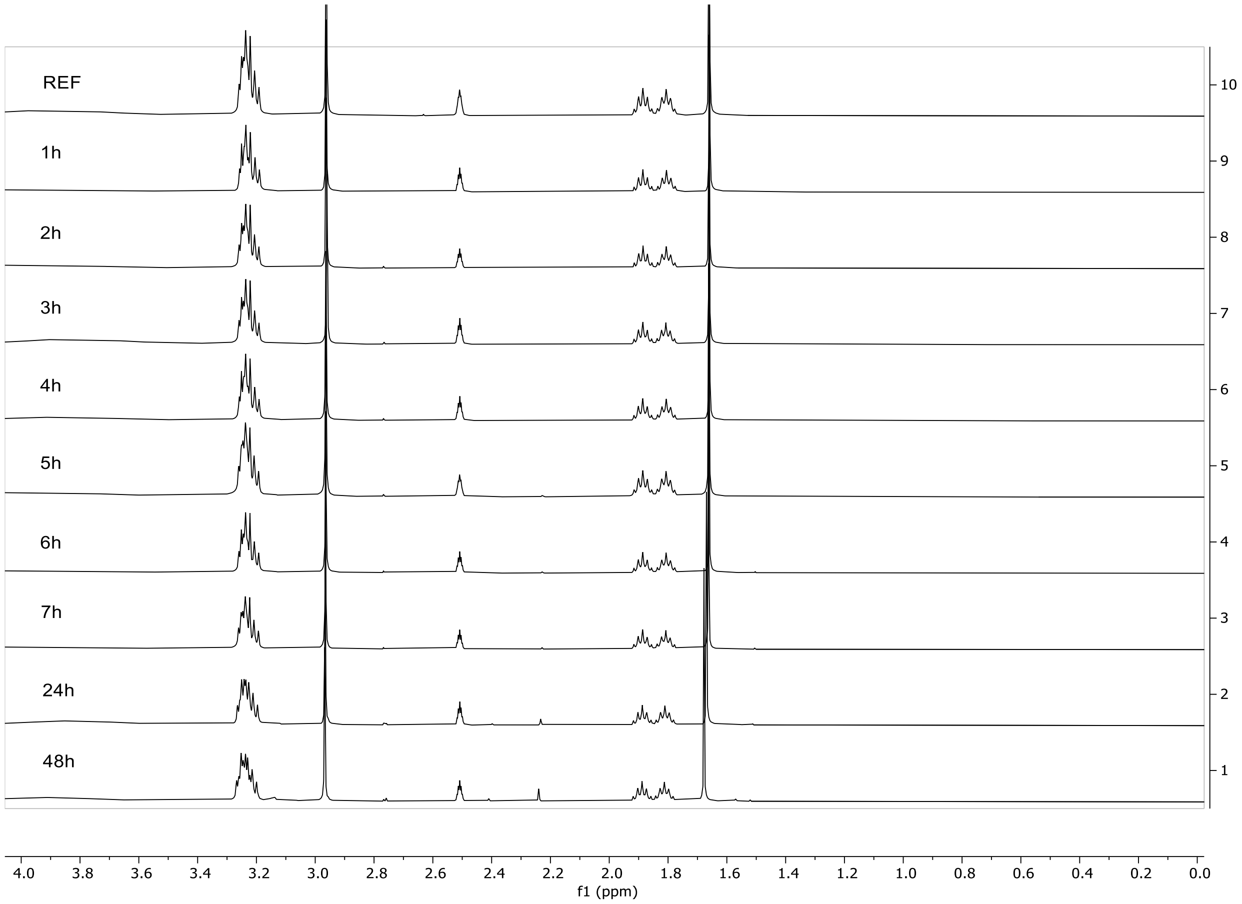

Figure S31 ^{1}H NMR 1 molar equivalent of [mTBDH][OAc] and 5 molar equivalents of water.

Structure	Time	Integral N-Methyl	Integral HP	% HP
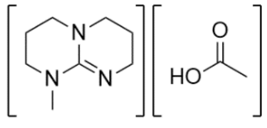	REF	113686000.00	279413.00	0
	1h	128149000.00	376667.00	0.29
	2h	135585000.00	552584.00	0.41
	3h	160454000.00	784448.00	0.49
	4h	137643000.00	928327.00	0.67
	5h	146880000.00	994372.00	0.67
	6h	150129000.00	1203190.00	0.80
	7h	163902000.00	1939600.00	1.17
	24h	137901000.00	6795400.00	4.70
	48h	152243000.00	7097530.00	4.45

Table S32 1 molar equivalent of [mTBDH][OAc] and 10 molar equivalents of water.

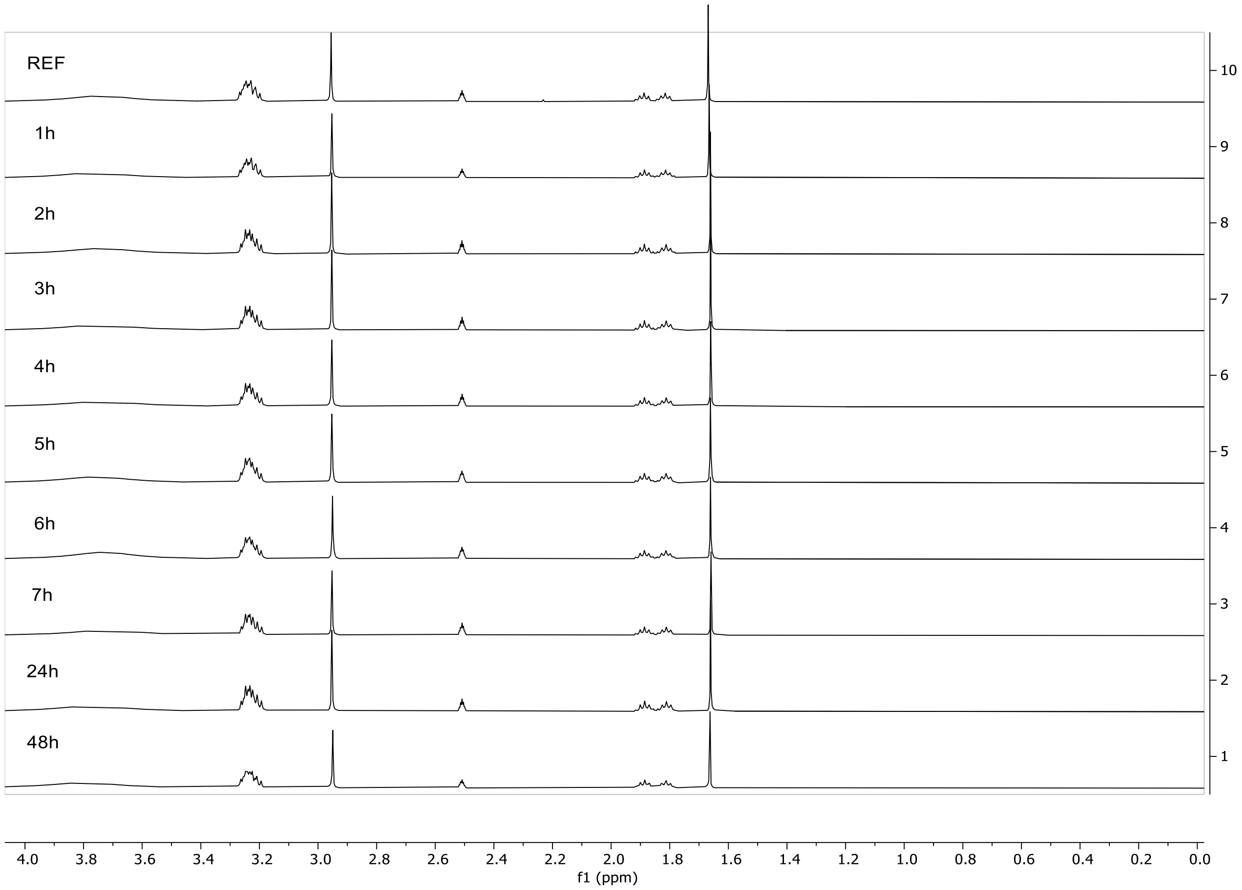

Figure S32 ^{1}H NMR molar equivalent of [mTBDH][OAc] and 10 molar equivalents of water.

b. [mTBDH][OAc] + water + DMSO

Structure	Time	Integral N-Methyl	Integral HP1	Integral HP2	% HP
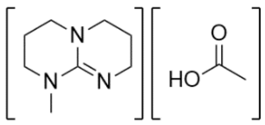	REF	78371600.00	243088.00	703550.00	0
	1h	83511900.00	98416.60	265601.00	0.43
	2h	88478200.00	444580.00	260308.00	0.79
	3h	63505200.00	556307.00	34901.20	0.92
	4h	94270700.00	832201.00	332312.00	1.22
	5h	73173300.00	1193900.00	143321.00	1.79
	6h	73144100.00	2261940.00	573641.00	3.73
	7h	66777400.00	2547550.00	716724.00	4.66
	8h	81239800.00	3153940.00	1076300.00	4.94
	24h	56388900.00	6293810.00	1448840.00	12.07
	48h	67374100.00	12472100.00	2964220.00	18.64

Table S33 1 molar equivalent of [mTBDH][OAc], 1 molar equivalent of water and 0.2 equivalent of DMSO.

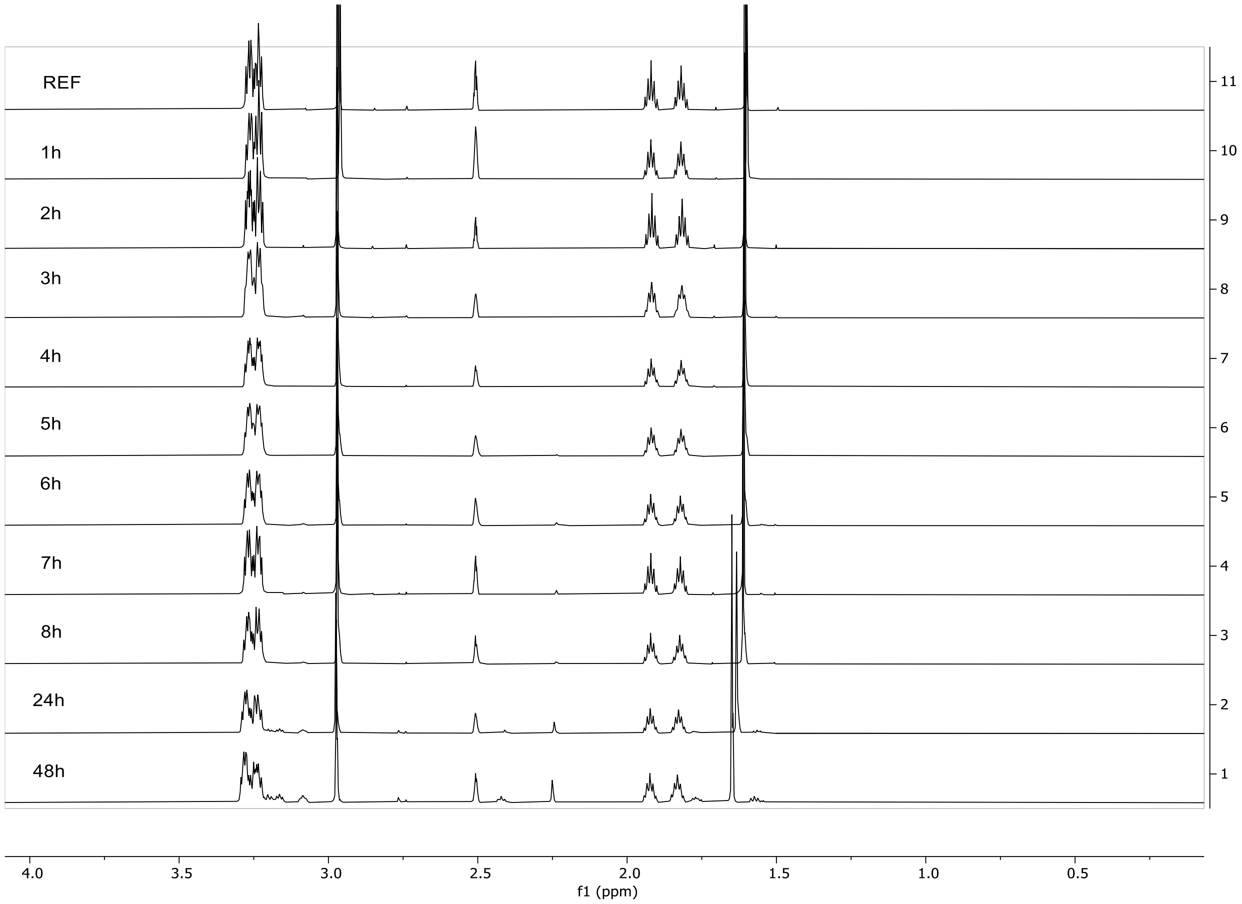

Figure S33 ^{1}H NMR 1 molar equivalent of [mTBDH][OAc], 1 molar equivalent of water and 0.2 equivalent of DMSO.

Structure	Time	integral N-Methyl	integral HP1	integral HP2	% HP
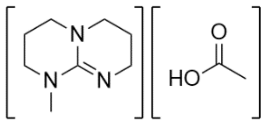	REF	80329200.00	9353.72	0.00	0.00
	1h	79329100.00	46900.60	87319.30	0.17
	2h	74831900.00	939780.00	118091.00	1.39
	3h	107013000.00	1403360.00	1164000.00	2.34
	4h	78254700.00	1700260.00	313737.00	2.51
	5h	75940500.00	1067510.00	965735.00	2.61
	6h	83477000.00	2105260.00	558440.00	3.09
	7h	74752700.00	2254390.00	479391.00	3.53
	8h	72930800.00	2266790.00	935072.00	4.21
	24h	64866100.00	6133640.00	1493080.00	10.52
	48h	49839500.00	7984200.00	1850410.00	16.48

Table S34 1 molar equivalent of [mTBDH][OAc], 1 molar equivalent of water and 0.5 equivalent of DMSO.

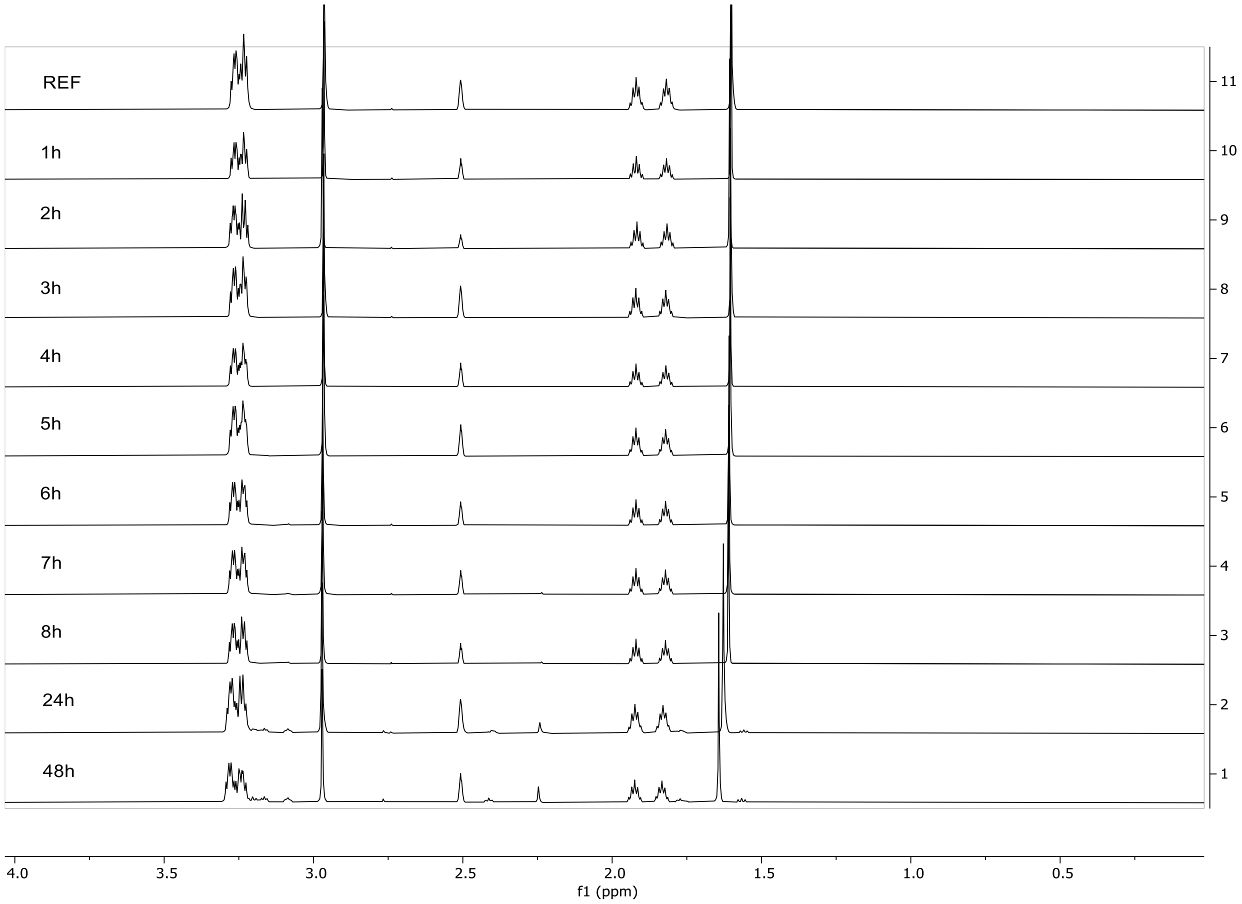

Figure S34 ^{1}H NMR 1 molar equivalent of [mTBDH][OAc], 1 molar equivalent of water and 0.5 equivalent of DMSO.

Structure	Time	Integral N-Methyl	Integral HP1	Integral HP2	% HP
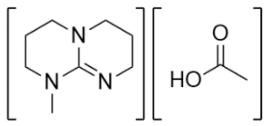	REF	58154100.00	0.00	0.00	0.00
	1h	70220100.00	42502.40	109167.00	0.22
	2h	74917700.00	156862.00	181328.00	0.45
	3h	69300700.00	598713.00	398560.00	1.42
	4h	85425800.00	764573.00	725572.00	1.71
	5h	67493900.00	939376.00	578338.00	2.20
	6h	72697600.00	1311680.00	493552.00	2.42
	7h	67118100.00	1503420.00	652837.00	3.11
	8h	76323500.00	1788930.00	780287.00	3.26
	24h	64426300.00	5217720.00	1267270.00	9.15
	48h	57848300.00	7977170.00	1803450.00	14.46

Table S35 1 molar equivalent of [mTBDH][OAc], 1 molar equivalent of water and 1 equivalent of DMSO.

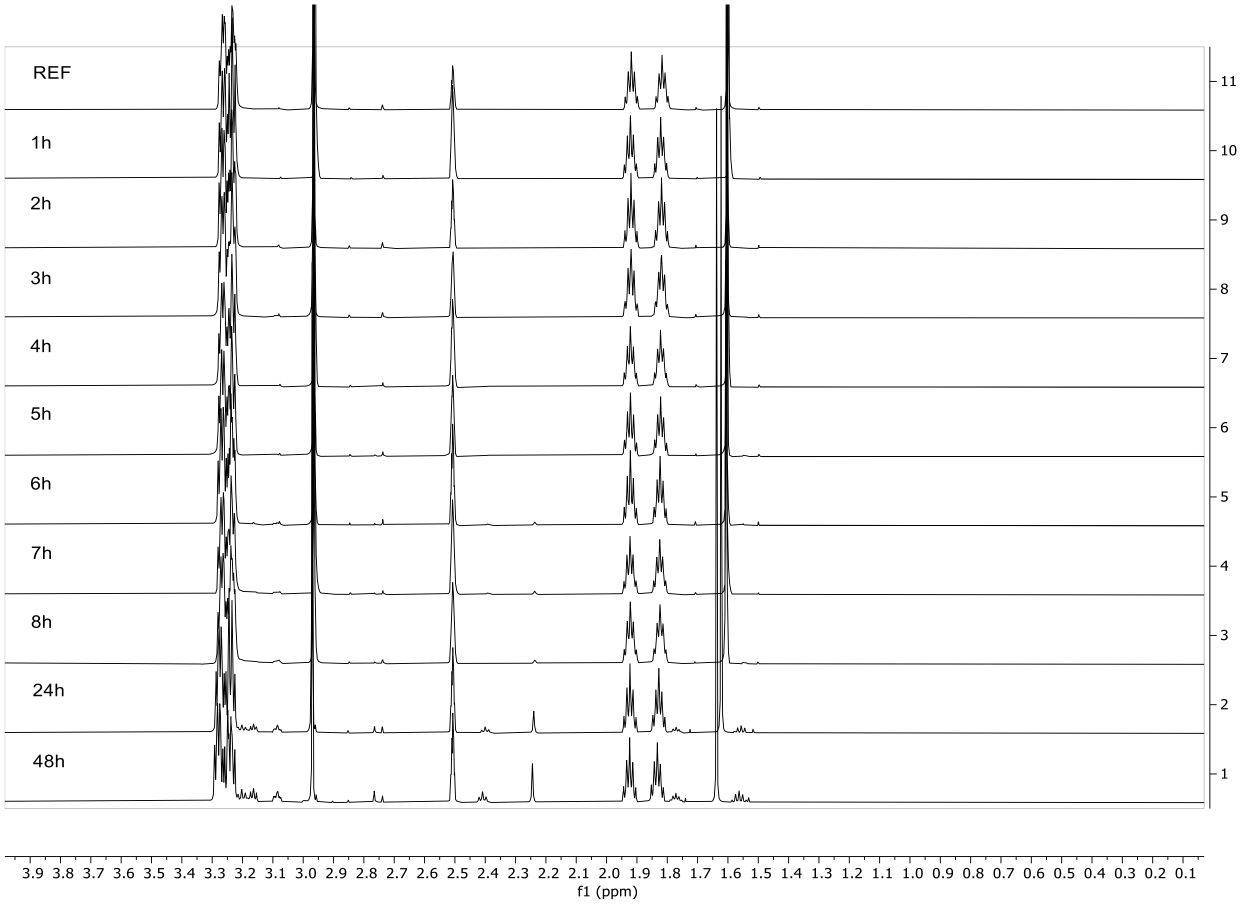

Figure S35 ^{1}H NMR 1 molar equivalent of [mTBDH][OAc], 1 molar equivalent of water and 1 equivalent of DMSO.

Structure	Time	integral N-Methyl	integral HP1	integral HP2	% HP
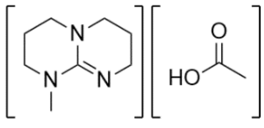	REF	64361600.00	82381.20	395302.00	0.00
	1h	72074400.00	445992.00	89685.50	0.74
	2h	48786200.00	144237.00	258007.00	0.82
	3h	74984900.00	348075.00	408524.00	1.00
	4h	74950100.00	710143.00	376640.00	1.43
	5h	59758400.00	840972.00	545333.00	2.27
	6h	61935600.00	1138070.00	595007.00	2.72
	7h	67214700.00	1494540.00	718712.00	3.19
	8h	75414300.00	1801040.00	851461.00	3.40
	24h	63763200.00	4733060.00	1336410.00	8.69
	48h	50749200.00	6393490.00	1664020.00	13.70

Table S36 1 molar equivalent of [mTBDH][OAc], 1 molar equivalent of water and 1 equivalent of DMSO.

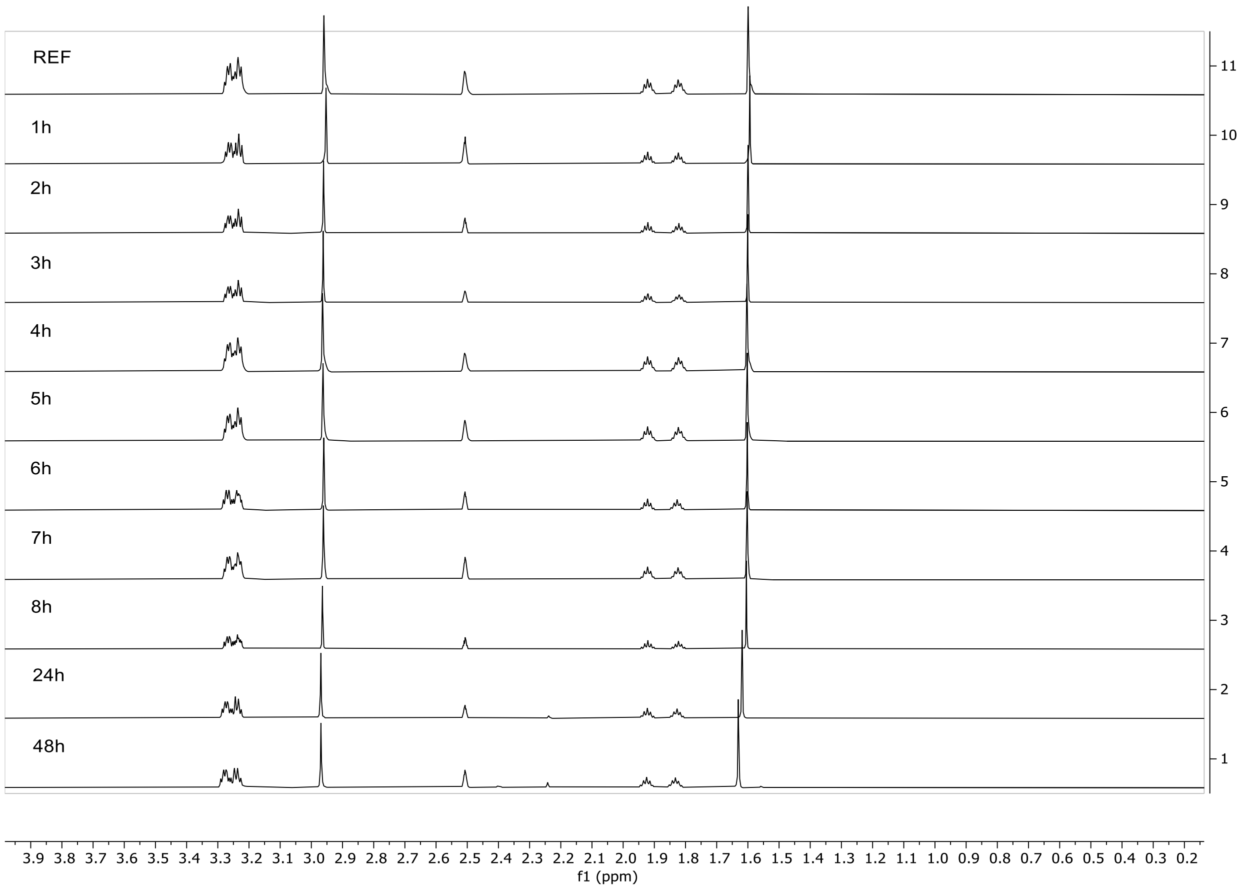

Figure S36 ^{1}H NMR 1 molar equivalent of [mTBDH][OAc], 1 molar equivalent of water and 1 equivalent of DMSO.

c. [mTBDH][OAc] + water + ethylene glycol

Structure	Time	Integral N-Methyl	Integral HP1	Integral HP2	% HP
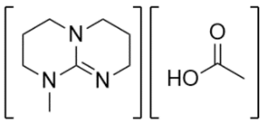	REF	111760000.00	548617.00	511257.00	0.00
	1h	115907000.00	826142.00	608174.00	1.22
	2h	93735400.00	1146900.00	538388.00	1.77
	3h	91353500.00	1275990.00	590450.00	2.00
	4h	116948000.00	2102800.00	651254.00	2.30
	5h	88299000.00	1831990.00	520049.00	2.59
	6h	108851000.00	2606220.00	659531.00	2.91
	7h	159277000.00	3934360.00	962008.00	2.98
	8h	124557000.00	3597230.00	1060010.00	3.60
	24h	91952400.00	9186860.00	1797670.00	10.67
	48	87087900.00	11648200.00	2474910.00	13.95

Table S37 1 molar equivalent of [mTBDH][OAc], 1 molar equivalent of water and 0.2 equivalent of ethylene glycol.

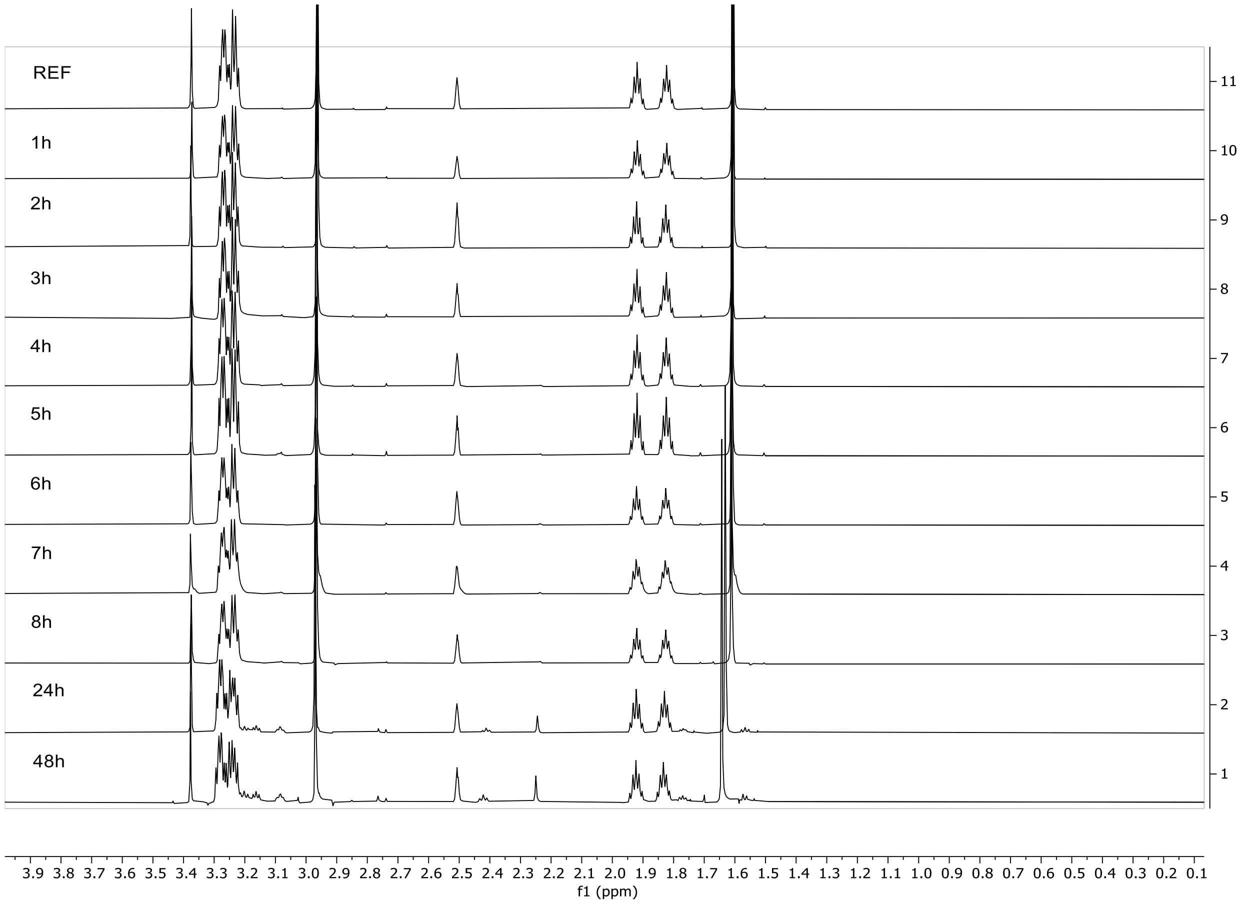

Figure S37 ^{1}H NMR 1 molar equivalent of [mTBDH][OAc], 1 molar equivalent of water and 0.2 equivalent of ethylene glycol.

Structure	Time	Integral N-Methyl	Integral HP1	% HP
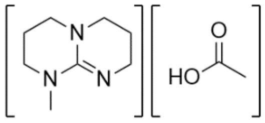	REF	70075900.00	0.00	0.00
	1h	62777500.00	0.00	0.00
	2h	58262300.00	34906.30	0.05
	3h	58220100.00	87476.00	0.15
	4h	68552800.00	335163.00	0.48
	5h	51284400.00	622976.00	1.20
	6h	75724100.00	937438.00	1.22
	7h	60709400.00	755779.00	1.22
	8h	57140100.00	715548.00	1.23
	24h	69178500.00	4388280.00	5.96
	48h	49425400.00	5054170.00	9.27

Table S38 1 molar equivalent of [mTBDH][OAc], 1 molar equivalent of water and 0.5 equivalent of ethylene glycol.

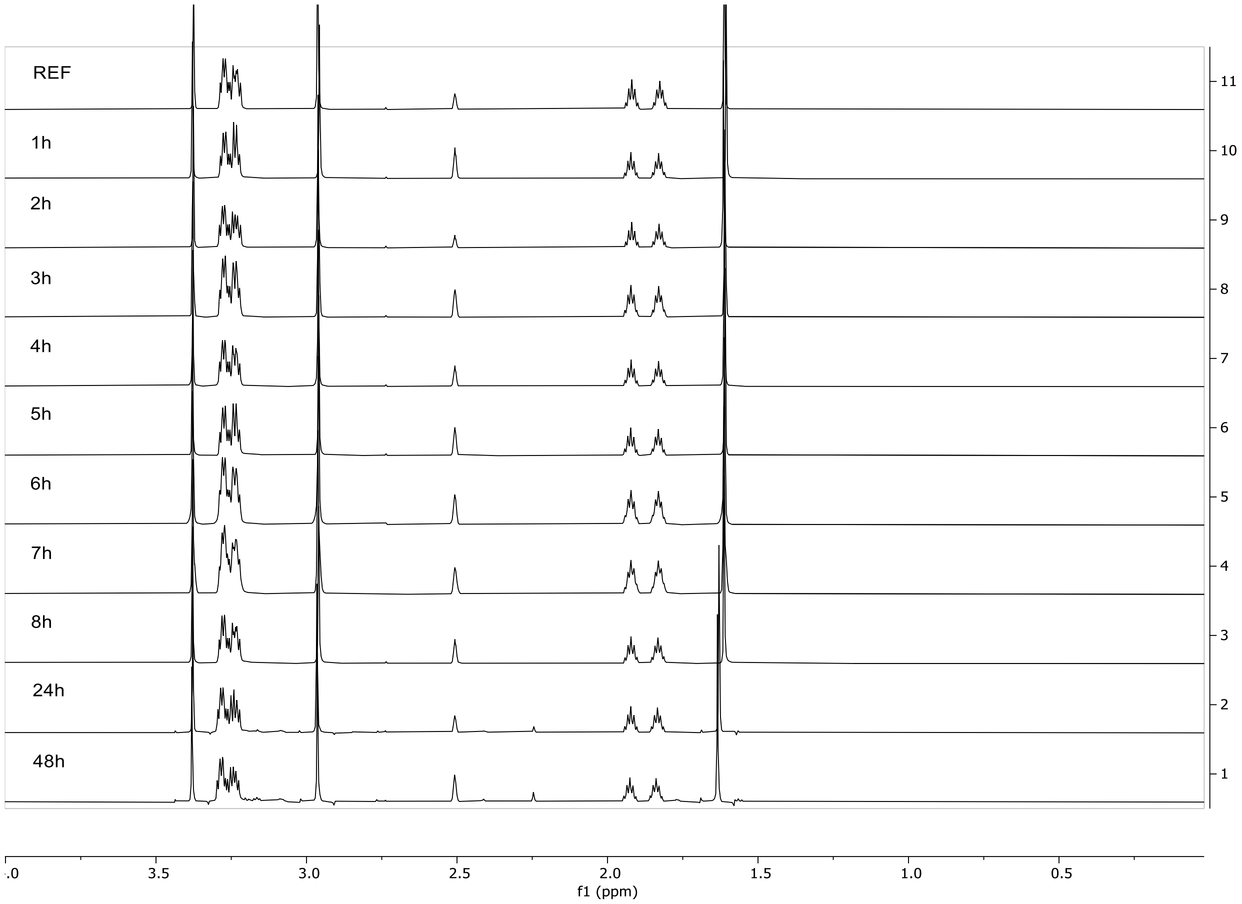

Figure S38 ^{1}H NMR 1 molar equivalent of [mTBDH][OAc], 1 molar equivalent of water and 0.5 equivalent of ethylene glycol.

Structure	Time	Integral N-Methyl	Integral HP	Integral HP2	% HP
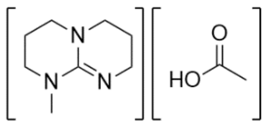	REF	58097600.00	0.00	0.00	0.00
	1h	61083800.00	0.00	0.00	0.00
	2h	56344700.00	0.00	0.00	0.00
	3h	57140400.00	0.00	0.00	0.00
	4h	51349400.00	0.00	0.00	0.00
	5h	61728400.00	0.00	0.00	0.00
	6h	63476800.00	4355.20	82240.80	0.14
	7h	53287900.00	247305.00	150366.00	0.74
	8h	57173700.00	268434.00	183873.00	0.78
	24h	43917900.00	2011660.00	524529.00	5.46
	48h	39934100.00	2966660.00	603222.00	8.21

Table S39 1 molar equivalent of [mTBDH][OAc], 1 molar equivalent of water and 1 equivalent of ethylene glycol.

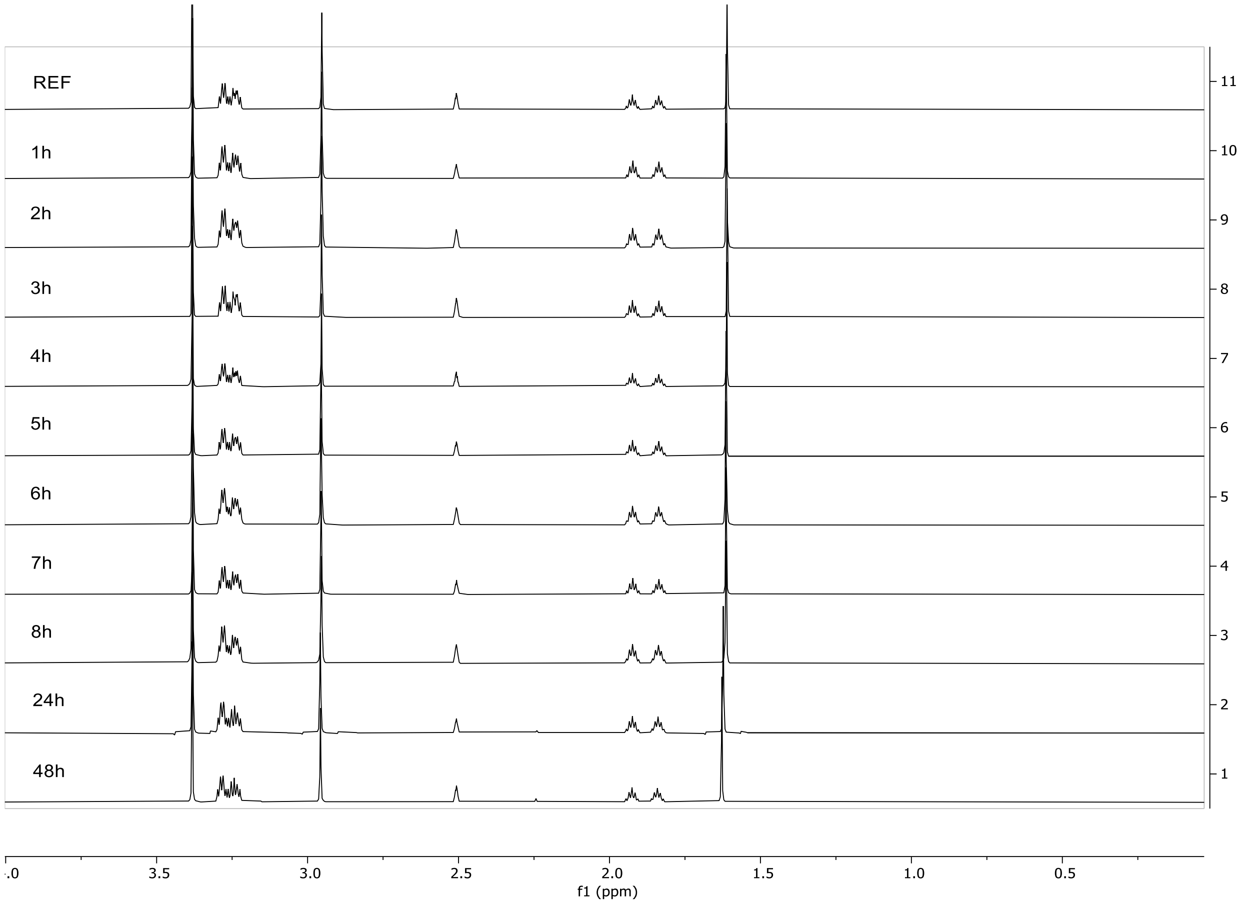

Figure S39 ^{1}H NMR 1 molar equivalent of [mTBDH][OAc], 1 molar equivalent of water and 1 equivalent of ethylene glycol.

Structure	Time	Integral N-Methyl	Integral HP	% HP
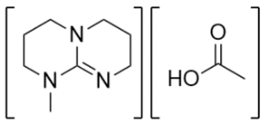	REF	40728200.00	0	0.00
	1h	39432200.00	0.00	0.00
	2h	52533400.00	0.00	0.00
	3h	44815700.00	0.00	0.00
	4h	54913200.00	0.00	0.00
	5h	40823300.00	0.00	0.00
	6h	45810700.00	0.00	0.00
	7h	45927100.00	447332.00	0.96
	8h	54772000.00	1110760.00	1.98
	24h	49803000.00	1012790.00	1.99
	48h	38693800.00	1058890.00	2.66

Table S40 1 molar equivalent of [mTBDH][OAc], 1 molar equivalent of water and 2 equivalent of ethylene glycol.

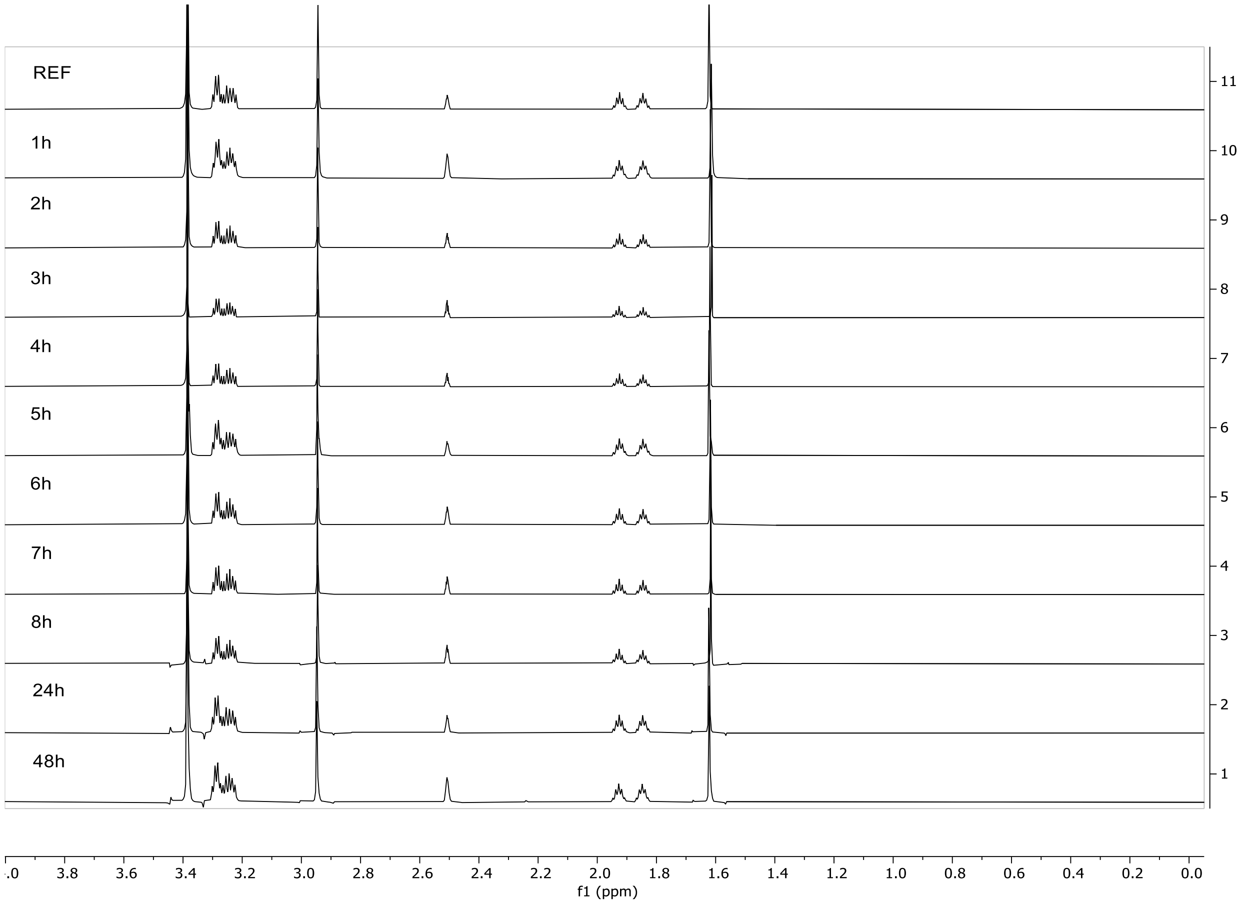

Figure S40 ^{1}H NMR 1 molar equivalent of [mTBDH][OAc], 1 molar equivalent of water and 2 equivalent of ethylene glycol.

d. [mTBDH][OAc] + water + phenol

Structure	Time	integral N-Methyl	integral HP1	integral HP2	% HP
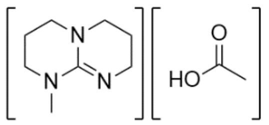	REF	165294000.00	76785.50	0.00	0.00
	1h	147810000.00	79809.10	392839.00	0.32
	2h	161510000.00	292915.00	1544320.00	1.12
	3h	119936000.00	408654.00	1865310.00	1.86
	4h	168495000.00	580824.00	3299660.00	2.25
	5h	140427000.00	605066.00	3511120.00	2.85
	6h	152032000.00	460523.00	4319370.00	3.05
	7h	125511000.00	654547.00	4492950.00	3.94
	8h	118258000.00	523084.00	4510660.00	4.08
	24h	116058000.00	2366320.00	14320700.00	12.57
	48h	122396000.00	4054290.00	23128600.00	18.17

Table S41 1 molar equivalent of [mTBDH][OAc], 1 molar equivalent of water and 0.2 equivalent of phenol.

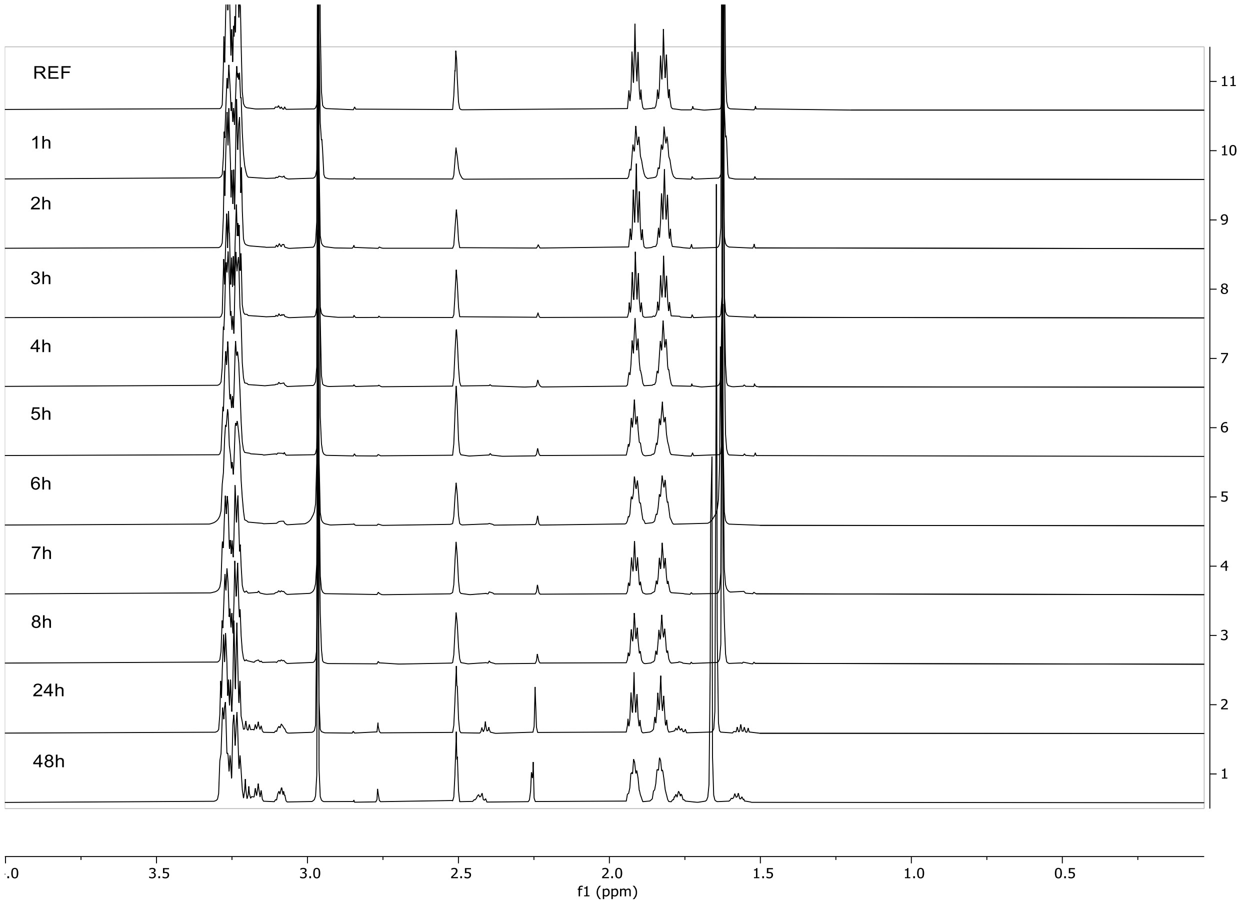

Figure S41 ^{1}H NMR 1 molar equivalent of [mTBDH][OAc], 1 molar equivalent of water and 0.2 equivalent of phenol.

Structure	Time	Integral N-Methyl	Integral HP1	integral HP2	% HP
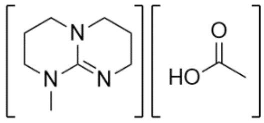	REF	167377000.00	0.00	9634.23	0.00
	1h	118982000.00	532701.00	112618.00	0.54
	2h	118458000.00	1000700.00	300268.00	1.09
	3h	157765000.00	2090440.00	628590.00	1.69
	4h	148808000.00	2619370.00	734559.00	2.20
	5h	155827000.00	3350090.00	893023.00	2.65
	6h	143590000.00	3765590.00	899659.00	3.15
	7h	109142000.00	3114390.00	732393.00	3.40
	8h	149036000.00	4997910.00	1173020.00	3.98
	24h	135401000.00	12265300.00	2754340.00	9.99
	48h	136813000.00	18332000.00	4443030.00	14.27

Table S42 1 molar equivalent of [mTBDH][OAc], 1 molar equivalent of water and 0.5 equivalent of phenol.

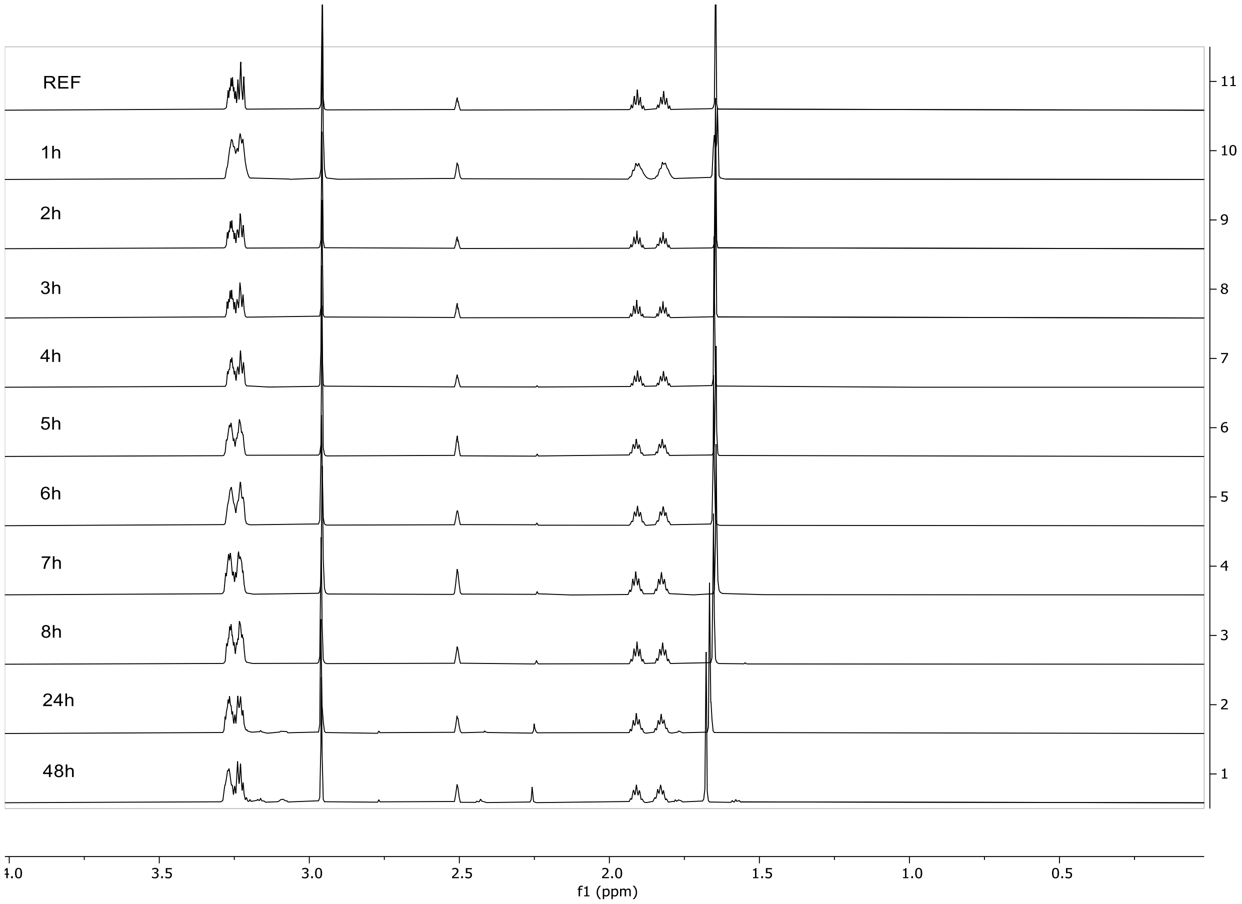

Figure S42 ^{1}H NMR 1 molar equivalent of [mTBDH][OAc], 1 molar equivalent of water and 0.5 equivalent of phenol.

Structure	Time	Integral N-Methyl	Integral HP1	Integral HP2	% HP
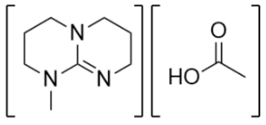	REF	110274000.00	0.00	0.00	0.00
	1h	115888000.00	55360.00	0.00	0.05
	2h	117582000.00	241886.00	0.00	0.21
	3h	115735000.00	411067.00	0.00	0.35
	4h	114949000.00	599663.00	0.00	0.54
	5h	125686000.00	707271.00	0.00	0.56
	6h	96610300.00	813468.00	43969.10	0.88
	7h	91623400.00	853002.00	86507.70	1.01
	8h	105091000.00	1092300.00	180675.00	1.20
	24h	125779000.00	5733440.00	1000280.00	5.08
	48h	127394000.00	8290200.00	1374600.00	7.05

Table S43 1 molar equivalent of [mTBDH][OAc], 1 molar equivalent of water and 1 equivalent of phenol.

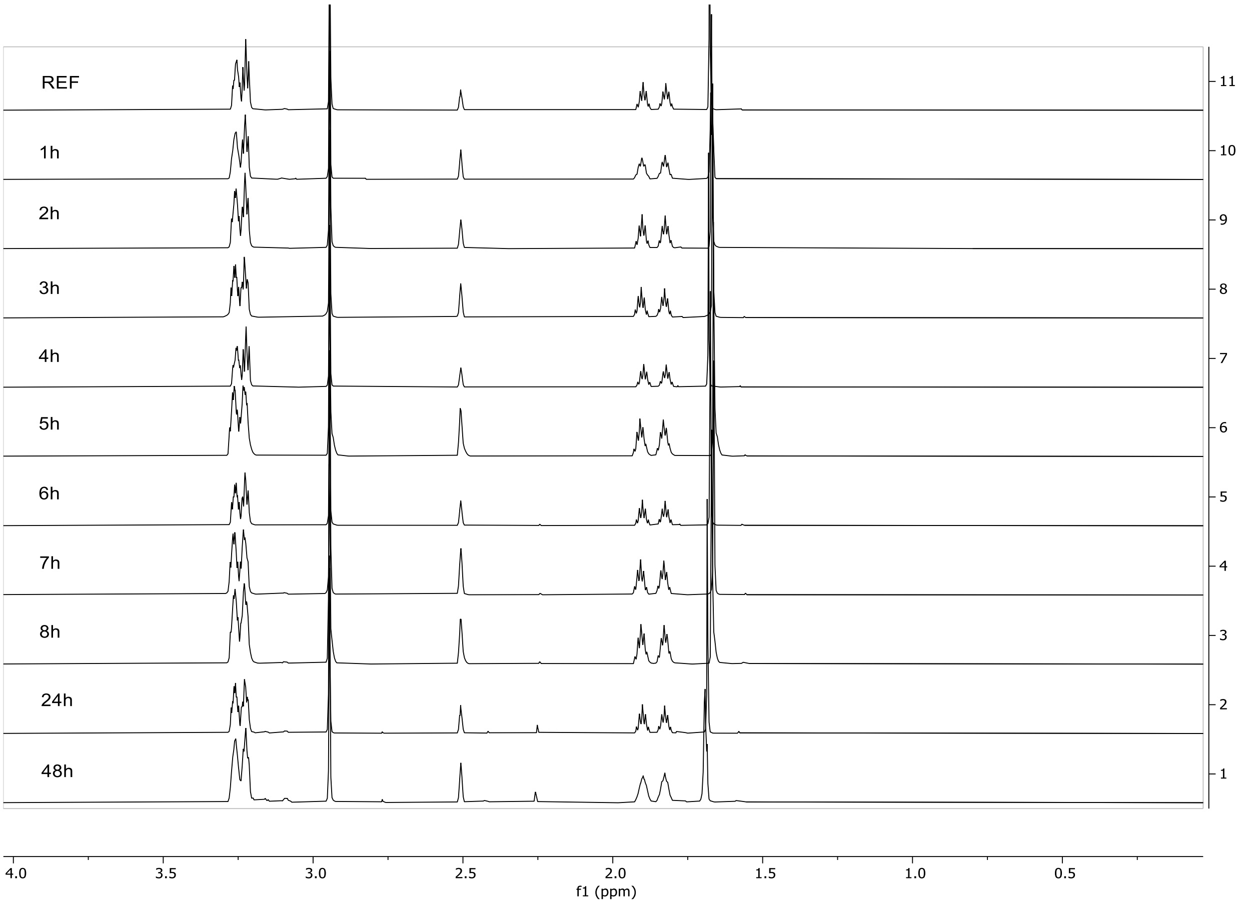

Figure S43 ^{1}H NMR 1 molar equivalent of [mTBDH][OAc], 1 molar equivalent of water and 1 equivalent of phenol.

Structure	Time	Integral N-Methyl	Integral HP1	Integral HP2	% HP
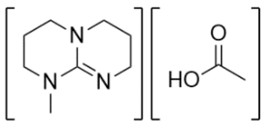	REF	94477100.00	0.00	0.00	0.00
	1h	94304500.00	0.00	0.00	0.00
	2h	96218400.00	0.00	0.00	0.00
	3h	89197500.00	0.00	0.00	0.00
	4h	107398000.00	0.00	0.00	0.00
	5h	96372800.00	0.00	0.00	0.00
	6h	93533200.00	69782.40	0.00	0.07
	7h	94355800.00	70723.50	0.00	0.07
	8h	105450000.00	138904.00	0.00	0.13
	24h	98567200.00	367650.00	0.00	0.37
	48h	123046000.00	785203.00	0.00	0.63

Table S44 1 molar equivalent of [mTBDH][OAc], 1 molar equivalent of water and 2 equivalent of phenol.

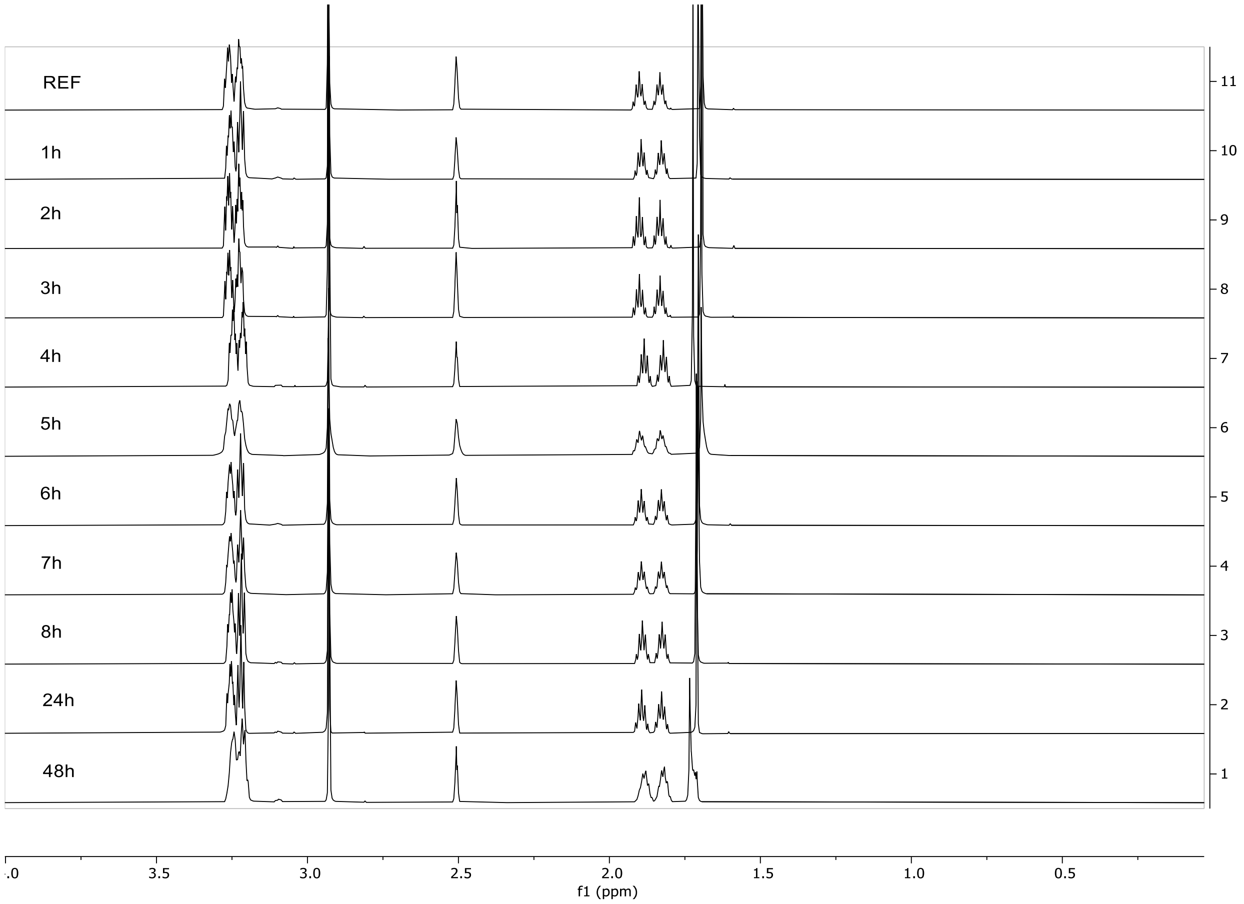

Figure S44 ^{1}H NMR 1 molar equivalent of [mTBDH][OAc], 1 molar equivalent of water and 2 equivalent of phenol.

e. [mTBDH][OAc] + water + propanol

Structure	Time	Integral N-Methyl	Integral HP1	Integral HP2	% HP
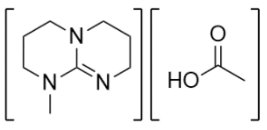	REF	84484500.00	0.00	0.00	0.00
	1h	68408800.00	18307.60	38540.10	0.08
	2h	79791000.00	175047.00	174703.00	0.44
	3h	66184700.00	217301.00	104381.00	0.48
	4h	74511800.00	724356.00	191304.00	1.21
	5h	65528300.00	1035380.00	234082.00	1.90
	6h	73712500.00	2782910.00	614932.00	4.41
	7h	59115300.00	2310500.00	616419.00	4.72
	8h	74154700.00	3475430.00	985239.00	5.67
	24h	65042200.00	8021190.00	1950690.00	13.29
	48h	46565200.00	10879700.00	2760910.00	22.66

Table S45 1 molar equivalent of [mTBDH][OAc], 1 molar equivalent of water and 0.2 equivalent of propanol.

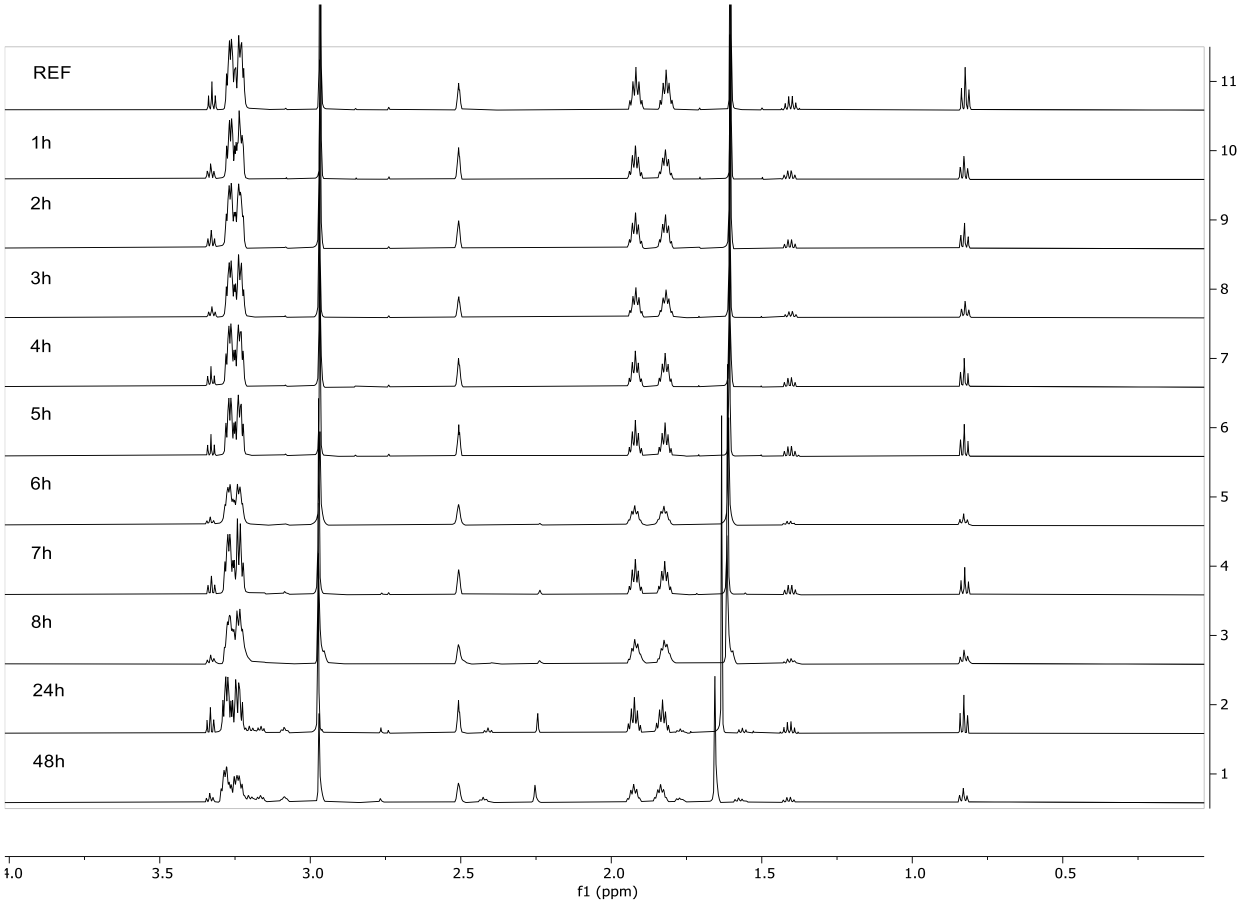

Figure S45 ^{1}H NMR 1 molar equivalent of [mTBDH][OAc], 1 molar equivalent of water and 0.2 equivalent of propanol.

Structure	Time	Integral N-Methyl	Integral HP1	Integral HP2	% HP
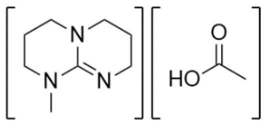	REF	64812100.00	0.00	0.00	0.00
	1h	61347200.00	32511.30	64286.20	0.16
	2h	57469100.00	326234.00	89793.90	0.72
	3h	64071900.00	388087.00	135482.00	0.81
	4h	34639700.00	422720.00	76244.50	1.42
	5h	64904500.00	924000.00	178794.00	1.67
	6h	62346200.00	1630630.00	342097.00	3.07
	7h	57966100.00	1900510.00	443752.00	3.89
	8h	67526400.00	2550020.00	616949.00	4.48
	24h	62620200.00	5871490.00	1179400.00	10.12
	48h	55048100.00	9768040.00	1896910.00	17.49

Table S46 1 molar equivalent of [mTBDH][OAc], 1 molar equivalent of water and 0.5 equivalent of propanol.

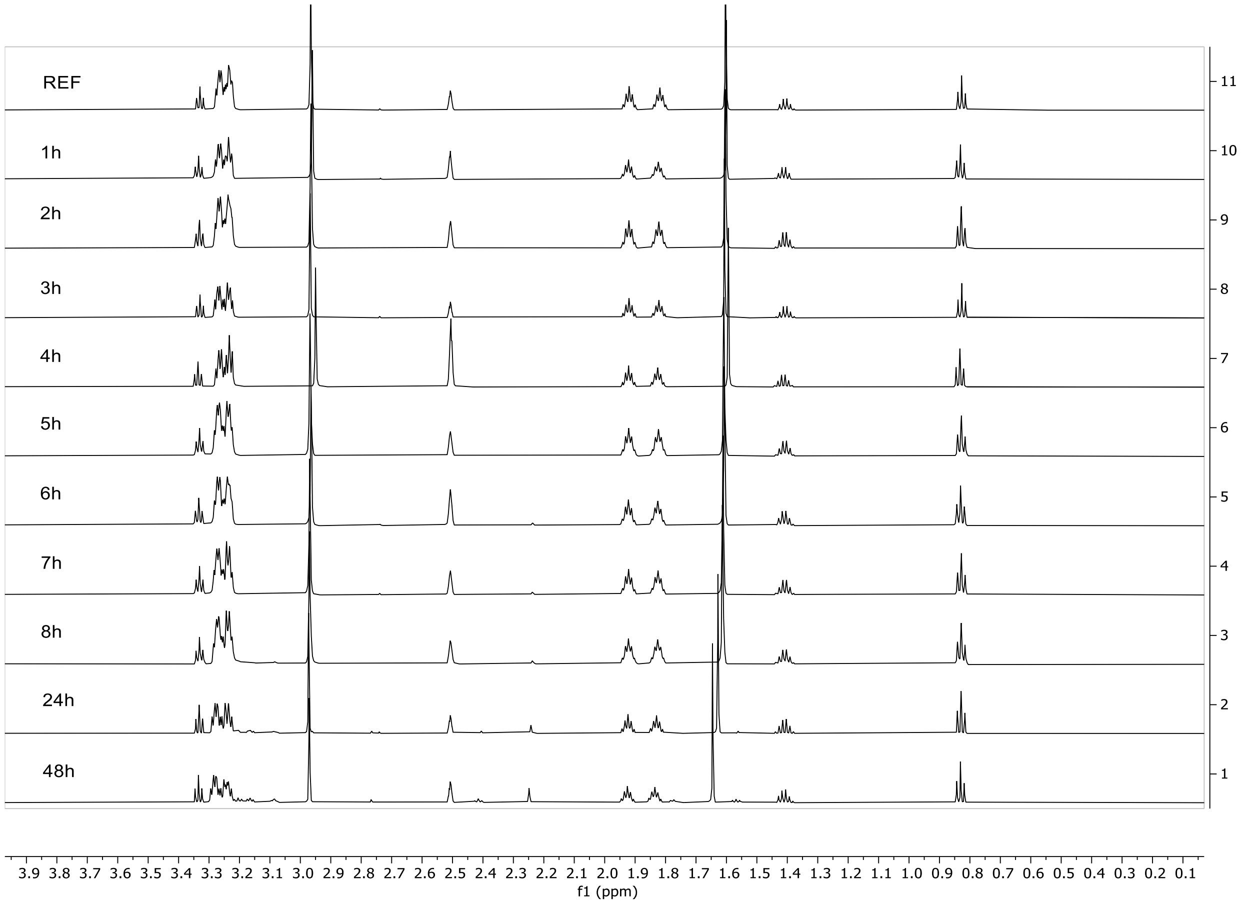

Figure S46 ^1H NMR 1 molar equivalent of [mTBDH][OAc], 1 molar equivalent of water and 0.5 equivalent of propanol.

Structure	Time	Integral N-Methyl	Integral HP1	Integral HP2	% HP
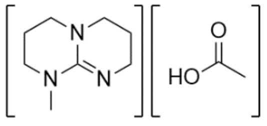	REF	57015800.00	79476.40	271673.00	0.00
	1h	67016500.00	243119.00	269865.00	0.76
	2h	56704200.00	307788.00	234908.00	0.95
	3h	52275700.00	397297.00	206769.00	1.14
	4h	54649400.00	498359.00	205603.00	1.27
	5h	51427100.00	994750.00	355901.00	2.56
	6h	22130900.00	585583.00	231556.00	3.56
	7h	63917600.00	2044520.00	686176.00	4.10
	8h	68008800.00	2786640.00	952591.00	5.21
	24h	50579700.00	4411450.00	1042500.00	9.73
	48h	58312800.00	8668970.00	1435500.00	14.77

Table S47 1 molar equivalent of [mTBDH][OAc], 1 molar equivalent of water and 1 equivalent of propanol.

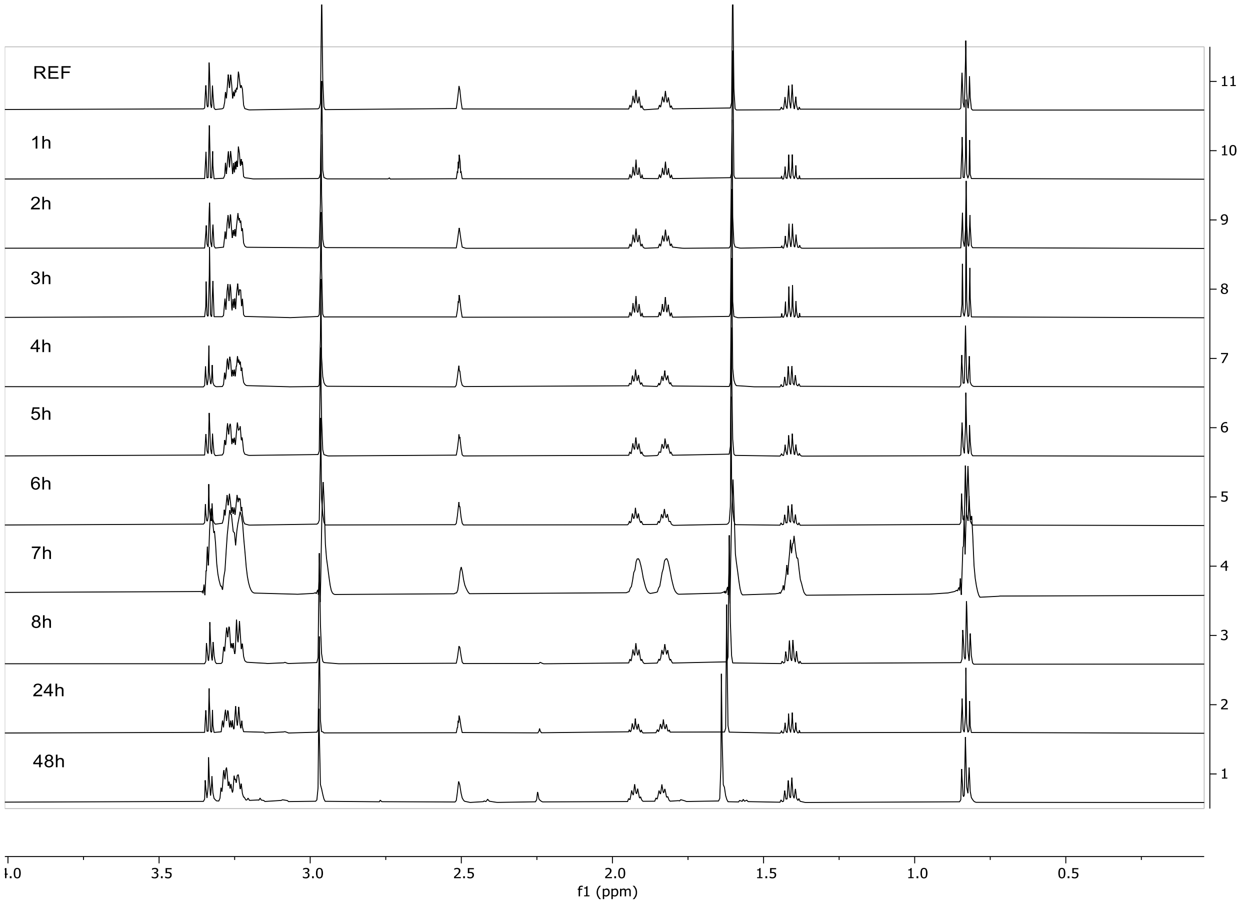

Figure S47 ^{1}H NMR 1 molar equivalent of [mTBDH][OAc], 1 molar equivalent of water and 1 equivalent of propanol.

Structure	Time	Integral N-Methyl	Integral HP1	Integral HP2		% HP
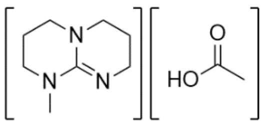	REF	37058200.00	170807.00	201449.00		0.00
	1h	49564900.00	318944.00	249547.00		1.13
	2h	44361900.00	353305.00	213274.00		1.26
	3h	41176200.00	392978.00	228685.00		1.49
	4h	41144800.00	409330.00	305697.00		1.71
	5h	40652900.00	561238.00	267665.00		2.00
	6h	44733700.00	804905.00	438262.00		2.70
	7h	47402600.00	911997.00	466868.00		2.83
	8h	48070900.00	1020670.00	363106.00		2.80
	24h	33430100.00	1096870.00	251963.00		3.88
	48h	45382500.00	3518530.00	719857.00		8.54

Table S48 1 molar equivalent of [mTBDH][OAc], 1 molar equivalent of water and 2 equivalents of propanol.

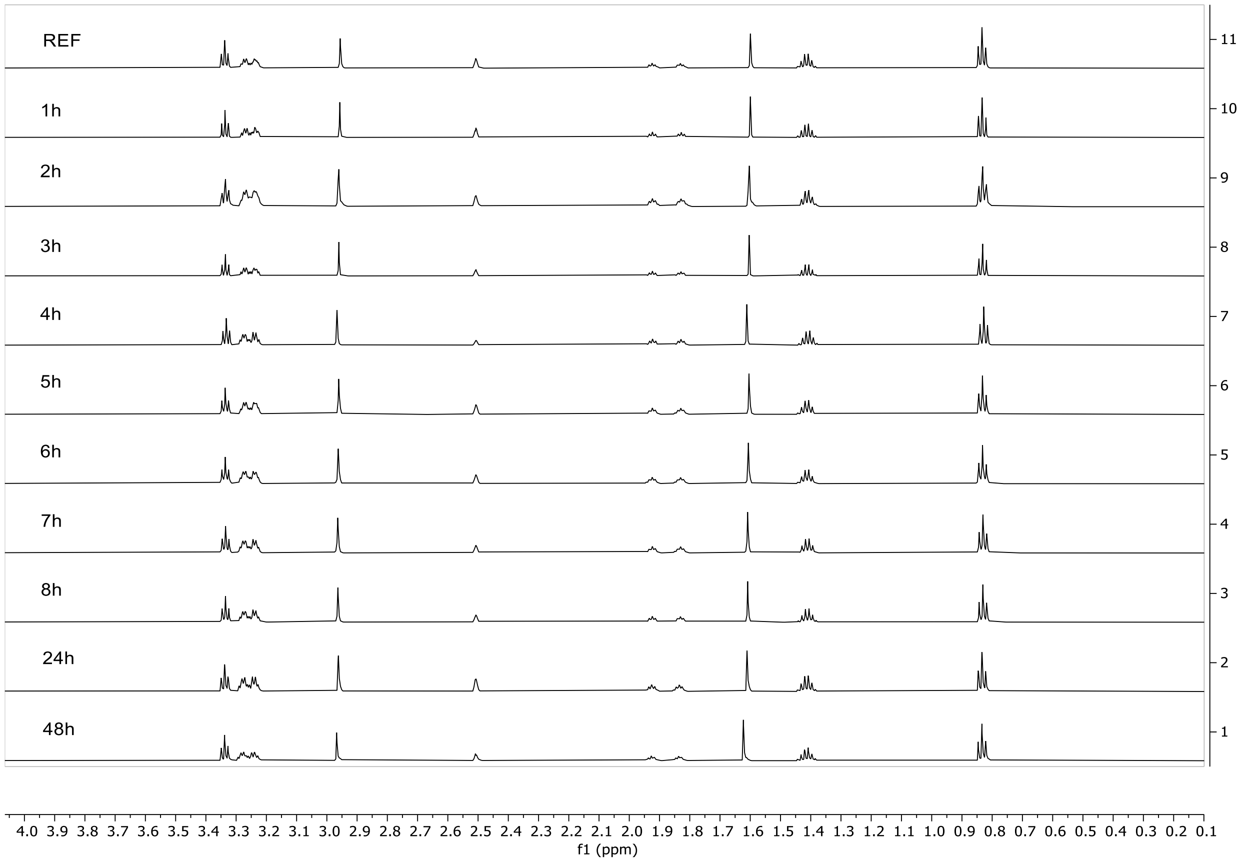

Figure S48 ^{1}H NMR 1 molar equivalent of [mTBDH][OAc], 1 molar equivalent of water and 2 equivalents of propanol.

f. [mTBDH][ClOAc] + water

Structure	Time	Integral N-Methyl	Integral HP	% HP
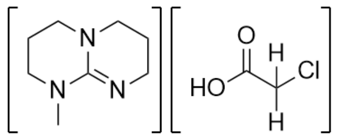	REF	104503000.00	0.00	0.00
	1h	93095900.00	0.00	0.00
	2h	92597000.00	0.00	0.00
	3h	110276000.00	0.00	0.00
	4h	87645400.00	0.00	0.00
	5h	92377800.00	0.00	0.00
	6h	91907800.00	0.00	0.00
	7h	99368900.00	0.00	0.00
	8h	92520900.00	0.00	0.00
	24h	104512000.00	0.00	0.00
	48h	90166700.00	0.00	0.00

Table S49 1 molar equivalent of [mTBDH][ClOAc], and 0.2 molar equivalent of water.

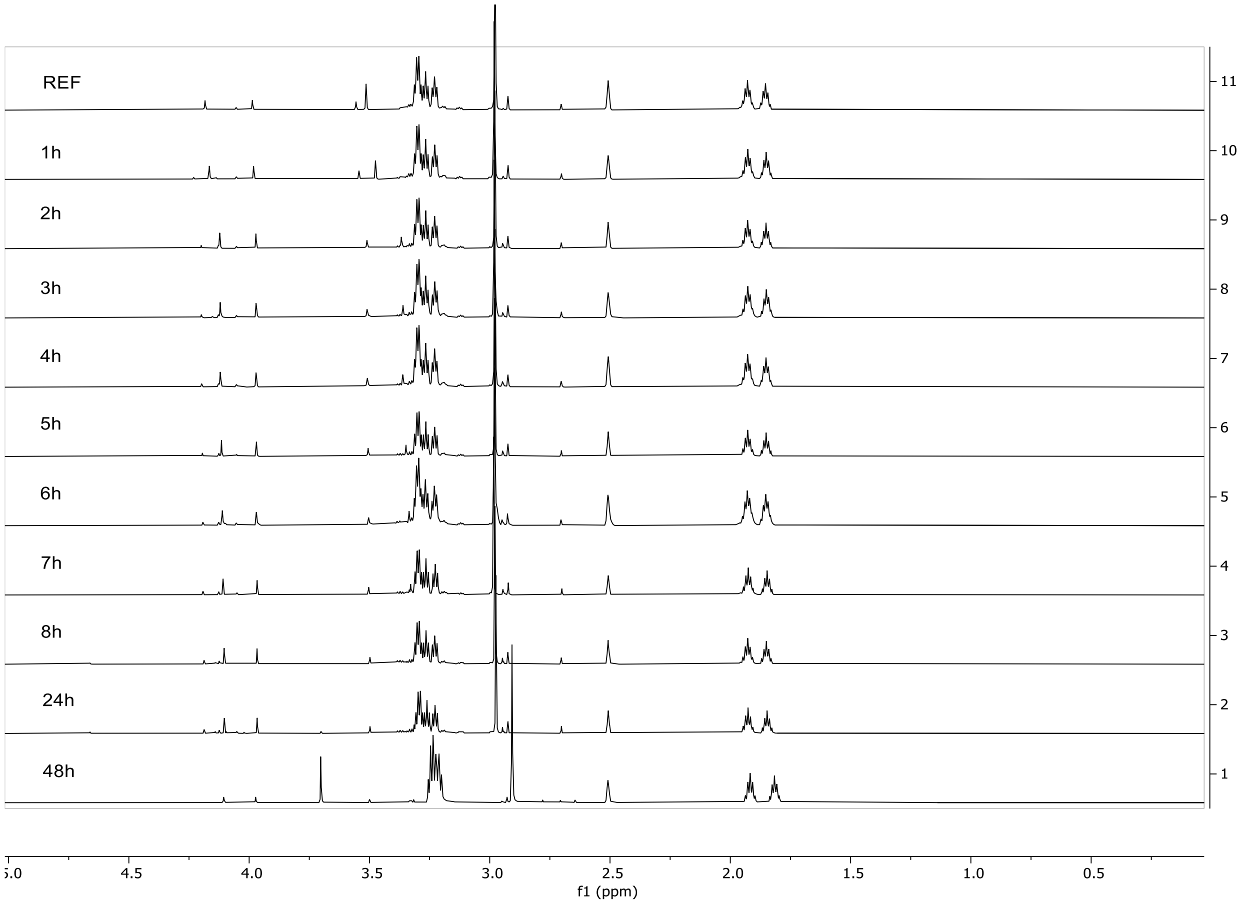

Figure S49 ^1H NMR 1 molar equivalent of [mTBDH][ClOAc], and 0.2 molar equivalent of water.

Structure	Time	Integral N-Methyl	Integral HP	% HP
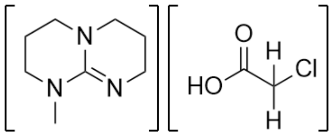	REF	93985000.00	0.00	0.00
	1h	70867500.00	0.00	0.00
	2h	84298500.00	0.00	0.00
	3h	78981400.00	0.00	0.00
	4h	91804600.00	0.00	0.00
	5h	82298900.00	0.00	0.00
	6h	72674200.00	0.00	0.00
	7h	81127700.00	0.00	0.00
	8h	79763300.00	0.00	0.00
	24h	76718000.00	0.00	0.00
	48h	75877500.00	0.00	0.00

Table S50 1 molar equivalent of [mTBDH][ClOAc], and 0,5 molar equivalent of water.

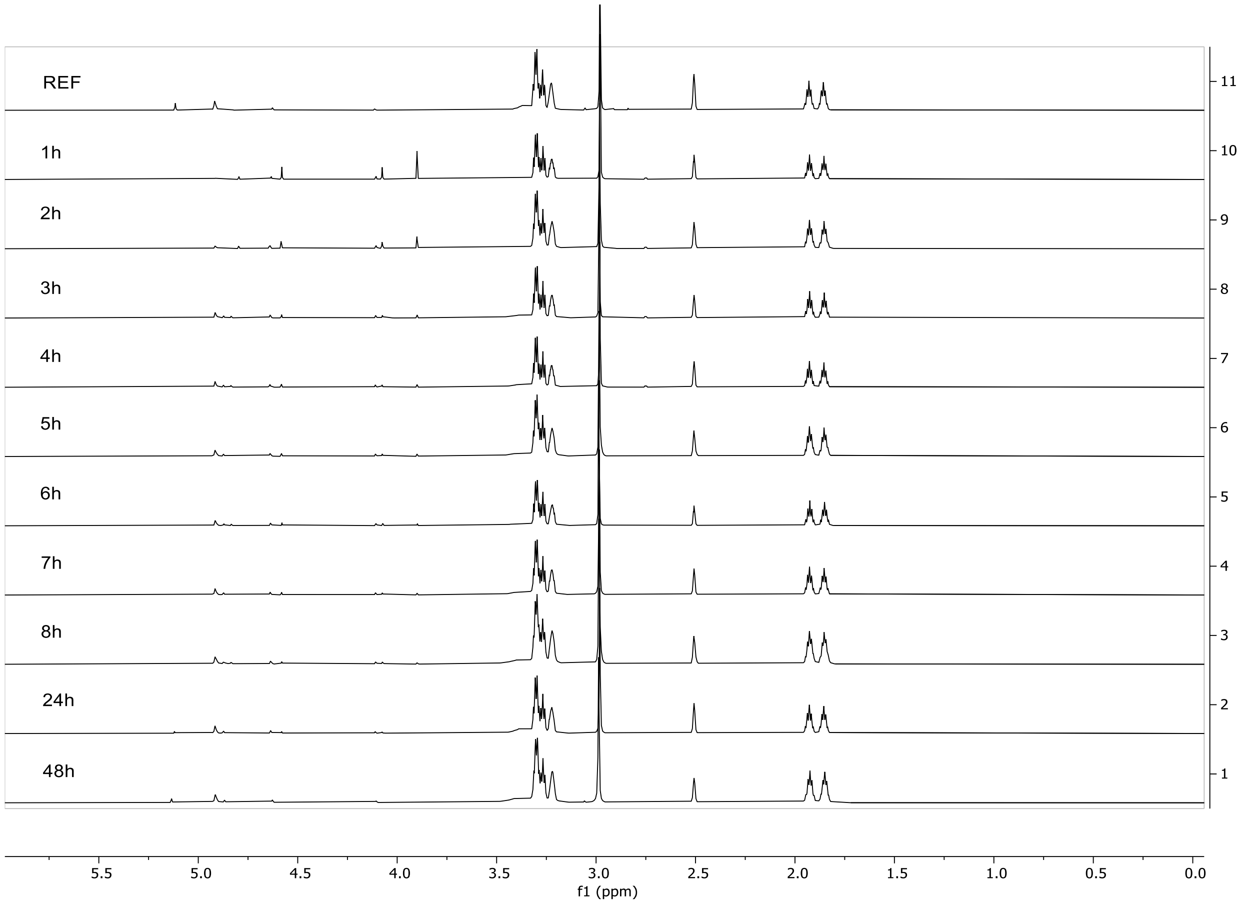

Figure S50 ¹H NMR 1 molar equivalent of [mTBDH][ClOAc], and 0,5 molar equivalent of water.

Structure	Time	Integral N-Methyl	Integral HP	% HP
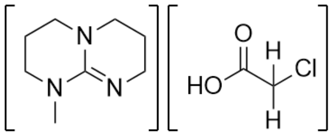	REF	93985000.00	0.00	0.00
	1h	70867500.00	0.00	0.00
	2h	84298500.00	0.00	0.00
	3h	78981400.00	0.00	0.00
	4h	91804600.00	0.00	0.00
	5h	82298900.00	0.00	0.00
	6h	72674200.00	0.00	0.00
	7h	81127700.00	0.00	0.00
	8h	79763300.00	0.00	0.00
	24h	76718000.00	0.00	0.00
	48h	75877500.00	0.00	0.00

Table S51 1 molar equivalent of [mTBDH][ClOAc], and 1 molar equivalent of water.

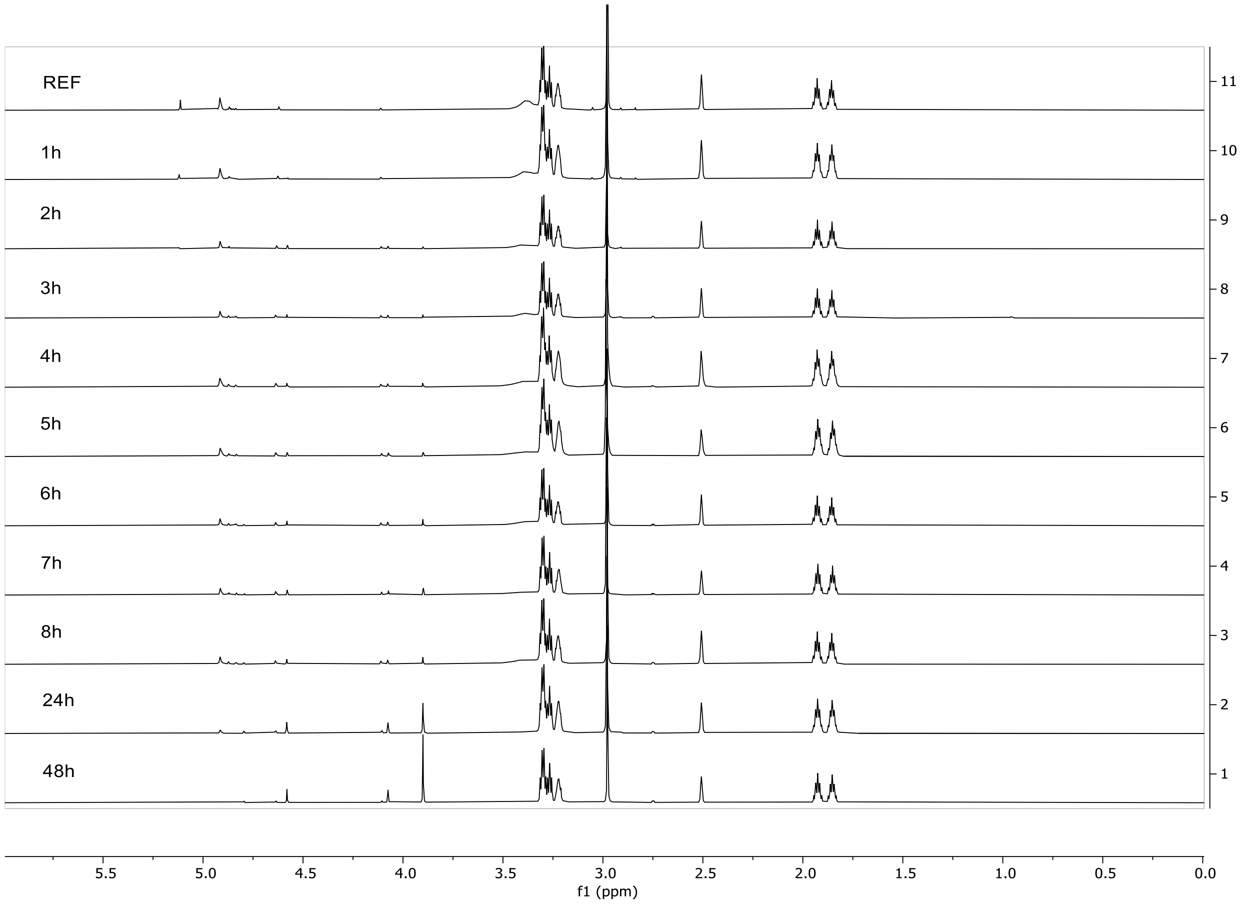

Figure S51 ^{1}H NMR 1 molar equivalent of [mTBDH][ClOAc], and 1 molar equivalent of water.

Structure	Time	integral N-Methyl	integral HP	% HP
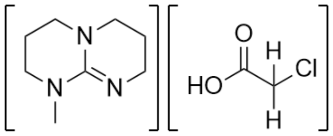	REF	77083000.00	0.00	0.00
	1h	64573900.00	0.00	0.00
	2h	61348100.00	0.00	0.00
	3h	64695500.00	0.00	0.00
	4h	67582900.00	0.00	0.00
	5h	71526600.00	0.00	0.00
	6h	72275500.00	0.00	0.00
	7h	69152100.00	0.00	0.00
	8h	66048100.00	0.00	0.00
	24h	54592000.00	0.00	0.00
	48h	65177800.00	0.00	0.00

Table S52 1 molar equivalent of [mTBDH][ClOAc], and 5 molar equivalents of water.

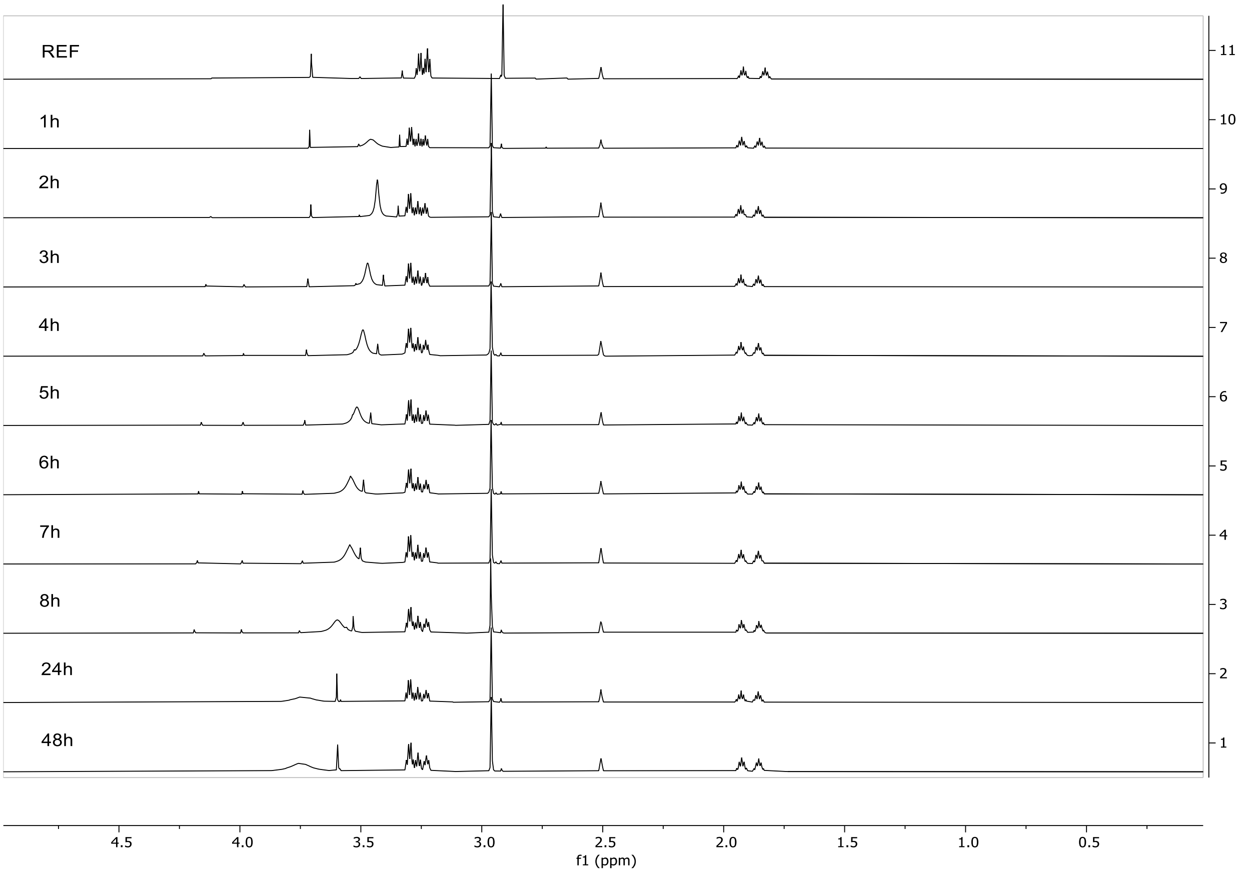

Figure S52 ^{1}H NMR 1 molar equivalent of [mTBDH][ClOAc], and 5 molar equivalents of water.

Structure	Time	Integral N-Methyl	Integral HP	% HP
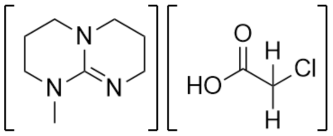	REF	36177400.00	0.00	0.00
	1h	39996600.00	0.00	0.00
	2h	43278100.00	0.00	0.00
	3h	36470500.00	0.00	0.00
	4h	34948500.00	0.00	0.00
	5h	35100800.00	0.00	0.00
	6h	38051500.00	0.00	0.00
	7h	41024100.00	0.00	0.00
	8h	39730200.00	0.00	0.00
	24h	37385900.00	0.00	0.00
	48h	43431400.00	0.00	0.00

Table S53 1 molar equivalent of [mTBDH][ClOAc], and 10 molar equivalents of water.

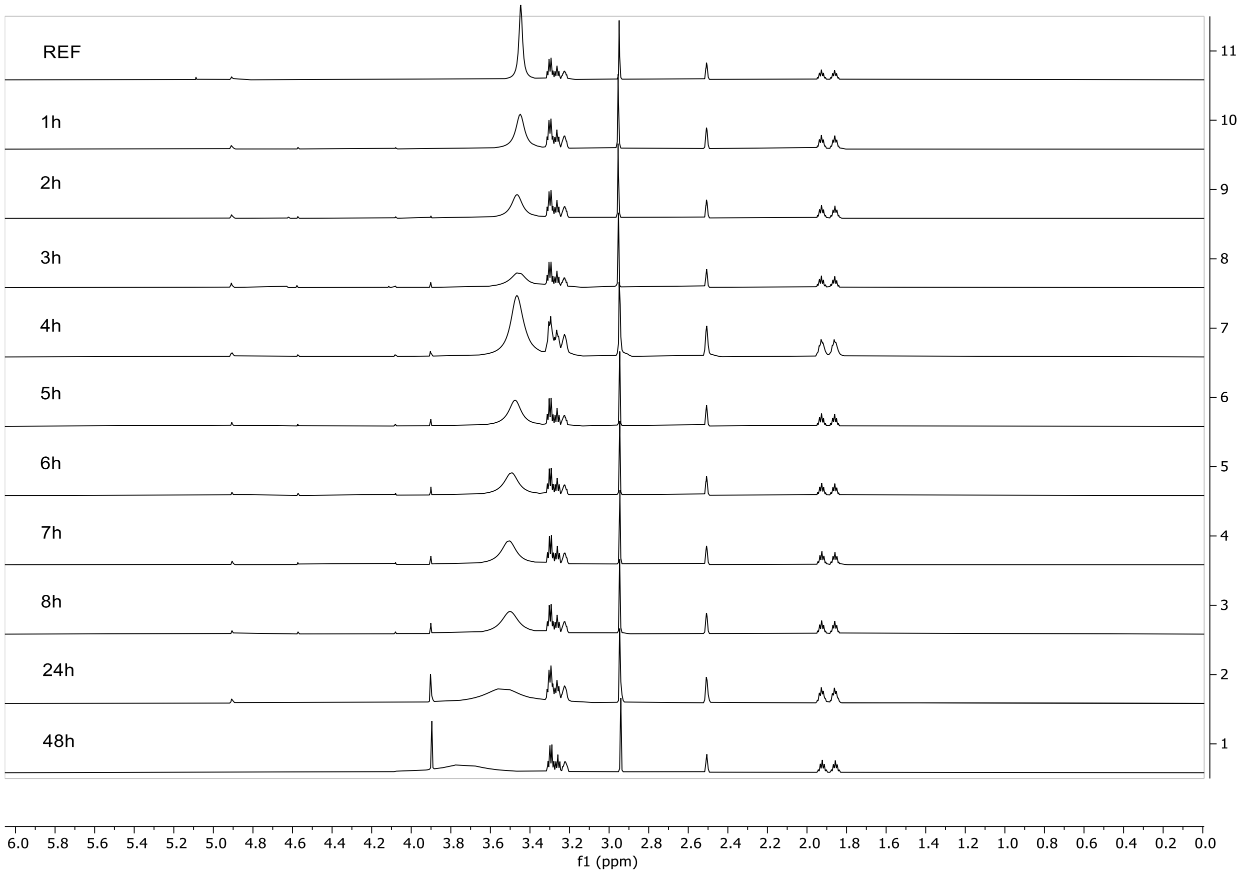

Figure S53 ^{1}H NMR 1 molar equivalent of [mTBDH][ClOAc], and 10 molar equivalents of water.

g. [mTBDH][Cl2OAc] + water

Structure	Time	Integral N-Methyl	Integral HP	% HP
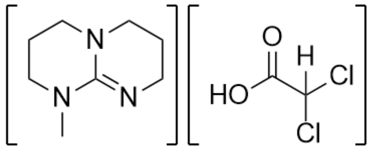	REF	104503000.00	0.00	0.00
	1h	93095900.00	0.00	0.00
	2h	92597000.00	0.00	0.00
	3h	110276000.00	0.00	0.00
	4h	87645400.00	0.00	0.00
	5h	92377800.00	0.00	0.00
	6h	91907800.00	0.00	0.00
	7h	99368900.00	0.00	0.00
	8hh	92520900.00	0.00	0.00
	24h	104512000.00	0.00	0.00
	48h	90166700.00	0.00	0.00

Table S54 1 molar equivalent of [mTBDH][Cl2OAc], and 0.2 molar equivalents of water.

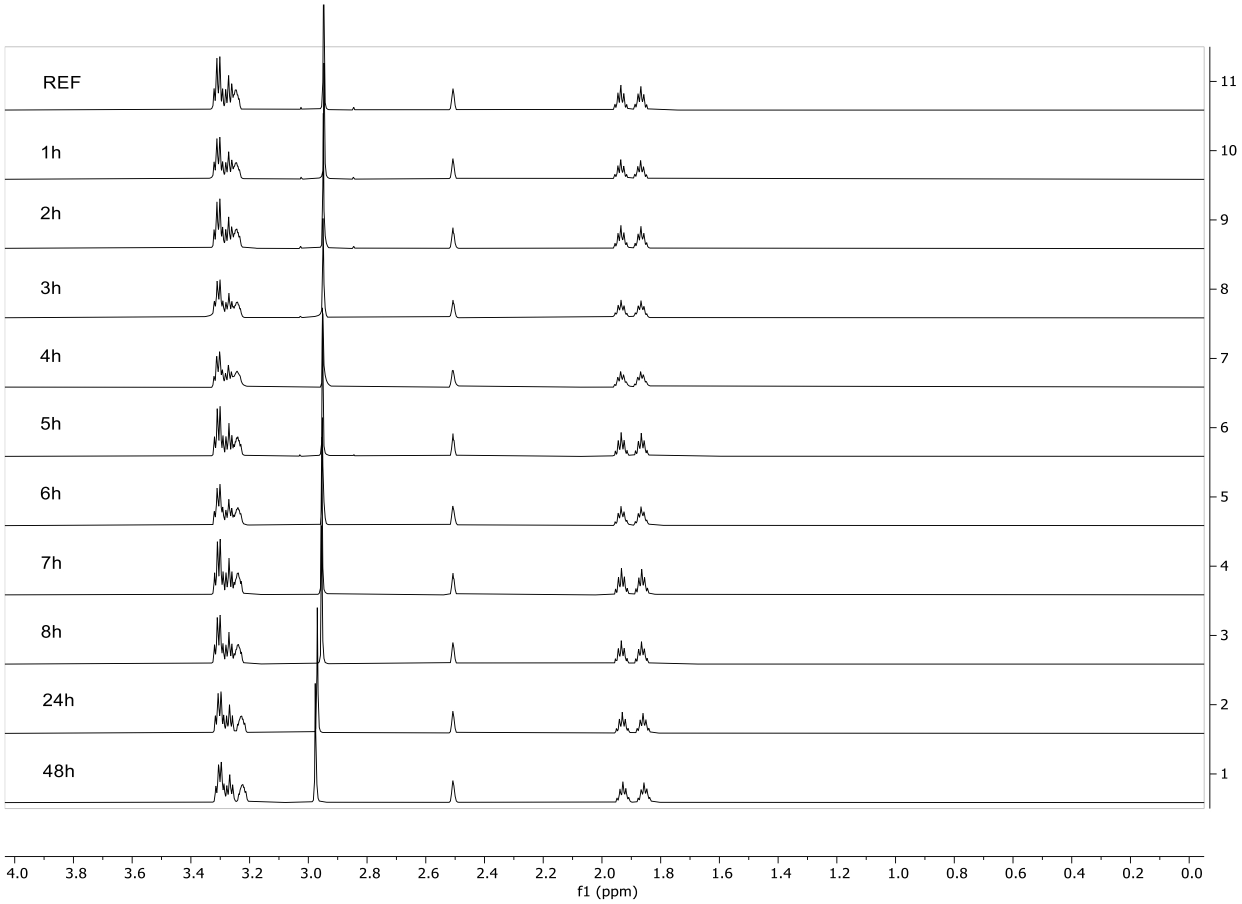

Figure S54 ^{1}H NMR 1 molar equivalent of [mTBDH][Cl2OAc], and 0.2 molar equivalents of water.

Structure	Time	Integral N-Methyl	Integral HP	% HP
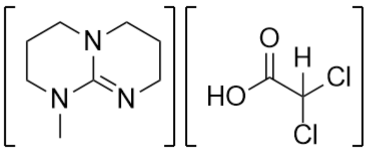	REF	95727400.00	0.00	0.00
	1h	104952000.00	0.00	0.00
	2h	80083700.00	0.00	0.00
	3h	88984100.00	0.00	0.00
	4h	105413000.00	0.00	0.00
	5h	94504700.00	0.00	0.00
	6h	96958700.00	0.00	0.00
	7h	82995400.00	0.00	0.00
	8h	89189500.00	0.00	0.00
	24h	87372400.00	0.00	0.00
	48h	98684300.00	0.00	0.00

Table S55 1 molar equivalent of [mTBDH][Cl2OAc], and 0.5 molar equivalents of water.

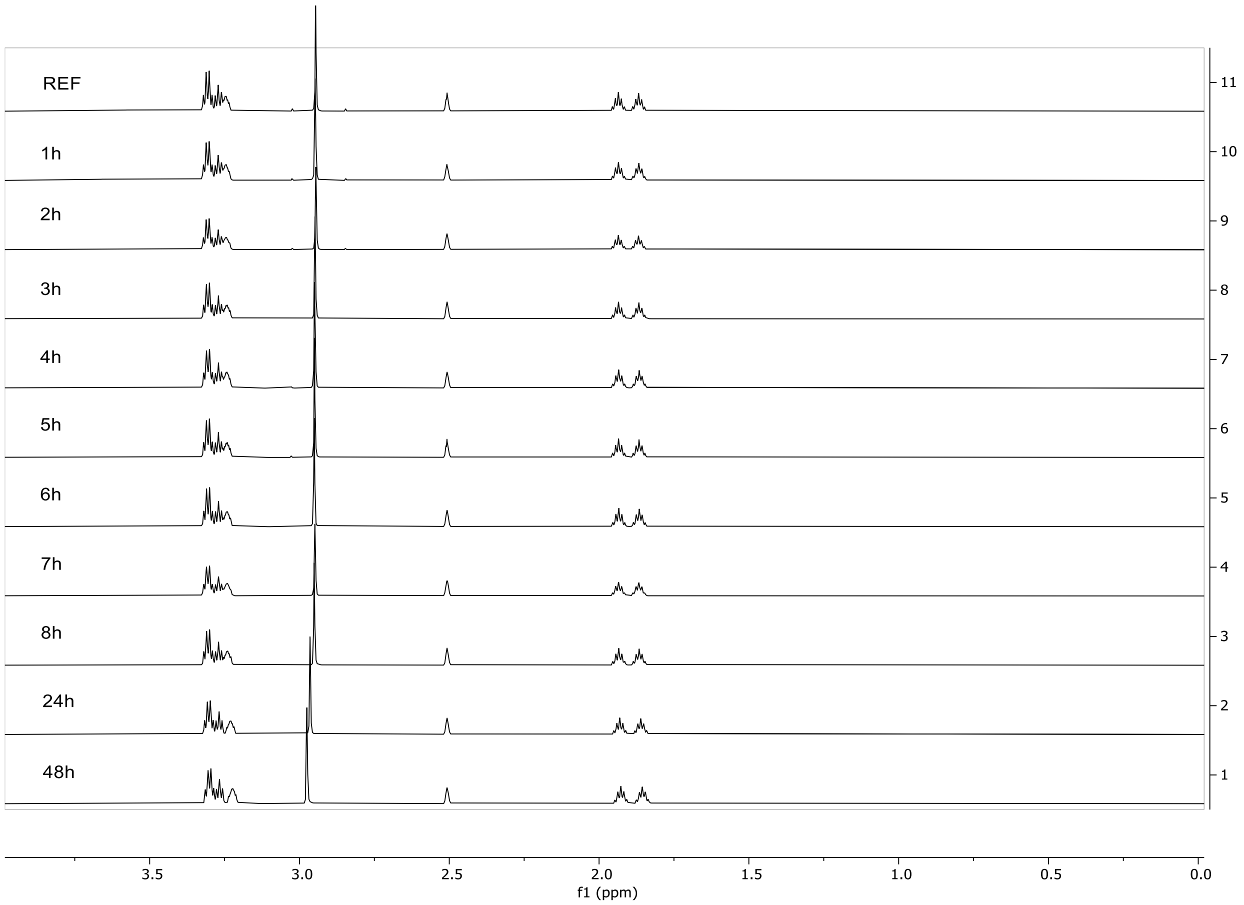

Figure S55 ^{1}H NMR 1 molar equivalent of [mTBDH][Cl2OAc], and 0.5 molar equivalents of water.

Structure	Time	Integral N-Methyl	Integral HP	% HP
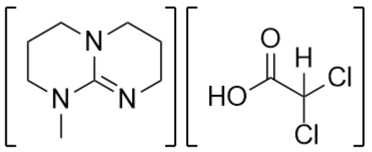	REF	95914400.00	0.00	0.00
	1h	91519400.00	0.00	0.00
	2h	80262000.00	0.00	0.00
	3h	87870300.00	0.00	0.00
	4h	94979200.00	0.00	0.00
	5h	85492800.00	0.00	0.00
	6h	94911700.00	0.00	0.00
	7h	90155300.00	0.00	0.00
	8h	87162900.00	0.00	0.00
	24h	84835700.00	0.00	0.00
	48h	84549700.00	0.00	0.00

Table S56 1 molar equivalent of [mTBDH][Cl2OAc], and 1 molar equivalents of water.

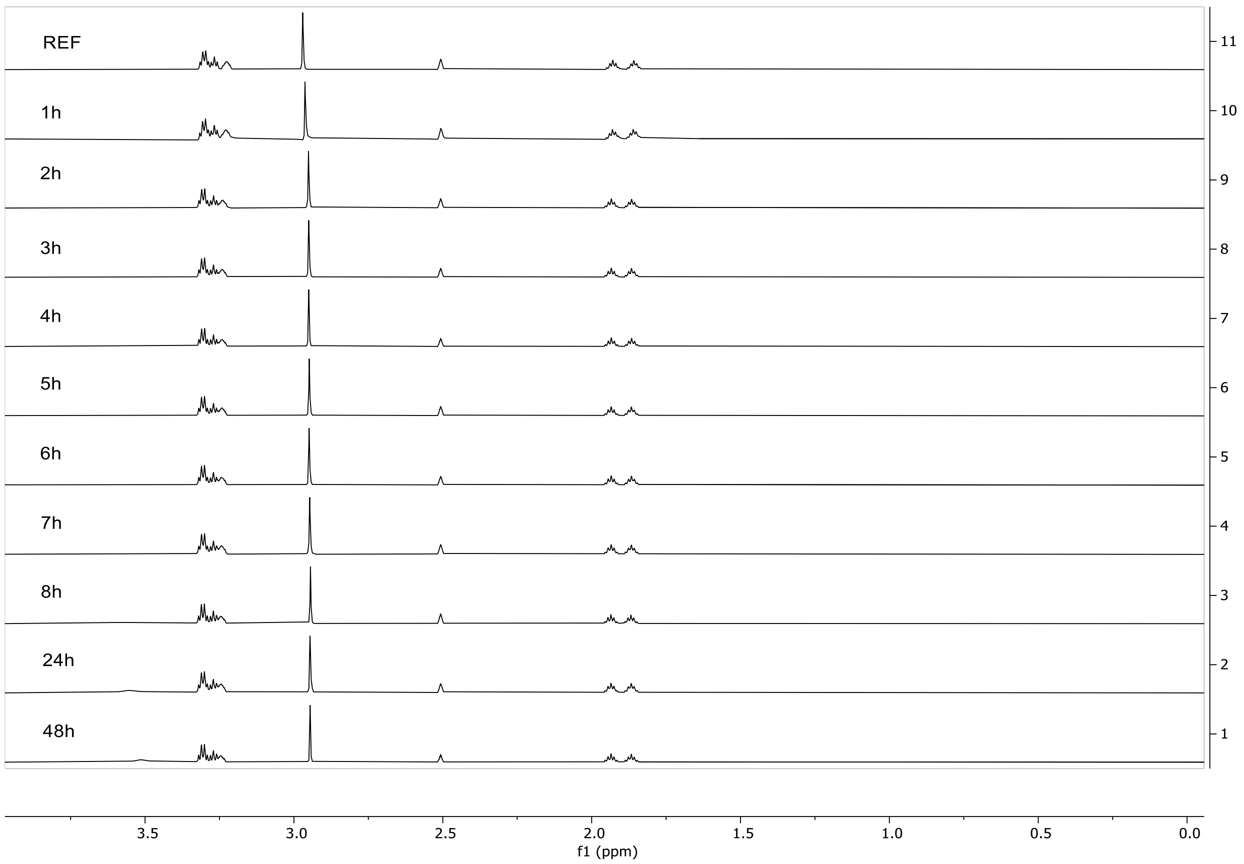

Figure S56 ^{1}H NMR 1 molar equivalent of [mTBDH][Cl2OAc], and 1 molar equivalents of water.

Structure	Time	Integral N-Methyl	Integral HP	% HP
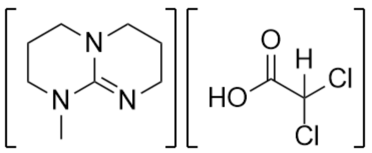	REF	74052700.00	0.00	0.00
	1h	70707400.00	0.00	0.00
	2h	71223200.00	0.00	0.00
	3h	75359100.00	0.00	0.00
	4h	69089200.00	0.00	0.00
	5h	74640600.00	0.00	0.00
	6h	66418700.00	0.00	0.00
	7h	70187400.00	0.00	0.00
	24h	66157100.00	0.00	0.00
	48h	90166700.00	0.00	0.00

Table S57 1 molar equivalent of [mTBDH][Cl2OAc], and 5 molar equivalents of water.

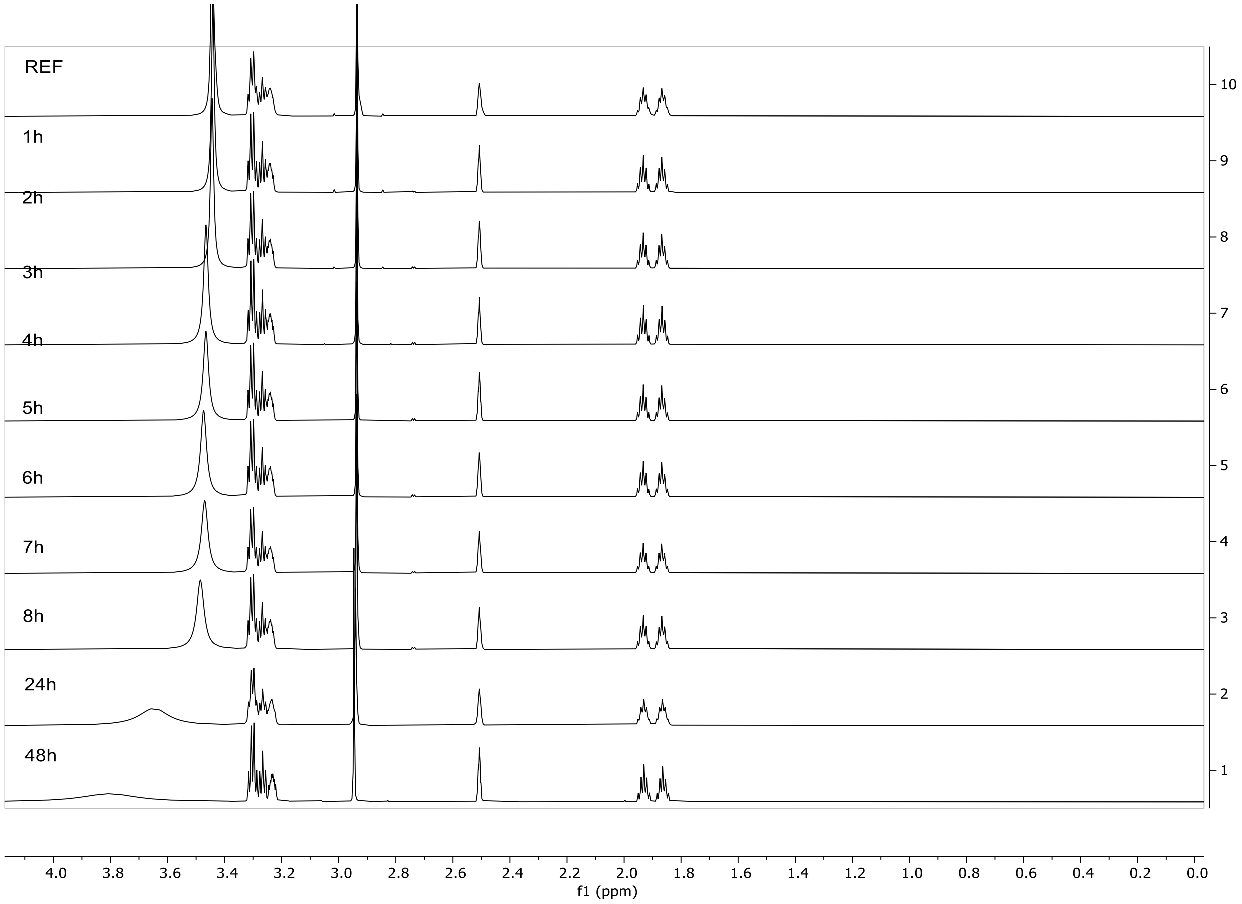

Figure S57 ^{1}H NMR 1 molar equivalent of [mTBDH][Cl2OAc], and 5 molar equivalents of water.

Structure	Time	Integral N-Methyl	Integral HP	% HP
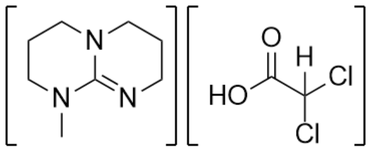	REF	59747200.00	0.00	0.00
	1h	54866300.00	0.00	0.00
	2h	54554900.00	0.00	0.00
	3h	53412400.00	0.00	0.00
	4h	58450400.00	0.00	0.00
	5h	57131900.00	0.00	0.00
	6h	60565300.00	0.00	0.00
	7h	44876400.00	0.00	0.00
	8h	49536000.00	0.00	0.00
	24h	48412500.00	0.00	0.00
	48h	49651700.00	0.00	0.00

Table S58 1 molar equivalent of [mTBDH][Cl2OAc], and 10 molar equivalents of water.

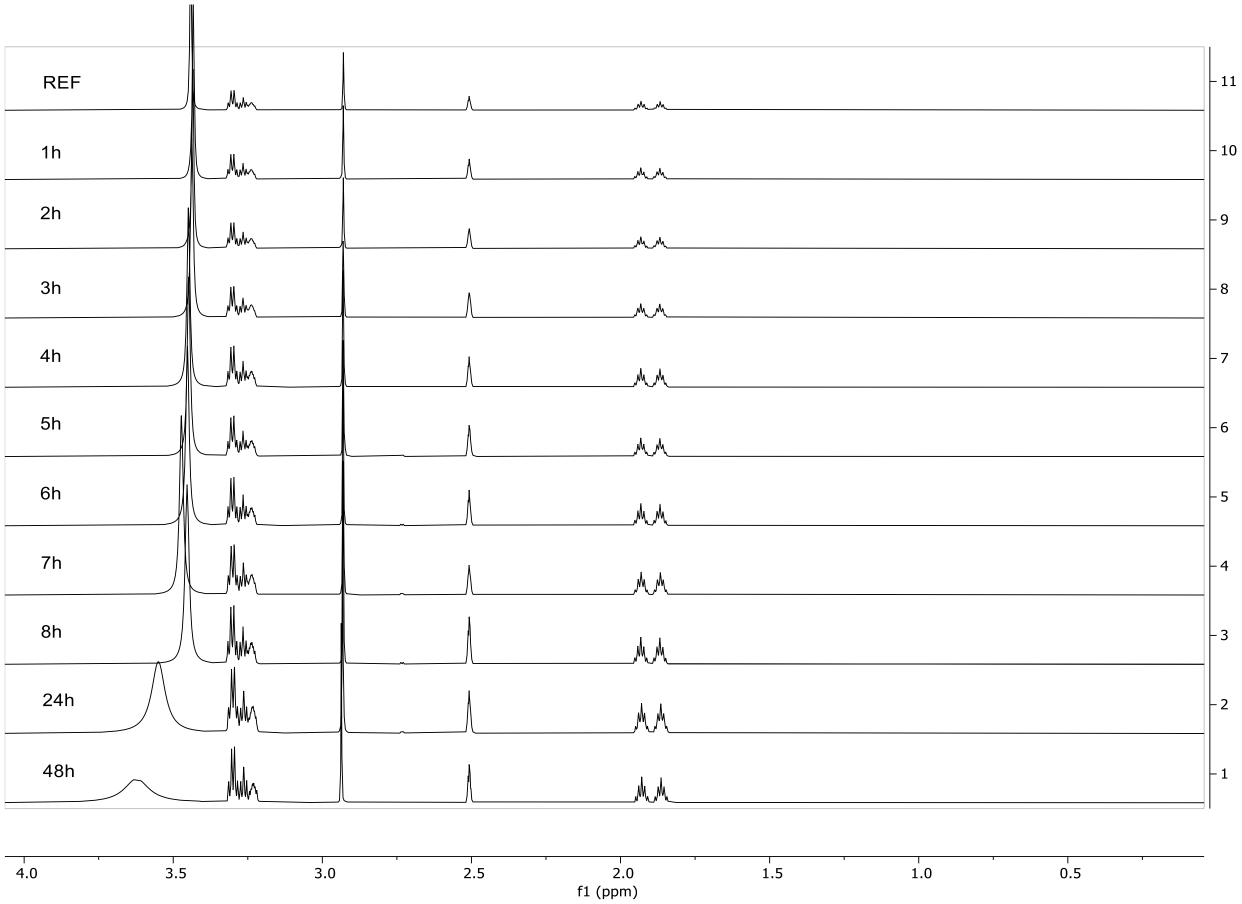

Figure S58 ¹H NMR 1 molar equivalent of [mTBDH][Cl2OAc], and 10 molar equivalents of water.

h. [mTBDH][Cl3OAc] + water

Structure	Time	Integral N-Methyl	Integral HP	% HP
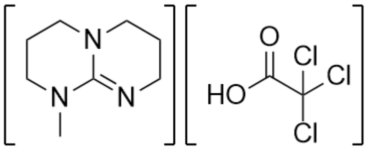	REF	96335100.00	0.00	0.00
	1h	110222000.00	0.00	0.00
	2h	105830000.00	0.00	0.00
	3h	104821000.00	0.00	0.00
	4h	103922000.00	0.00	0.00
	5h	102696000.00	0.00	0.00
	6h	102966000.00	0.00	0.00
	7h	116841000.00	0.00	0.00
	8h	119564000.00	0.00	0.00
	24h	96649800.00	0.00	0.00
	48h	87251700.00	0.00	0.00

Table S59 1 molar equivalent of [mTBDH][Cl3OAc], and 0.2 molar equivalents of water.

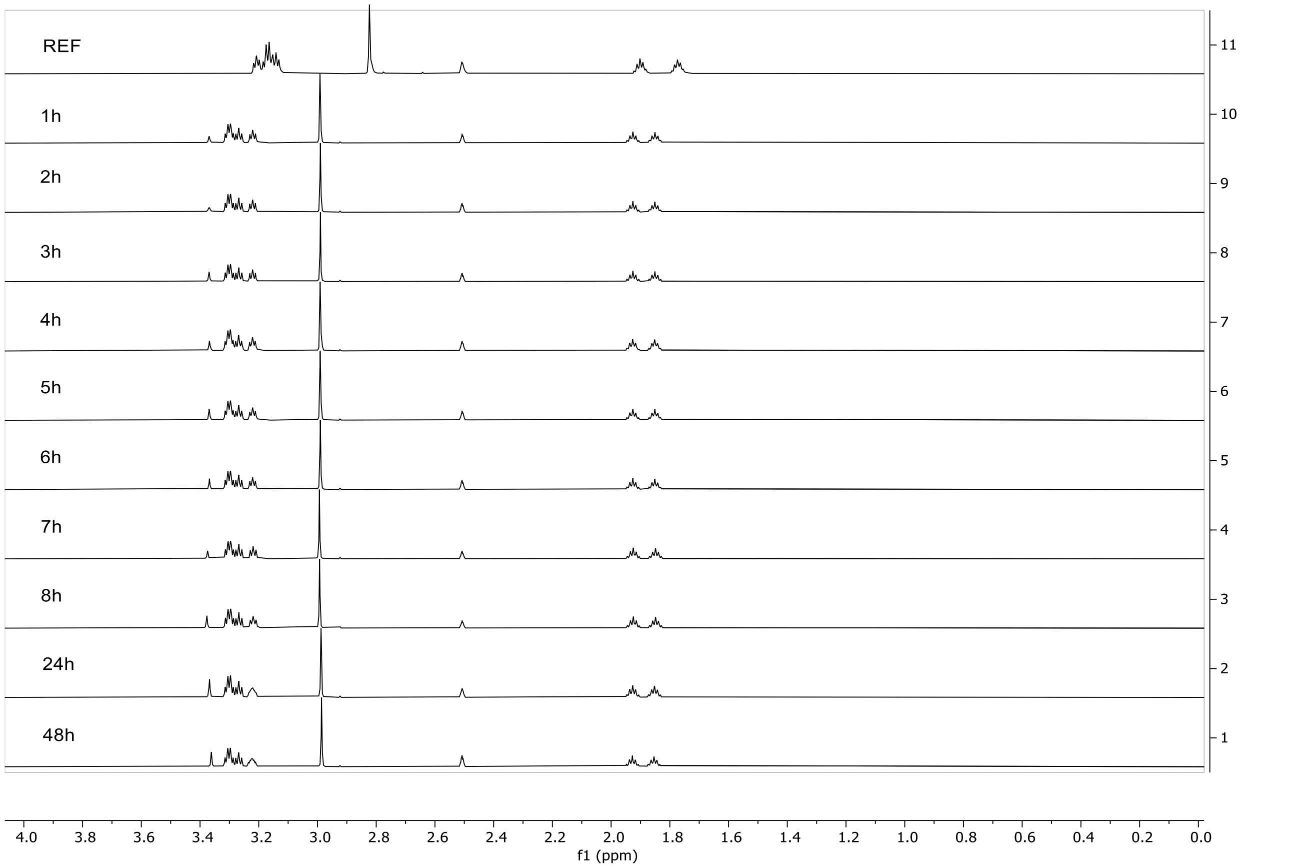

Figure S59 ^{1}H NMR 1 molar equivalent of [mTBDH][Cl3OAc], and 0.2 molar equivalents of water.

Structure	Time	Integral N-Methyl	Integral HP	% HP
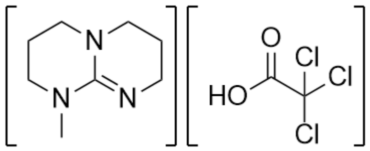	REF	81627100.00	0.00	0.00
	1	99403500.00	0.00	0.00
	2	90889900.00	0.00	0.00
	3	113107000.00	0.00	0.00
	4	102479000.00	0.00	0.00
	5	96517900.00	0.00	0.00
	6	95205500.00	0.00	0.00
	7	93837500.00	0.00	0.00
	8	108069000.00	0.00	0.00
	24	113480000.00	0.00	0.00
	48	92516900.00	0.00	0.00

Table S60 1 molar equivalent of [mTBDH][Cl3OAc], and 0.5 molar equivalents of water.

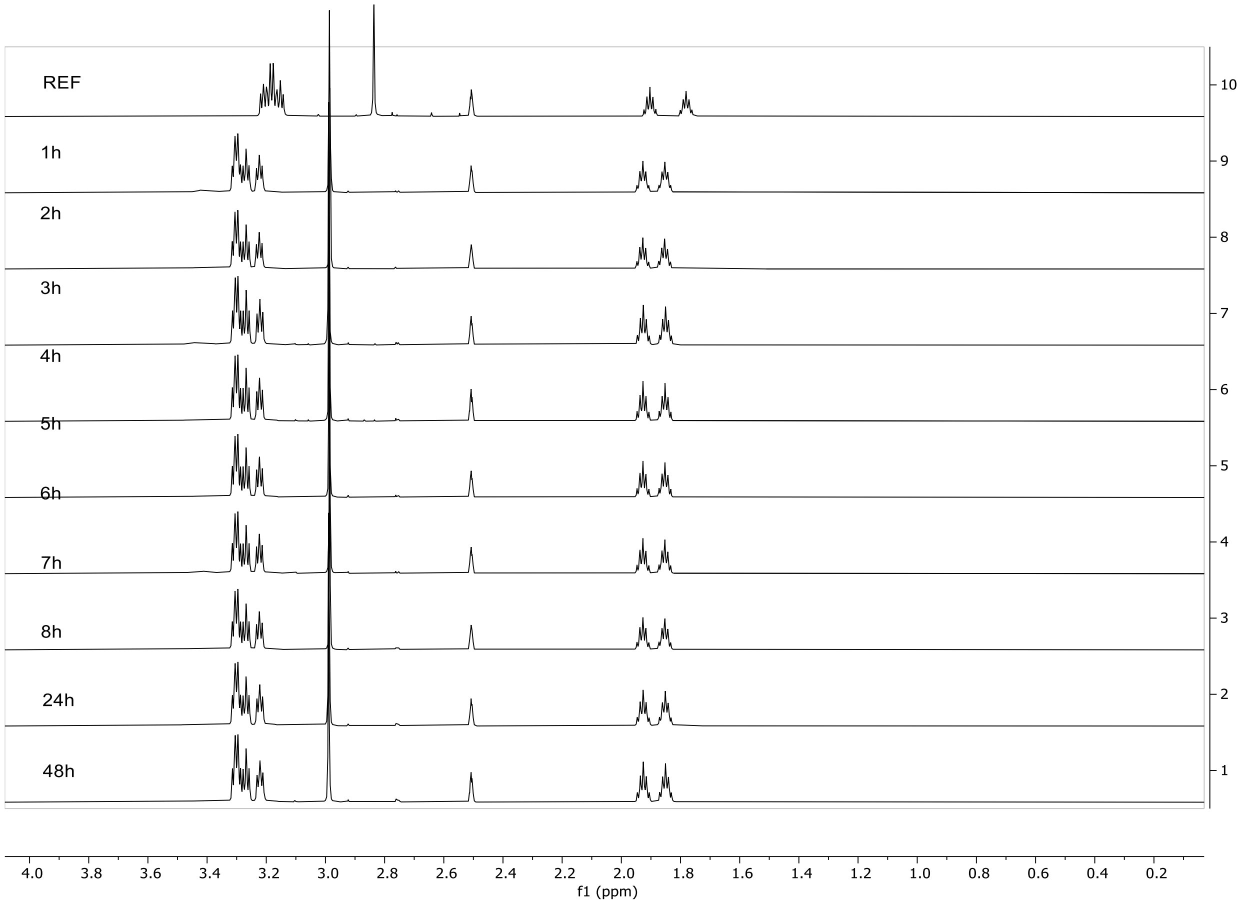

Figure S60 ^{1}H NMR 1 molar equivalent of [mTBDH][Cl3OAc], and 0.5 molar equivalents of water.

Structure	Time	Integral N-Methyl	Integral HP	% HP
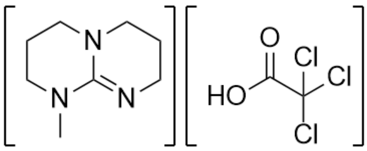	REF	90018900.00	0.00	0.00
	1h	100833000.00	0.00	0.00
	2h	113748000.00	0.00	0.00
	3h	94176000.00	0.00	0.00
	4h	106493000.00	0.00	0.00
	5h	103751000.00	0.00	0.00
	6h	106153000.00	0.00	0.00
	7h	118480000.00	0.00	0.00
	8h	104895000.00	0.00	0.00
	24h	110603000.00	0.00	0.00
	48h	100934000.00	0.00	0.00

Table S61 1 molar equivalent of [mTBDH][Cl3OAc], and 1 molar equivalents of water.

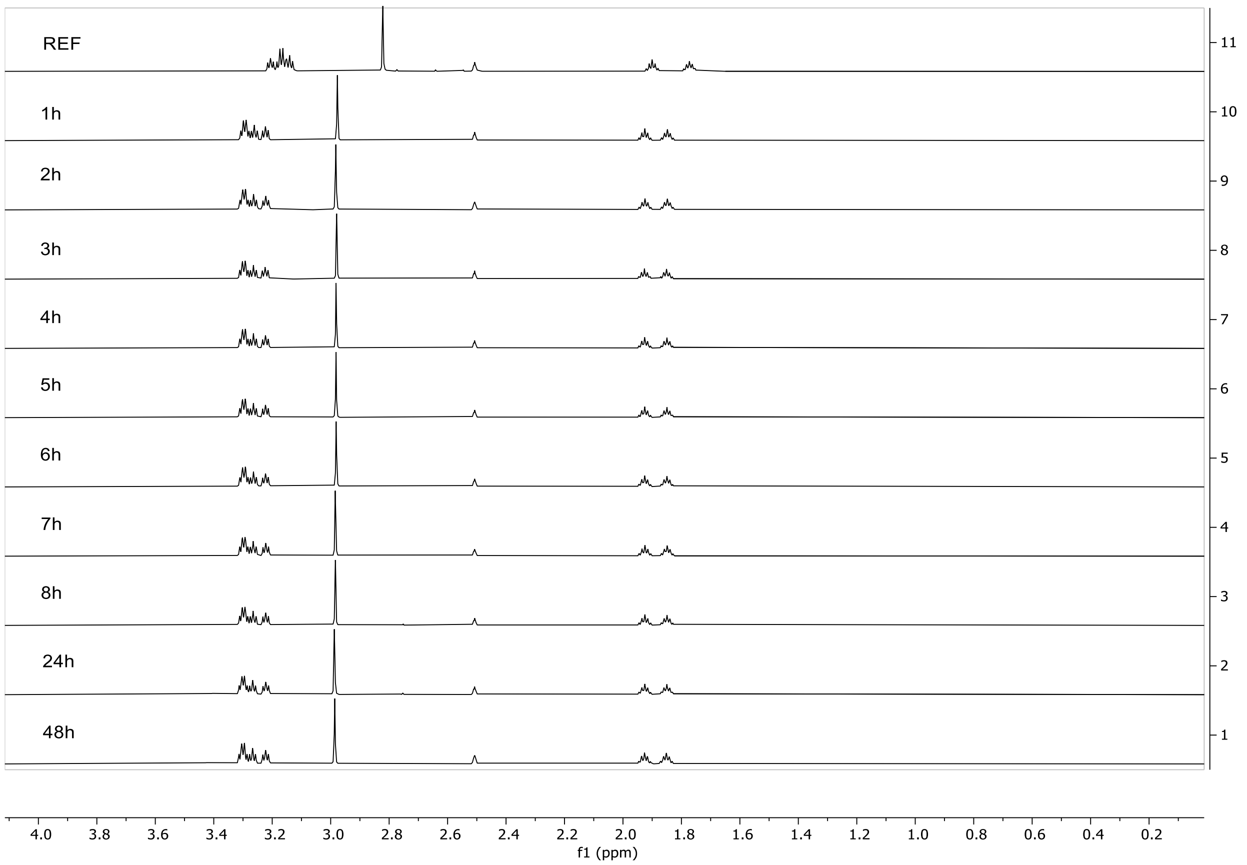

Figure S61 ^{1}H NMR 1 molar equivalent of [mTBDH][Cl3OAc], and 1 molar equivalents of water.

Structure	Time	Integral N-Methyl	Integral HP	% HP
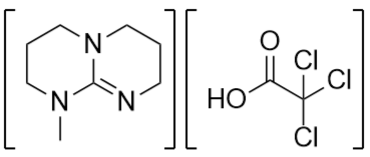	REF	54843500.00	0.00	0.00
	1h	75534200.00	0.00	0.00
	2h	76088900.00	0.00	0.00
	3h	74796200.00	0.00	0.00
	4h	68778400.00	0.00	0.00
	5h	66787400.00	0.00	0.00
	6h	70216200.00	0.00	0.00
	7h	72818500.00	0.00	0.00
	8h	72453800.00	0.00	0.00
	24h	77692700.00	0.00	0.00
	48h	67356500.00	0.00	0.00

Table S62 1 molar equivalent of [mTBDH][Cl3OAc], and 5 molar equivalents of water.

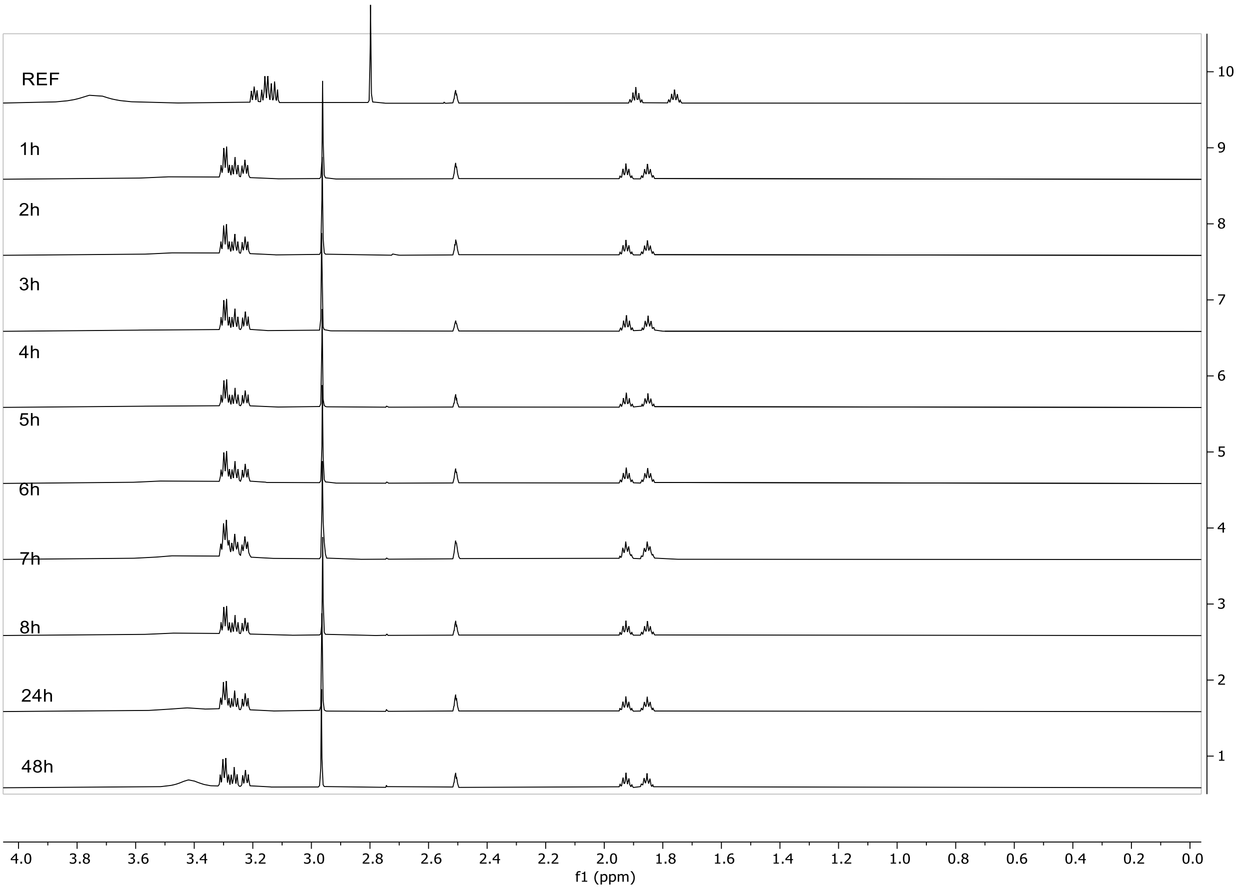

Figure S62 ^{1}H NMR 1 molar equivalent of [mTBDH][Cl3OAc], and 5 molar equivalents of water.

Structure	Time	Integral N-Methyl	Integral HP	% HP
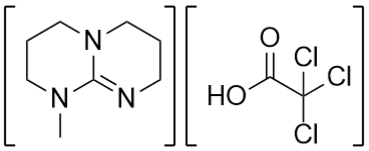	REF	56061600.00	0.00	0.00
	1h	71439800.00	0.00	0.00
	2h	72501900.00	0.00	0.00
	3h	77403300.00	0.00	0.00
	4h	69895500.00	0.00	0.00
	5h	76007300.00	0.00	0.00
	6h	58829200.00	0.00	0.00
	7h	68086700.00	0.00	0.00
	8h	72990100.00	0.00	0.00
	24h	70579600.00	0.00	0.00
	48h	67356500.00	0.00	0.00

Table S63 1 molar equivalent of [mTBDH][Cl3OAc], and 10 molar equivalents of water.

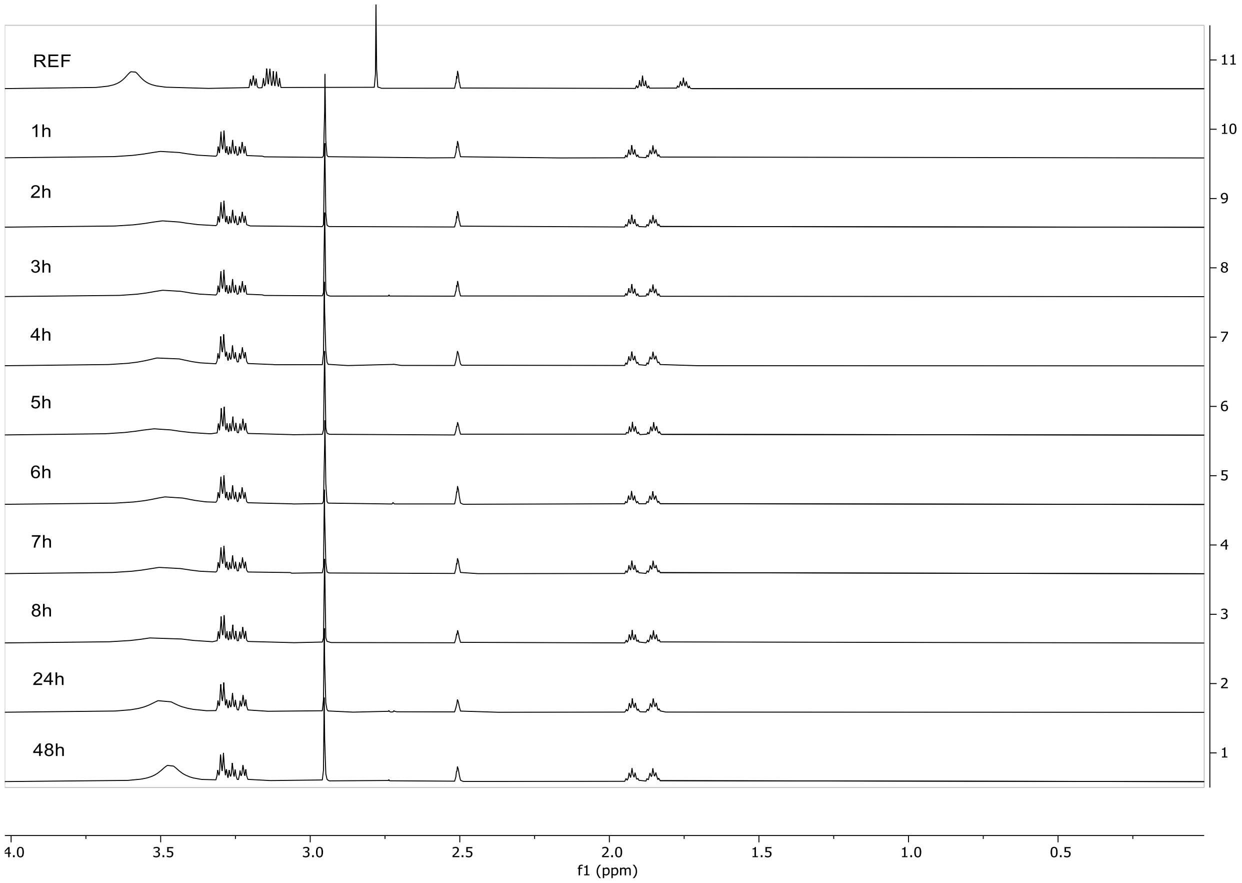

Figure S63 ^1H NMR 1 molar equivalent of [mTBDH][Cl3OAc], and 10 molar equivalents of water.

3. mTBN

a. mTBN + water over 48h at 90°C.

Structure	Time	Integral N-Methyl 1	Integral N-Methyl 2	Integral N Methyl total	Integral HP 1	Integral HP 2	Integral HP Total	% HP
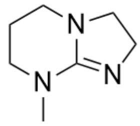	REF	298091000.00	195578000.00	493669000.00	55132.70	1681960.00	1737092.70	0.00
	1h	349551000.00	230791000.00	580342000.00	481570.00	1864300.00	2345870.00	0.40
	2h	298004000.00	196620000.00	494624000.00	438015.00	1731190.00	2169205.00	0.44
	3h	338630000.00	222949000.00	561579000.00	685109.00	2071850.00	2756959.00	0.49
	4h	320946000.00	212222000.00	533168000.00	1053340.00	2011020.00	3064360.00	0.57
	5h	255585000.00	169078000.00	424663000.00	663843.00	1792650.00	2456493.00	0.58
	6h	252087000.00	166595000.00	418682000.00	1128170.00	1634740.00	2762910.00	0.66
	7h	269936000.00	178838000.00	448774000.00	1911480.00	2053020.00	3964500.00	0.88
	8h	219878000.00	145905000.00	365783000.00	1626550.00	1651060.00	3277610.00	0.89
	24h	245837000.00	167868000.00	413705000.00	7777580.00	3859430.00	11637010.00	2.74
	48h	89825800.00	63212800.00	153038600.00	5541390.00	2298140.00	7839530.00	4.87

Table S64 1 molar equivalent of mTBN and 0.2 molar equivalents of water.

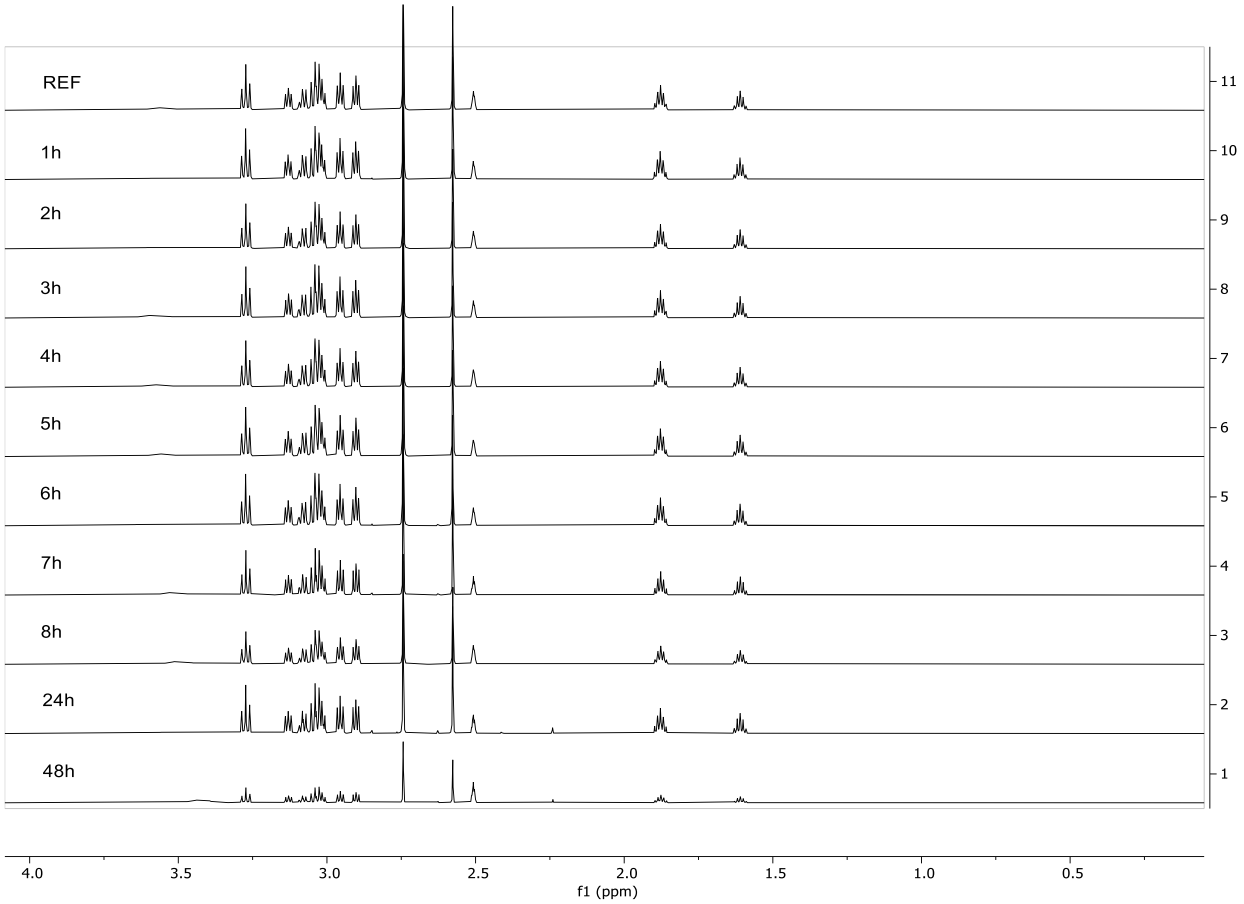

Figure S64 ^{1}H NMR 1 molar equivalent of mTBN and 0.2 molar equivalents of water.

Structure	Time	Integral N-Methyl 1	Integral N-Methyl 2	Integral N Methyl total	Integral HP 1	Integral HP 2	Integral HP Total	% HP
	REF	280048000.00	184077000.00	464125000.00	273318.00	1754780.00	2028098.00	0.00
	1h	73777000.00	49340300.00	123117300.00	412100.00	556470.00	968570.00	0.78
	2h	236071000.00	157586000.00	393657000.00	2255510.00	2202360.00	4457870.00	1.12
	3h	314600000.00	210682000.00	525282000.00	3884350.00	3312120.00	7196470.00	1.35
	4h	321153000.00	215468000.00	536621000.00	5746180.00	3586550.00	9332730.00	1.71
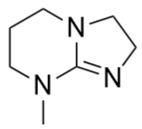	5h	231491000.00	155993000.00	387484000.00	4844040.00	3214270.00	8058310.00	2.04
	6h	275714000.00	186703000.00	462417000.00	7491140.00	4074990.00	11566130.00	2.44
	7h	193207000.00	130011000.00	323218000.00	6540140.00	2733420.00	9273560.00	2.79
	8h	226046000.00	154146000.00	380192000.00	8815560.00	3968070.00	12783630.00	3.25
	24h	282851000.00	204179000.00	487030000.00	37986600.00	16388900.00	54375500.00	10.04
	48h	54085500.00	41427500.00	95513000.00	13528400.00	5689460.00	19217860.00	16.75

Table S65 1 molar equivalent of mTBN and 0.5 molar equivalents of water.

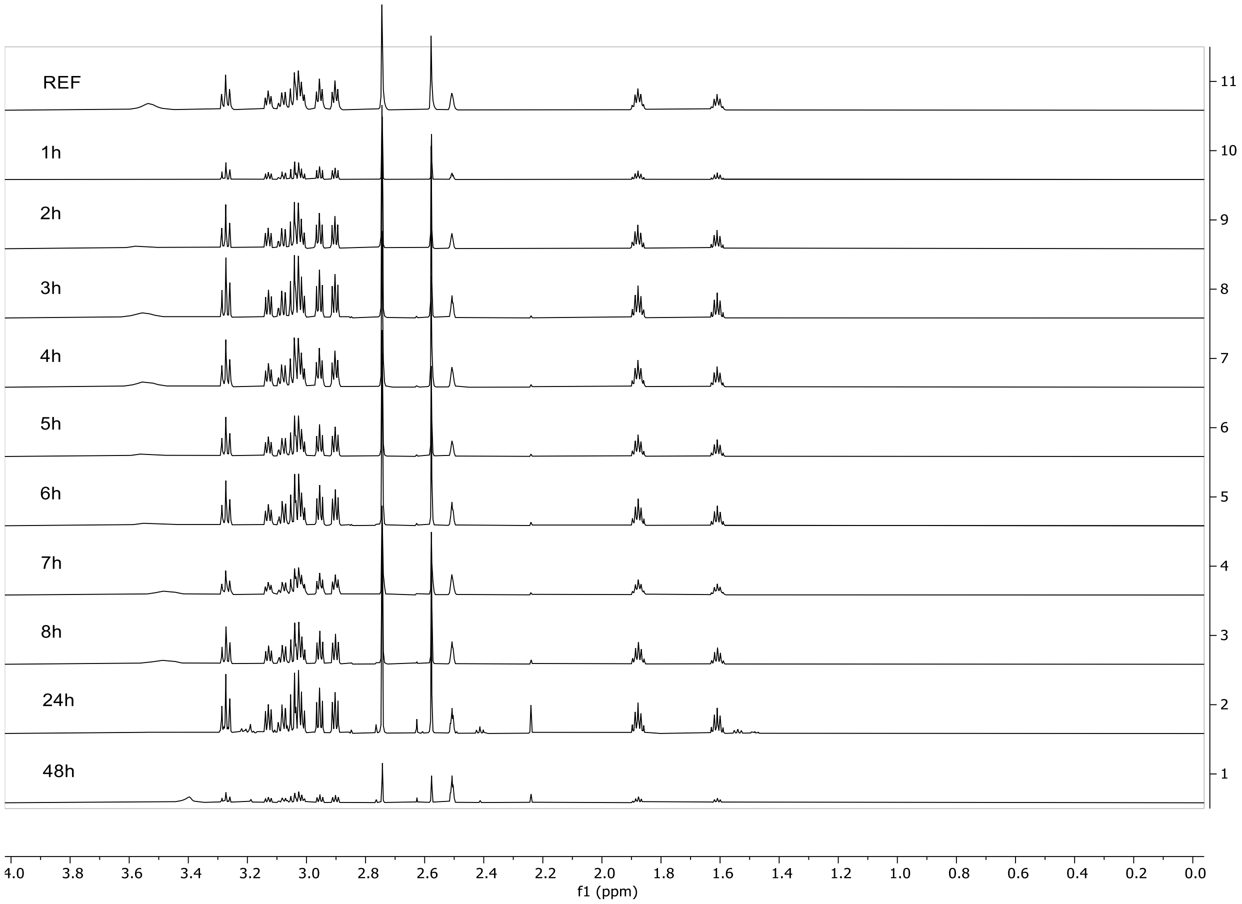

Figure S65 ^{1}H NMR 1 molar equivalent of mTBN and 0.5 molar equivalents of water.

Structure	Time	Integral N-Methyl 1	Integral N-Methyl 2	Integral N Methyl total	Integral HP 1	Integral HP 2	Integral HP Total	% HP
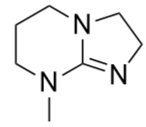	REF	274171000.00	181770000.00	455941000.00	129767.00	1619550.00	1749317.00	0.00
	1h	315067000.00	210657000.00	525724000.00	3739540.00	3461060.00	7200600.00	1.35
	2h	291983000.00	195666000.00	487649000.00	6321530.00	5067400.00	11388930.00	2.28
	3h	238699000.00	161101000.00	399800000.00	9190270.00	5926340.00	15116610.00	3.64
	4h	205631000.00	138475000.00	344106000.00	8998240.00	6219860.00	15218100.00	4.24
	5h	283644000.00	191512000.00	475156000.00	15491300.00	10727900.00	26219200.00	5.23
	6h	196895000.00	132209000.00	329104000.00	12935500.00	9070740.00	22006240.00	6.27
	7h	218589000.00	148718000.00	367307000.00	17576600.00	12153300.00	29729900.00	7.49
	8h	260152000.00	177942000.00	438094000.00	24233400.00	16371800.00	40605200.00	8.48
	24h	201303000.00	144169000.00	345472000.00	63328200.00	45229100.00	108557300.00	23.91
	48h	92316800.00	68213900.00	160530700.00	49917400.00	30050600.00	79968000.00	33.25

Table S66 1 molar equivalent of mTBN and 1 molar equivalent of water.

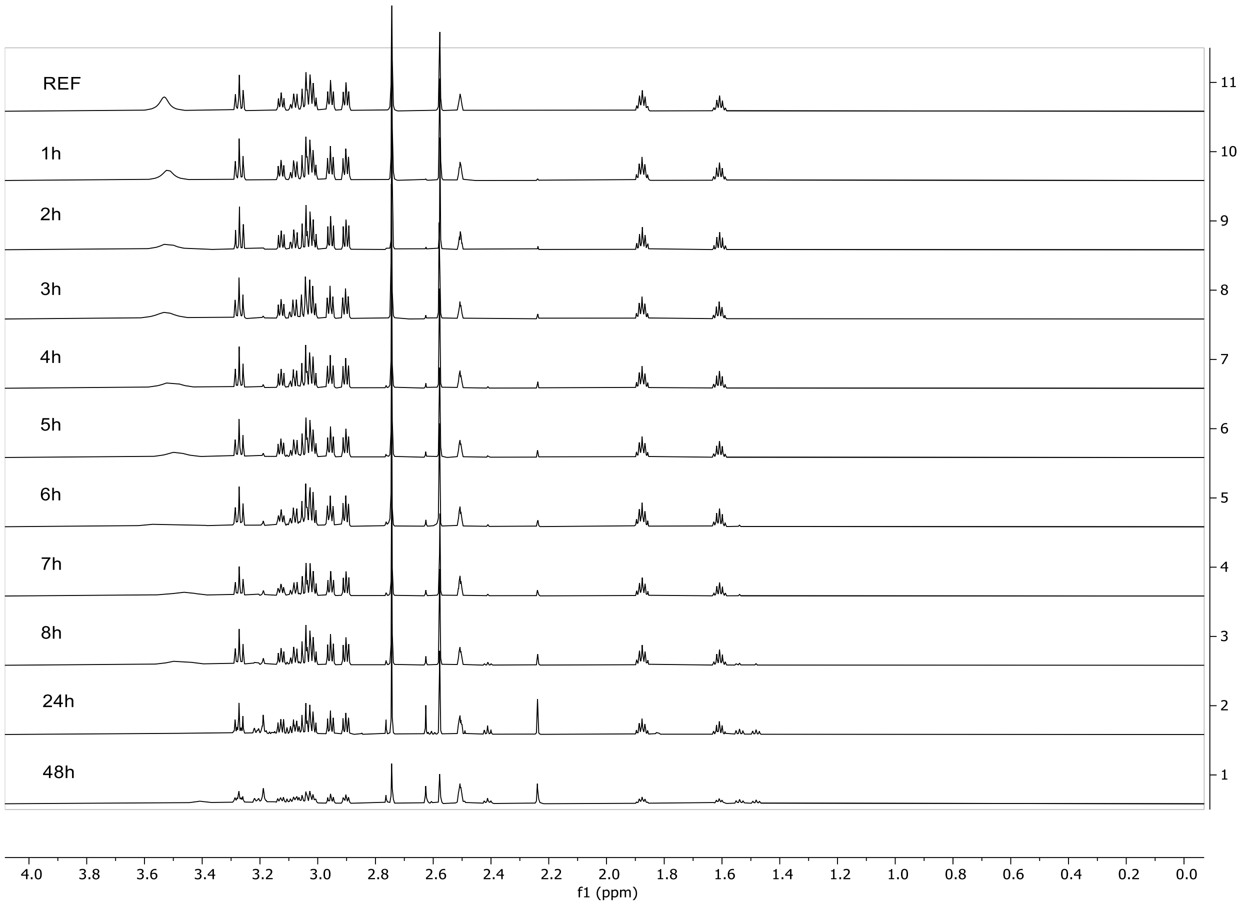

Figure S66 ^{1}H NMR 1 molar equivalent of mTBN and 0.5 molar equivalents of water.

Structure	HT_21	Integral N-Methyl 1	Integral N-Methyl 2	Integral N Methyl total	Integral HP 1	Integral HP 2	Integral HP Total	% HP
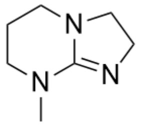	REF	137447000.00	101702000.00	239149000.00	130896.00	512611.00	643507.00	0.00
	1h	103803000.00	109633000.00	213436000.00	88630200.00	16830800.00	105461000.00	33.07
	2h	53692600.00	72153100.00	125845700.00	91262600.00	20134600.00	111397200.00	46.95
	3h	46352600.00	86280500.00	132633100.00	146363000.00	33586200.00	179949200.00	57.57
	4h	7882830.00	33269500.00	41152330.00	56006000.00	20942000.00	76948000.00	65.15
	5h	21653800.00	53793400.00	75447200.00	116365000.00	29775700.00	146140700.00	65.95
	6h	13787100.00	47165300.00	60952400.00	119044000.00	32524400.00	151568400.00	71.32
	7h	3069770.00	24563900.00	27633670.00	81868800.00	29166300.00	111035100.00	80.07
	8h	5801680.00	31334000.00	37135680.00	112207000.00	37744700.00	149951700.00	80.15
	24h	770444.00	28201500.00	28971944.00	223388000.00	93140000.00	316528000.00	91.61
	48h	65510.60	831274.00	896784.60	57911100.00	25160100.00	83071200.00	98.93

Table S67 1 molar equivalent of mTBN and 5 molar equivalents of water.

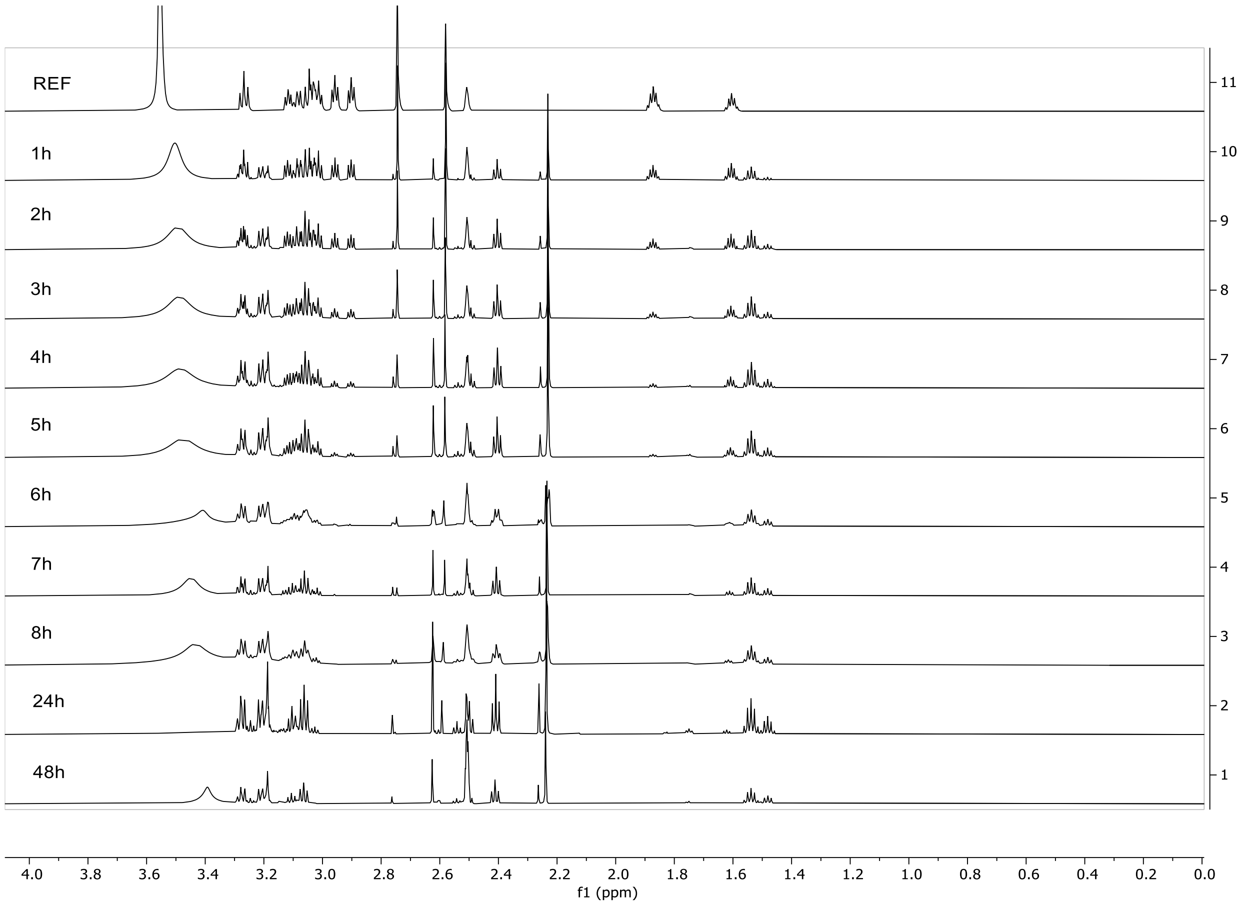

Figure S67 ^{1}H NMR 1 molar equivalent of mTBN and 5 molar equivalents of water.

Structure	Time	Integral N-Methyl 1	Integral N-Methyl 2	Integral N Methyl total	Integral HP 1	Integral HP 2	Integral HP Total	% HP
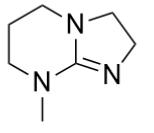	REF	69744700.00	32482300.00	102227000.00	277544.00	1021180.00	1298724.00	0.00
	1h	49150000.00	23760600.00	72910600.00	79668200.00	21413300.00	101081500.00	58.10
	2h	38374300.00	13062900.00	51437200.00	100278000.00	30951800.00	131229800.00	71.84
	3h	28816900.00	6446250.00	35263150.00	110950000.00	38198000.00	149148000.00	80.88
	4h	25939600.00	2309950.00	28249550.00	147285000.00	56498000.00	203783000.00	87.83
	5h	5710880.00	1575720.00	7286600.00	112341000.00	46091100.00	158432100.00	95.60
	6h	5866360.00	3192210.00	9058570.00	143775000.00	69839300.00	213614300.00	95.93
	7h	4326910.00	1391270.00	5718180.00	224374000.00	104998000.00	329372000.00	98.29
	8h	2304490.00	1082220.00	3386710.00	99774800.00	48548300.00	148323100.00	97.77
	24h	5488030.00	1701960.00	7189990.00	136252000.00	65773200.00	202025200.00	96.56
	48h	5080660.00	1391270.00	6471930.00	227558000.00	104610000.00	332168000.00	98.09

Table S68 1 molar equivalent of mTBN and 10 molar equivalents of water.

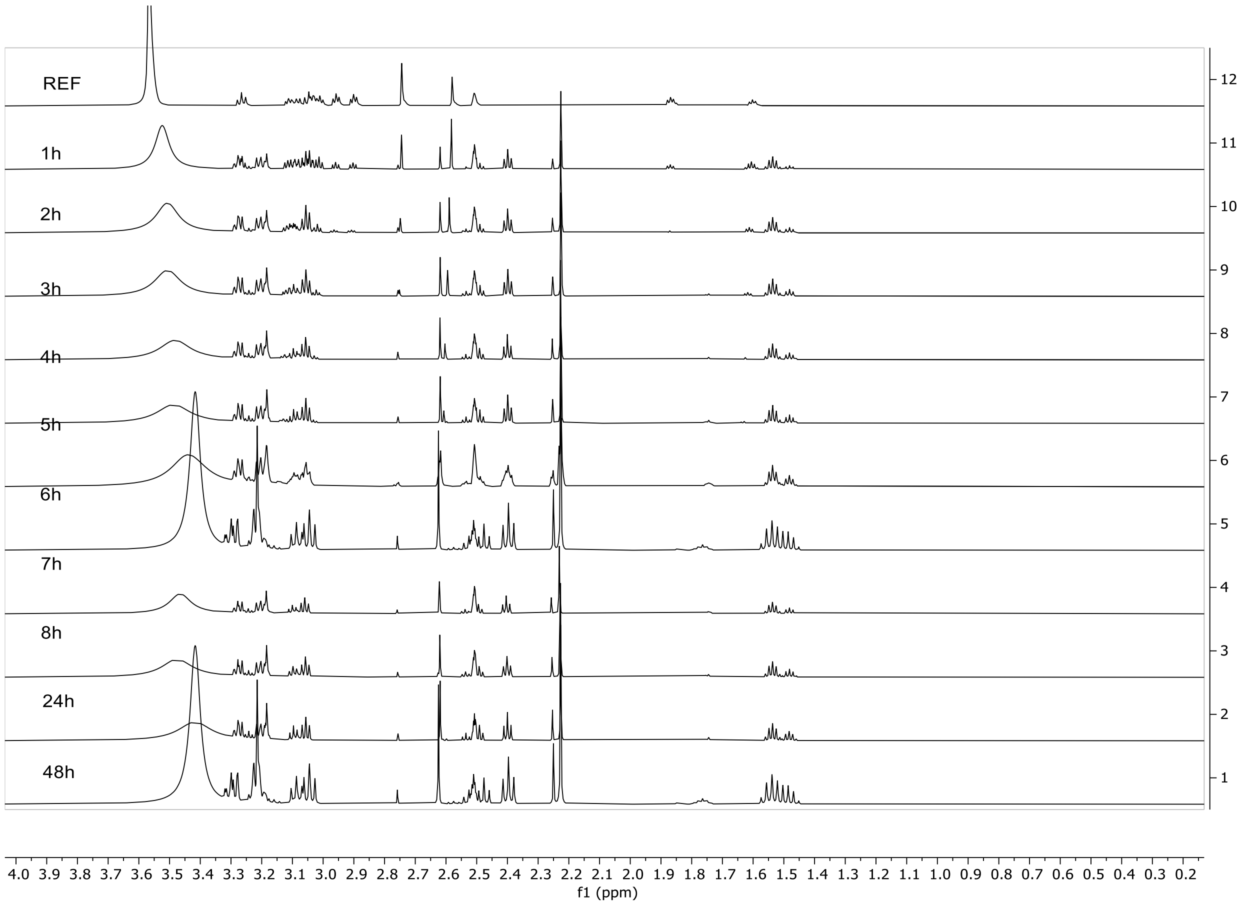

Figure S68 ^{1}H NMR 1 molar equivalent of mTBN and 10 molar equivalents of water.

b. mTBN + water over 12 days at 70°C.

Structure	Time	Integral N-Methyl 1	Integral N-Methyl 2	Integral N Methyl total	Integral HP 1	Integral HP 2	Integral HP Total	% HP
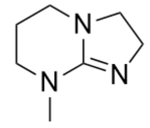	3 days	42122700.00	30666200.00	72788900.00	4485920.00	3247540.00	7733460.00	0.00
	6 days	51387400.00	40088700.00	91476100.00	12080100.00	7988440.00	20068540.00	17.99
	9 days	36534600.00	30708400.00	67243000.00	13176000.00	8689920.00	21865920.00	24.54
	12 days	35435900.00	31295100.00	66731000.00	15970600.00	10529300.00	26499900.00	28.42

Table S69 1 molar equivalent of mTBN and 1 molar equivalents of water.

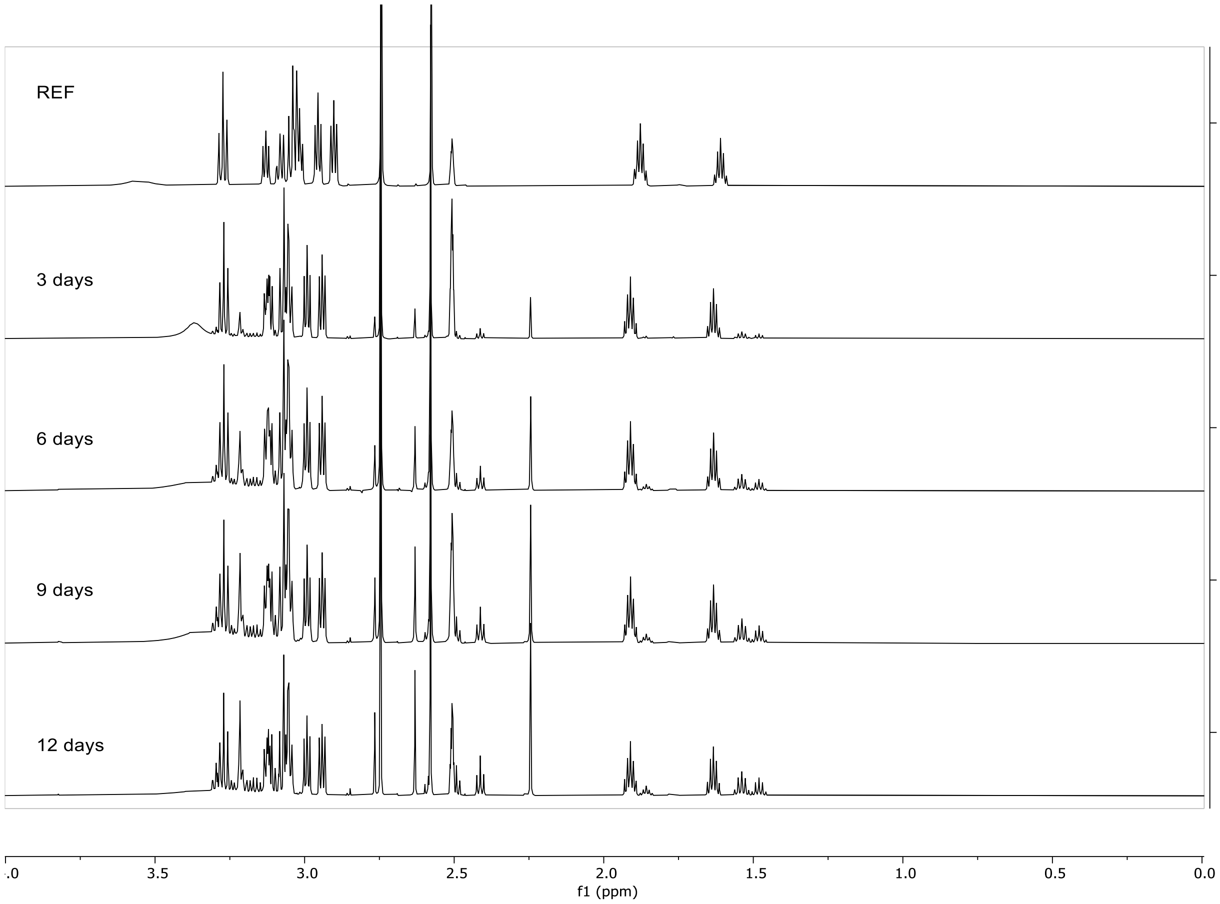

Figure S69 ^{1}H NMR 1 molar equivalent of mTBN and 1 molar equivalents of water.

Structure	Time	Integral N-Methyl 1	Integral N-Methyl 2	Integral N Methyl total	Integral HP 1	Integral HP 2	Integral HP Total	% HP
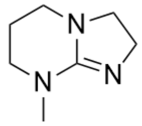	3 days	8964480.00	1150710.00	10115190.00	49062000.00	16027000.00	65089000.00	0.00
	6 days	4074290.00	377125.00	4451415.00	51339100.00	18715700.00	70054800.00	94.03
	9 days	2134300.00	114464.00	2248764.00	49210300.00	18962200.00	68172500.00	96.81
	12 days	588394.00	267989.00	856383.00	59995400.00	24959200.00	84954600.00	99.00

Table S70 1 molar equivalent of mTBN and 5 molar equivalents of water.

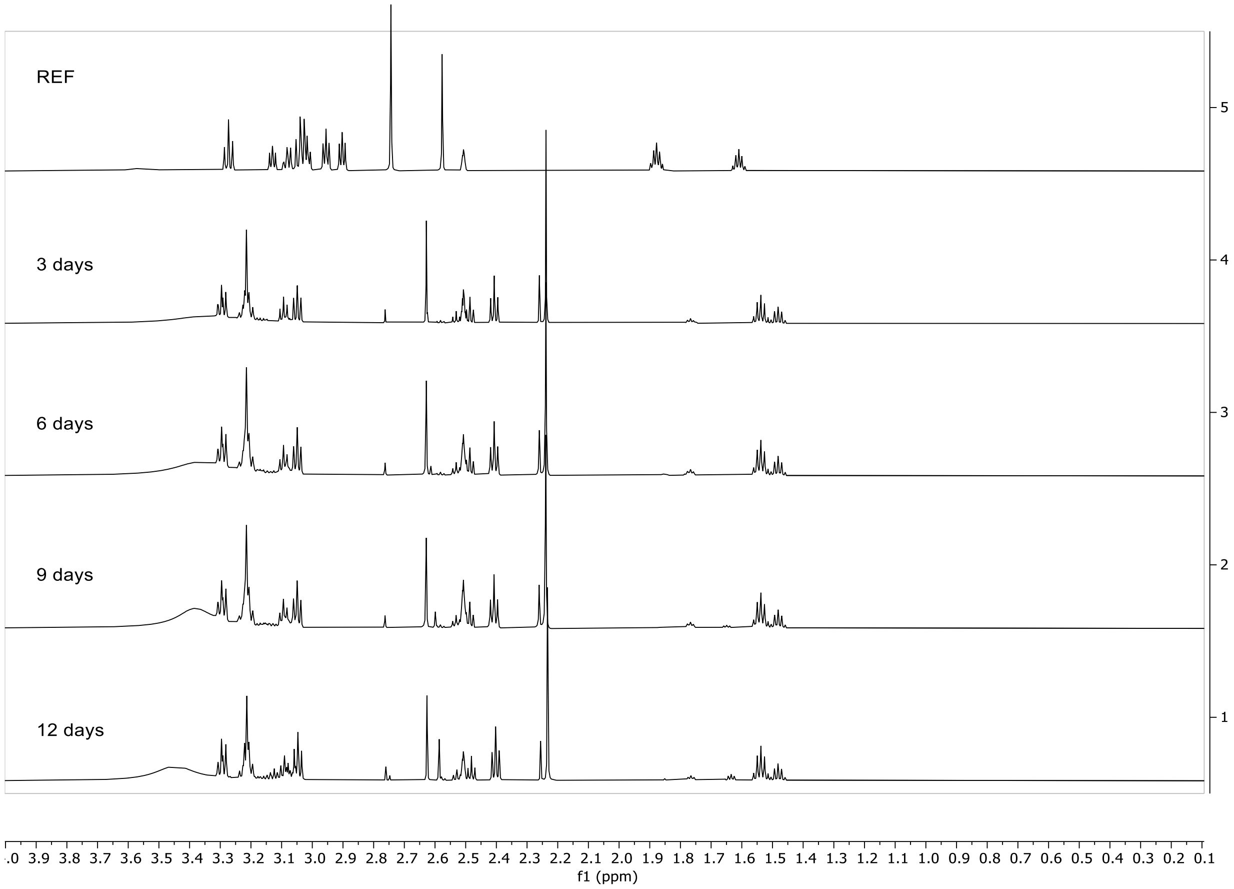

Figure S70 ^{1}H NMR 1 molar equivalent of mTBN and 5 molar equivalents of water.

Structure	Time	Integral N-Methyl 1	Integral N-Methyl 2	Integral N Methyl total	Integral HP 1	Integral HP 2	Integral HP Total	% HP
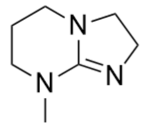	3 days	0.00	0.00	0.00	14865900.00	37827400.00	52693300.00	0.00
	6 days	0.00	0.00	0.00	14774800.00	38572800.00	53347600.00	100.00
	9 days	0.00	0.00	0.00	11135400.00	28766100.00	39901500.00	100.00
	12 days	0.00	0.00	0.00	14762000.00	37944700.00	52706700.00	100.00

Table S71 1 molar equivalent of mTBN and 10 molar equivalents of water.

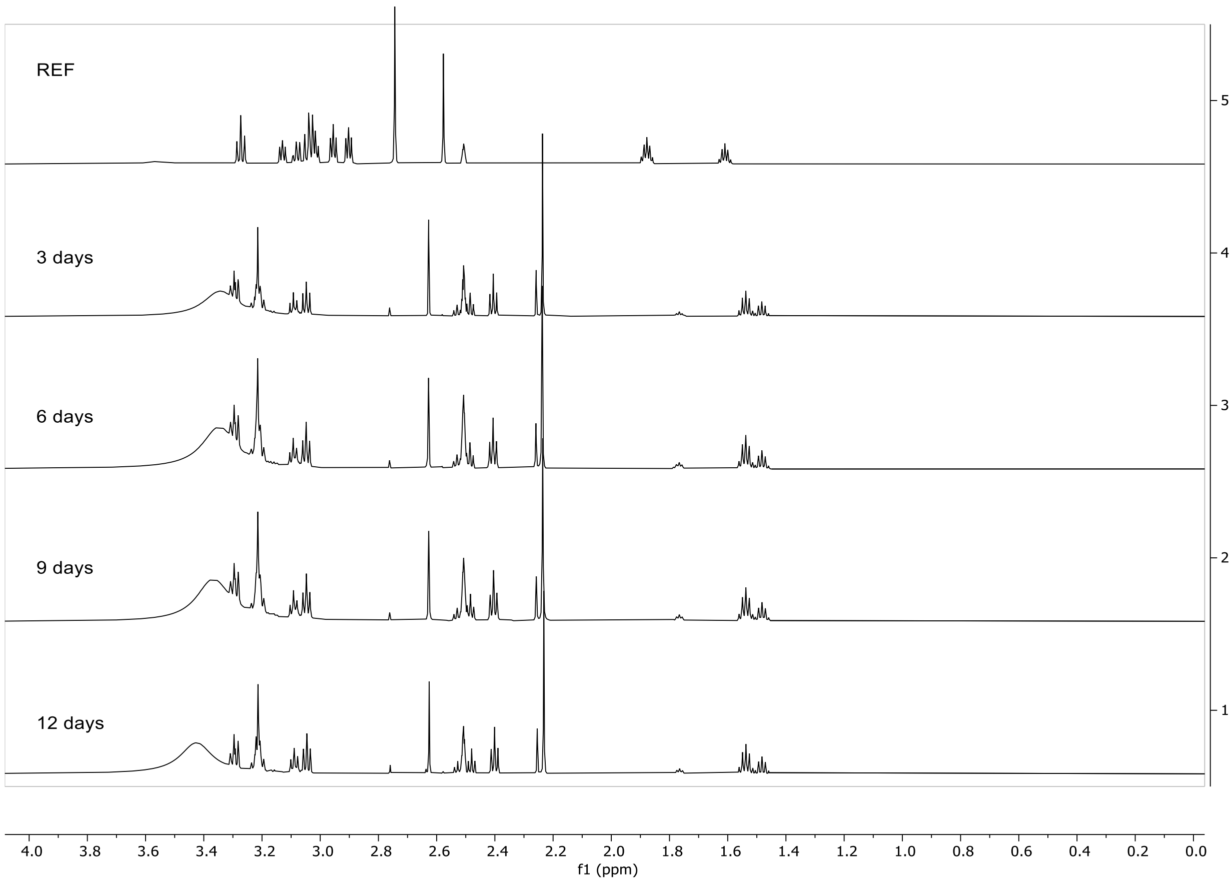

Figure S71 ^{1}H NMR 1 molar equivalent of mTBN and 10 molar equivalents of water.

c. *mTBN + water over 12 days at 90°C.*

Structure	Time	integral N-Methyl 1	integral N-Methyl 2	integral N Methyl total	integral HP 1	integral HP 2	integral HP Total	% HP
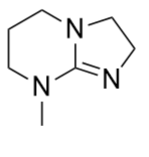	3 days	23842200.00	29305100.00	53147300.00	20631500.00	15517900.00	36149400.00	0.00
	6 days	19722700.00	21872200.00	41594900.00	31151000.00	23394600.00	54545600.00	56.74
	9 days	15981600.00	16093100.00	32074700.00	33268700.00	25196300.00	58465000.00	64.57
	12 days	14027900.00	12235800.00	26263700.00	33432900.00	26403900.00	59836800.00	69.50

Table S72 1 molar equivalent of mTBN and 1 molar equivalents of water.

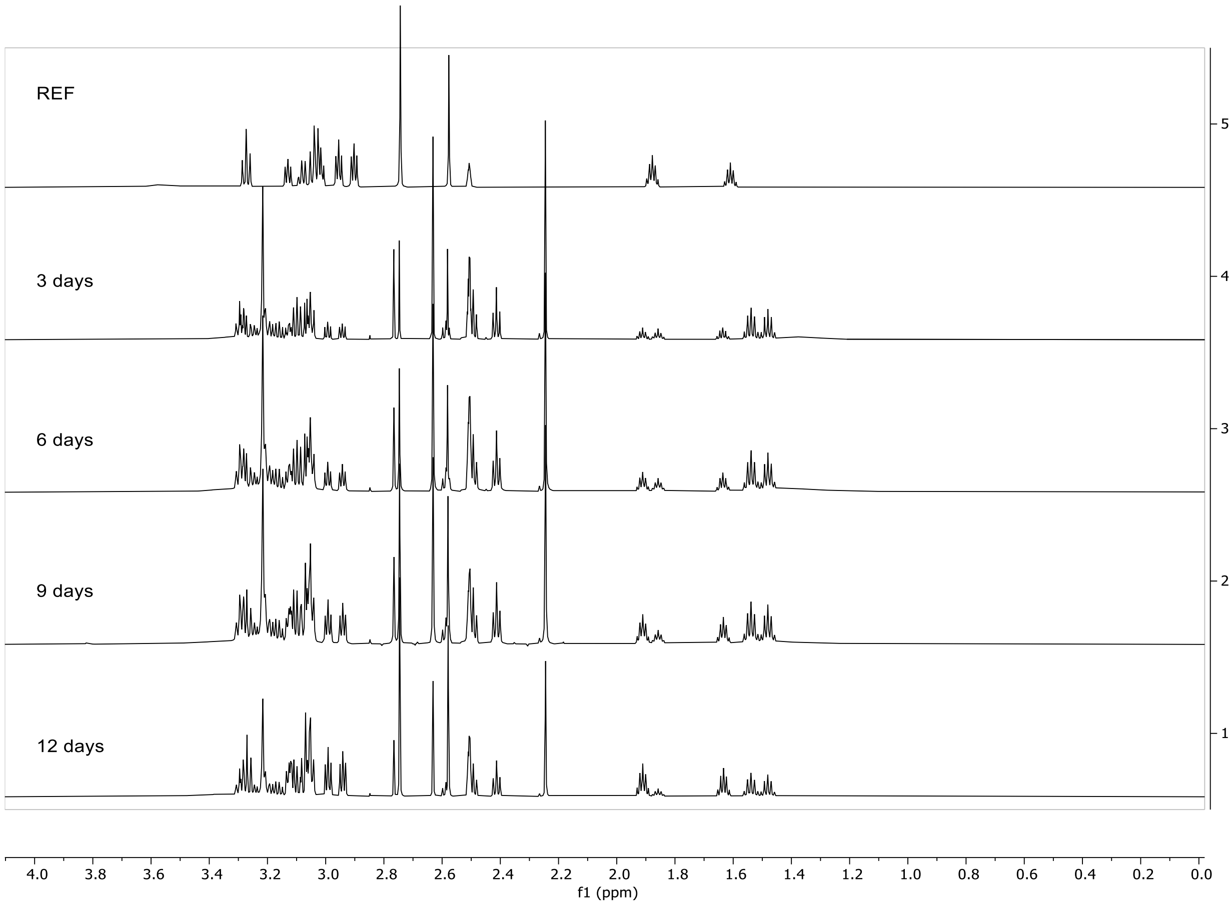

Figure S72 ^{1}H NMR 1 molar equivalent of mTBN and 1 molar equivalents of water.

Structure	HT_67	Integral N-Methyl 1	Integral N-Methyl 2	Integral N Methyl total	Integral HP 1	Integral HP 2	Integral HP Total	% HP
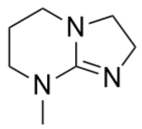	3 days	0.00	0.00	0.00	61522200.00	26043300.00	87565500.00	0.00
	6 days	0.00	0.00	0.00	47064700.00	19365500.00	66430200.00	100.00
	9 days	0.00	0.00	0.00	57465100.00	24332200.00	81797300.00	100.00
	12 days	0.00	0.00	0.00	50824700.00	21528700.00	72353400.00	100.00

Table S73 1 molar equivalent of mTBN and 5 molar equivalents of water.

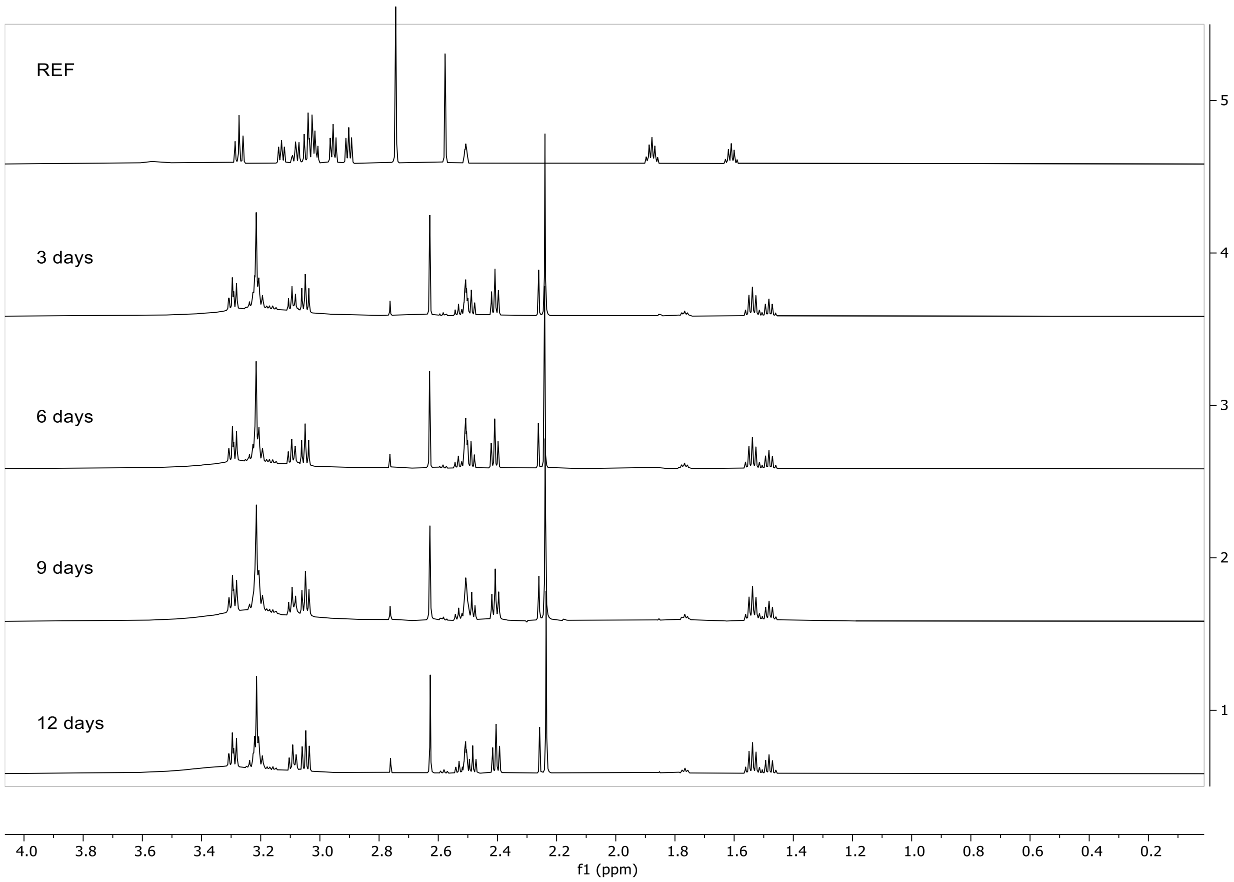

Figure S73 ^{1}H NMR 1 molar equivalent of mTBN and 5 molar equivalents of water.

Structure	Time	Integral N-Methyl 1	Integral N-Methyl 2	Integral N Methyl total	Integral HP 1	Integral HP 2	Integral HP Total	% HP
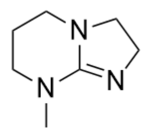	3 days	0.00	0.00	0.00	41235200.00	15406000.00	56641200.00	0.00
	6 days	0.00	0.00	0.00	31242600.00	12217800.00	43460400.00	100.00
	9 days	0.00	0.00	0.00	38728600.00	15024800.00	53753400.00	100.00
	12 days	0.00	0.00	0.00	38544000.00	14651800.00	53195800.00	100.00

Table S74 1 molar equivalent of mTBN and 10 molar equivalents of water.

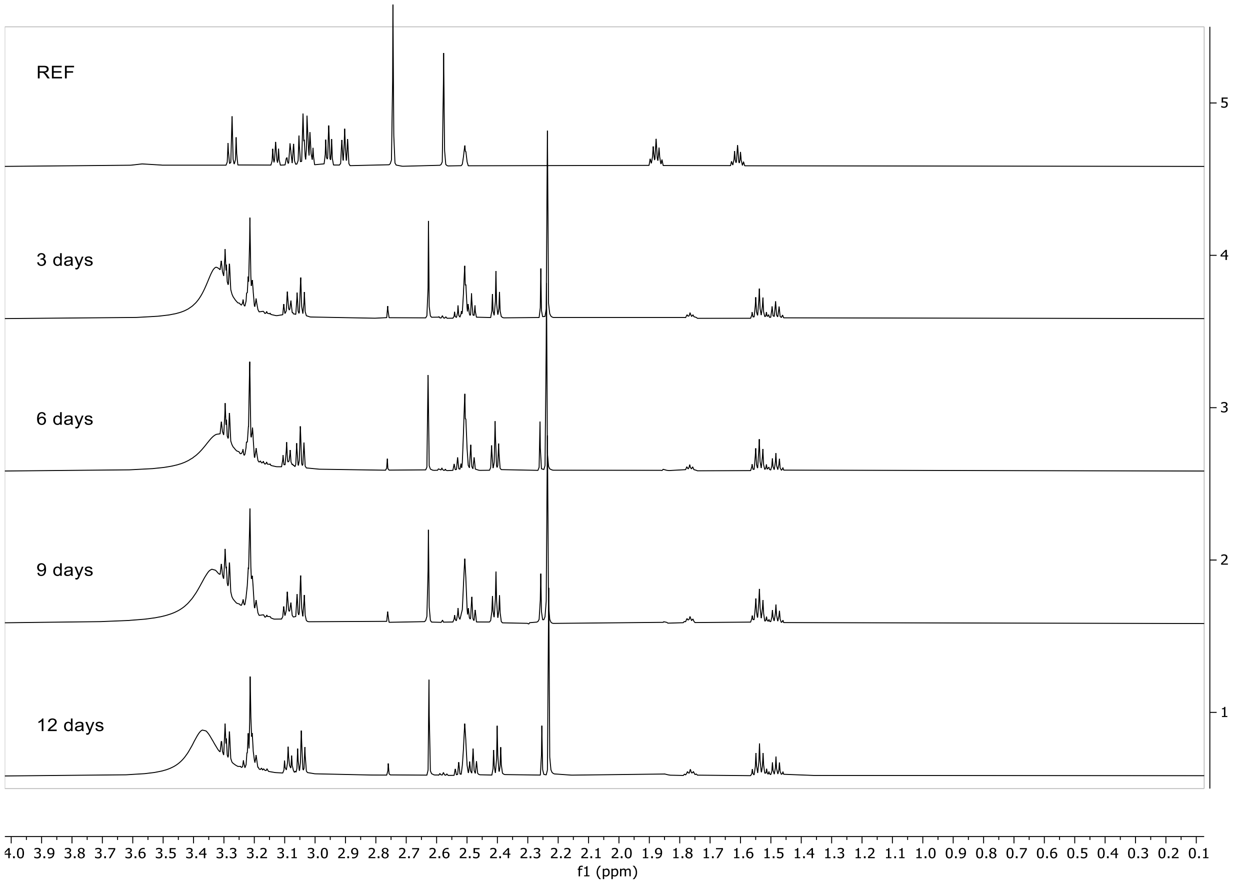

Figure S74 ^{1}H NMR 1 molar equivalent of mTBN and 10 molar equivalents of water.

4. [mTBNH][OAc] + water

a. [mTBNH][OAc] + water over 48h at 90°C.

Structure	Time	integral N-Methyl 1	Integral N-Methyl 2	Integral N Methyl total	Integral HP 1	Integral HP 2	Integral HP Total	% HP
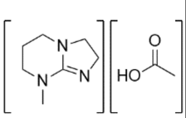	REF	33666500000000.00	22630400000000.00	56296900000000.00	23651300000.00	35772400000.00	59423700000.00	0.11
	1h	34431200000000.00	22887300000000.00	57318500000000.00	75204100000.00	47463300000.00	122667400000.00	0.21
	2h	32756500000000.00	21791500000000.00	54548000000000.00	148240000000.00	55030600000.00	203270600000.00	0.37
	3h	42487600000000.00	28277300000000.00	70764900000000.00	301394000000.00	92590500000.00	393984500000.00	0.55
	4h	58989300000000.00	37021700000000.00	96011000000000.00	560689000000.00	129331000000.00	690020000000.00	0.71
	5h	54927000000000.00	34113800000000.00	89040800000000.00	655415000000.00	143193000000.00	798608000000.00	0.89
	6h	54572200000000.00	34895800000000.00	89468000000000.00	770693000000.00	167399000000.00	938092000000.00	1.04
	7h	40057700000000.00	26400000000000.00	66457700000000.00	673493000000.00	143192000000.00	816685000000.00	1.21
	8h	14943200000000.00	10023500000000.00	24966700000000.00	270284000000.00	57500100000.00	327784100000.00	1.30
	24h	35248900000000.00	24718900000000.00	59967800000000.00	2087040000000.00	453534000000.00	2540574000000.00	4.06
	48h	40818400000000.00	31047300000000.00	71865700000000.00	5017920000000.00	1058190000000.00	6076110000000.00	7.80

Table S75 1 molar equivalent of [mTBNH][OAc] and 0.2 molar equivalents of water.

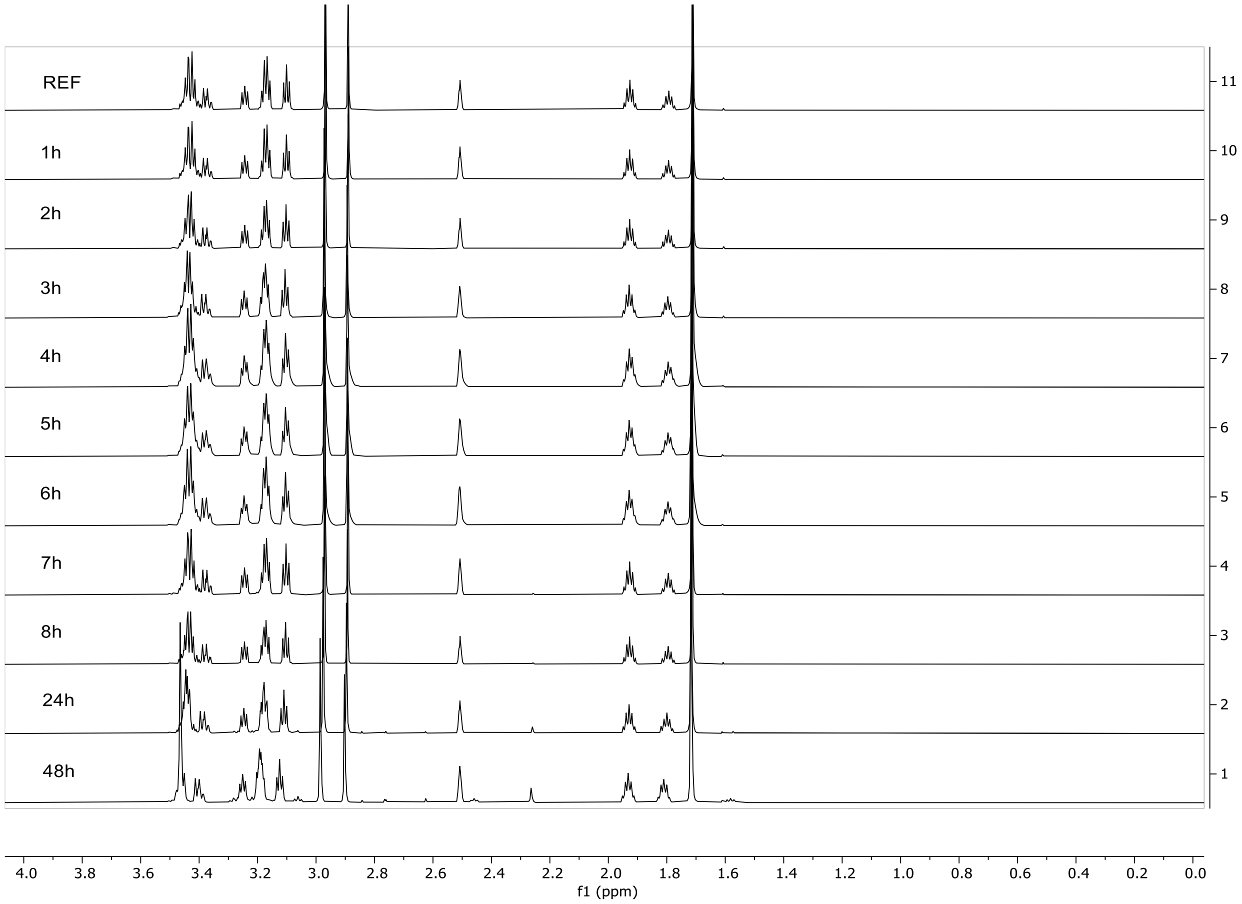

Figure S75 ^{1}H NMR 1 molar equivalent of [mTBNH][OAc] and 0.2 molar equivalents of water.

Structure	Time	Integral N-Methyl	Integral N-Methyl	Integral N Methyl total	Integral HP 1	Integral HP 2	Integral HP Total	% HP
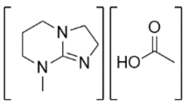	REF	213554000.00	142810000.00	356364000.00	143008.00	175407.00	318415.00	0.09
	1h	223300000.00	149067000.00	372367000.00	558485.00	284023.00	842508.00	0.23
	2h	213868000.00	142280000.00	356148000.00	1185810.00	478905.00	1664715.00	0.47
	3h	213490000.00	140803000.00	354293000.00	1811730.00	528672.00	2340402.00	0.66
	4h	207204000.00	135660000.00	342864000.00	2564280.00	788882.00	3353162.00	0.97
	5h	181008000.00	121659000.00	302667000.00	2911130.00	836769.00	3747899.00	1.22
	6h	214612000.00	144045000.00	358657000.00	4035350.00	1093630.00	5128980.00	1.41
	7h	223478000.00	151878000.00	375356000.00	5110500.00	1329430.00	6439930.00	1.69
	8h	197162000.00	134645000.00	331807000.00	4954200.00	1287290.00	6241490.00	1.85
	24h	122861000.00	89042900.00	211903900.00	9928710.00	1919070.00	11847780.00	5.30
	48h	551909000.00	444737000.00	996646000.00	101972000.00	19104700.00	121076700.00	10.83

Table S76 1 molar equivalent of [mTBNH][OAc] and 0,5 molar equivalents of water.

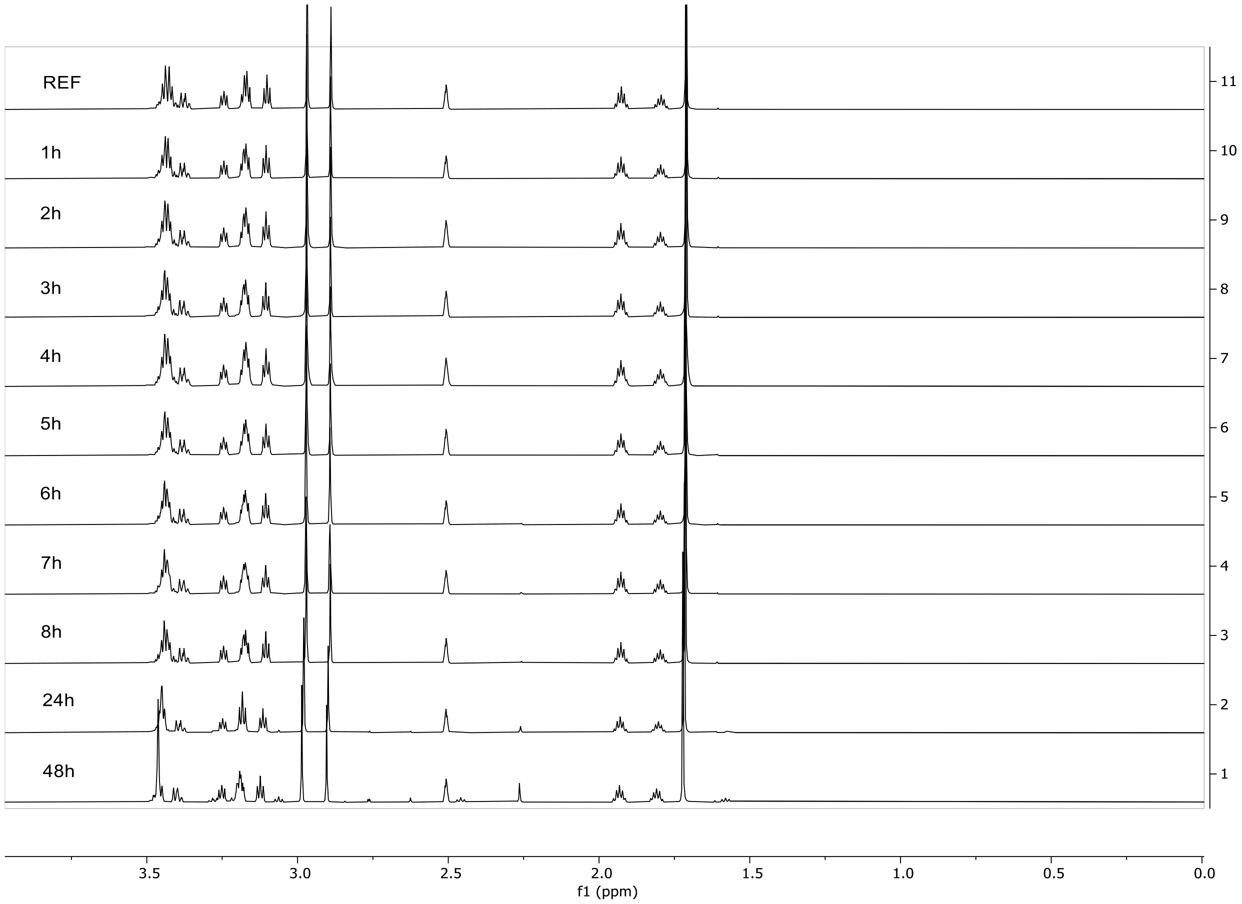

Figure S76 ^1H NMR 1 molar equivalent of [mTBNH][OAc] and 0,5 molar equivalents of water.

Structure	Time	Integral N-Methyl	Integral N-Methyl	Integral N Methyl total	Integral HP 1	Integral HP 2	Integral HP Total	% HP
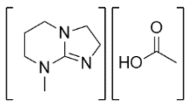	REF	218724000.00	147430000.00	366154000.00	0.00	0.00	0.00	0.00
	1h	210968000.00	144586000.00	355554000.00	327610.00	315059.00	642669.00	0.18
	2h	211877000.00	145307000.00	357184000.00	1008150.00	477596.00	1485746.00	0.41
	3h	199022000.00	136587000.00	335609000.00	1548820.00	598917.00	2147737.00	0.64
	4h	214490000.00	149088000.00	363578000.00	2764430.00	816421.00	3580851.00	0.98
	5h	225148000.00	154708000.00	379856000.00	3528860.00	973696.00	4502556.00	1.17
	6h	185807000.00	125472000.00	311279000.00	3170250.00	877822.00	4048072.00	1.28
	7h	177393000.00	121825000.00	299218000.00	3244530.00	933490.00	4178020.00	1.38
	8h	198386000.00	136337000.00	334723000.00	4460040.00	1246870.00	5706910.00	1.68
	24h	182436000.00	137243000.00	319679000.00	17432100.00	3895720.00	21327820.00	6.25
	48h	151916000.00	125530000.00	277446000.00	32571500.00	6553030.00	39124530.00	12.36

Table S77 1 molar equivalent of [mTBNH][OAc] and 1 molar equivalents of water.

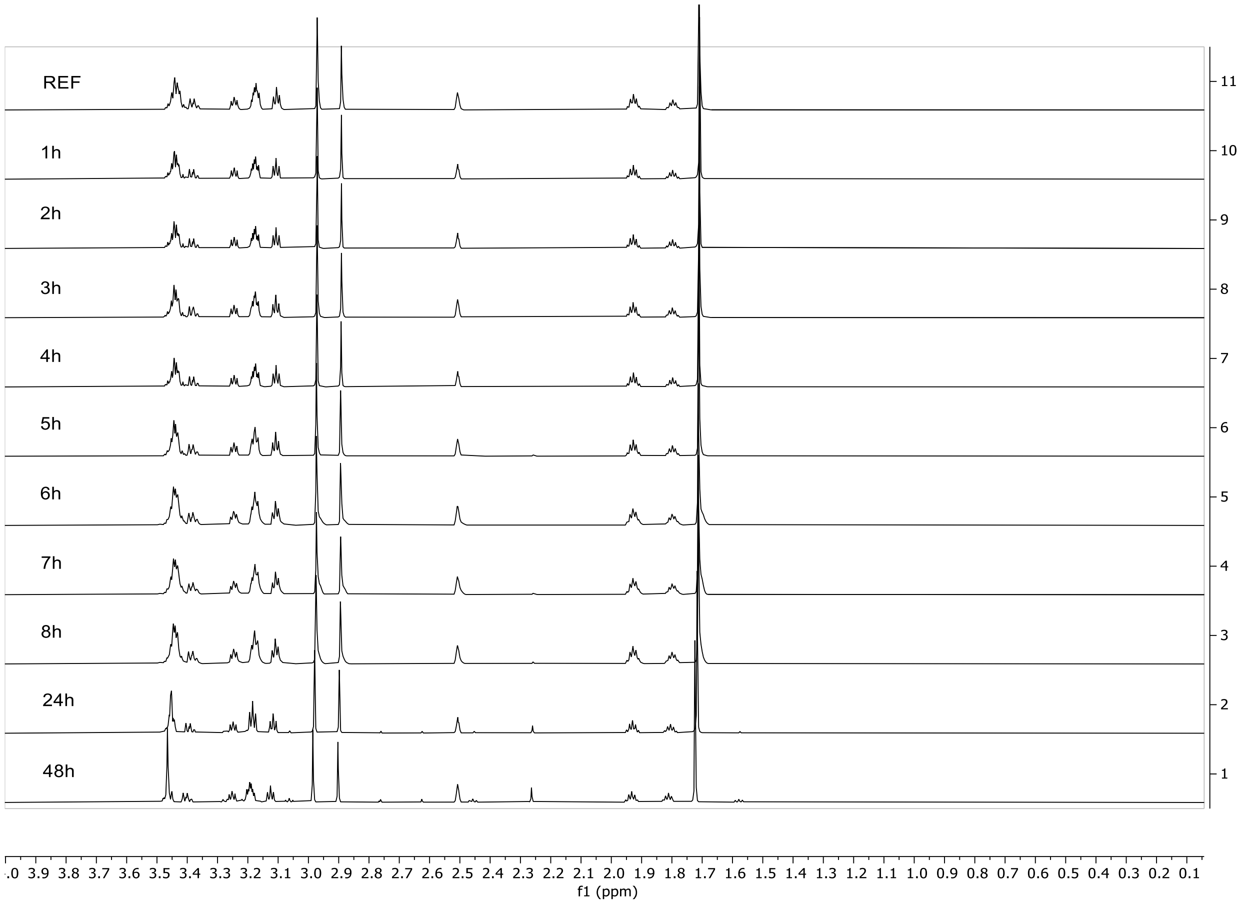

Figure S77 ^{1}H NMR 1 molar equivalent of [mTBNH][OAc] and 1 molar equivalents of water.

Structure	Time	integral N-Methyl	integral N-Methyl	integral N Methyl total	integral HP 1	integral HP 2	integral HP Total	% HP
	REF	163742000.00	111893000.00	275635000.00	40961.10	179526.00	220487.10	
	1h	177484000.00	120743000.00	298227000.00	220661.00	237864.00	458525.00	0.15
	2h	169317000.00	114283000.00	283600000.00	415335.00	279484.00	694819.00	0.24
	3h	152955000.00	101732000.00	254687000.00	647744.00	275147.00	922891.00	0.36
	4h	174700000.00	116986000.00	291686000.00	985405.00	395518.00	1380923.00	0.47
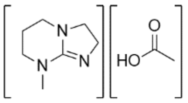	5h	171060000.00	115399000.00	286459000.00	1197990.00	429394.00	1627384.00	0.56
	6h	176529000.00	119765000.00	296294000.00	1403780.00	421767.00	1825547.00	0.61
	7h	138106000.00	95870100.00	233976100.00	1353170.00	438154.00	1791324.00	0.76
	8h	155870000.00	107448000.00	263318000.00	1646190.00	519846.00	2166036.00	0.82
	24h	152868000.00	105868000.00	258736000.00	5172080.00	998419.00	6170499.00	2.33
	48h	148394000.00	106352000.00	254746000.00	11090200.00	2181700.00	13271900.00	4.95

Table S78 1 molar equivalent of [mTBNH][OAc] and 5 molar equivalents of water.

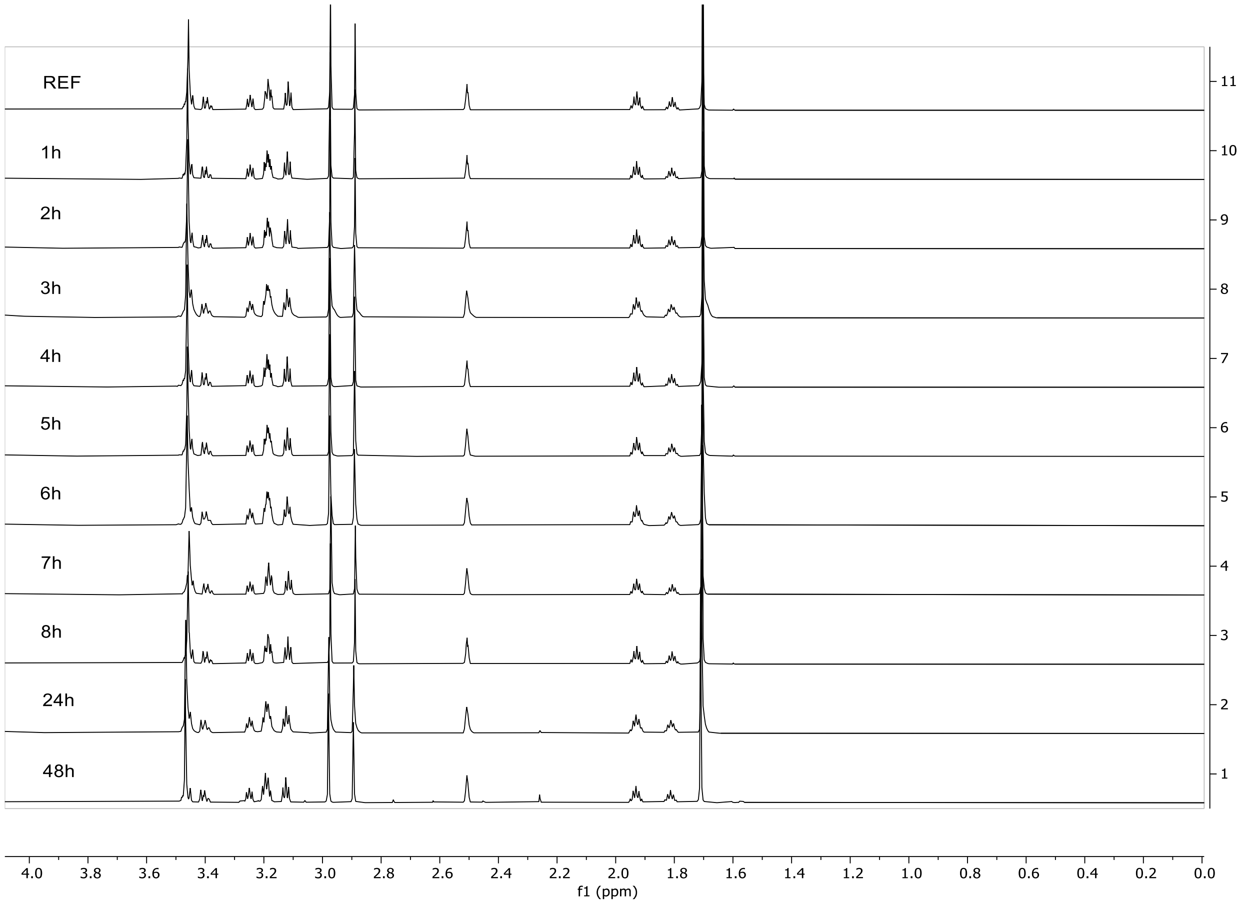

Figure S78 ^{1}H NMR 1 molar equivalent of [mTBNH][OAc] and 5 molar equivalents of water.

Structure	Time	Integral N-Methyl	Integral N-Methyl	Integral N Methyl total	Integral HP 1	Integral HP 2	Integral HP Total	% HP
	REF	127954000.00	85446800.00	213400800.00	79223.20	191353.00	270576.20	
	1h	132023000.00	88060500.00	220083500.00	104384.00	234173.00	338557.00	0.15
	2h	137858000.00	92166500.00	230024500.00	152727.00	234848.00	387575.00	0.17
	3h	121263000.00	80096100.00	201359100.00	231998.00	294935.00	526933.00	0.26
	4h	137695000.00	92140700.00	229835700.00	345971.00	331375.00	677346.00	0.29
	5h	129706000.00	85911300.00	215617300.00	369118.00	326471.00	695589.00	0.32
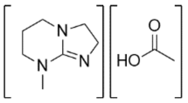	6h	141257000.00	94705000.00	235962000.00	462608.00	379181.00	841789.00	0.36
	7h	121419000.00	80648600.00	202067600.00	527291.00	480460.00	1007751.00	0.50
	8h	126393000.00	84383600.00	210776600.00	646993.00	529049.00	1176042.00	0.55
	24h	134462000.00	88856500.00	223318500.00	2178680.00	782227.00	2960907.00	1.31
	48h	204864000.00	144396000.00	349260000.00	15149900.00	3358300.00	18508200.00	5.03

Table S79 1 molar equivalent of [mTBNH][OAc] and 10 molar equivalents of water.

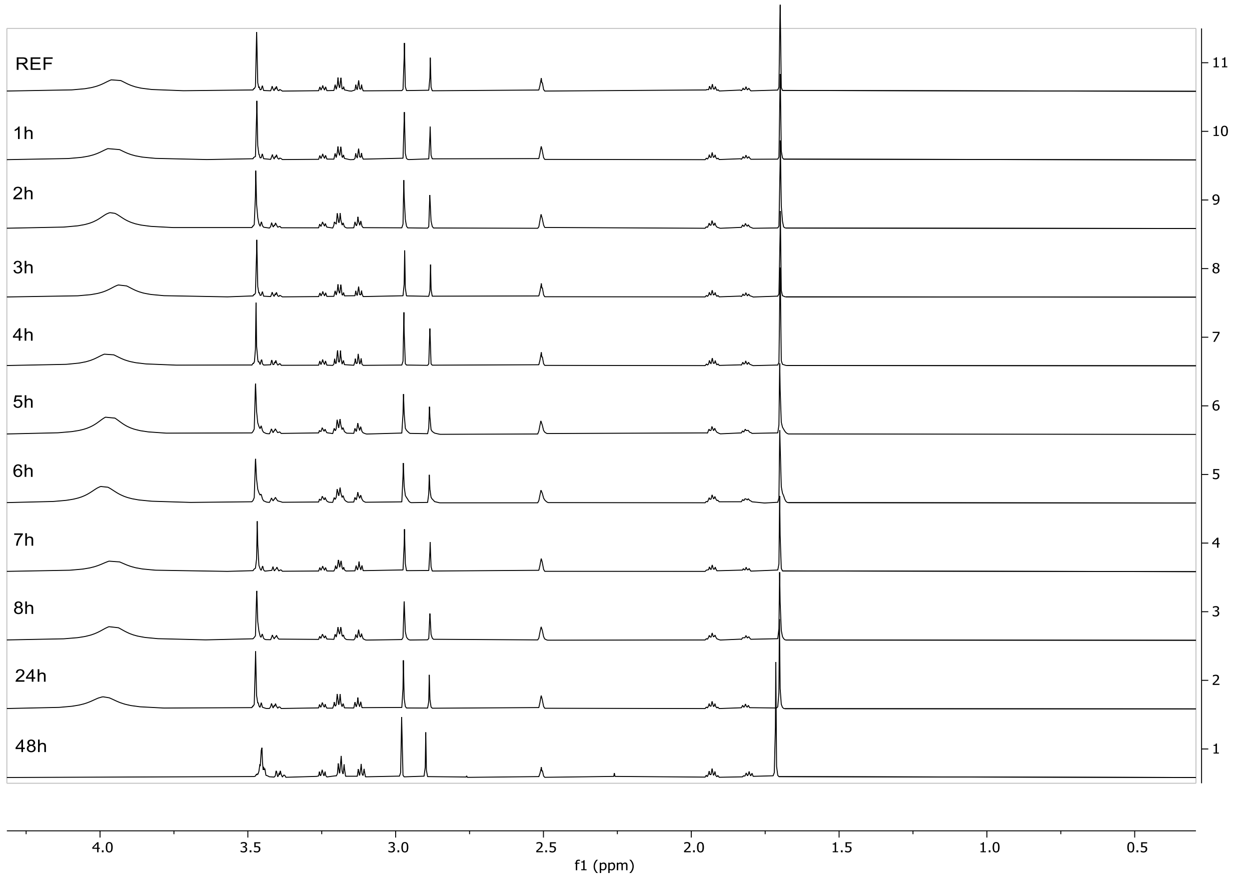

Figure S79 ^{1}H NMR 1 molar equivalent of [mTBNH][OAc] and 10 molar equivalents of water.

b. [mTBNH][OAc] + water at 90°C over 12 days.

Structure	Time	Integral N-Methyl 1	Integral N-Methyl 2	Integral N Methyl total	Integral HP 1	Integral HP 2	Integral HP Total	% HP
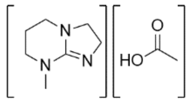	3 days	34001700.00	28664200.00	62665900.00	10204400.00	2904460.00	13108860.00	17.30
	6 days	25639500.00	25201700.00	50841200.00	14294700.00	4630280.00	18924980.00	27.13
	9 days	23818200.00	25662100.00	49480300.00	17872100.00	6828110.00	24700210.00	33.30
	12 days	23519800.00	25227200.00	48747000.00	18814600.00	8341380.00	27155980.00	35.78

Table S80 1 molar equivalent of [mTBNH][OAc] and 1 molar equivalents of water.

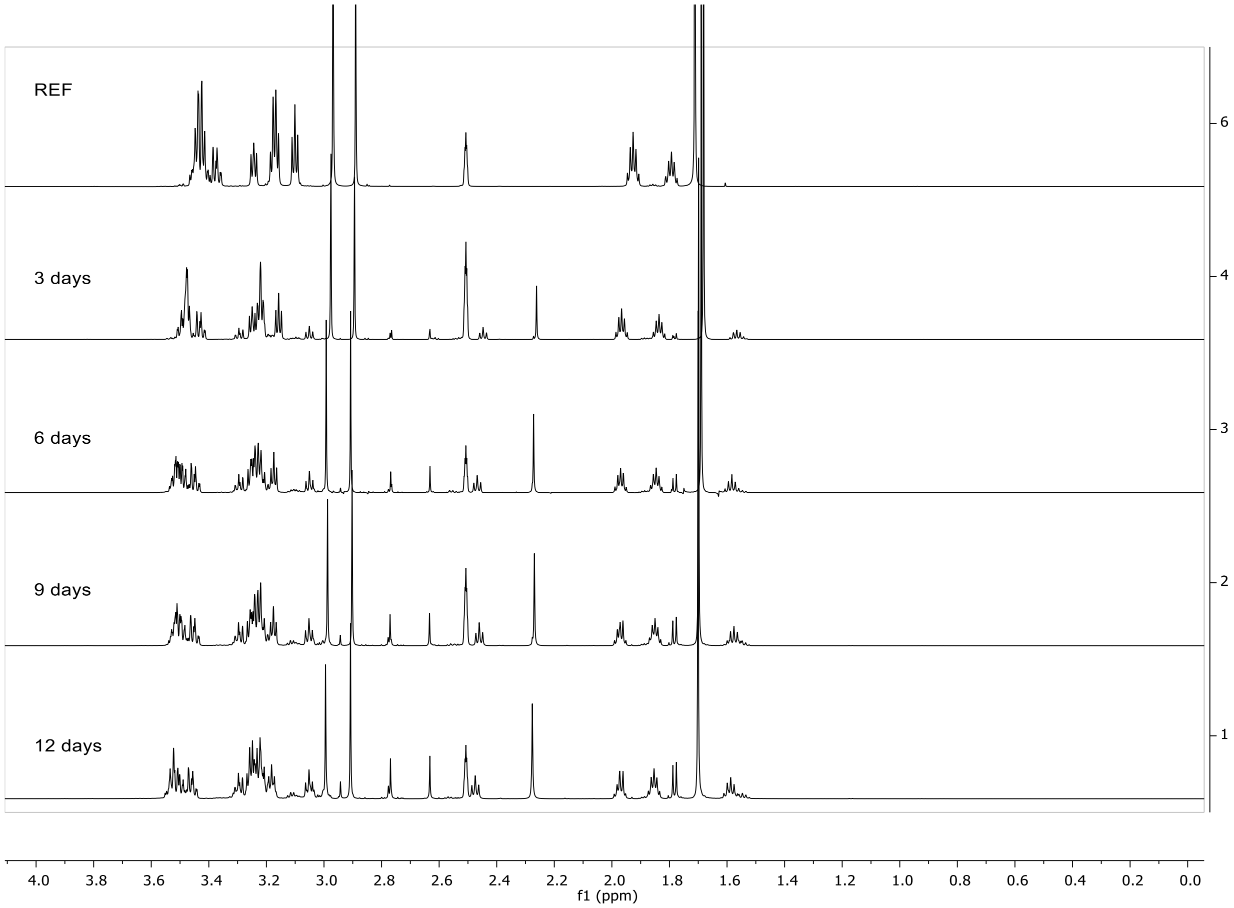

Figure S80 ^{1}H NMR 1 molar equivalent of [mTBNH][OAc] and 1 molar equivalents of water.

Structure	Time	Integral N-Methyl 1	Integral N-Methyl 2	Integral N Methyl total	Integral HP 1	Integral HP 2	Integral HP Total	% HP
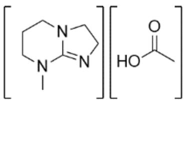	3 days	38420400.00	27596500.00	66016900.00	4182720.00	1441130.00	5623850.00	0.00
	6 days	24514000.00	19230500.00	43744500.00	5397700.00	1785630.00	7183330.00	14.10
	9 days	25897000.00	21121900.00	47018900.00	7891490.00	2580550.00	10472040.00	18.22
	12 days	27752300.00	23631900.00	51384200.00	10403600.00	3502850.00	13906450.00	21.30

Table S81 1 molar equivalent of [mTBNH][OAc] and 5 molar equivalents of water.

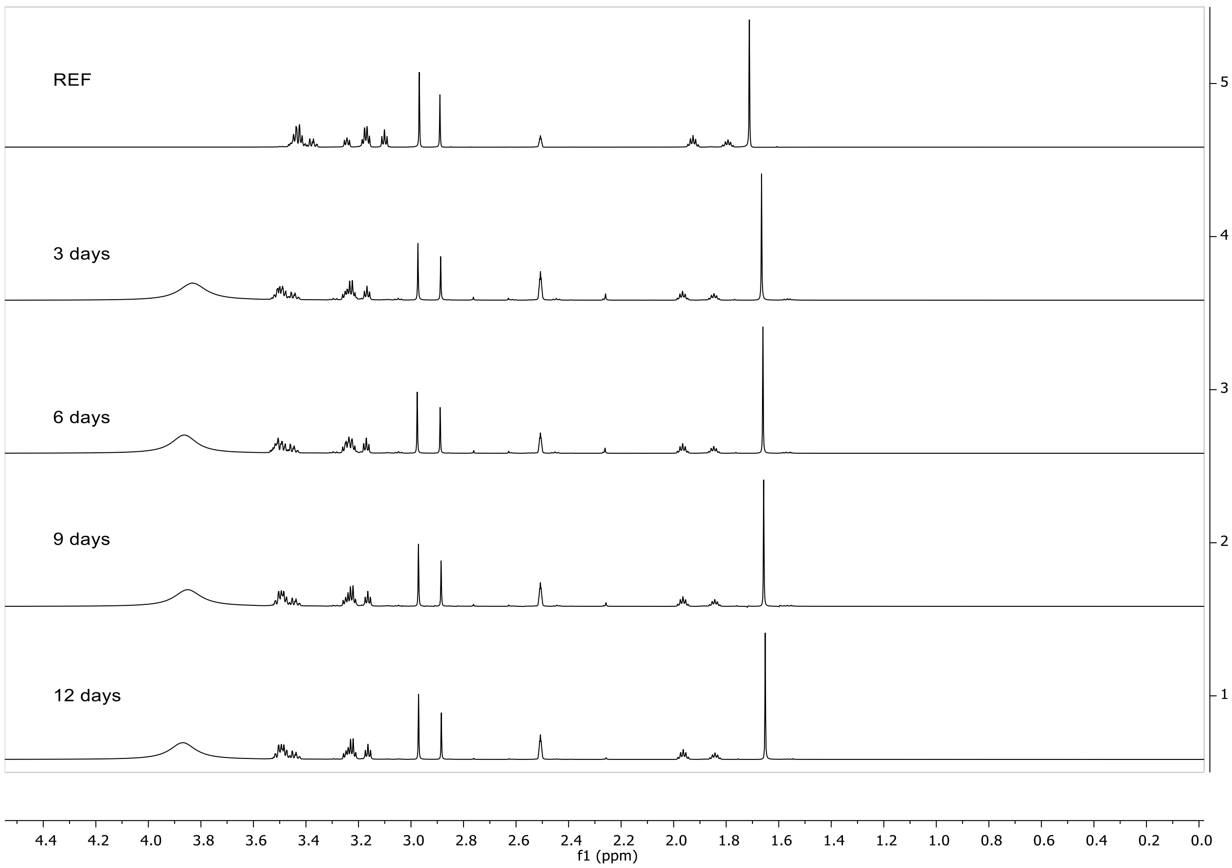

Figure S81 ^{1}H NMR 1 molar equivalent of [mTBNH][OAc] and 5 molar equivalents of water.

Structure	Time	Integral N-Methyl 1	Integral N-Methyl 2	Integral N Methyl total	Integral HP 1	Integral HP 2	Integral HP Total	% HP
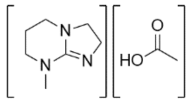	3 days	21957100.00	15203800.00	37160900.00	1327510.00	1001140.00	2328650.00	0.00
	6 days	27629500.00	20076800.00	47706300.00	3029630.00	1999850.00	5029480.00	9.54
	9 days	23755200.00	17248600.00	41003800.00	3295790.00	1697480.00	4993270.00	10.86
	12 days	25644400.00	19079500.00	44723900.00	4488240.00	2445020.00	6933260.00	13.42

Table S82 1 molar equivalent of [mTBNH][OAc] and 10 molar equivalents of water.

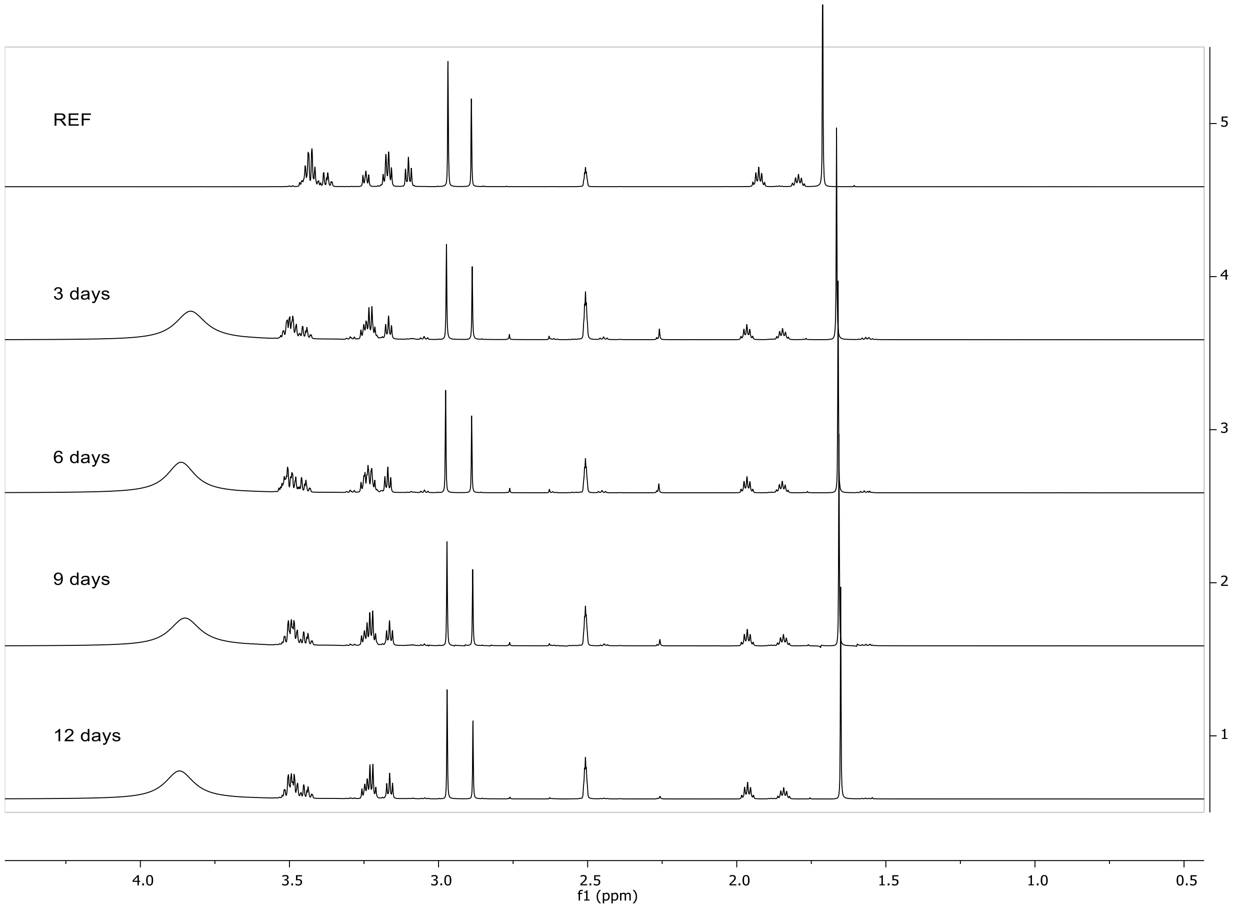

Figure S82 ^{1}H NMR 1 molar equivalent of [mTBNH][OAc] and 10 molar equivalents of water.

c. [mTBNH][OAc] + water at 70°C over 12 days.

Structure	Time	Integral N-Methyl 1	Integral N-Methyl 2	Integral N Methyl total	Integral HP 1	Integral HP 2	Integral HP Total	% HP
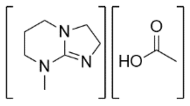	3 days	54770200.00	38247000.00	93017200.00	892626.00	747923.00	1640549.00	1.73
	6 days	46872600.00	32819400.00	79692000.00	2242430.00	67743.50	2310173.50	2.82
	9 days	49915100.00	35436900.00	93017200.00	2059980.00	977342.00	3037322.00	3.44
	12 days	45398200.00	31443800.00	79692000.00	2878130.00	000	2878130.00	3.61

Table S83 1 molar equivalent of [mTBNH][OAc] and 1 molar equivalents of water.

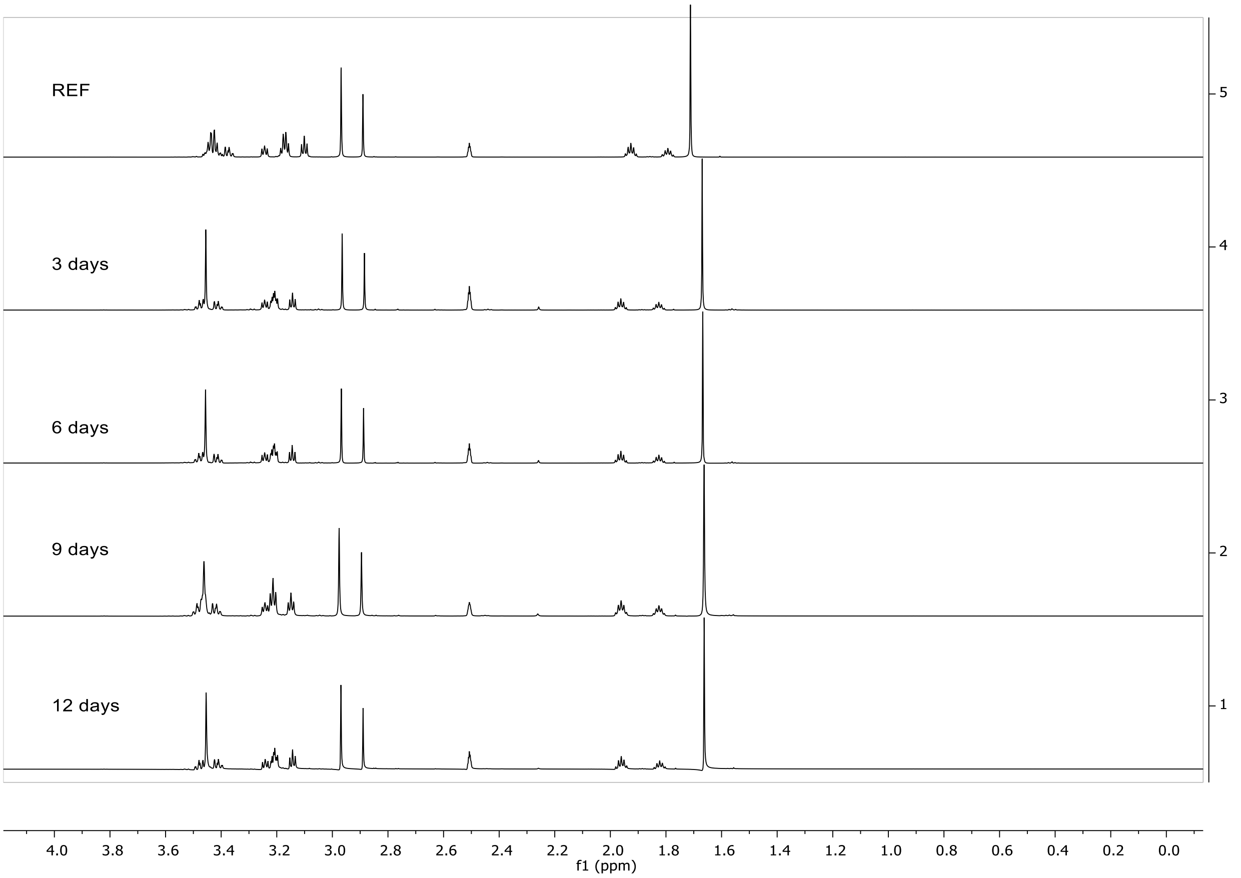

Figure S83 ^1H NMR 1 molar equivalent of [mTBNH][OAc] and 1 molar equivalents of water.

Structure	Time	Integral N-Methyl 1	Integral N-Methyl 2	Integral N Methyl total	Integral HP 1	Integral HP 2	Integral HP Total	% HP
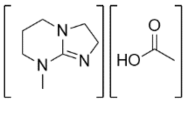	3 days	34039100.00	23021700.00	57060800.00	487475.00	434087.00	921562.00	0.00
	6 days	29677500.00	20901000.00	50578500.00	713798.00	635828.00	1349626.00	2.60
	9 days	37423000.00	26251300.00	63674300.00	1117470.00	896663.00	2014133.00	3.07
	12 days	29474800.00	21031000.00	57060800.00	1172510.00	963284.00	2135794.00	4.06

Table S84 1 molar equivalent of [mTBNH][OAc] and 5 molar equivalents of water.

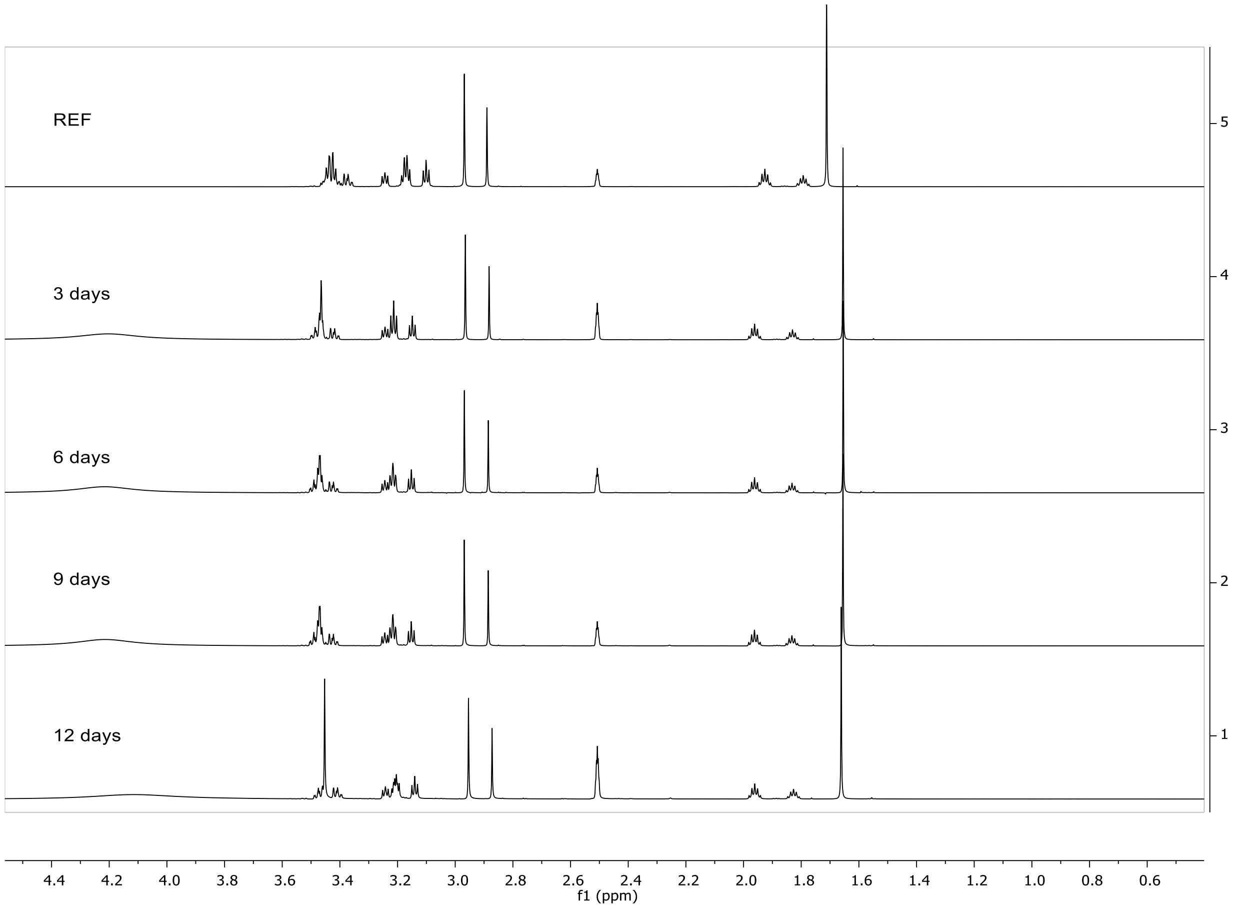

Figure S84 ^{1}H NMR 1 molar equivalent of [mTBNH][OAc] and 5 molar equivalents of water.

Structure	Time	Integral N-Methyl 1	Integral N-Methyl 2	Integral N Methyl total	Integral HP 1	Integral HP 2	Integral HP Total	% HP
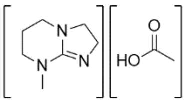	3 days	30104700.00	19942700.00	50047400.00	39123.00	0.00	39123.00	0.00
	6 days	22034100.00	14994000.00	37028100.00	273432.00	0.00	273432.00	0.73
	9 days	32627500.00	23188100.00	55815600.00	803870.00	0.00	803870.00	1.42
	12 days	22478100.00	16450300.00	38928400.00	643565.00	0.00	643565.00	1.63

Table S85 1 molar equivalent of [mTBNH][OAc] and 10 molar equivalents of water.

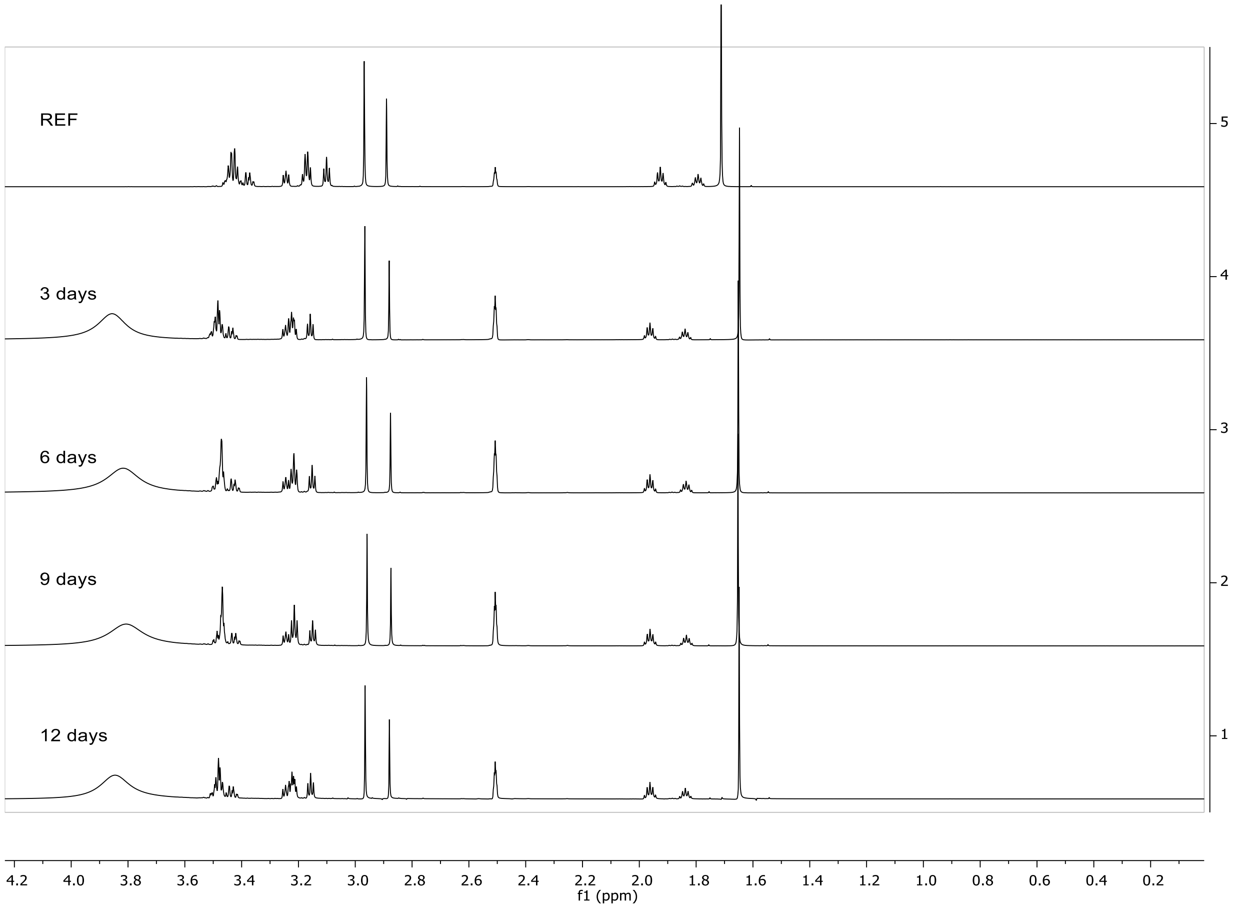

Figure S85 ^{1}H NMR 1 molar equivalent of [mTBNH][OAc] and 10 molar equivalents of water.

d. [mTBNH][ClOAc] + water

structure	Time	Integral N-Methyl 1	Integral N-Methyl 2	Integral N Methyl total	Integral HP 1	Integral HP 2	Integral HP Total	% HP
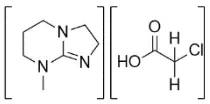	REF	49993600.00	33658200.00	83651800.00	0.00	0.00	0.00	0.00
	1h	59393600.00	40208500.00	99602100.00	0.00	0.00	0.00	0.00
	2h	48709000.00	33100100.00	81809100.00	0.00	0.00	0.00	0.00
	3h	51996700.00	35567500.00	87564200.00	0.00	0.00	0.00	0.00
	4h	59885200.00	40419400.00	100304600.00	0.00	0.00	0.00	0.00
	5h	53025400.00	36103500.00	89128900.00	0.00	0.00	0.00	0.00
	6h	55324000.00	37274100.00	92598100.00	0.00	0.00	0.00	0.00
	7h	54383600.00	36843400.00	91227000.00	0.00	0.00	0.00	0.00
	8h	55411800.00	35650100.00	91061900.00	0.00	0.00	0.00	0.00
	24h	46449800.00	31365700.00	77815500.00	0.00	0.00	0.00	0.00
	48h	54093700.00	35271500.00	89365200.00	0.00	0.00	0.00	0.00

Table S86 1 molar equivalent of [mTBNH][ClOAc] and 0.2 molar equivalents of water.

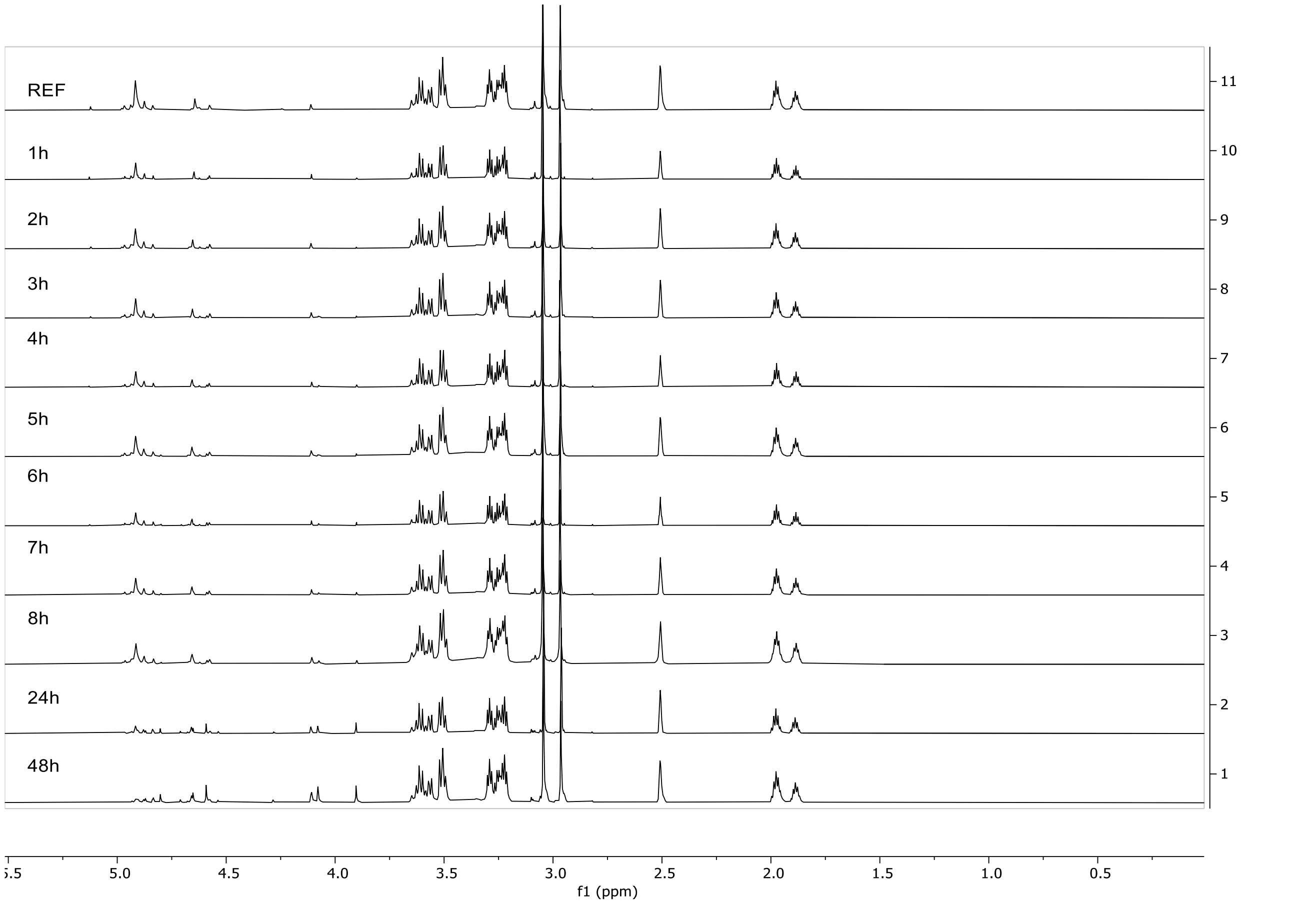

Figure S86 ^{1}H NMR 1 molar equivalent of [mTBNH][ClOAc] and 0.2 molar equivalents of water.

Structure	Time	Integral N-Methyl	Integral N-Methyl	Integral N Methyl total	Integral HP 1	Integral HP 2	Integral HP Total	% HP
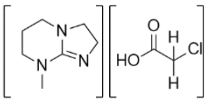	REF	54592000.00	37083600.00	91675600.00	0.00	0.00	0.00	0.00
	1h	45425800.00	27212400.00	72638200.00	0.00	0.00	0.00	0.00
	2h	49262300.00	33432100.00	82694400.00	0.00	0.00	0.00	0.00
	3h	54292200.00	36533600.00	90825800.00	0.00	0.00	0.00	0.00
	4h	51045100.00	34840100.00	85885200.00	0.00	0.00	0.00	0.00
	5h	53670900.00	36204100.00	89875000.00	0.00	0.00	0.00	0.00
	6h	50972500.00	34739200.00	85711700.00	0.00	0.00	0.00	0.00
	7h	49074600.00	32202400.00	81277000.00	0.00	0.00	0.00	0.00
	8h	50352800.00	34264200.00	84617000.00	0.00	0.00	0.00	0.00
	24h	59369500.00	39553500.00	98923000.00	0.00	0.00	0.00	0.00
	48h	56830200.00	37710200.00	94540400.00	0.00	0.00	0.00	0.00

Table S87 1 molar equivalent of [mTBNH][ClOAc] and 0.5 molar equivalents of water.

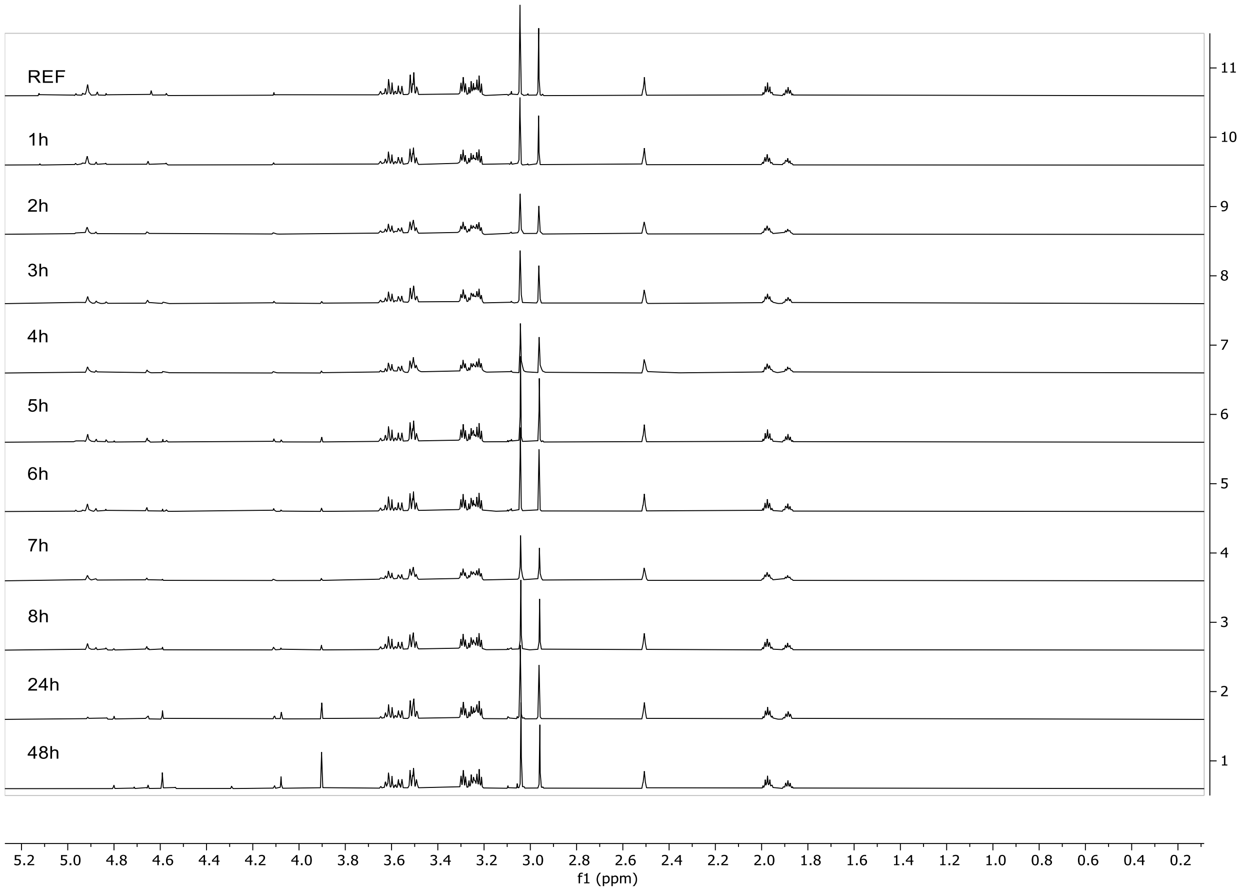

Figure S87 ^{1}H NMR 1 molar equivalent of [mTBNH][ClOAc] and 0.5 molar equivalents of water.

Structure	Time	Integral N-Methyl	Integral N-Methyl	Integral N Methyl total	Integral HP 1	Integral HP 2	Integral HP Total	% HP
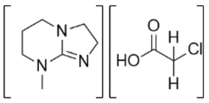	REF	41257400.00	28927900.00	70185300.00	0.00	0.00	0.00	0.00
	1h	48790500.00	34278600.00	83069100.00	0.00	0.00	0.00	0.00
	2h	47353300.00	33078000.00	80431300.00	0.00	0.00	0.00	0.00
	3h	47992600.00	33598800.00	81591400.00	0.00	0.00	0.00	0.00
	4h	51372200.00	35706100.00	87078300.00	0.00	0.00	0.00	0.00
	5h	54546000.00	38109800.00	92655800.00	0.00	0.00	0.00	0.00
	6h	47423400.00	33141300.00	80564700.00	0.00	0.00	0.00	0.00
	7h	50459100.00	35216100.00	85675200.00	0.00	0.00	0.00	0.00
	8h	49850300.00	34870400.00	84720700.00	0.00	0.00	0.00	0.00
	24h	58990400.00	40699300.00	99689700.00	0.00	0.00	0.00	0.00
	48h	54850600.00	39352100.00	94202700.00	0.00	0.00	0.00	0.00

Table S88 1 molar equivalent of [mTBNH][ClOAc] and 1 molar equivalents of water.

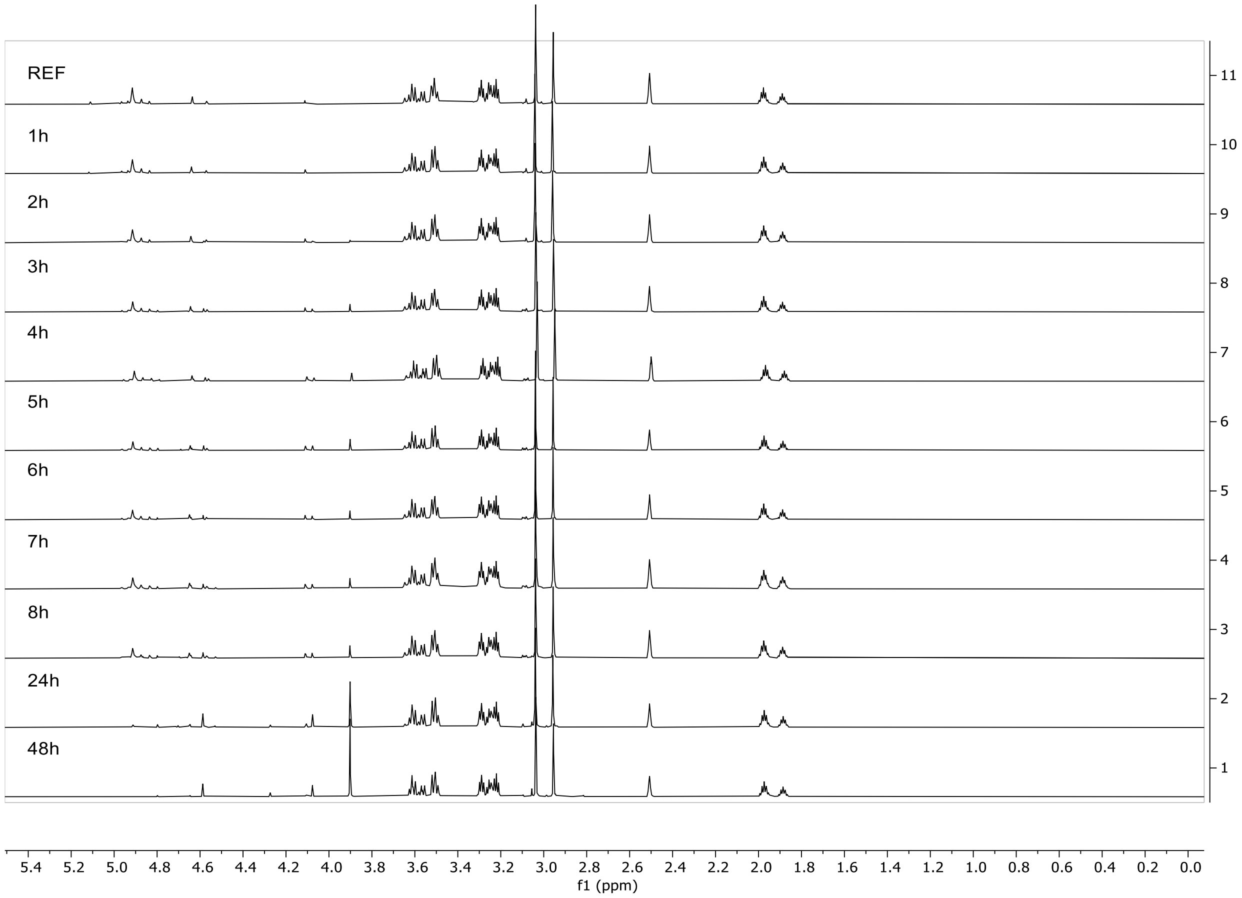

Figure S88 ^{1}H NMR 1 molar equivalent of [mTBNH][ClOAc] and 1 molar equivalents of water.

Structure	Time	Integral N-Methyl	Integral N-Methyl	Integral N Methyl total	Integral HP 1	Integral HP 2	Integral HP Total	% HP
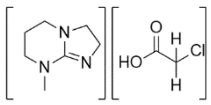	REF	163742000.00	111893000.00	275635000.00	0.00	0.00	0.00	0.00
	1h	177484000.00	120743000.00	298227000.00	0.00	0.00	0.00	0.00
	2h	169317000.00	114283000.00	283600000.00	0.00	0.00	0.00	0.00
	3h	152955000.00	101732000.00	254687000.00	0.00	0.00	0.00	0.00
	4h	174700000.00	116986000.00	291686000.00	0.00	0.00	0.00	0.00
	5h	171060000.00	115399000.00	286459000.00	0.00	0.00	0.00	0.00
	6h	176529000.00	119765000.00	296294000.00	0.00	0.00	0.00	0.00
	7h	138106000.00	95870100.00	233976100.00	0.00	0.00	0.00	0.00
	8h	155870000.00	107448000.00	263318000.00	0.00	0.00	0.00	0.00
	24h	152868000.00	105868000.00	258736000.00	0.00	0.00	0.00	0.00
	48h	148394000.00	106352000.00	254746000.00	0.00	0.00	0.00	0.00

Table S89 1 molar equivalent of [mTBNH][ClOAc] and 5 molar equivalents of water.

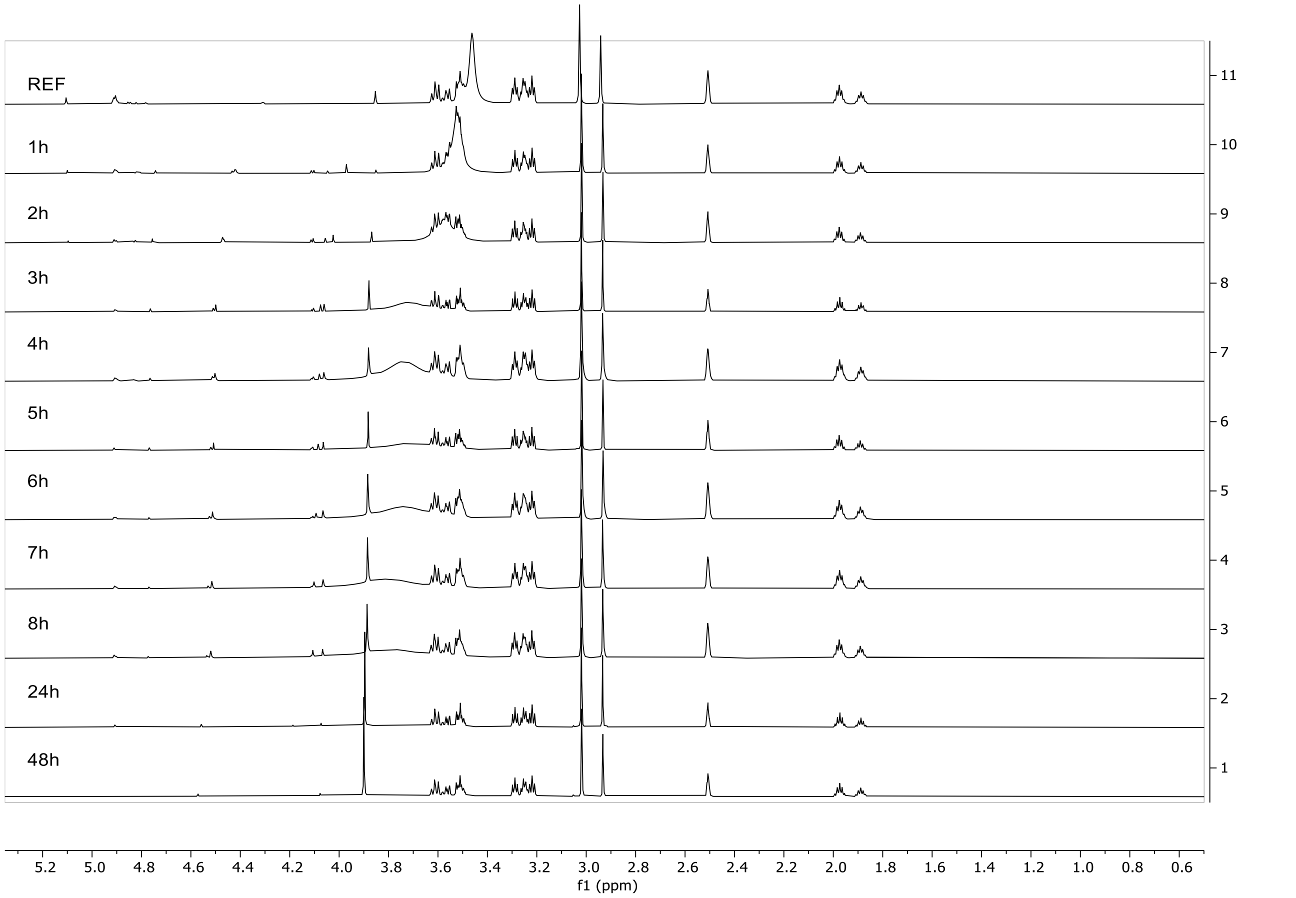

Figure S89 ^{1}H NMR 1 molar equivalent of [mTBNH][ClOAc] and 5 molar equivalents of water.

Structure	HT_110	Integral N-Methyl	Integral N-Methyl	Integral N Methyl total	Integral HP 1	Integral HP 2	Integral HP Total	% HP
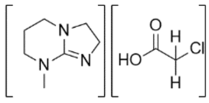	REF	44152400.00	29862400.00	74014800.00	0.00	0.00	0.00	
	1h	55578600.00	37861300.00	93439900.00	0.00	0.00	0.00	0.00
	2h	49818300.00	33980600.00	83798900.00	0.00	0.00	0.00	0.00
	3h	48734100.00	32947500.00	81681600.00	0.00	0.00	0.00	0.00
	4h	50054800.00	33951500.00	84006300.00	0.00	0.00	0.00	0.00
	5h	51267300.00	34400700.00	85668000.00	0.00	0.00	0.00	0.00
	6h	49420300.00	33304400.00	82724700.00	0.00	0.00	0.00	0.00
	7h	55917100.00	37479400.00	93396500.00	0.00	0.00	0.00	0.00
	8h	49287200.00	33073000.00	82360200.00	0.00	0.00	0.00	0.00
	24h	51774000.00	34342900.00	86116900.00	0.00	0.00	0.00	0.00
	48h	60739300.00	40960300.00	101699600.00	0.00	0.00	0.00	0.00

Table S90 1 molar equivalent of [mTBNH][ClOAc] and 10 molar equivalents of water.

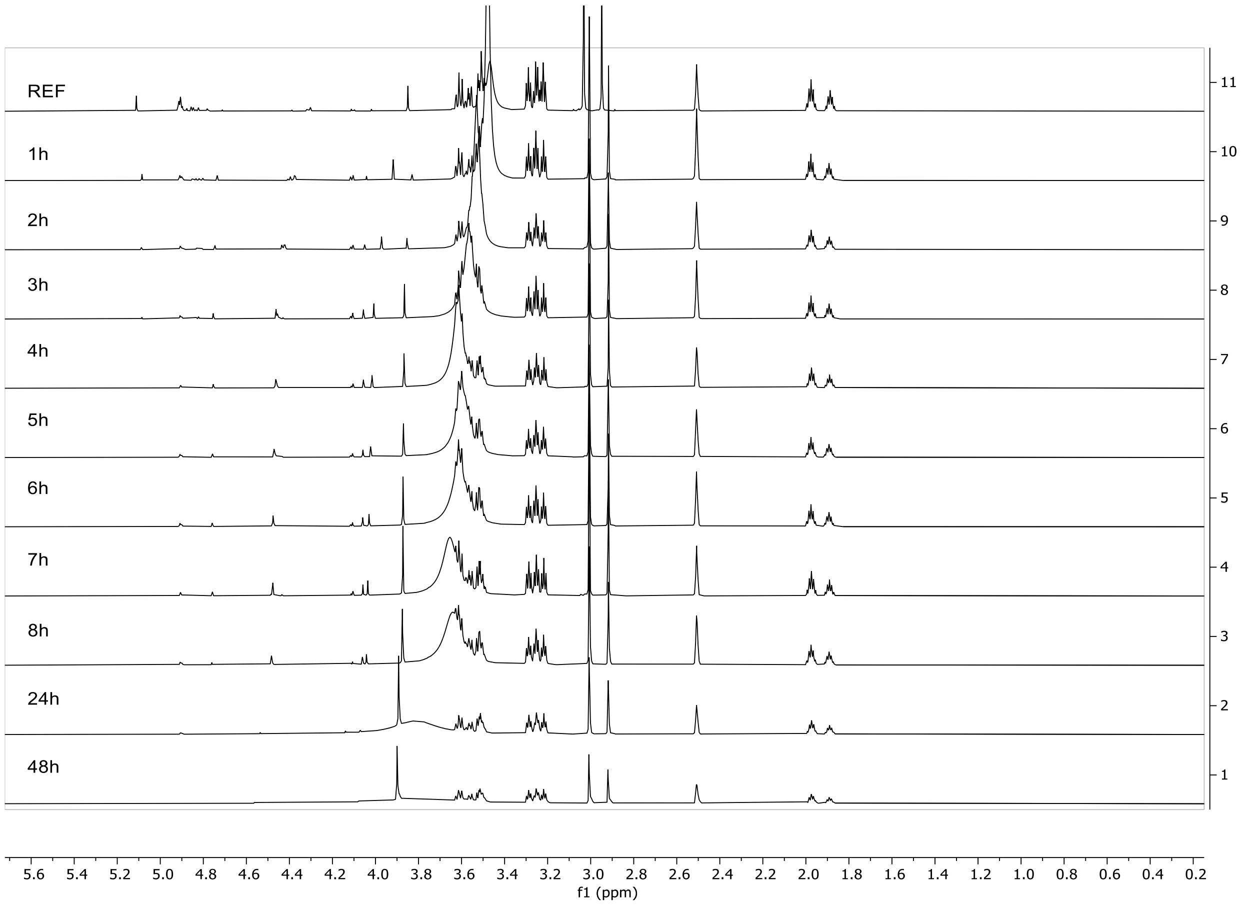

Figure S90 ^{1}H NMR 1 molar equivalent of [mTBNH][ClOAc] and 10 molar equivalents of water.

e. [mTBNH][Cl2OAc] + water

Structure	Time	Integral N-Methyl 1	Integral N-Methyl 2	Integral N Methyl total	Integral HP 1	Integral HP 2	Integral HP Total	% HP
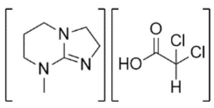	REF	49416000.00	34589200.00	84005200.00	0.00	0.00	0.00	0.00
	1h	53690900.00	36806700.00	90497600.00	0.00	0.00	0.00	0.00
	2h	53374100.00	36748300.00	90122400.00	0.00	0.00	0.00	0.00
	3h	56157400.00	39395800.00	95553200.00	0.00	0.00	0.00	0.00
	4h	55627700.00	39705300.00	95333000.00	0.00	0.00	0.00	0.00
	5h	56240300.00	40415800.00	96656100.00	0.00	0.00	0.00	0.00
	6h	62549900.00	44395100.00	106945000.00	0.00	0.00	0.00	0.00
	7h	69670000.00	49756700.00	119426700.00	0.00	0.00	0.00	0.00
	8h	68664700.00	49462300.00	118127000.00	0.00	0.00	0.00	0.00
	24h	55887000.00	45729100.00	101616100.00	0.00	0.00	0.00	0.00
	48h	60799100.00	63193600.00	123992700.00	0.00	0.00	0.00	0.00

Table S91 1 molar equivalent of [mTBNH][Cl_2OAc] and 0.2 molar equivalents of water.

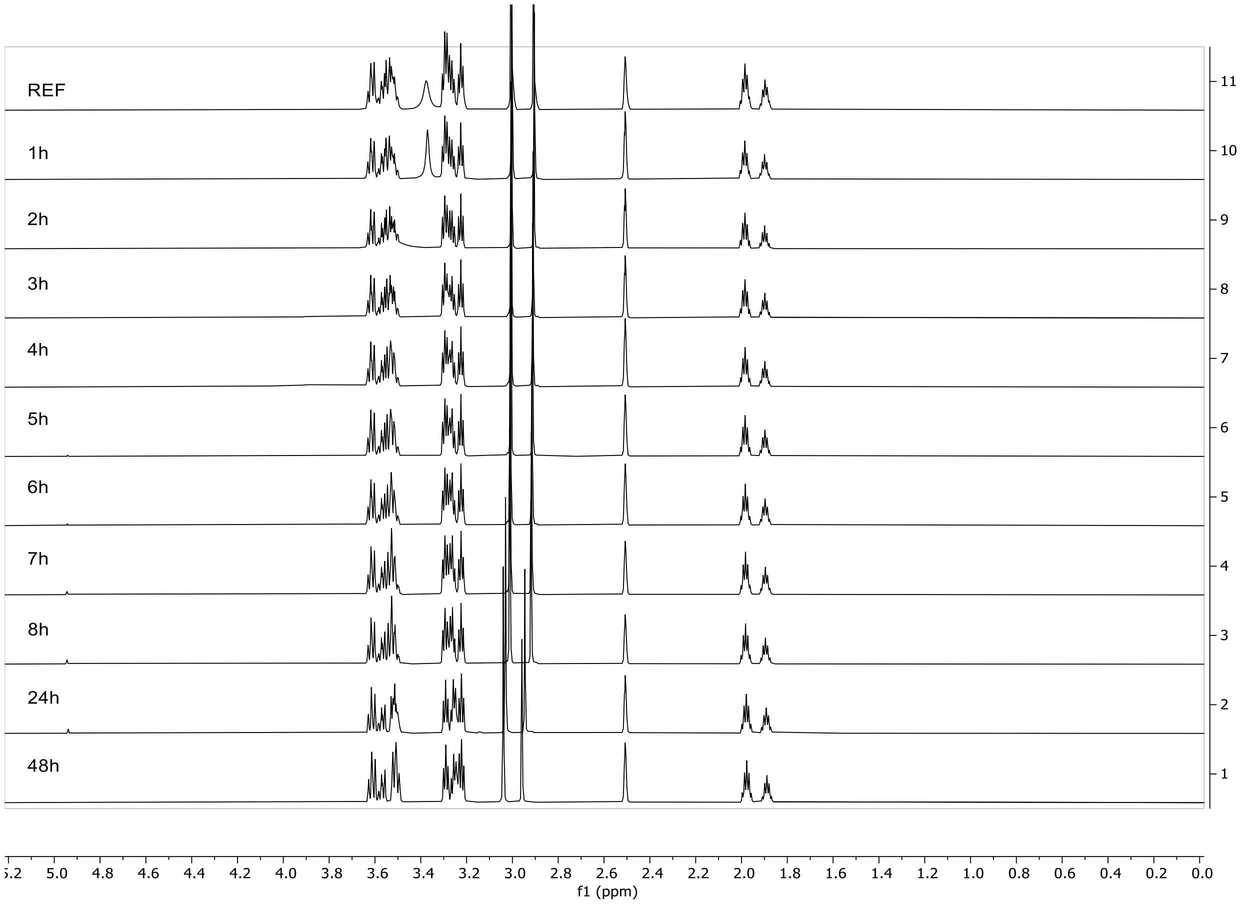

Figure S91 ^{1}H NMR 1 molar equivalent of [mTBNH][Cl_2OAc] and 0.2 molar equivalents of water.

Structure	Time	Integral N-Methyl	Integral N-Methyl	Integral N Methyl total	Integral HP 1	Integral HP 2	Integral HP Total	% HP
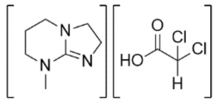	REF	58972100.00	39794100	98766200.00	0.00	0.00	0.00	0.00
	1h	60670600.00	40671400	101342000.00	0.00	0.00	0.00	0.00
	2h	67166100.00	45692700	112858800.00	0.00	0.00	0.00	0.00
	3h	61292900.00	41539100	102832000.00	0.00	0.00	0.00	0.00
	4h	59335600.00	40555000	99890600.00	0.00	0.00	0.00	0.00
	5h	60120700.00	41172200	101292900.00	0.00	0.00	0.00	0.00
	6h	67885000.00	46948000	114833000.00	0.00	0.00	0.00	0.00
	7h	63554300.00	43095700	106650000.00	0.00	0.00	0.00	0.00
	8h	54637500.00	37831600	92469100.00	0.00	0.00	0.00	0.00
	24h	57165700.00	42627000	99792700.00	0.00	0.00	0.00	0.00
	48h	60992700.00	46202700	107195400.00	0.00	0.00	0.00	0.00

Table S92 1 molar equivalent of [mTBNH][Cl_2OAc] and 0.5 molar equivalents of water.

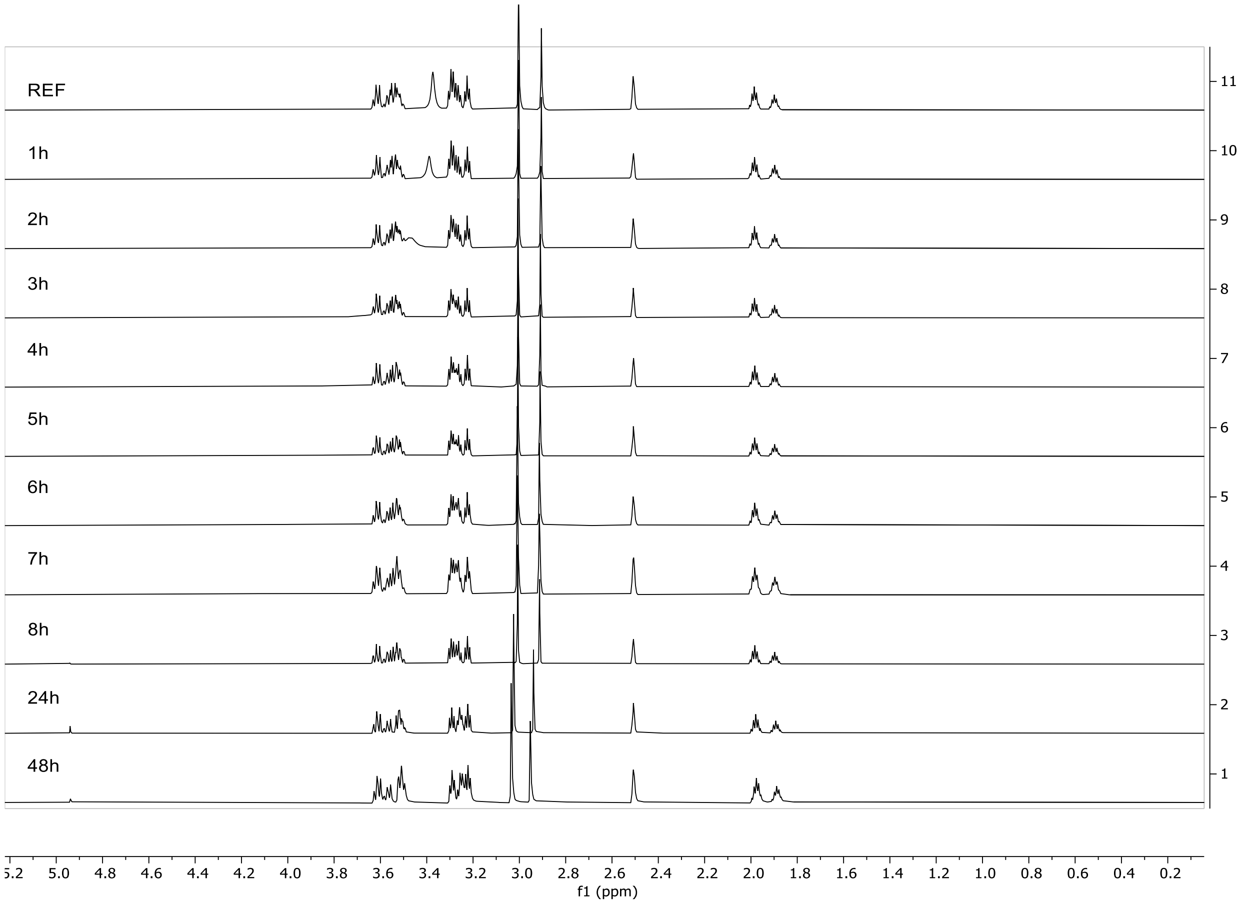

Figure S92 ^{1}H NMR 1 molar equivalent of [mTBNH][Cl_2OAc] and 0.5 molar equivalents of water.

Structure	Time	Integral N-Methyl	Integral N-Methyl	Integral N Methyl total	Integral HP 1	Integral HP 2	Integral HP Total	% HP
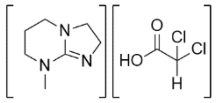	REF	53584700.00	36078000.00	89662700.00	0.00	0.00	0.00	0.00
	1h	60230500.00	40593300.00	100823800.00	0.00	0.00	0.00	0.00
	2h	50106400.00	33874600.00	83981000.00	0.00	0.00	0.00	0.00
	3h	54275500.00	36697000.00	90972500.00	0.00	0.00	0.00	0.00
	4h	58302700.00	39503900.00	97806600.00	0.00	0.00	0.00	0.00
	5h	53256900.00	35963800.00	89220700.00	0.00	0.00	0.00	0.00
	6h	58406800.00	39976000.00	98382800.00	0.00	0.00	0.00	0.00
	7h	49470600.00	33425500.00	82896100.00	0.00	0.00	0.00	0.00
	24h	58567800.00	39744700.00	98312500.00	0.00	0.00	0.00	0.00
	48h	57530800.00	39998900.00	97529700.00	0.00	0.00	0.00	0.00

Table S93 1 molar equivalent of [mTBNH][Cl_2OAc] and 1 molar equivalents of water.

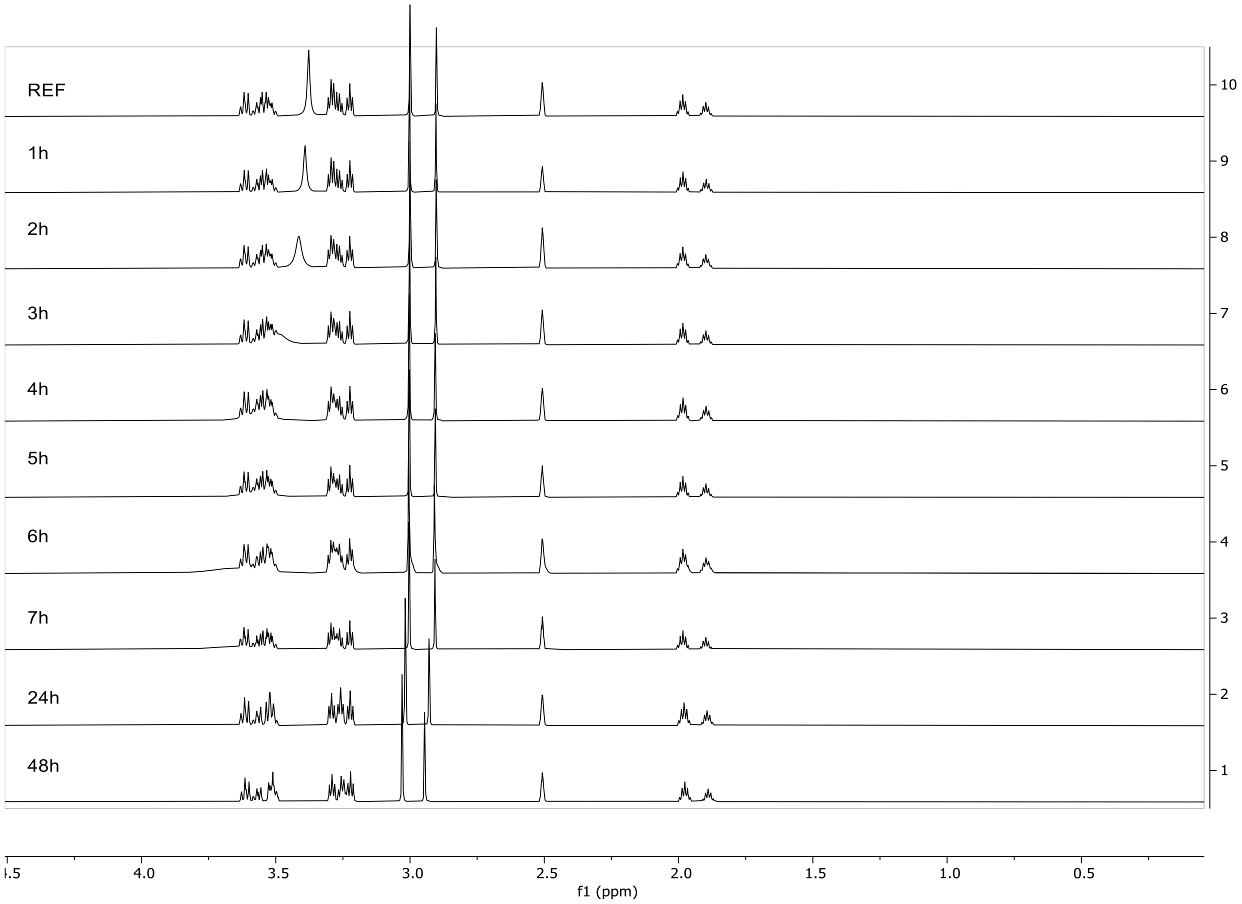

Figure S93 ^{1}H NMR 1 molar equivalent of [mTBNH][Cl_2OAc] and 1 molar equivalents of water.

Structure	Time	Integral N-Methyl	Integral N-Methyl	Integral N Methyl total	Integral HP 1	Integral HP 2	Integral HP Total	% HP
	REF	48730000.00	32702000.00	81432000.00	0.00	0.00	0.00	0.00
	1h	50781200.00	33994000.00	84775200.00	0.00	0.00	0.00	0.00
	2h	47436800.00	31866000.00	79302800.00	0.00	0.00	0.00	0.00
	3h	47653700.00	32029000.00	79682700.00	0.00	0.00	0.00	0.00
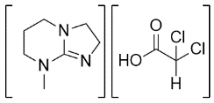	4h	43698700.00	29351200.00	73049900.00	0.00	0.00	0.00	0.00
	6h	23030900.00	15418100.00	38449000.00	0.00	0.00	0.00	0.00
	7h	48196200.00	32344900.00	80541100.00	0.00	0.00	0.00	0.00
	8h	44839900.00	30087300.00	74927200.00	0.00	0.00	0.00	0.00
	24h	51202100.00	35055500.00	86257600.00	0.00	0.00	0.00	0.00
	48h	56626500.00	39513700.00	96140200.00	0.00	0.00	0.00	0.00

Table S94 1 molar equivalent of [mTBNH][Cl_2OAc] and 5 molar equivalents of water.

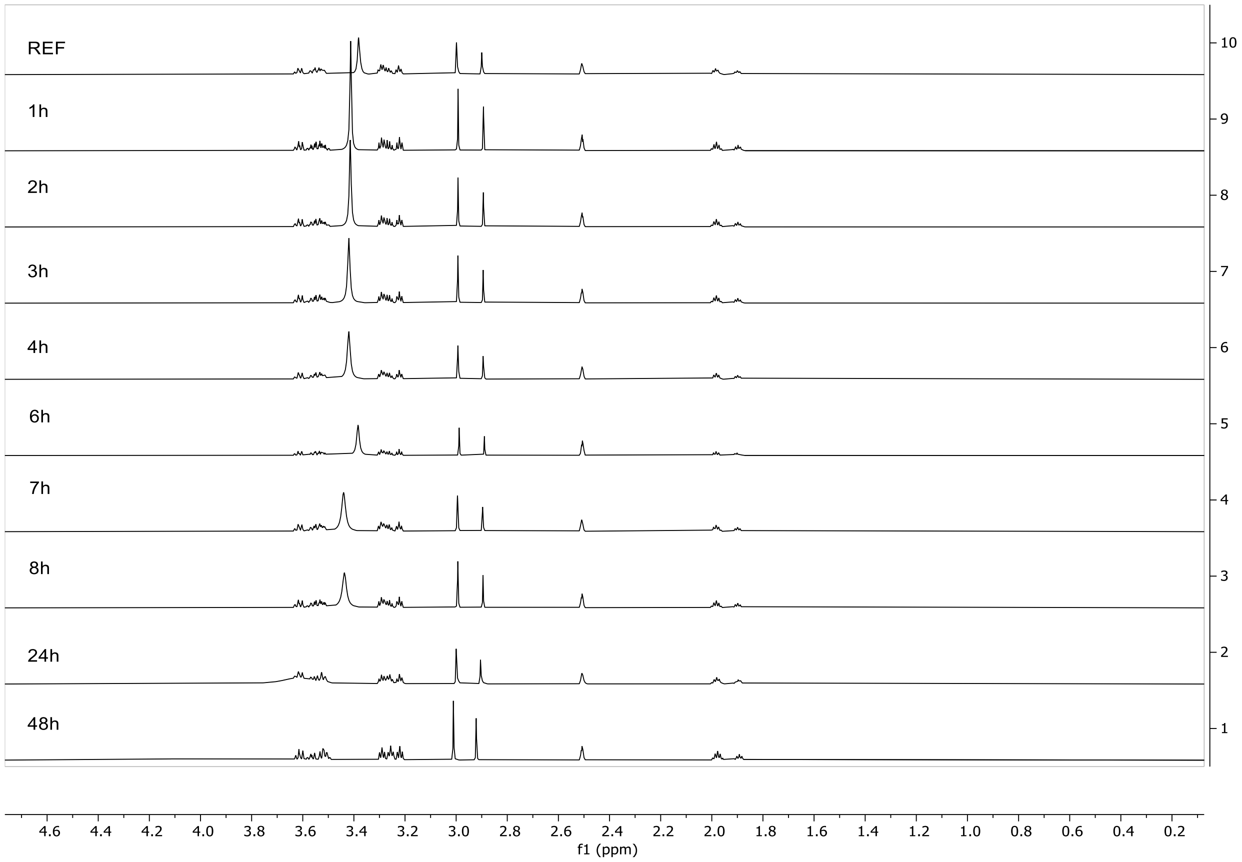

Figure S94 ^{1}H NMR 1 molar equivalent of [mTBNH][Cl_2OAc] and 5 molar equivalents of water.

Structure	Time	Integral N-Methyl	Integral N-Methyl	Integral N Methyl total	Integral HP 1	Integral HP 2	Integral HP Total	% HP
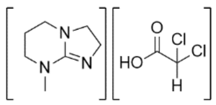	REF	36885200.00	24715500.00	61600700.00	0.00	0.00	0.00	0.00
	1h	27659700.00	18526100.00	46185800.00	0.00	0.00	0.00	0.00
	2h	26020000.00	17368500.00	43388500.00	0.00	0.00	0.00	0.00
	3h	23823600.00	15927200.00	39750800.00	0.00	0.00	0.00	0.00
	4h	24080000.00	16128900.00	40208900.00	0.00	0.00	0.00	0.00
	5h	27268600.00	18277600.00	45546200.00	0.00	0.00	0.00	0.00
	6h	30634700.00	20477500.00	51112200.00	0.00	0.00	0.00	0.00
	7h	28260400.00	18965700.00	47226100.00	0.00	0.00	0.00	0.00
	8h	25225600.00	16940500.00	42166100.00	0.00	0.00	0.00	0.00
	24h	27959400.00	19037800.00	46997200.00	0.00	0.00	0.00	0.00
	48h	31350400.00	21496200.00	52846600.00	0.00	0.00	0.00	0.00

Table S95 1 molar equivalent of [mTBNH][Cl_2OAc] and 10 molar equivalents of water.

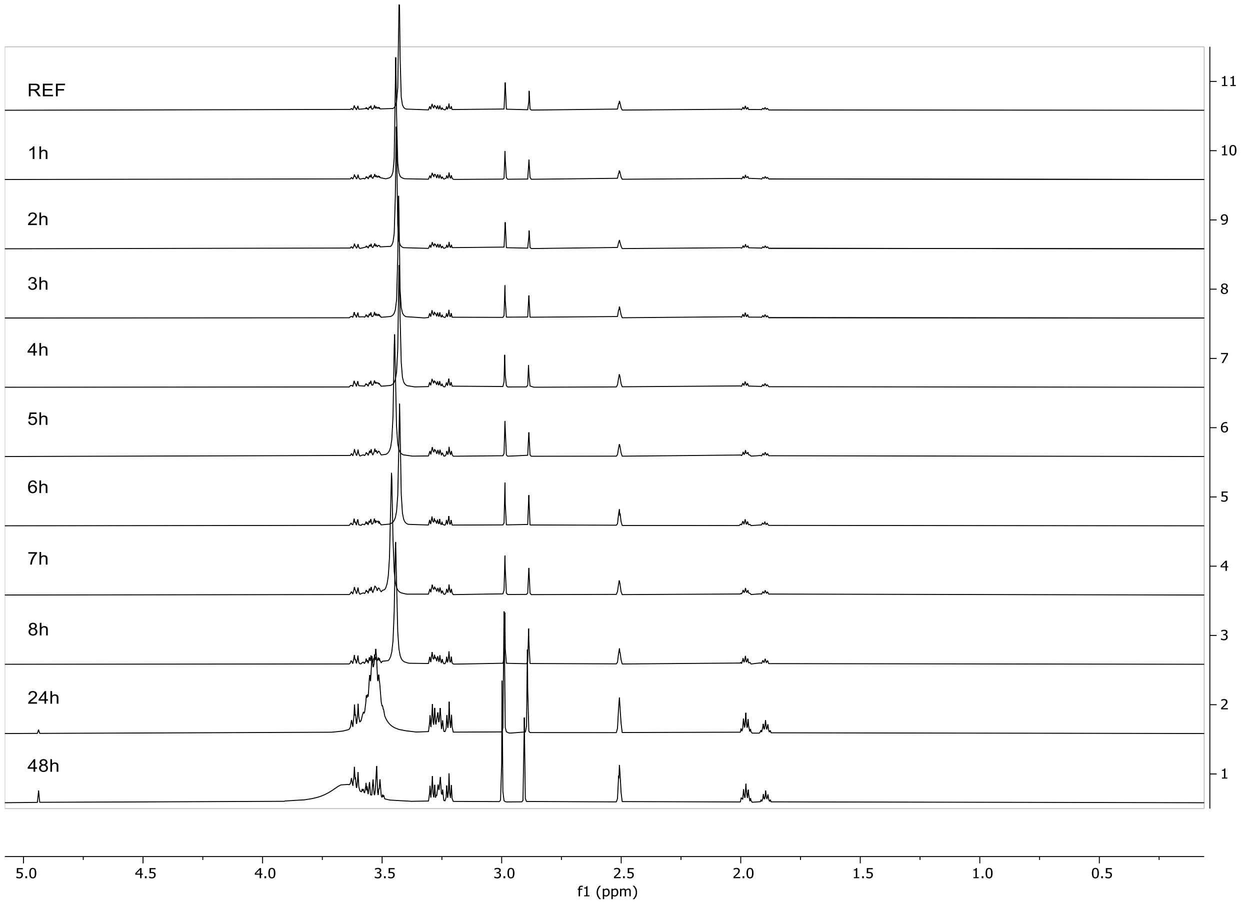

Figure S95 ^{1}H NMR 1 molar equivalent of [mTBNH][Cl_2OAc] and 10 molar equivalents of water.

f. [mTBNH][Cl3OAc] + water

Structure	Time	Integral N-Methyl 1	Integral N-Methyl 2	Integral N Methyl total	Integral HP 1	Integral HP 2	Integral HP Total	% HP
	REF	70597300.00	48727400.00	119324700.00	0.00	0.00	0.00	0.00
	1h	75005000.00	53074300.00	128079300.00	0.00	0.00	0.00	0.00
	2h	66565900.00	47641400.00	114207300.00	0.00	0.00	0.00	0.00
	3h	51696200.00	37366400.00	89062600.00	0.00	0.00	0.00	0.00
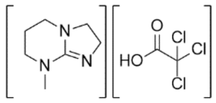	4h	53352400.00	38475100.00	91827500.00	0.00	0.00	0.00	0.00
	5h	55255700.00	40838800.00	96094500.00	0.00	0.00	0.00	0.00
	6h	66521800.00	49858200.00	116380000.00	0.00	0.00	0.00	0.00
	7h	50095100.00	37070700.00	87165800.00	0.00	0.00	0.00	0.00
	8h	58891000.00	46119700.00	105010700.00	0.00	0.00	0.00	0.00
	24h	69046100.00	51284500.00	120330600.00	0.00	0.00	0.00	0.00
	48h	24277300.00	18077500.00	42354800.00	0.00	0.00	0.00	0.00

Table S96 1 molar equivalent of [mTBNH][Cl_3OAc] and 0.2 molar equivalents of water.

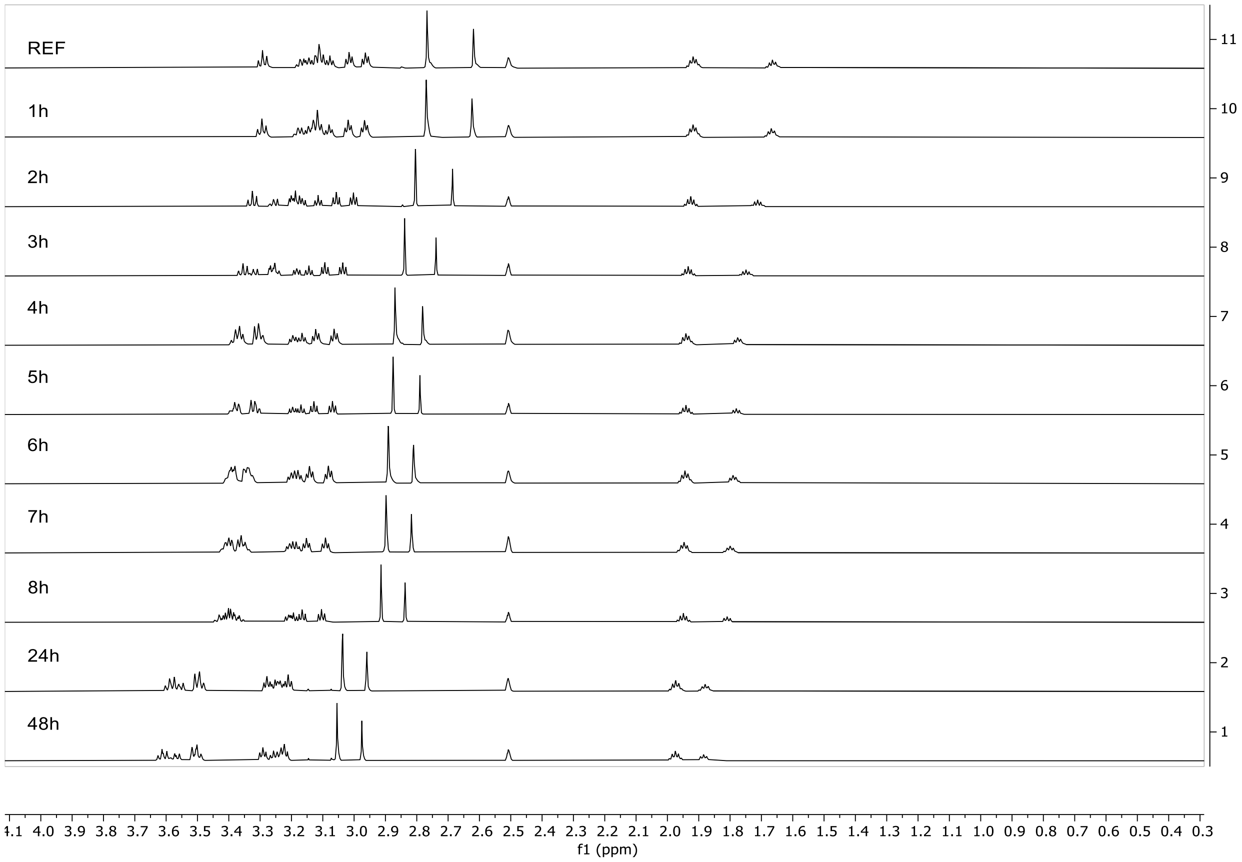

Figure S96 ^{1}H NMR 1 molar equivalent of [mTBNH][Cl_3OAc] and 0.2 molar equivalents of water.

Structure	Time	Integral N-Methyl	Integral N-Methyl	Integral N Methyl total	Integral HP 1	Integral HP 2	Integral HP Total	% HP
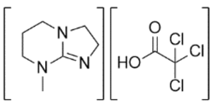	REF	60311100.00	42933300.00	103244400.00	0.00	0.00	0.00	0.00
	1h	77361600.00	54632500.00	131994100.00	0.00	0.00	0.00	0.00
	2h	59379200.00	43761800.00	103141000.00	0.00	0.00	0.00	0.00
	3h	66249800.00	50787700.00	117037500.00	0.00	0.00	0.00	0.00
	4h	49749000.00	40038100.00	89787100.00	0.00	0.00	0.00	0.00
	5h	61127400.00	47910200.00	109037600.00	0.00	0.00	0.00	0.00
	6h	50391400.00	40128400.00	90519800.00	0.00	0.00	0.00	0.00
	7h	60366400.00	50751900.00	111118300.00	0.00	0.00	0.00	0.00
	8h	52655600.00	45979500.00	98635100.00	0.00	0.00	0.00	0.00
	24h	45872100.00	35205900.00	81078000.00	0.00	0.00	0.00	0.00
	48h	58240500.00	42403400.00	100643900.00	0.00	0.00	0.00	0.00

Table S97 1 molar equivalent of [mTBNH][Cl_3OAc] and 0.5 molar equivalents of water.

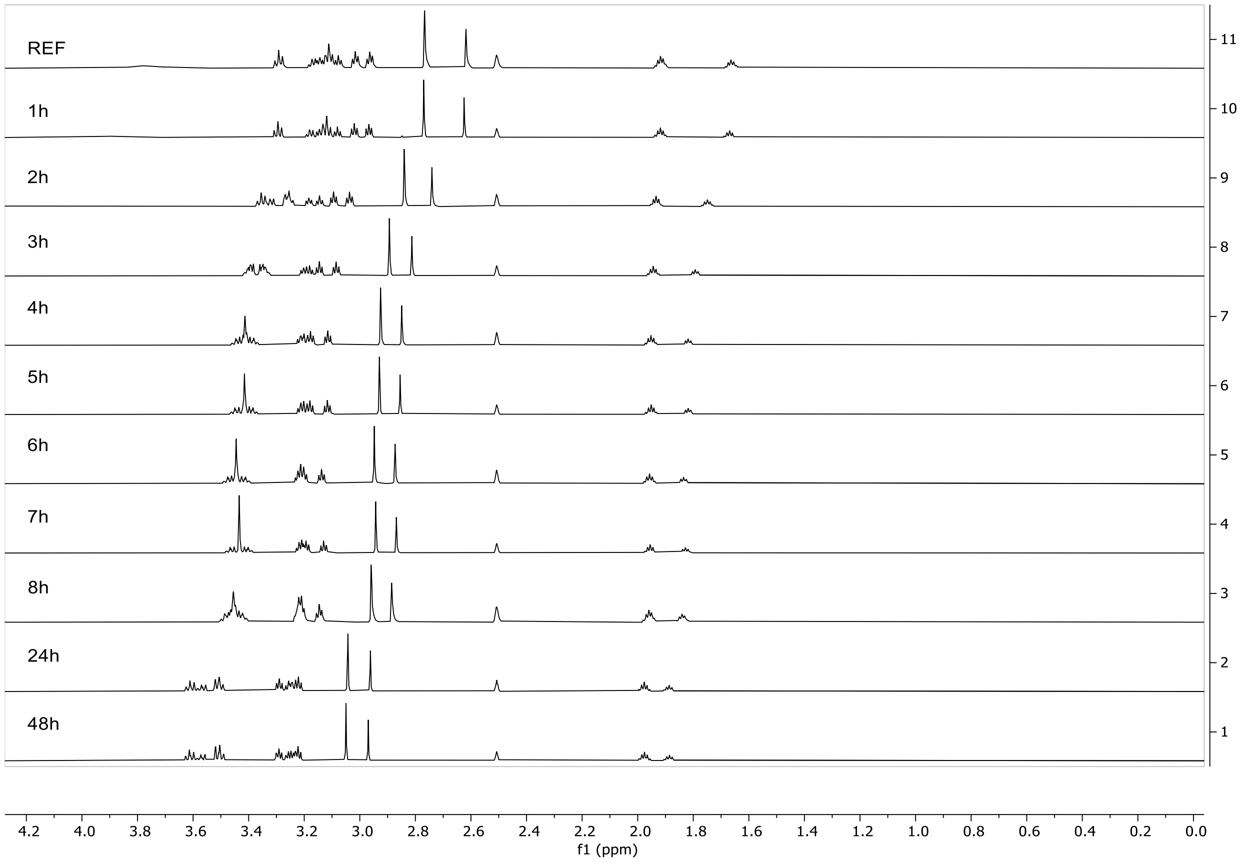

Figure S97 ^{1}H NMR 1 molar equivalent of [mTBNH][Cl_3OAc] and 0.5 molar equivalents of water.

Structure	Time	Integral N-Methyl	Integral N-Methyl	Integral N Methyl total	Integral HP 1	Integral HP 2	Integral HP Total	% HP
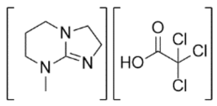	REF	65028500.00	42904400.00	107932900.00	0.00	0.00	0.00	0.00
	1h	55118700.00	37663400.00	92782100.00	0.00	0.00	0.00	0.00
	2h	58843100.00	40425500.00	99268600.00	0.00	0.00	0.00	0.00
	3h	58808200.00	40665100.00	99473300.00	0.00	0.00	0.00	0.00
	4h	58013500.00	39619700.00	97633200.00	0.00	0.00	0.00	0.00
	5h	57359800.00	37166300.00	94526100.00	0.00	0.00	0.00	0.00
	6h	58818700.00	40376200.00	99194900.00	0.00	0.00	0.00	0.00
	7h	71732100.00	49117800.00	120849900.00	0.00	0.00	0.00	0.00
	8h	58468600.00	44319600.00	102788200.00	0.00	0.00	0.00	0.00
	24h	55578300.00	39291600.00	94869900.00	0.00	0.00	0.00	0.00
	48h	70409100.00	47828200.00	118237300.00	0.00	0.00	0.00	0.00

Table S98 1 molar equivalent of [mTBNH][Cl_3OAc] and 1 molar equivalent of water.

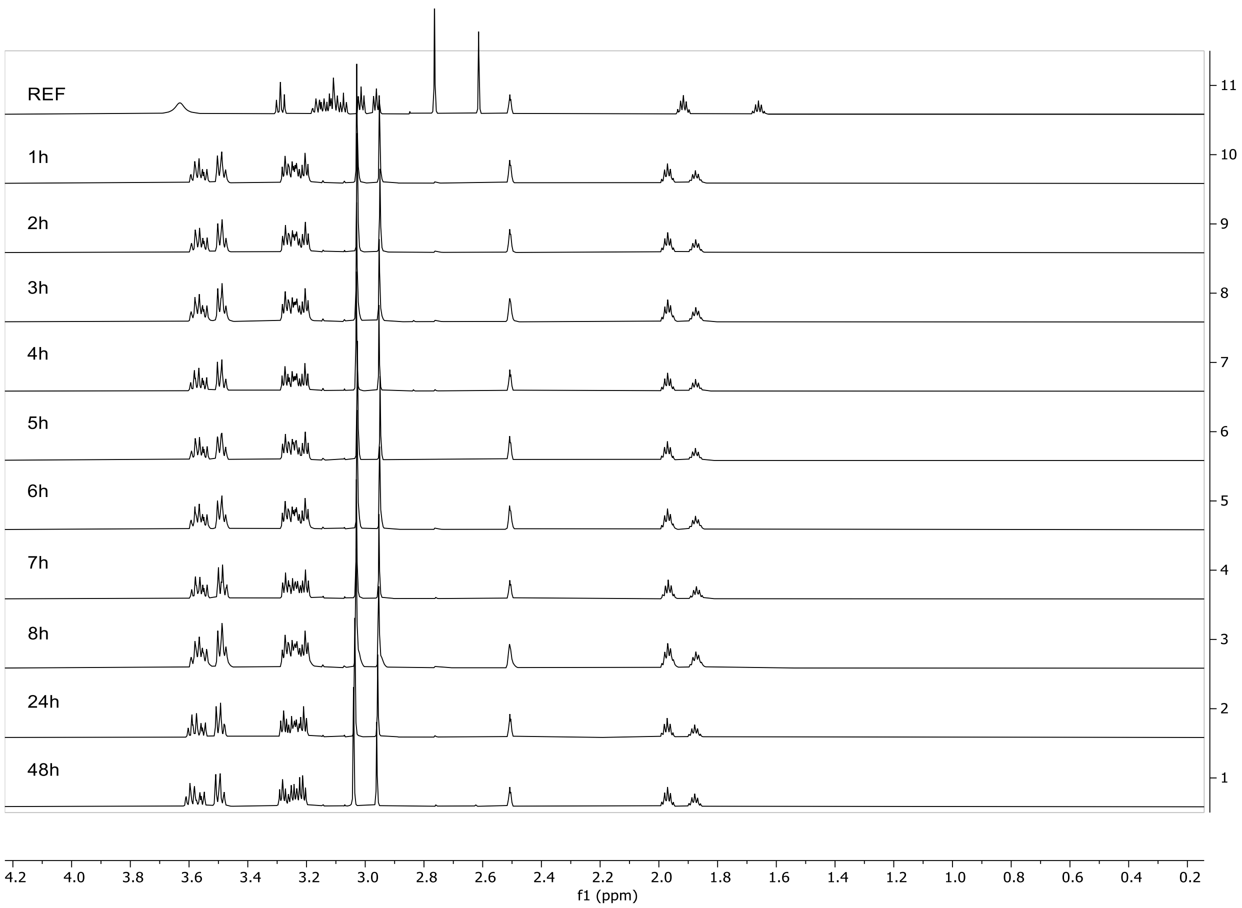

Figure S98 ^{1}H NMR 1 molar equivalent of [mTBNH][Cl_3OAc] and 1 molar equivalent of water.

Structure	Time	Integral N-Methyl	Integral N-Methyl	Integral N Methyl total	Integral HP 1	Integral HP 2	Integral HP Total	% HP
	REF	51746900.00	35079600.00	86826500.00	0.00	0.00	0.00	
	1h	2443240.00	1792960.00	4236200.00	0.00	0.00	0.00	0.00
	2h	33470800.00	23395900.00	56866700.00	0.00	0.00	0.00	0.00
	3h	46053100.00	31895800.00	77948900.00	0.00	0.00	0.00	0.00
	4h	42228100.00	29507400.00	71735500.00	0.00	0.00	0.00	0.00
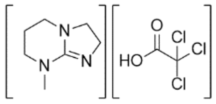	5h	46695000.00	32442000.00	79137000.00	0.00	0.00	0.00	0.00
	6h	40791400.00	28702100.00	69493500.00	0.00	0.00	0.00	0.00
	7h	44255800.00	30651400.00	74907200.00	0.00	0.00	0.00	0.00
	8h	50818000.00	35140200.00	85958200.00	0.00	0.00	0.00	0.00
	24h	41950300.00	29305700.00	71256000.00	0.00	0.00	0.00	0.00
	48h	46762800.00	32331500.00	79094300.00	0.00	0.00	0.00	0.00

Table S99 1 molar equivalent of [mTBNH][Cl_3OAc] and 5 molar equivalents of water.

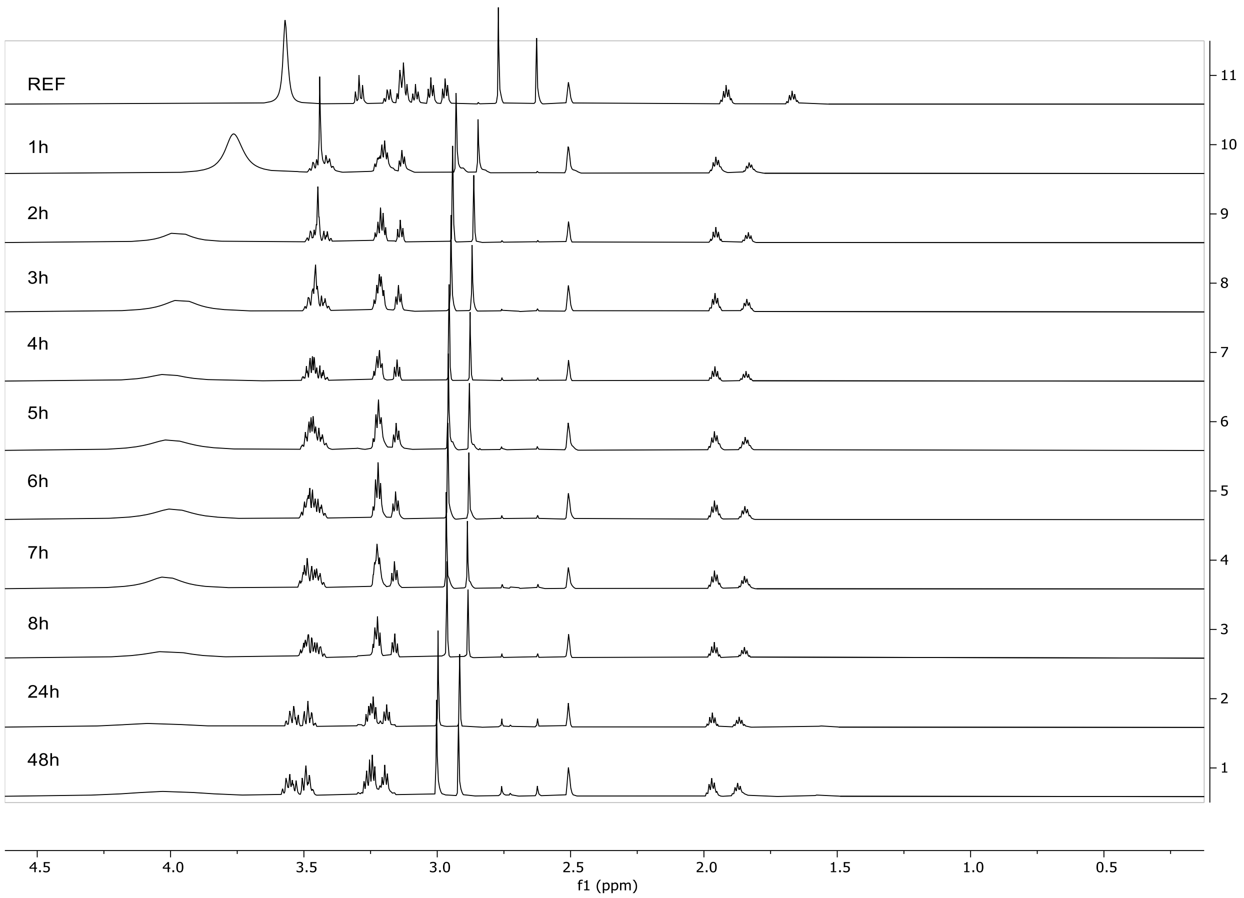

Figure S99 ^{1}H NMR 1 molar equivalent of [mTBNH][Cl_3OAc] and 5 molar equivalents of water.

Structure	Time	integral N-Methyl	integral N-Methyl	integral N Methyl total	integral HP 1	integral HP 2	integral HP Total	% HP
	REF	38717300.00	25923400.00	64640700.00	0.00	0.00	0.00	0.00
	1h	41541900.00	27782000.00	69323900.00	0.00	0.00	0.00	0.00
	2h	31895600.00	21536500.00	53432100.00	0.00	0.00	0.00	0.00
	3h	30799600.00	20925600.00	51725200.00	0.00	0.00	0.00	0.00
	4h	29150500.00	19556700.00	48707200.00	0.00	0.00	0.00	0.00
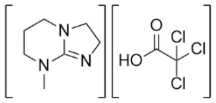	5h	29286600.00	20360800.00	49647400.00	0.00	0.00	0.00	0.00
	6h	31883300.00	22073600.00	53956900.00	0.00	0.00	0.00	0.00
	7h	34268800.00	23802100.00	58070900.00	0.00	0.00	0.00	0.00
	8h	38216100.00	26428400.00	64644500.00	0.00	0.00	0.00	0.00
	24h	35200300.00	25647100.00	60847400.00	0.00	0.00	0.00	0.00
	48h	34466100.00	25612900.00	60079000.00	0.00	0.00	0.00	0.00

Table S 100 1 molar equivalent of [mTBNH][Cl_3OAc] and 5 molar equivalents of water.

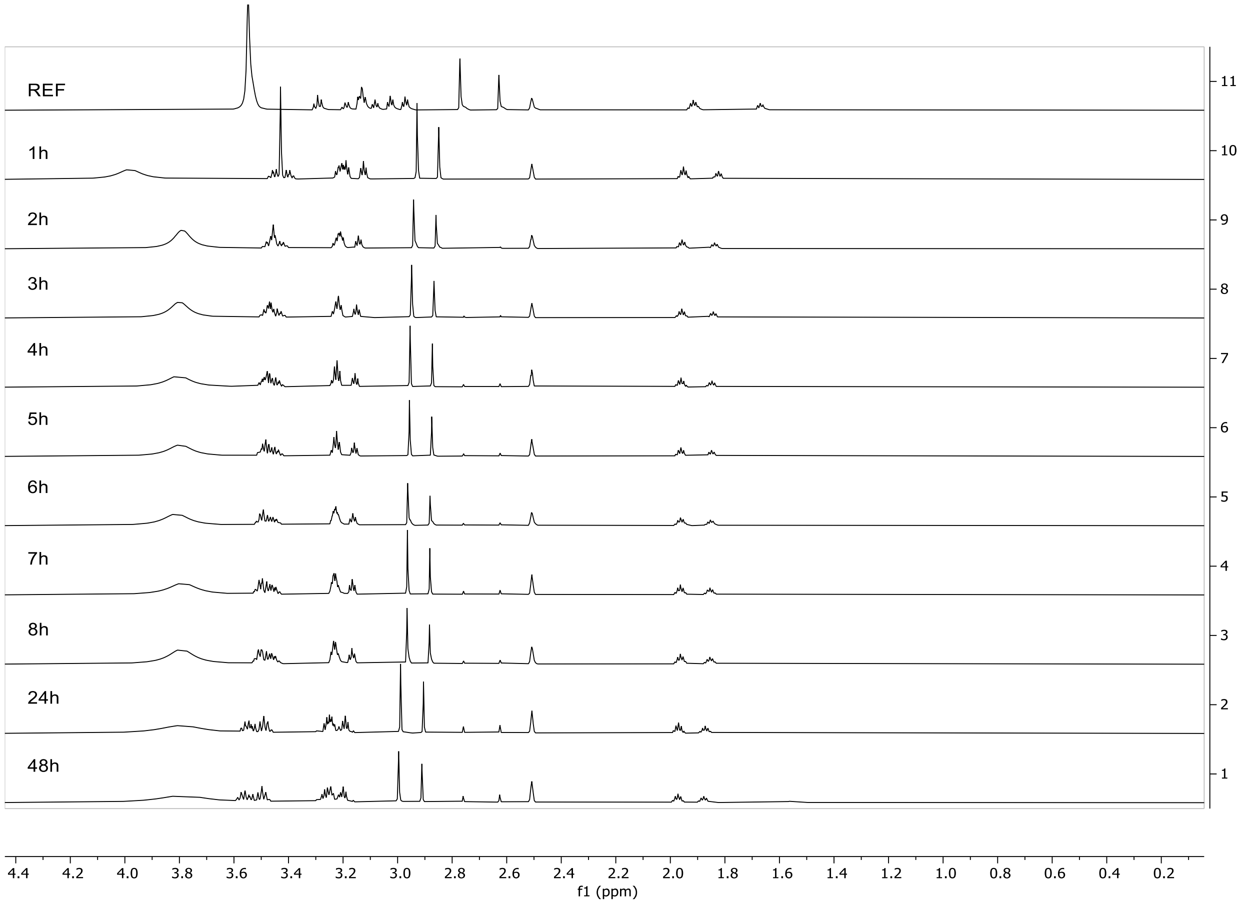

Figure S 100 ^{1}H NMR 1 molar equivalent of [mTBNH][Cl_3OAc] and 5 molar equivalents of water.

5. mTBO + water

Structure	Time	Integral N-Methyl	Integral HP1	Integral HP2	% HP
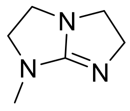	REF	170737000	3060420.00	0.00	0.0
	1h	203223000	718816.00	0.00	0.4
	2h	228050000	2002090.00	0.00	0.9
	3h	143989000	3211590.00	0.00	2.2
	4h	143074000	1905300.00	0.00	1.3
	5h	193733000	2442980.00	0.00	1.2
	6h	183065000	2183950.00	0.00	1.2
	7h	210372000	1724280.00	0.00	0.8
	8h	179768000	945788.00	0.00	0.5
	24h	145114000	2999100.00	0.00	2.0
	48h	165322000	7689370.00	0.00	4.4

Table S101 1 molar equivalent of mTBO and 0.2 molar equivalent of water.

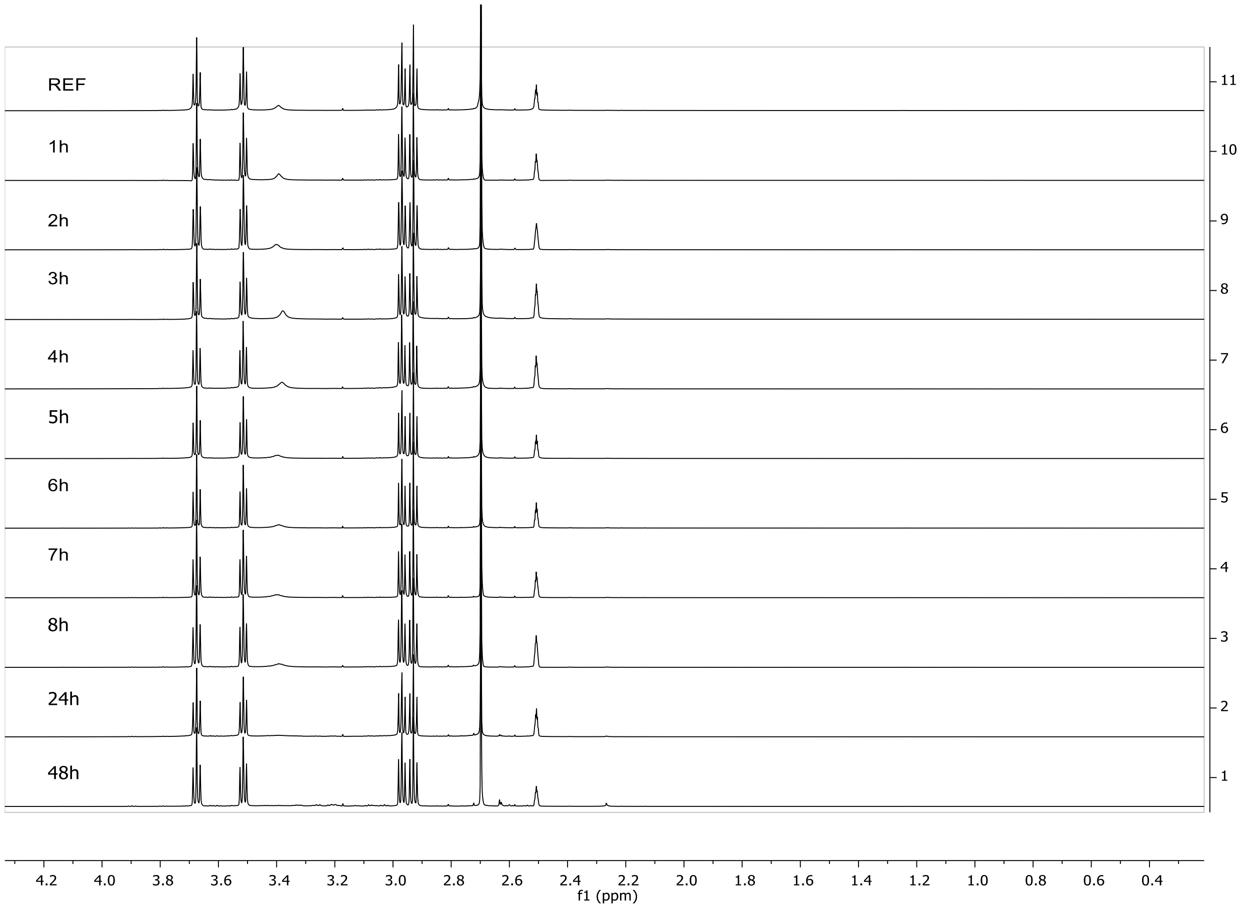

Figure S101 ^{1}H NMR 1 molar equivalent of mTBO and 0.2 molar equivalent of water.

Structure	Time	Integral N-Methyl	Integral HP1	Integral HP2	% HP
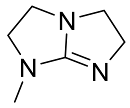	REF	261837000	1349240	0	0.5
	1h	329418000	2590250	0	0.8
	2h	214141000	1093130	0	0.5
	3h	429848000	4398150	0	1.0
	4h	203204000	2150660	0	1.0
	5h	206288000	5014340	0	2.4
	6h	268510000	6689080	0	2.4
	7h	273663000	8046830	0	2.5
	8h	420836000	10935100	0	2.9
	24h	463184000	32868000	0	6.6
	48h	412793000	36566700	0	8.1

Table S102 1 molar equivalent of mTBO and 0.5 molar equivalent of water.

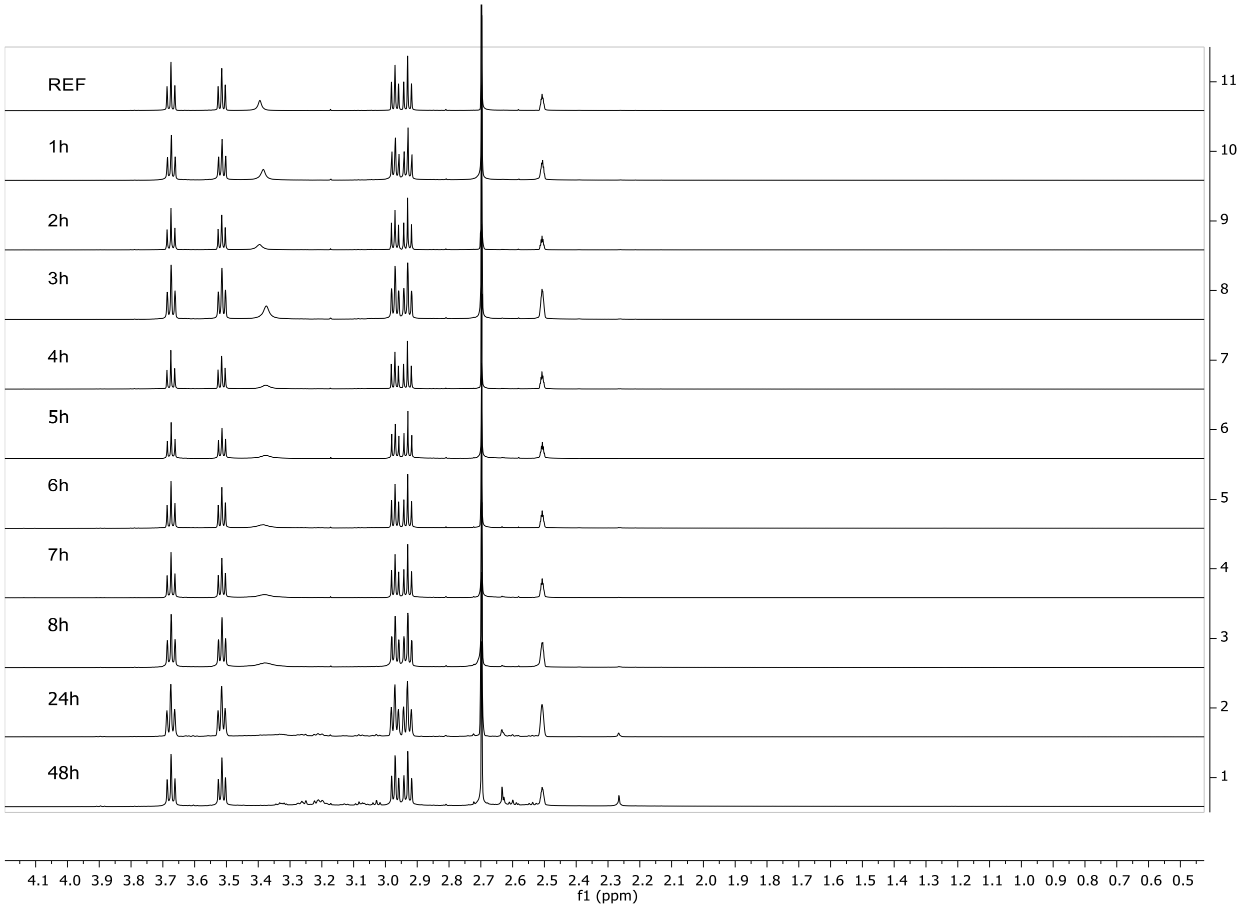

Figure S102 ^{1}H NMR 1 molar equivalent of mTBO and 0.5 molar equivalent of water.

Structure	Time	Integral N-Methyl	Integral HP1	Integral HP2	% HP
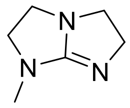	REF	167103000.00	2765370.00	1299810.00	0.0
	1h	162360000.00	3564660.00	1669860.00	3.1
	2h	131474000.00	3284430.00	1670490.00	3.6
	3h	143738000.00	5054900.00	2695360.00	4.1
	4h	184548000.00	4735270.00	3137220.00	5.1
	5h	158068000.00	6618980.00	4091120.00	6.3
	6h	154675000.00	6341340.00	4467220.00	6.5
	7h	125689000.00	7134100.00	4503110.00	8.5
	8h	117445000.00	7168560.00	4380260.00	9.0
	24h	121499000.00	16543900.00	10312700.00	18.1
	48h	118049000.00	31372800.00	21678500.00	31.0

Table S103 1 molar equivalent of mTBO and 1 molar equivalent of water.

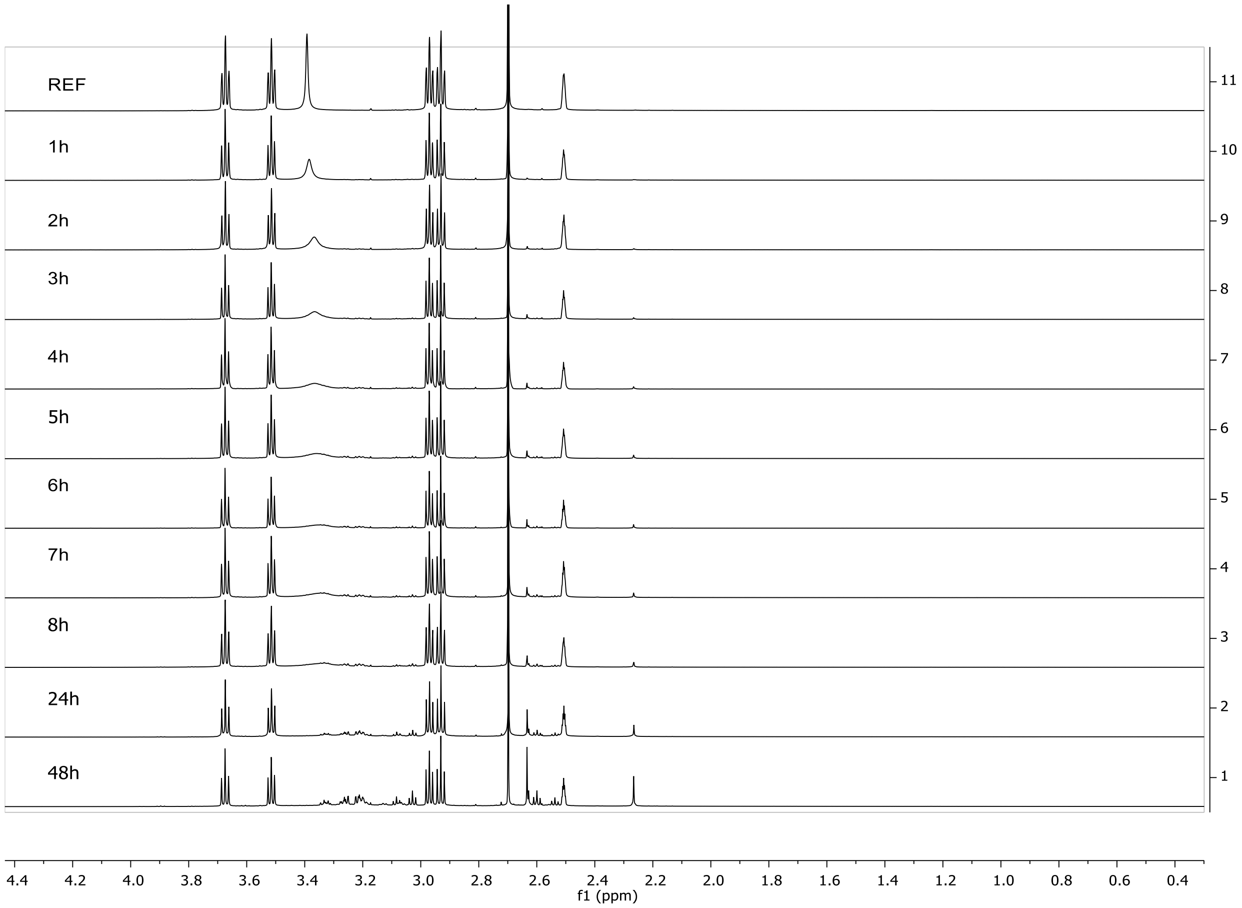

Figure S103 ^{1}H NMR 1 molar equivalent of mTBO and 1 molar equivalent of water.

Structure	Time	Integral N-Methyl	Integral HP1	Integral HP2	% HP
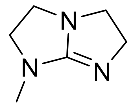	REF	123437000.00	0.00	0.00	0.0
	1h	83702700.00	22357600.00	37885700.00	41.9
	2h	42347300.00	31422000.00	51256100.00	66.1
	3h	24513200.00	34236300.00	54087300.00	78.3
	4h	15977800.00	34110700.00	54691200.00	84.8
	5h	16161100.00	55367600.00	84725700.00	89.7
	6h	13215200.00	58669300.00	90611800.00	91.9
	7h	8689120.00	46008200.00	70308600.00	93.0
	8h	3118540.00	30651400.00	58831200.00	96.6
	24h	3593340.00	54163900.00	77493000.00	96.6
	48h	4573250.00	53574000.00	77786500.00	97.3

Table S104 1 molar equivalent of mTBO and 5 molar equivalents of water.

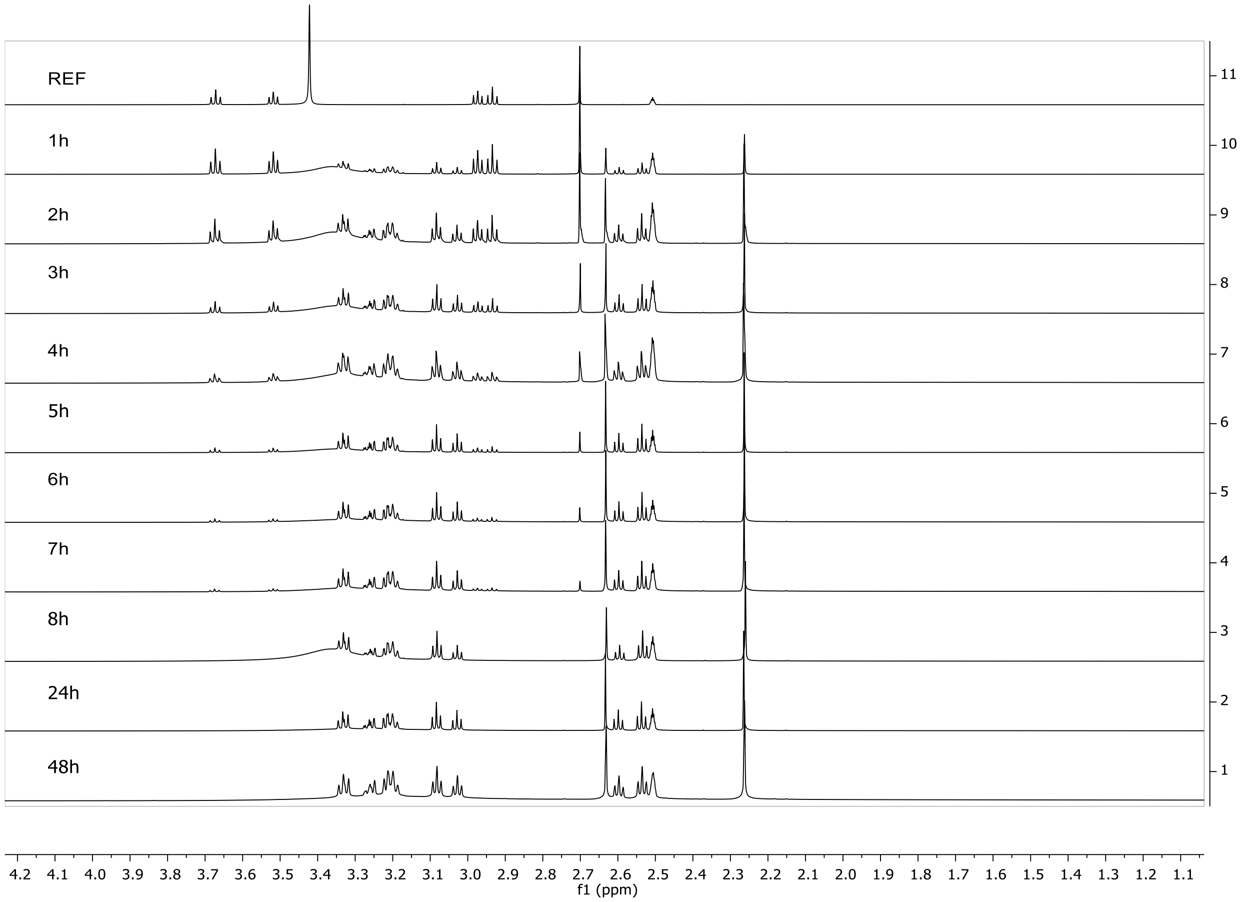

Figure S104 ¹H NMR 1 molar equivalent of mTBO and 5 molar equivalents of water.

Structure	Time	Integral N-Methyl	Integral HP1	Integral HP2	% HP
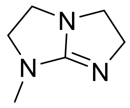	REF	90259500.00	0.00	0.00	0.0
	1h	28403500.00	17177700.00	36330700.00	65.3
	2h	11525600.00	28152000.00	54740400.00	87.8
	3h	6851310.00	29718700.00	57544900.00	92.7
	4h	4609080.00	27211200.00	54414400.00	94.4
	5h	3667500.00	24963600.00	51079200.00	94.5
	6h	4586590.00	31848300.00	64365900.00	94.7
	7h	5210700.00	28876000.00	58293800.00	95.4
	8h	4904740.00	28580400.00	55699300.00	95.4
	24h	3094730.00	29720800.00	58601600.00	96.1
	48h	3666710.00	29977100.00	60816000.00	96.6

Table S105 1 molar equivalent of mTBO and 10 molar equivalents of water.

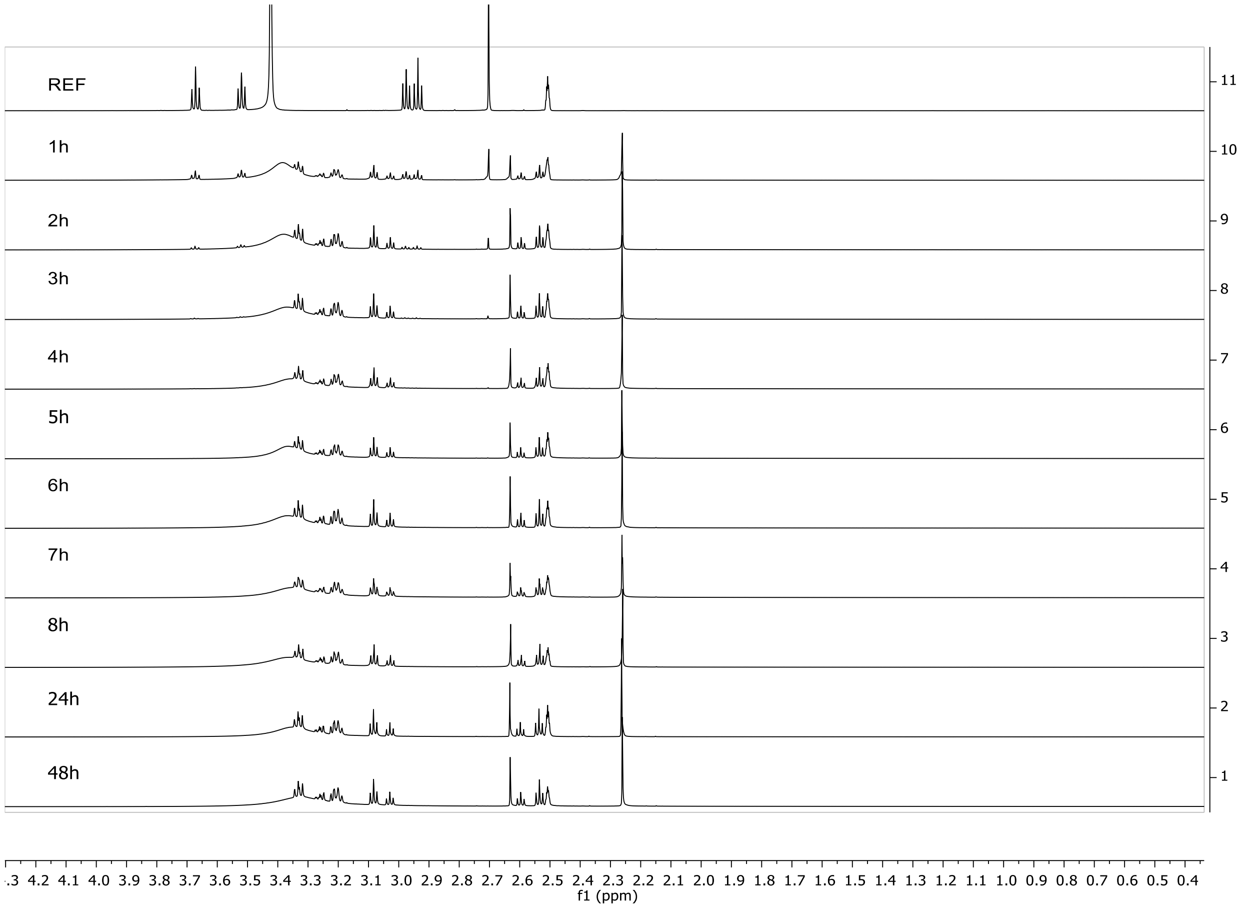

Figure S105 ^{1}H NMR 1 molar equivalent of mTBO and 10 molar equivalents of water.

6. dm39-mTBD + water

Structure	Time	N-Methyl	Integral HP1	Integral HP2	% HP
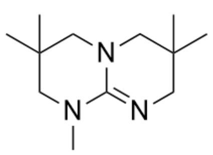	REF	85352178.04	0.00	0.00	0.0
	1h	84532200.00	839102.00	603506.00	1.7
	2h	98110600.00	1098030.00	995408.00	2.1
	3h	96759400.00	1219780.00	1285490.00	2.5
	4h	93399000.00	957765.00	1412110.00	2.5
	5h	76445100.00	1155550.00	1814960.00	3.7
	6h	90863800.00	2029840.00	3699780.00	5.9
	24h	46773300.00	3454810.00	8262060.00	20.0
	48h	325871.00	7416920.00	10095600.00	98.2

Table S106 1 molar equivalent of dm39-mTBD and 1 molar equivalent of water.

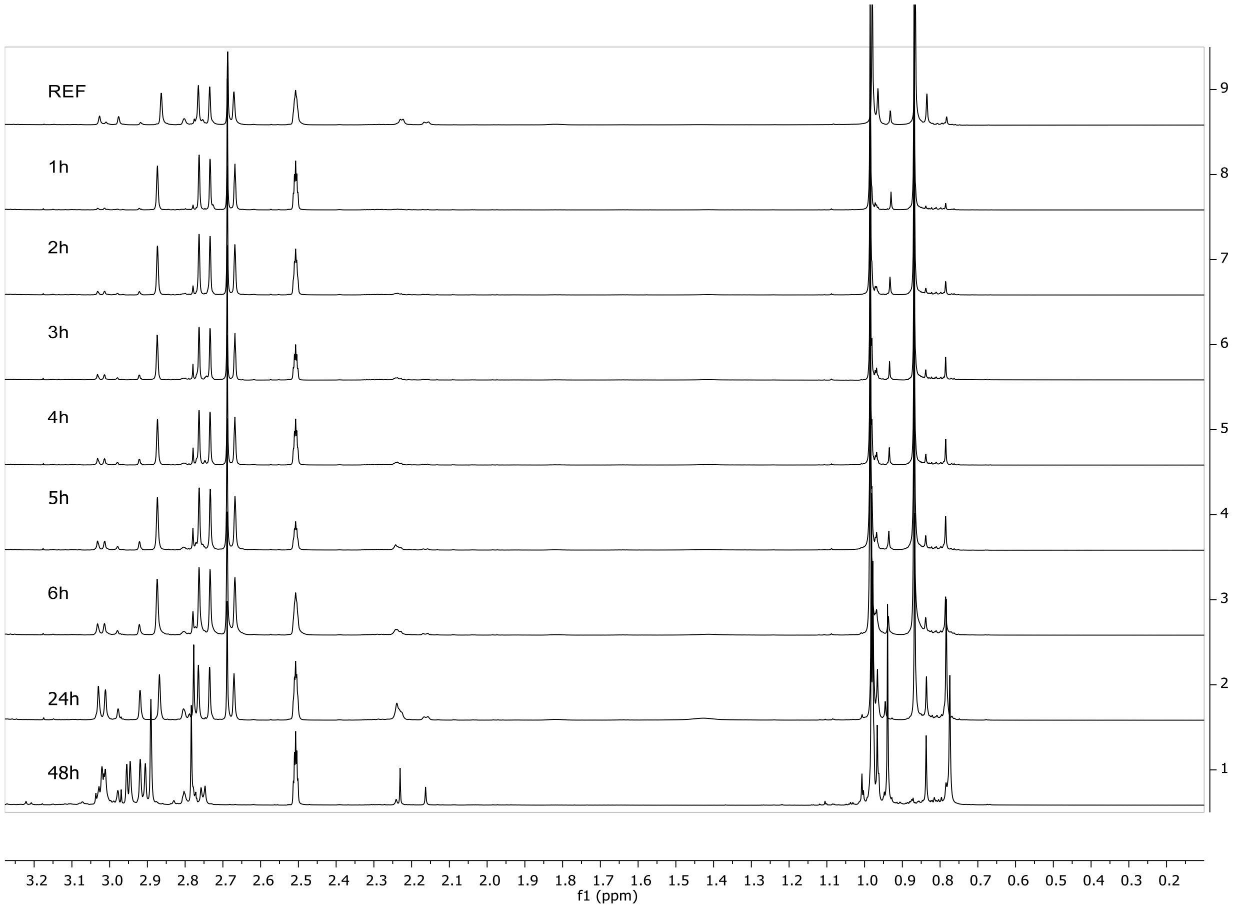

Figure S106 ¹H NMR 1 molar equivalent of dm39-mTBD and 1 molar equivalent of water.

Structure	Time	N-Methyl	Integral HP1	Integral HP2	% HP
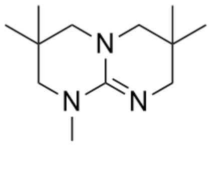	REF	32751400.00	70108.20	25873.30	0.0
	1h	35756500.00	10127900.00	10432500.00	0.3
	2h	14356500.00	20557000.00	23266600.00	36.5
	3h	8210260.00	27552200.00	39683900.00	75.3
	4h	5866660.00	27222400.00	38416900.00	89.1
	5h	4912980.00	31331300.00	45758200.00	91.8
	6h	4285670.00	34932400.00	51141800.00	94.0
	24h	200779.00	33766500.00	49383400.00	95.3
	48h	46737.40	30307600.00	44827900.00	99.8

Table S107 1 molar equivalent of dm39-mTBD and 5 molar equivalents of water.

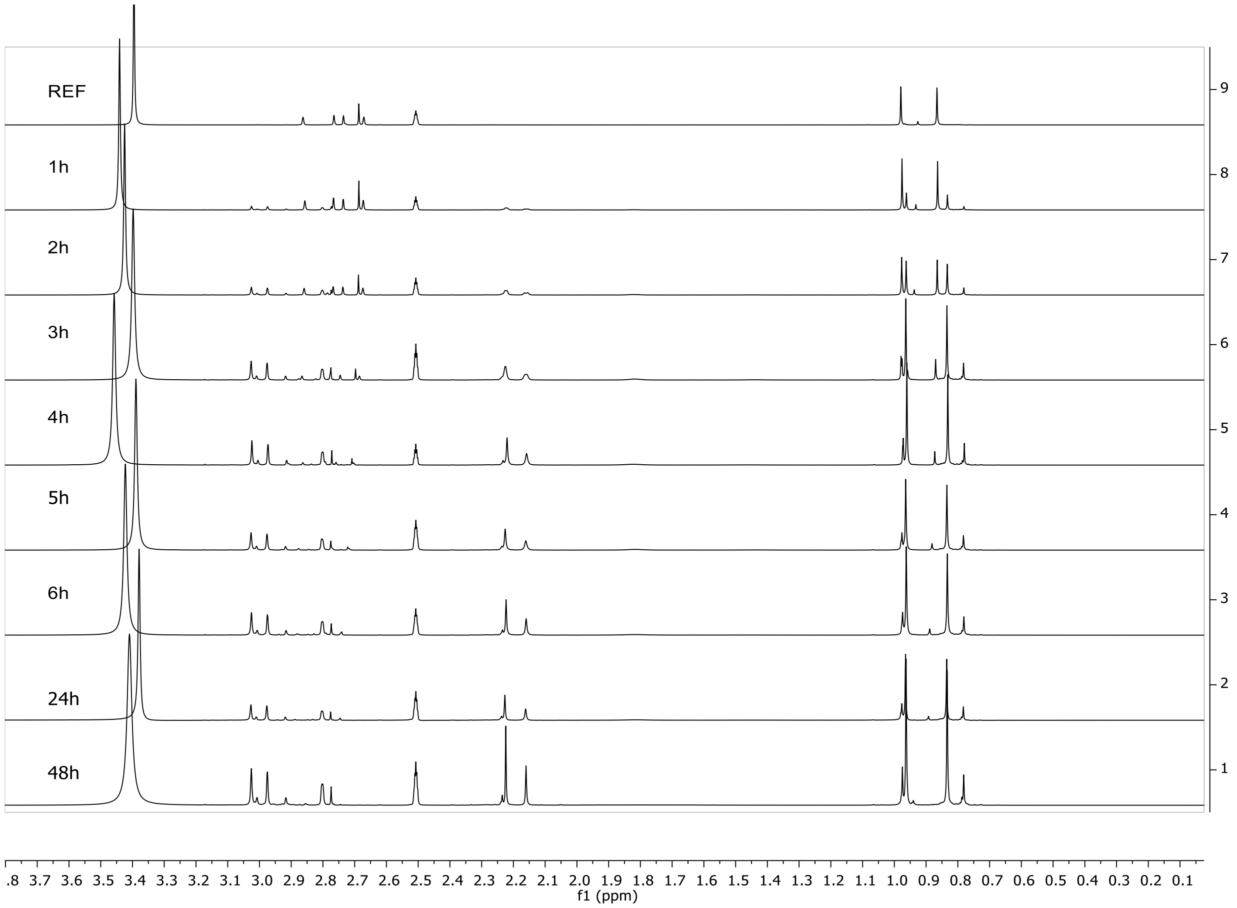

Figure S107 ^{1}H NMR 1 molar equivalent of dm39-mTBD and 5 molar equivalents of water.

Structure	Time	N-Methyl	Integral HP1	Integral HP2	% HP
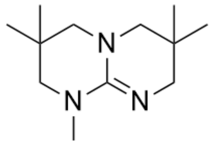	REF	41412400.00	0.00	0.00	0.0
	1h	33629400.00	15152500.00	8657860.00	41.5
	2h	18910000.00	24753600.00	14257300.00	67.4
	3h	1858860.00	28965800.00	16770600.00	96.1
	4h	159613.00	45950200.00	26667300.00	99.8
	5h	114750.00	42735200.00	24485900.00	99.8
	6h	167893	49105900.00	28352300.00	99.8
	24h	87826.50	45154700.00	26062100.00	99.9
	48h	7638.89	45238300.00	24934100.00	100.0

Table S108 1 molar equivalent of dm39-mTBD and 10 molar equivalents of water.

7. [dm39-mTBDH][OAc] + water

Structure	Time	Integral acetate	Integral HP1	Integral HP2	% HP
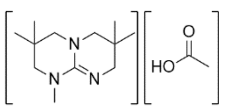	REF	111981000.00	8817.60	16283.80	0.0
	1h	106531000.00	136093.00	533505.00	0.6
	2h	111298000.00	333511.00	857829.00	1.1
	3h	103761000.00	756007.00	1505570.00	2.1
	4h	111628000.00	846729.00	1585400.00	2.1
	5h	102269000.00	973053.00	1639830.00	2.5

	6h	105335000.00	1333910.00	1897750.00	3.0
	24h	105307000.00	4478140.00	6301890.00	9.3
	48h	98609200.00	12351600.00	18392700.00	23.8

Table S109 1 molar equivalent of [dm39-mTBDH][OAc] and 1 molar equivalent of water.

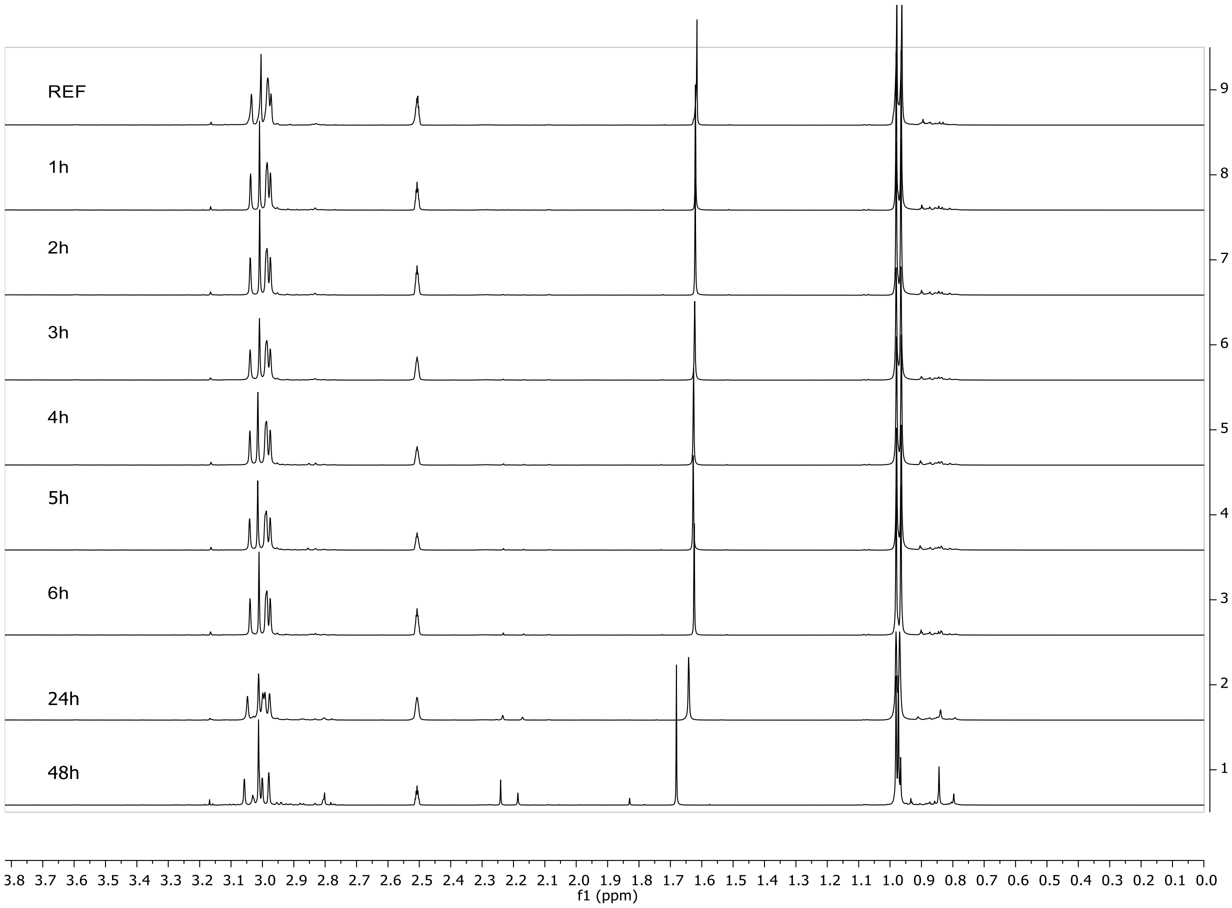

Figure S108 ^{1}H NMR 1 molar equivalent of [dm39-mTBDH][OAc] and 1 molar equivalent of water.

Structure	Time	Integral acetate	Integral HP1	Integral HP2	% HP
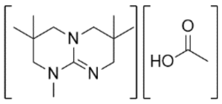	REF	67253200.00	0.00	0.00	0.0
	1h	54472900.00	0.00	0.00	0.0
	2h	63343500.00	0.00	0.00	0.0
	3h	63211700.00	0.00	0.00	0.0
	4h	243528000.00	0.00	0.00	0.0
	5h	60959700.00	0.00	0.00	0.0
	6h	65456600.00	0.00	0.00	0.0
	24h	62143800.00	0.00	0.00	0.0
	48h	95352300.00	0.00	0.00	0.0

Table S110 1 molar equivalent of [dm39-mTBDH][OAc] and 5 molar equivalent of water.

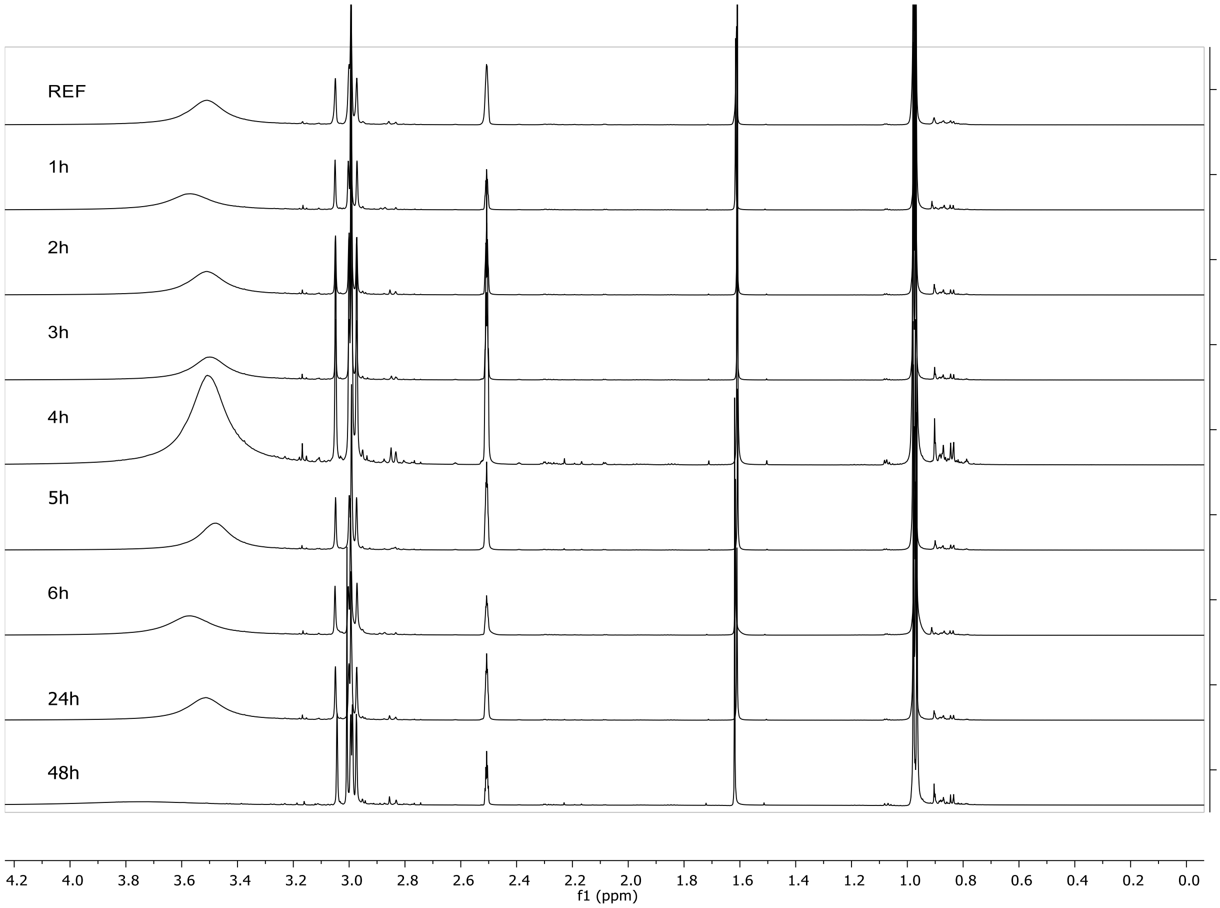

Figure S109 ^{1}H NMR 1 molar equivalent of [dm39-mTBDH][OAc] and 5 molar equivalent of water.

Structure	Time	Integral acetate	Integral HP1	Integral HP2	% HP
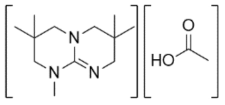	REF	39006400.00	0.00	0.00	0.0
	1h	52842600.00	0.00	0.00	0.0
	2h	46272400.00	0.00	0.00	0.0
	3h	51247100.00	0.00	0.00	0.0
	4h	51325500.00	0.00	0.00	0.0
	5h	49779500.00	0.00	0.00	0.0
	6h	52092100.00	0.00	0.00	0.0
	24h	51698100.00	0.00	0.00	0.0
	48h	55511600.00	0.00	0.00	0.0

Table S111 1 molar equivalent of [dm39-mTBDH][OAc] and 10 molar equivalent of water.

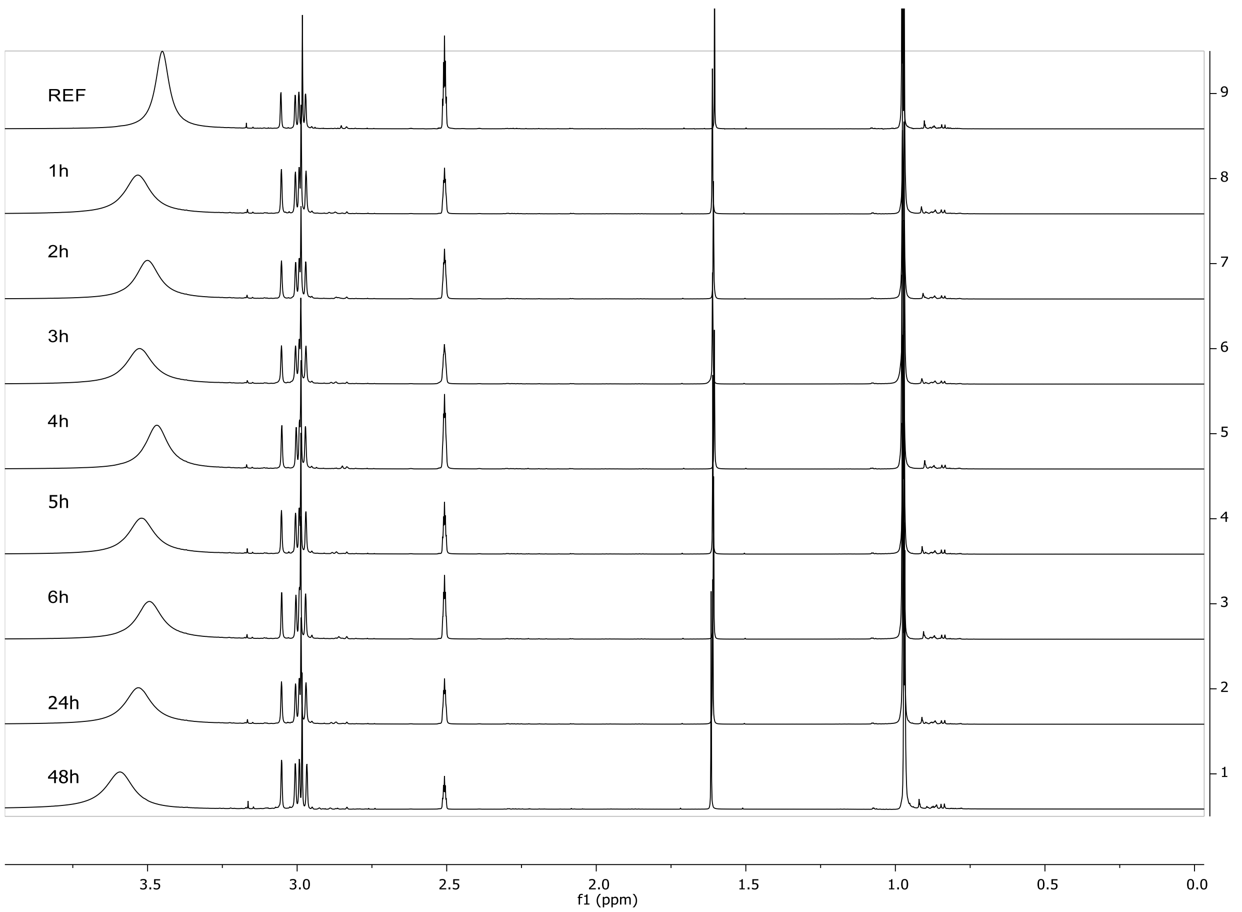

Figure S110 ^{1}H NMR S111 1 molar equivalent of [dm39-mTBDH][OAc] and 10 molar equivalent of water.

8. dm3-m0-mTBD + water

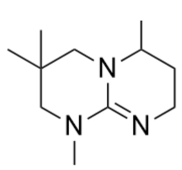

Structure	Time	Integral N-Methyl 1	Integral N-Methyl 2	Integral N Methyl total	Integral HP 1	Integral HP 2	Integral HP Total	% HP
	REF	14898500.00	13780900.00	28679400.00	57607.60	125259.00	182866.60	0.63
	1h	14301200.00	12162800.00	26464000.00	1127190.00	2241110.00	3368300.00	11.29
	2h	14652900.00	11173400.00	25826300.00	1204360.00	3831670.00	5036030.00	16.32
	3h	14436500.00	10082400.00	24518900.00	1225680.00	4981020.00	6206700.00	20.20
	4h	12788900.00	8735110.00	21524010.00	1099400.00	5464980.00	6564380.00	23.37
	5h	13448300.00	7752000.00	21200300.00	1187640.00	6702670.00	7890310.00	27.12
	6h	13267000.00	7232150.00	20499150.00	1244510.00	7824870.00	9069380.00	30.67
	7h	4523300.00	2421690.00	6944990.00	413116.00	2936660.00	3349776.00	32.54
	8h	24580700.00	12446400.00	37027100.00	2549210.00	18013600.00	20562810.00	35.71
	24h	13324600.00	3388010.00	16712610.00	1920270.00	18301700.00	20221970.00	54.75
	48h	36018700.00	17622500.00	53641200.00	6294600.00	57854900.00	64149500.00	54.46

Table S112 1 molar equivalent of dm3-m0-mTBD and 1 molar equivalent of water.

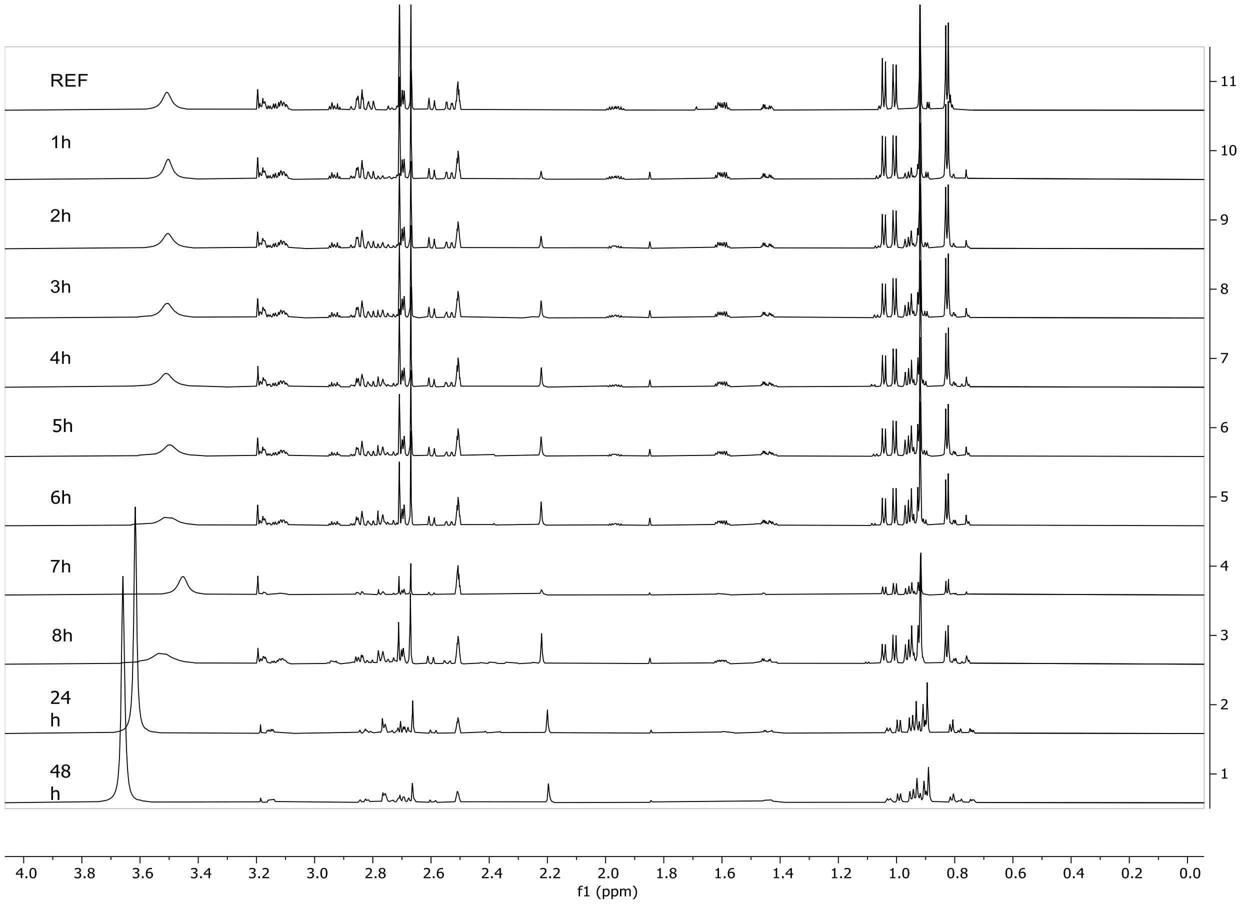

Figure S111 ¹H NMR 1 molar equivalent of dm3-m0-mTBD and 1 molar equivalent of water.

Structure	Time	Integral N-Methyl 1	Integral N-Methyl 2	Integral N Methyl total	Integral HP 1	Integral HP 2	Integral HP Total	% HP
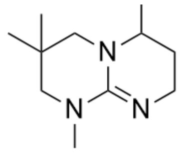	REF	115342000.00	109817000.00	225159000.00	1127840.00	1414690.00	2542530.00	1.12
	1h	68230200.00	99940400.00	168170600.00	83118400.00	11944600.00	95063000.00	36.11
	2h	43778300.00	89628500.00	133406800.00	129796000.00	12970200.00	142766200.00	51.69
	3h	34723300.00	97113400.00	131836700.00	180157000.00	15957600.00	196114600.00	59.80
	4h	20220300.00	70149800.00	90370100.00	151507000.00	12654500.00	164161500.00	64.50
	5h	15923800.00	68477300.00	84401100.00	166067000.00	12865600.00	178932600.00	67.95
	6h	10831600.00	56583900.00	67415500.00	156140000.00	11638900.00	167778900.00	71.34
	7h	8185200.00	46098800.00	54284000.00	148456000.00	10892800.00	159348800.00	74.59
	8h	31279400.00	4828000.00	36107400.00	178851000.00	13111200.00	191962200.00	84.17
	24h	9428020.00	18021100.00	27449120.00	154467000.00	13605400.00	168072400.00	85.96
	48h	1776160.00	889806.00	2665966.00	91933500.00	6760360.00	98693860.00	97.37

Table S113 1 molar equivalent of dm3-m0-mTBD and 5 molar equivalents of water.

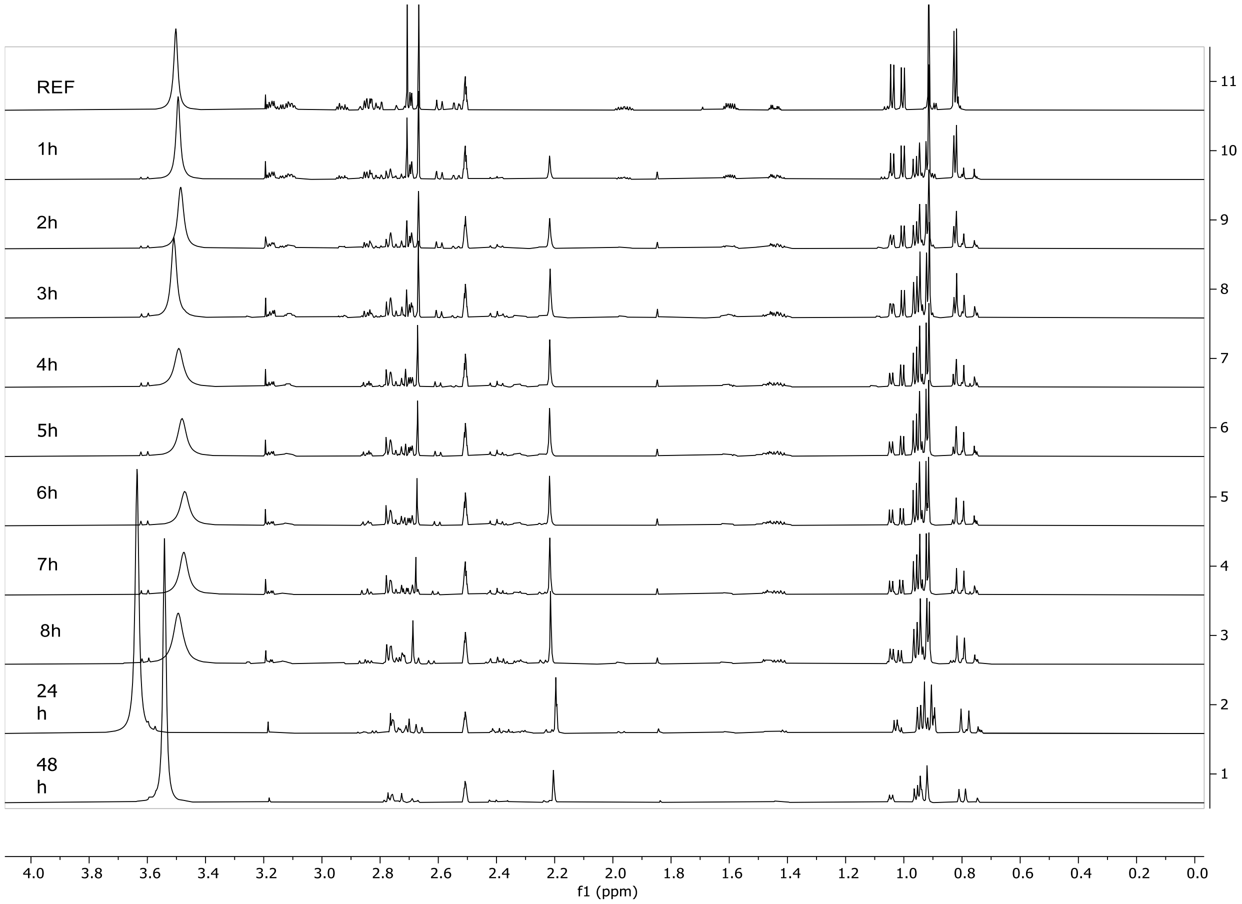

Figure S112 ^{1}H NMR 1 molar equivalent of dm3-m0-mTBD and 5 molar equivalents of water.

Structure	Time	Integral N-Methyl 1	Integral N-Methyl 2	Integral N Methyl total	Integral HP	Integral HP total	% HP
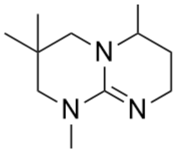	REF	30244000.00	30662400.00	60906400.00	225605.00	225605.00	0.37
	1h	8127670.00	16358000.00	24485670.00	24995500.00	24995500.00	50.52
	2h	1797800.00	2245380.00	4043180.00	33878500.00	33878500.00	89.34
	3h	19295.10	273456.00	292751.10	36654300.00	36654300.00	99.21
	4h	34513.50	334177.00	368690.50	46299200.00	46299200.00	99.21
	5h	59373.10	225933.00	285306.10	39845600.00	39845600.00	99.29
	6h	20292.70	145397.00	165689.70	42158300.00	42158300.00	99.61
	7h	8426.35	21129.40	29555.75	17966200.00	17966200.00	99.84
	24h	4270.05	4405.02	8675.07	13918300.00	13918300.00	99.94
	48h	0.00	0.00	0.00	55119800.00	55119800.00	100.00

Table S114 1 molar equivalent of dm3-m0-mTBD and 10 molar equivalent of water.

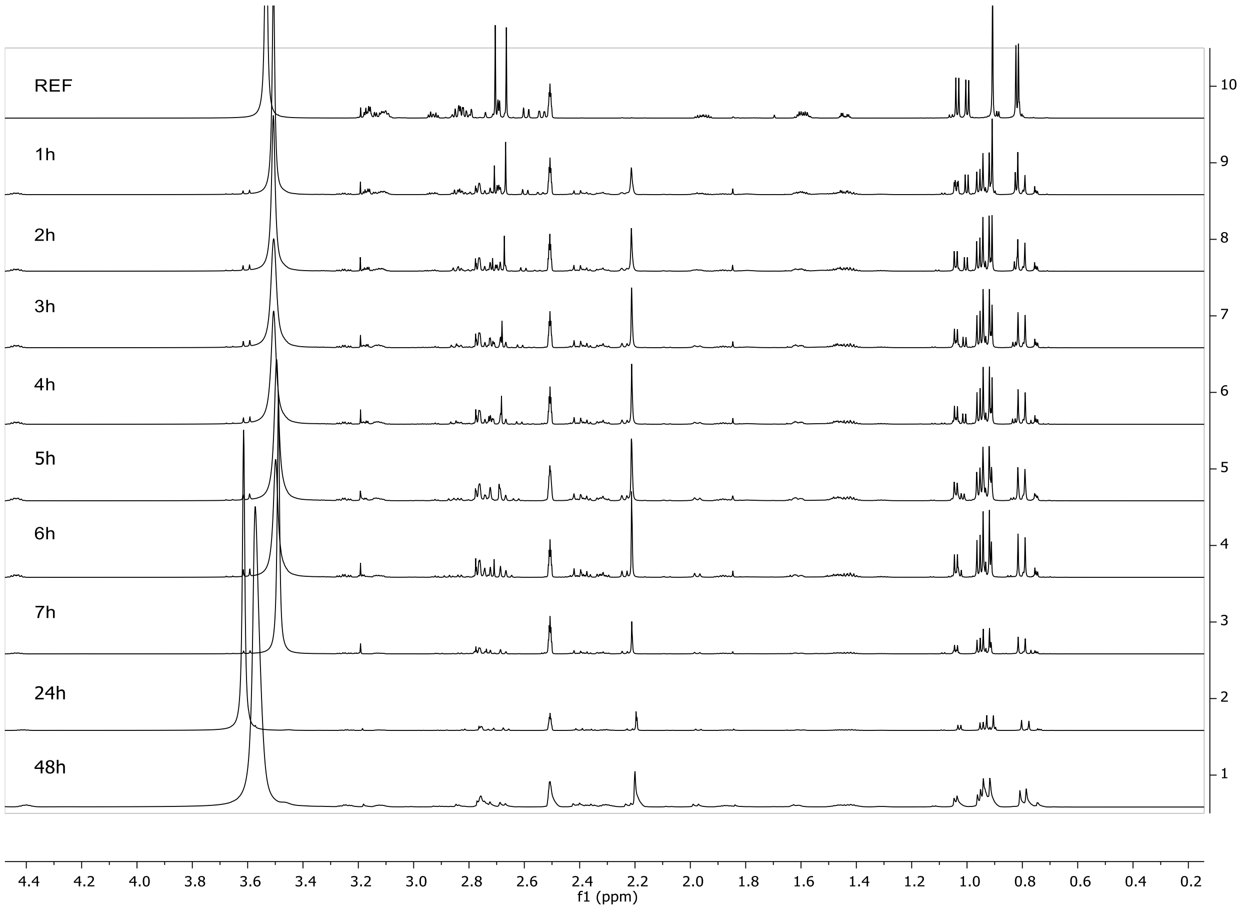

Figure S113 ^{1}H NMR 1 molar equivalent of dm3-m0-mTBD and 10 molar equivalent of water.

9. [dm3-m0-mTBDH][OAc] + water

Structure	Time	Integral N-Methyl 1	Integral N-Methyl 2	Integral N Methyl total	Integral HP 1	Integral HP 2	Integral HP Total	% HP
	REF	26980100.00	0.00	26980100.00	0.00	0.00	0.00	0.00
	1h	22540200.00	0.00	22540200.00	0.00	0.00	0.00	0.00
	2h	23601200.00	0.00	23601200.00	0.00	0.00	0.00	0.00
	3h	23832900.00	0.00	23832900.00	0.00	0.00	0.00	0.00
	4h	23779200.00	0.00	23779200.00	0.00	0.00	0.00	0.00
	5h	27657800.00	0.00	27657800.00	0.00	0.00	0.00	0.00
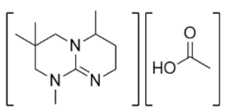	6h	24582200.00	0.00	24582200.00	0.00	0.00	0.00	0.00
	7h	8800900.00	0.00	8800900.00	0.00	0.00	0.00	0.00
	8h	51566600.00	0.00	51566600.00	0.00	0.00	0.00	0.00
	24h	34067200.00	0.00	34067200.00	0.00	0.00	0.00	0.00
	48h	32571300.00	0.00	32571300.00	0.00	0.00	0.00	0.00

Table S115 1 molar equivalent of [dm3-m0-mTBDH][OAc] and 1 molar equivalent of water.

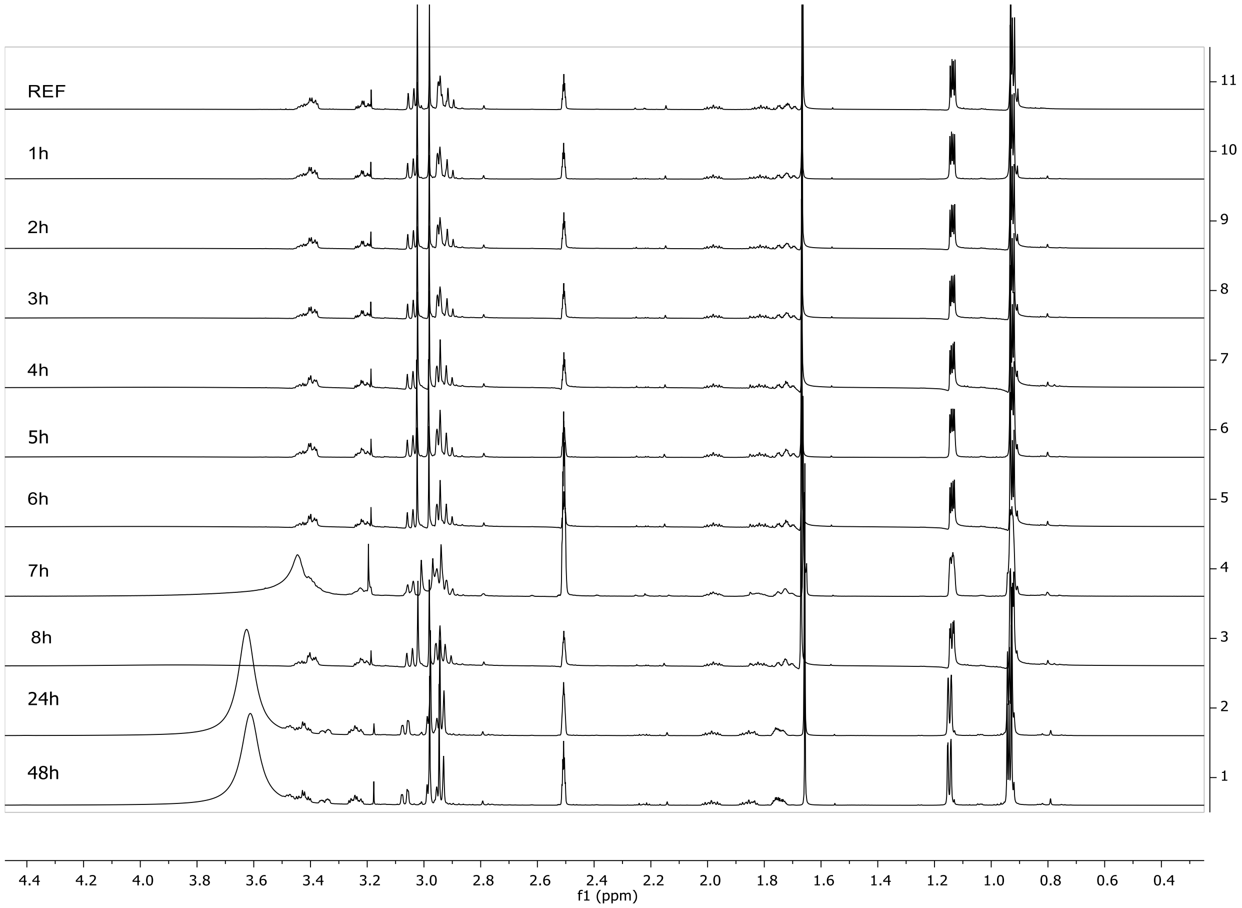

Figure S114 ^{1}H NMR 1 molar equivalent of [dm3-m0-mTBDH][OAc] and 1 molar equivalent of water.

Structure	Time	Integral N-Methyl 1	Integral N-Methyl 2	Integral N Methyl total	Integral HP 1	Integral HP 2	Integral HP Total	% HP
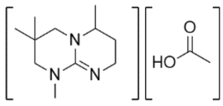	REF	19652000.00	0.00	19652000.00	0.00	0.00	0.00	0.00
	1h	19547100.00	0.00	19547100.00	0.00	0.00	0.00	0.00
	2h	19781600.00	0.00	19781600.00	0.00	0.00	0.00	0.00
	3h	27109000.00	0.00	27109000.00	0.00	0.00	0.00	0.00
	4h	20017300.00	0.00	20017300.00	0.00	0.00	0.00	0.00
	5h	20309100.00	0.00	20309100.00	0.00	0.00	0.00	0.00
	6h	18986300.00	0.00	18986300.00	0.00	0.00	0.00	0.00
	7h	17090300.00	0.00	17090300.00	0.00	0.00	0.00	0.00
	8h	43550900.00	0.00	43550900.00	0.00	0.00	0.00	0.00
	48h	29184100.00	0.00	29184100.00	0.00	0.00	0.00	0.00

Table S116 1 molar equivalent of [dm3-m0-mTBDH][OAc] and 5 molar equivalent of water.

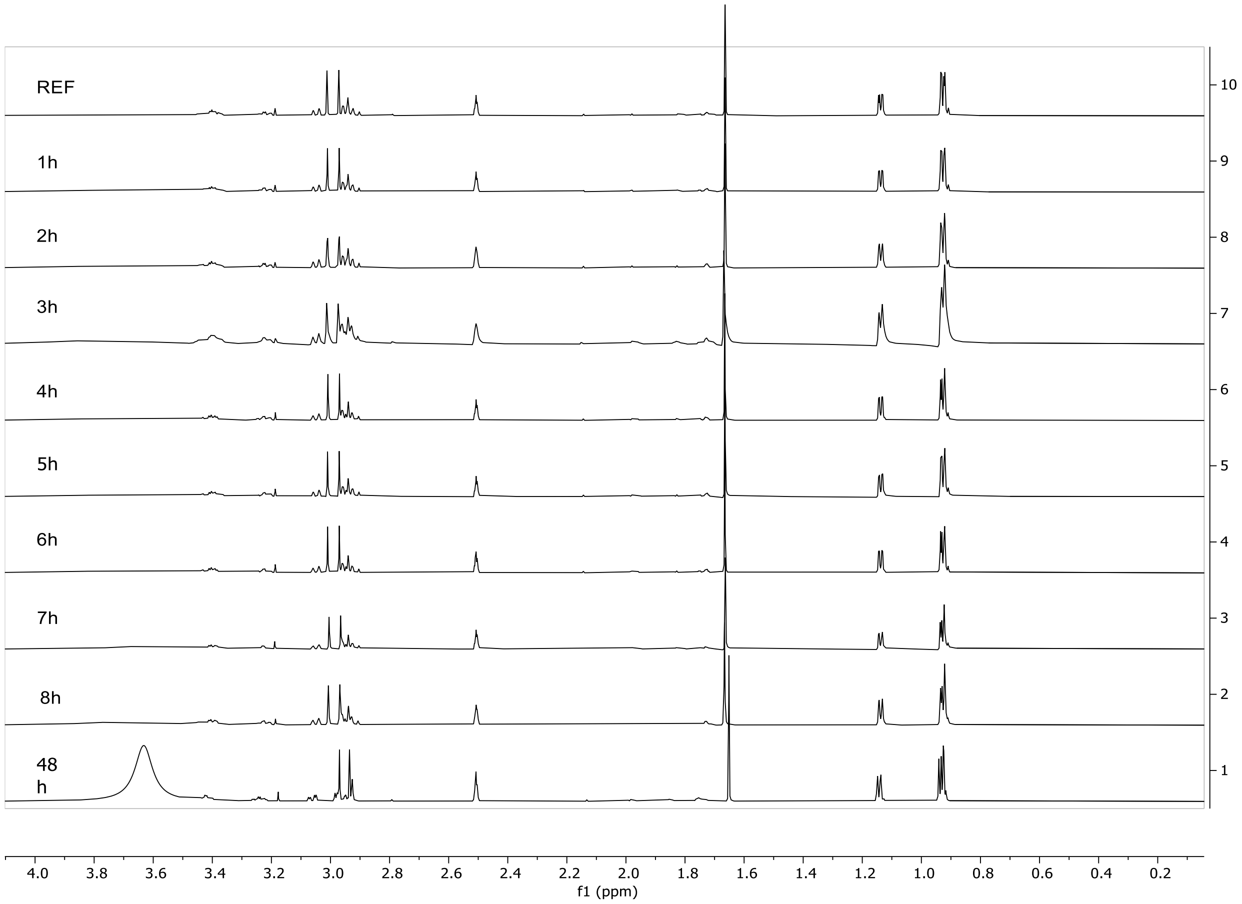

Figure S115 ^{1}H NMR 1 molar equivalent of [dm3-m0-mTBDH][OAc] and 5 molar equivalent of water.

Structure	Time	integral N-Methyl 1	integral N-Methyl 2	integral N Methyl total	integral HP 1	integral HP 2	integral HP Total	% HP
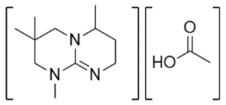	REF	16407500.00	0.00	16407500.00	0.00	0.00	0.00	0.00
	1h	15992500.00	0.00	15992500.00	0.00	0.00	0.00	0.00
	2h	16274100.00	0.00	16274100.00	0.00	0.00	0.00	0.00
	3h	18253000.00	0.00	18253000.00	0.00	0.00	0.00	0.00
	4h	16809100.00	0.00	16809100.00	0.00	0.00	0.00	0.00
	5h	17126400.00	0.00	17126400.00	0.00	0.00	0.00	0.00
	6h	17291200.00	0.00	17291200.00	0.00	0.00	0.00	0.00
	7h	11249700.00	0.00	11249700.00	0.00	0.00	0.00	0.00
	8h	34548300.00	0.00	34548300.00	0.00	0.00	0.00	0.00
	48h	24899100.00	0.00	24899100.00	0.00	0.00	0.00	0.00

Table S117 1 molar equivalent of [dm3-m0-mTBDH][OAc] and 10 molar equivalent of water.

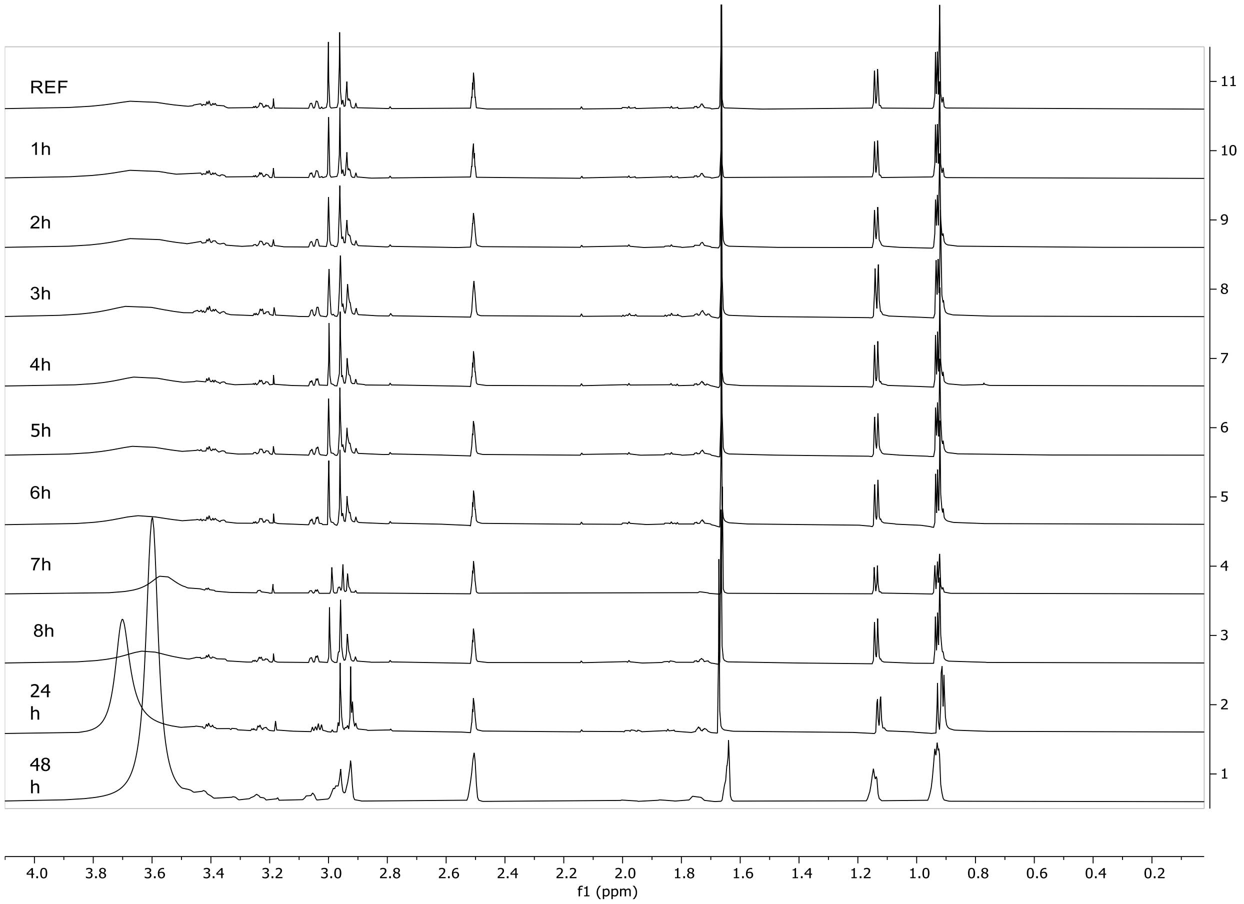

Figure S116 ^{1}H NMR 1 molar equivalent of [dm3-m0-mTBDH][OAc] and 10 molar equivalent of water.

10. dm3-mTBD + water

Structure	HT_28	Integral N-Methyl 1	Integral N-Methyl 2	Integral N Methyl total	Integral HP 1	Integral HP 2	Integral HP Total	% HP
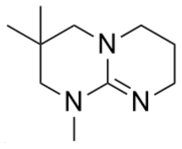	REF	102945000.00	125448000.00	228393000.00	1035700.00	299898.00	1335598.00	0.00
	1h	107742000.00	131671000.00	239413000.00	4228230.00	3176190.00	7404420.00	3.00
	2h	105342000.00	128846000.00	234188000.00	8581860.00	7039660.00	15621520.00	6.25
	3h	105944000.00	130043000.00	235987000.00	12548500.00	10608800.00	23157300.00	8.94
	4h	103859000.00	127291000.00	231150000.00	14967200.00	12776200.00	27743400.00	10.72
	5h	107420000.00	132032000.00	239452000.00	17972600.00	15376200.00	33348800.00	12.22
	6h	99294000.00	121986000.00	221280000.00	19131500.00	16488900.00	35620400.00	13.87
	7h	92150700.00	115004000.00	207154700.00	19763100.00	17496400.00	37259500.00	15.24
	8h	88418700.00	108903000.00	197321700.00	19081000.00	17403900.00	36484900.00	15.60
	24h	21975400.00	26751900.00	48727300.00	8765730.00	6195100.00	14960830.00	23.49
	48h	20222500.00	25135200.00	45357700.00	8982610.00	7477050.00	16459660.00	26.63

Table S118 1 molar equivalent of dm3-mTBD and 0.2 molar equivalent of water.

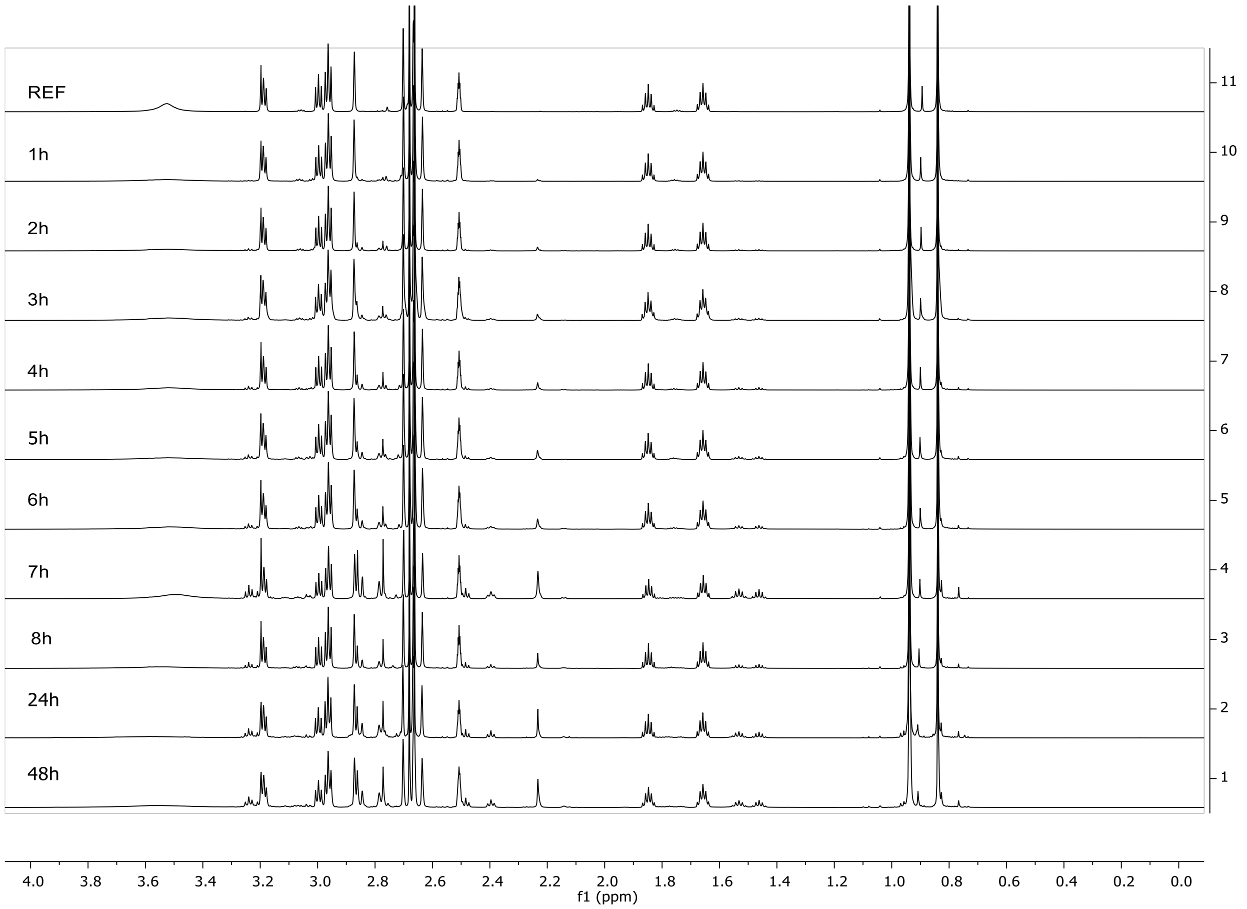

Figure S117 ¹H NMR molar equivalent of dm3-mTBD and 0.2 molar equivalent of water.

Structure	Time	Integral N-Methyl 1	Integral N-Methyl 2	Integral N Methyl total	Integral HP 1	Integral HP 2	Integral HP Total	% HP
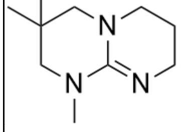	REF	102672000.00	126118000.00	228790000.00	960233.00	249511.00	1209744.00	0.00
	1h	105252000.00	132918000.00	238170000.00	13286300.00	10001600.00	23287900.00	8.91
	2h	88629900.00	114898000.00	203527900.00	23973300.00	17891900.00	41865200.00	17.06
	3h	74875700.00	98428200.00	173303900.00	30208900.00	22275900.00	52484800.00	23.25
	4h	75909500.00	102195000.00	178104500.00	37009600.00	27843600.00	64853200.00	26.69
	5h	79714100.00	108788000.00	188502100.00	44674300.00	34080600.00	78754900.00	29.47
	6h	74578600.00	103400000.00	177978600.00	49011500.00	37444000.00	86455500.00	32.69
	7h	72288300.00	97234500.00	169522800.00	50231600.00	37366200.00	87597800.00	34.07
	8h	65936500.00	93382400.00	159318900.00	48781000.00	39823000.00	88604000.00	35.74
	24h	12197500.00	18878100.00	31075600.00	17705100.00	13682600.00	31387700.00	50.25
	48h	10893800.00	17993900.00	28887700.00	19453400.00	15770800.00	35224200.00	54.94

Table S119 1 molar equivalent of dm3-mTBD and 0.5 molar equivalent of water.

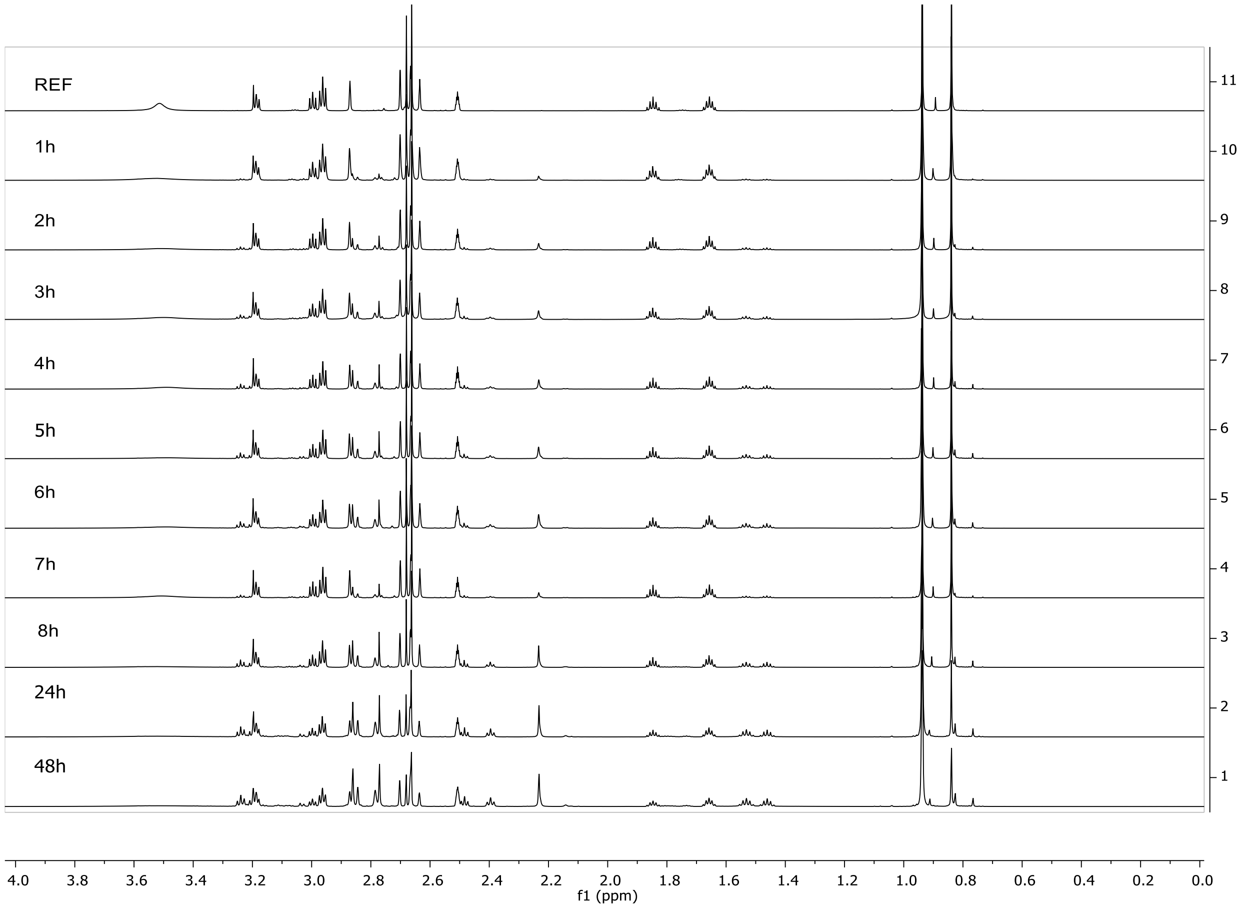

Figure S118 ^{1}H NMR 1 molar equivalent of dm3-mTBD and 0.5 molar equivalent of water.

Structure	Time	Integral N-Methyl 1	Integral N-Methyl 2	Integral N Methyl total	Integral HP 1	Integral HP 2	Integral HP Total	% HP
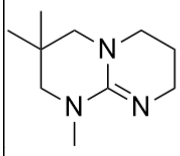	REF	100924000.00	125640000.00	226564000.00	920595.00	261539.00	1182134.00	0.00
	1h	87629300.00	117399000.00	205028300.00	22599100.00	15500500.00	38099600.00	15.67
	2h	72385800.00	105215000.00	177600800.00	40412400.00	28620600.00	69033000.00	27.99
	3h	58567700.00	89212300.00	147780000.00	50302700.00	35094300.00	85397000.00	36.62
	4h	53031200.00	84351700.00	137382900.00	59296700.00	41033000.00	100329700.00	42.21
	5h	49626300.00	80737800.00	130364100.00	65147800.00	45288300.00	110436100.00	45.86
	6h	45653100.00	77802100.00	123455200.00	72242800.00	51284900.00	123527700.00	50.01
	7h	37448500.00	65500600.00	102949100.00	66725500.00	47136600.00	113862100.00	52.52
	8h	41357100.00	74069300.00	115426400.00	75511300.00	58405400.00	133916700.00	53.71
	24h	8105000.00	16145500.00	24250500.00	22501200.00	17351700.00	39852900.00	62.17
	48h	7573510.00	16137800.00	23711310.00	23179500.00	19095900.00	42275400.00	64.07

Table S120 1 molar equivalent of dm3-mTBD and 1 molar equivalent of water.

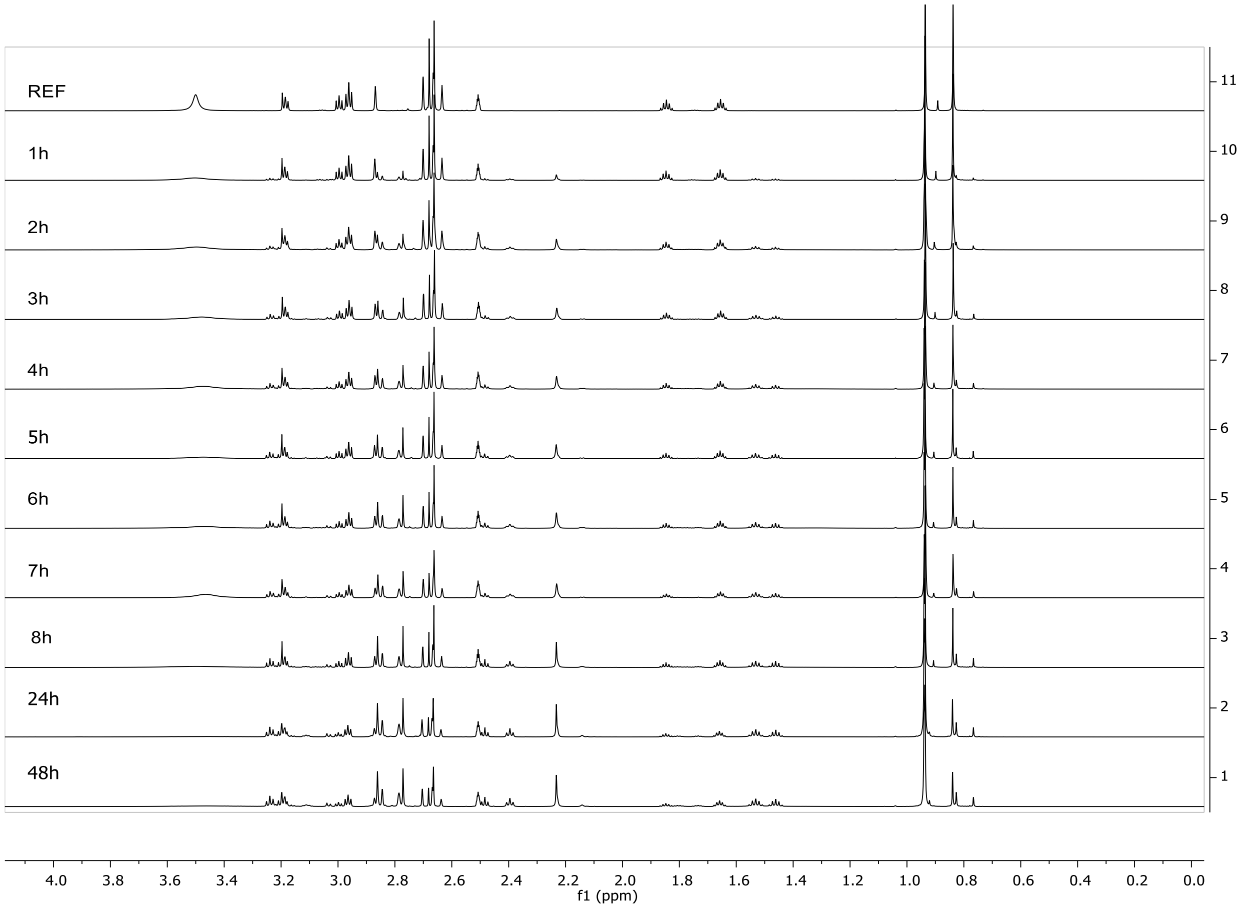

Figure S119 ^{1}H NMR 1 molar equivalent of dm3-mTBD and 1 molar equivalent of water.

Structure	Time	Integral N-Methyl 1	Integral N-Methyl 2	Integral N Methyl total	Integral HP 1	Integral HP 2	Integral HP Total	% HP
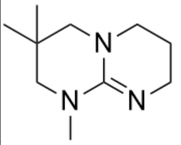	REF	72135100.00	88168000.00	160303100.00	666998.00	261539.00	928537.00	0.00
	1h	26906400.00	30382500.00	57288900.00	40977700.00	15500500.00	56478200.00	49.64
	2h	17817700.00	21382100.00	39199800.00	49732200.00	28620600.00	78352800.00	66.65
	3h	12880500.00	18845900.00	31726400.00	51974400.00	35094300.00	87068700.00	73.29
	4h	9877410.00	17749500.00	27626910.00	53182300.00	41033000.00	94215300.00	77.33
	5h	9549180.00	14391400.00	23940580.00	49934900.00	45288300.00	95223200.00	79.91
	6h	10630100.00	10525800.00	21155900.00	53055600.00	51284900.00	104340500.00	83.14
	7h	10991200.00	7438950.00	18430150.00	51768500.00	47136600.00	98905100.00	84.29
	8h	9276130.00	5362660.00	14638790.00	42624900.00	58405400.00	101030300.00	87.34
	24h	2483980.00	2288210.00	4772190.00	18172200.00	17351700.00	35523900.00	88.16
	48h	1875060.00	1582470.00	3457530.00	16797900.00	19095900.00	35893800.00	91.21

Table S121 1 molar equivalent of dm3-mTBD and 5 molar equivalents of water.

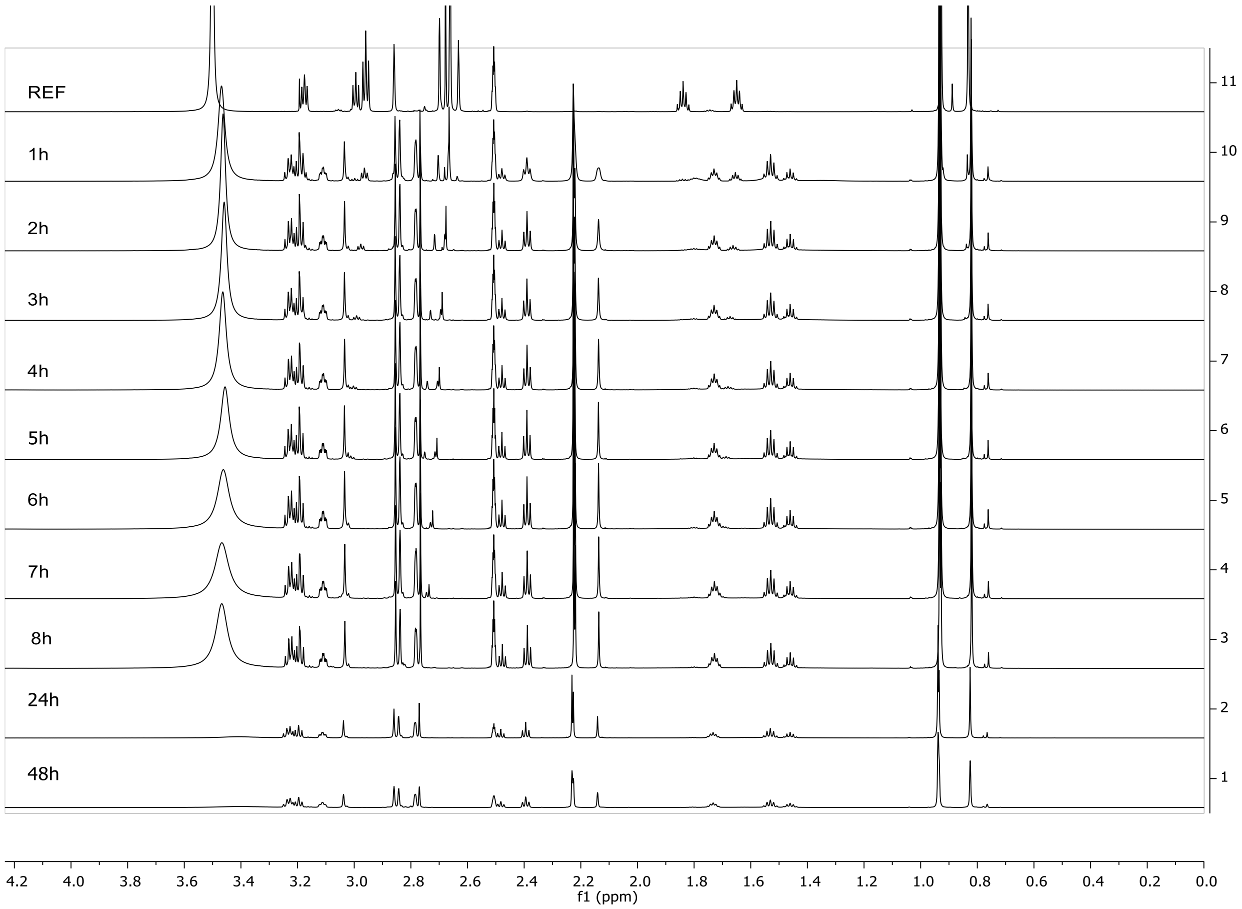

Figure S120 ^{1}H NMR 1 molar equivalent of dm3-mTBD and 5 molar equivalents of water.

Structure	Time	Integral N-Methyl 1	Integral N-Methyl 2	Integral N Methyl total	Integral HP 1	Integral HP 2	Integral HP Total	% HP
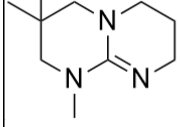	REF	55026600.00	67486300.00	122512900.00	2387960.00	666901.00	3054861.00	0.00
	1h	15162100.00	12259300.00	27421400.00	64246300.00	30049600.00	94295900.00	77.47
	2h	8812810.00	6359280.00	15172090.00	58528900.00	27380800.00	85909700.00	84.99
	3h	7609180.00	4844670.00	12453850.00	61692400.00	28804100.00	90496500.00	87.90
	4h	6100070.00	2808780.00	8908850.00	59689500.00	27884000.00	87573500.00	90.77
	5h	6292480.00	1735780.00	8028260.00	60614300.00	27362600.00	87976900.00	91.64
	6h	5573750.00	1739100.00	7312850.00	61177100.00	28569900.00	89747000.00	92.47
	7h	4903500.00	1922700.00	6826200.00	66342300.00	31035700.00	97378000.00	93.45
	8h	3303460.00	1140690.00	4444150.00	60518300.00	27695500.00	88213800.00	95.20
	24h	954567.00	286748.00	1241315.00	19334200.00	8518080.00	27852280.00	95.73
	48h	892530.00	274600.00	1167130.00	20040900.00	8799080.00	28839980.00	96.11

Table S122 1 molar equivalent of dm3-mTBD and 10 molar equivalents of water.

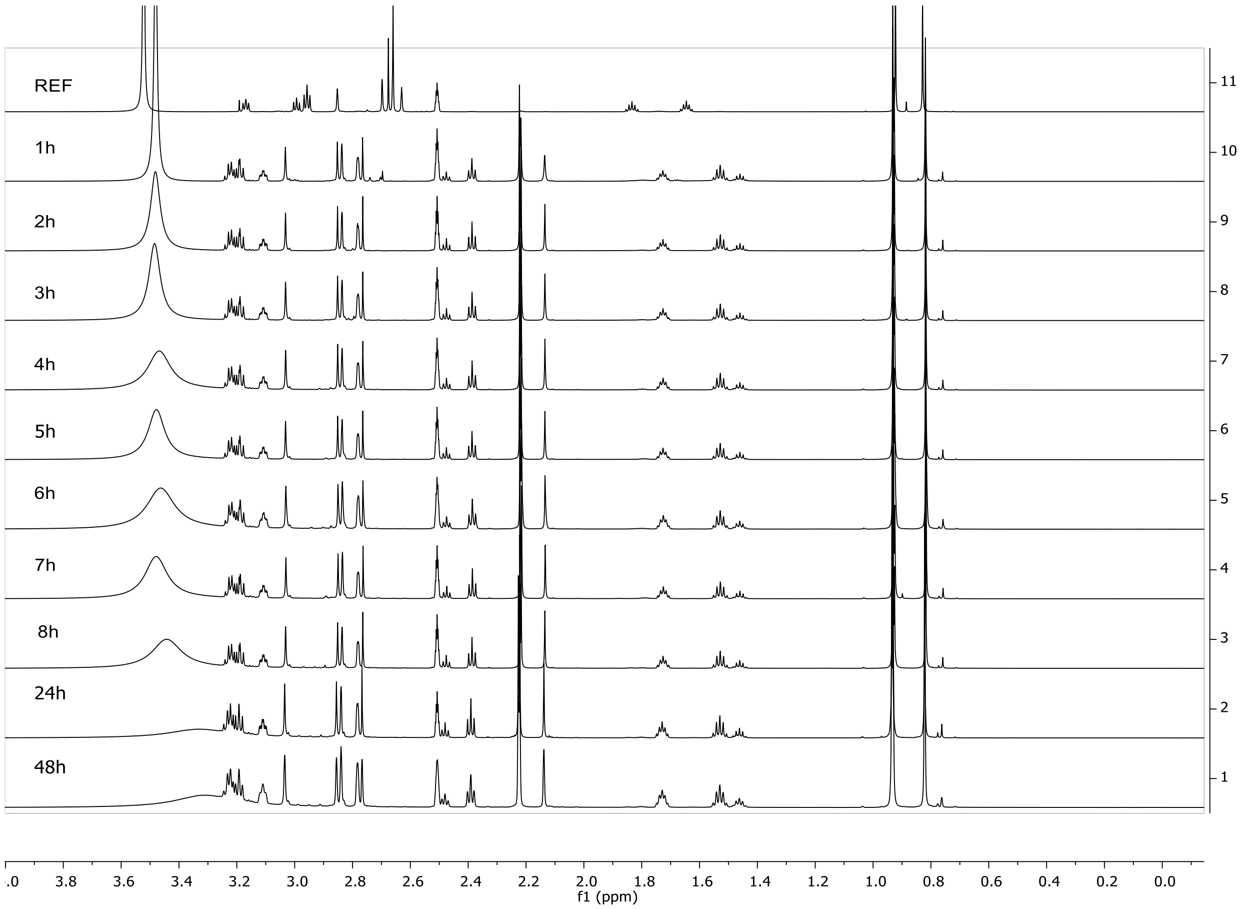

Figure S121 ^{1}H NMR 1 molar equivalent of dm3-mTBD and 10 molar equivalents of water.

11. [dm3-mTBDH][OAc] + water

Structure	Time	integral N-Methyls	integral HP	% HP
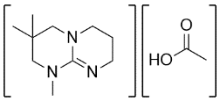	REF	83023600.00	40479.30	0.05
	1h	44786700.00	155179.00	0.35
	2h	21628500.00	145273.00	0.67
	3h	21775200.00	250679.00	1.14
	4h	21329300.00	315149.00	1.46
	5h	21311100.00	385238.00	1.78
	6h	19894100.00	428487.00	2.11
	7h	18193300.00	399583.00	2.15
	8h	20448000.00	646966.00	3.07
	24h	18792300.00	1857350.00	8.99
	48h	31783000.00	5390500.00	14.50

Table S123 1 molar equivalent of [dm3-mTBDH][OAc] and 0.2 molar equivalent of water.

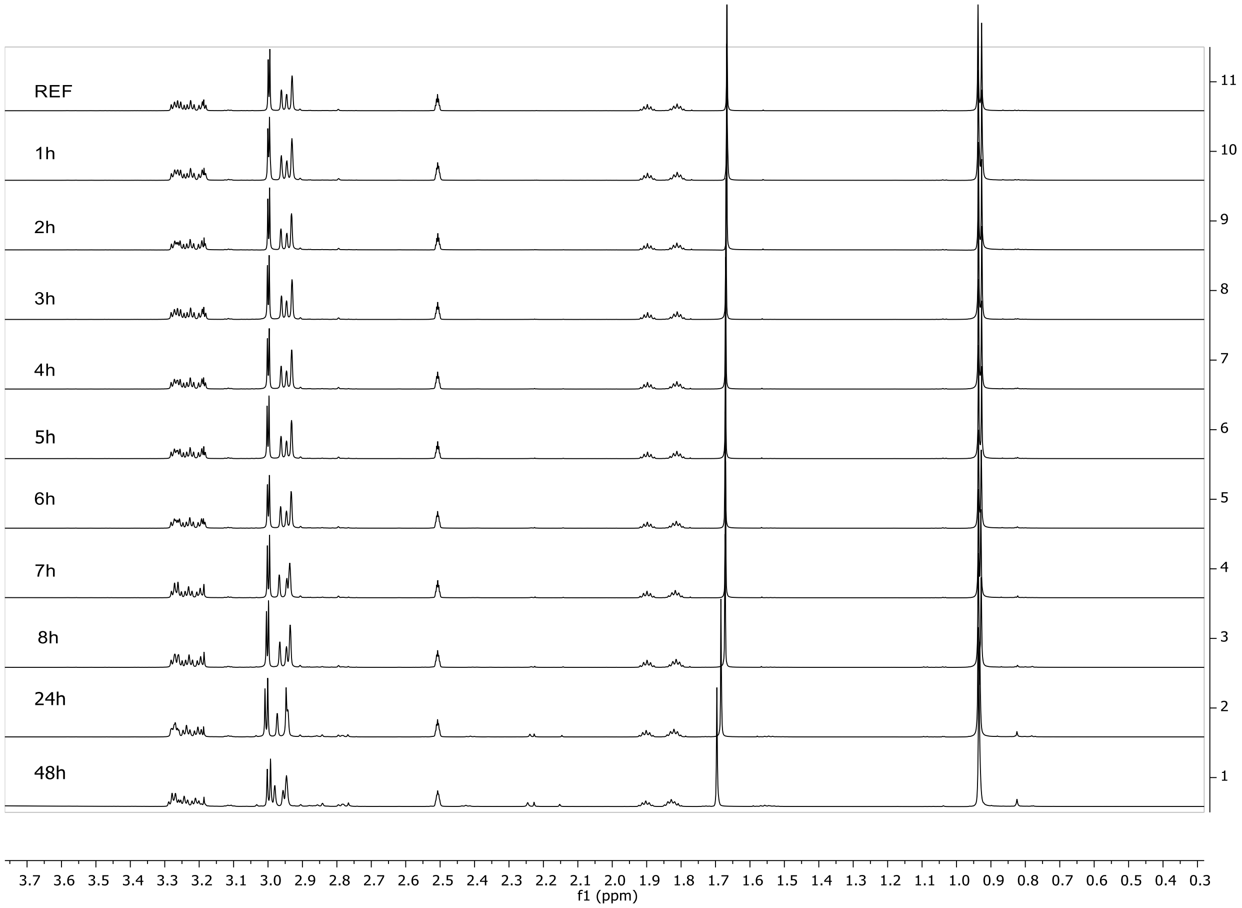

Figure S122 ^{1}H NMR 1 molar equivalent of [dm3-mTBDH][OAc] and 0.2 molar equivalent of water.

Structure	Time	Integral N-Methyls	Integral HP	% HP
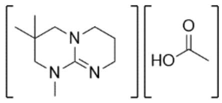	REF	81801100.00	0.00	0.00
	1h	82831900.00	292440.00	0.35
	2h	20990400.00	181960.00	0.86
	3h	19901200.00	284181.00	1.41
	4h	20785800.00	438545.00	2.07
	5h	20436200.00	557636.00	2.66
	6h	135233000.00	4678500.00	3.34
	7h	17654500.00	742448.00	4.04
	8h	17642200.00	854728.00	4.62
	24h	16780200.00	2653280.00	13.65
	48h	28229600.00	8228650.00	22.57

Table S124 1 molar equivalent of [dm3-mTBDH][OAc] and 0.5 molar equivalent of water.

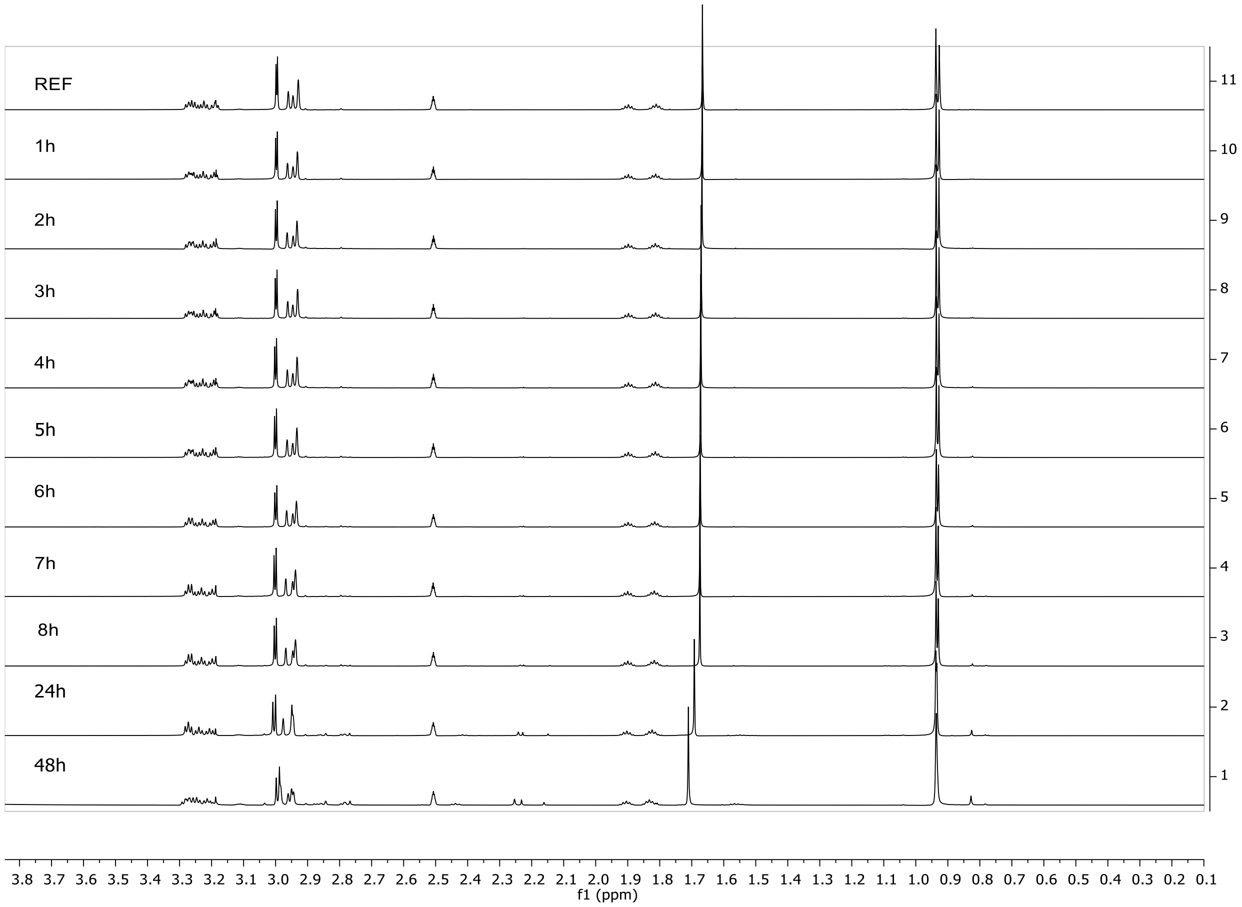

Figure S123 ^{1}H NMR 1 molar equivalent of [dm3-mTBDH][OAc] and 0.5 molar equivalent of water.

Structure	Time	Integral N-Methyls	Integral HP	% HP
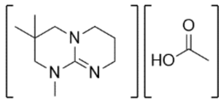	REF	85621600.00	0.00	0.00
	1h	82394100.00	250506.00	0.30
	2h	81392400.00	840100.00	1.02
	3h	21405300.00	361591.00	1.66
	4h	19925100.00	498824.00	2.44
	5h	20718400.00	635184.00	2.97
	6h	18946600.00	750779.00	3.81
	7h	16509700.00	779453.00	4.51
	8h	17045200.00	920399.00	5.12
	24h	15094000.00	2634270.00	14.86
	48h	23651500.00	7643870.00	24.42

Table S125 1 molar equivalent of [dm3-mTBDH][OAc] and 1 molar equivalent of water.

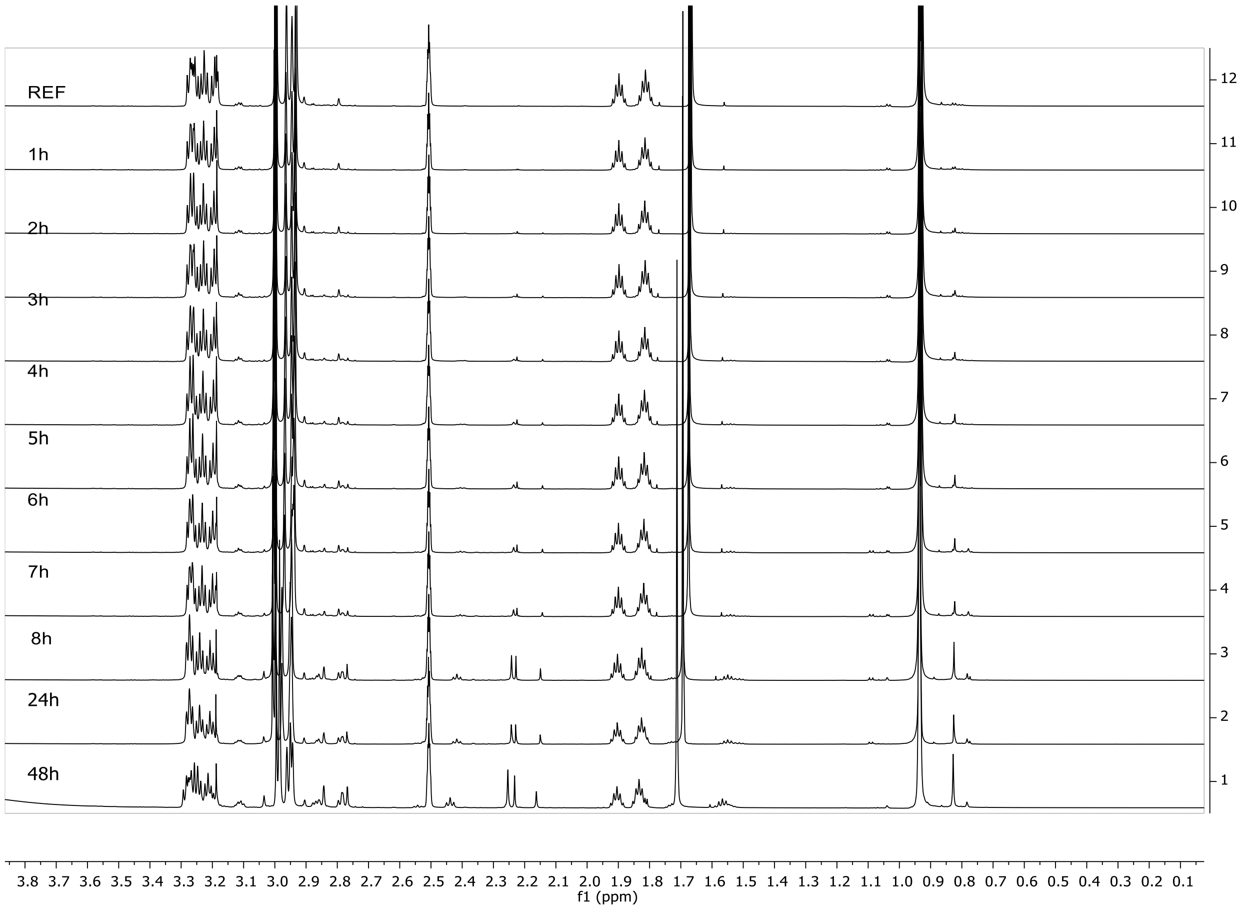

Figure S124 ^{1}H NMR 1 molar equivalent of [dm3-mTBDH][OAc] and 1 molar equivalent of water.

Structure	Time	Integral N-Methyls	Integral HP Total	% HP
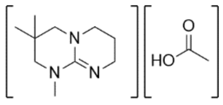	REF	26530000.00	0.00	0.00
	1h	14182900.00	31269.90	0.22
	2h	59369500.00	149758.00	0.25
	3h	16920800.00	45830.00	0.27
	4h	13295400.00	42602.60	0.32
	5h	12511800.00	42147.70	0.34
	6h	11707300.00	42654.80	0.36
	7h	12693200.00	50097.40	0.39
	8h	11973500.00	46929.60	0.39
	24h	15202300.00	144959.00	0.94
	48h	31257400.00	1503820.00	4.59

Table S126 1 molar equivalent of [dm3-mTBDH][OAc] and 5 molar equivalent of water.

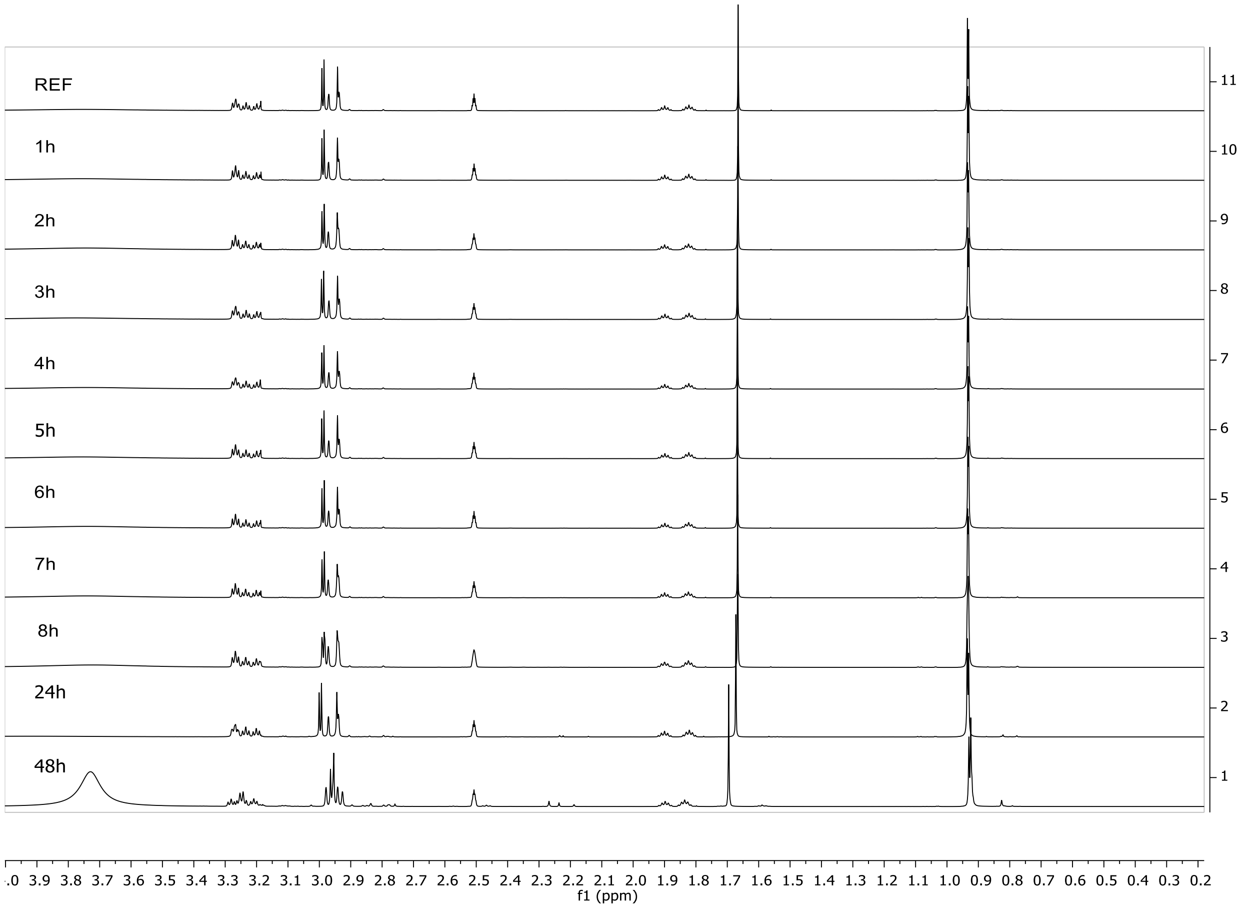

Figure S125 ¹H NMR 1 molar equivalent of [dm3-mTBDH][OAc] and 5 molar equivalent of water.

Structure	Time	Integral N-Methyls	Integral HP	% HP
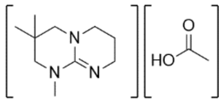	REF	64378800.00	81681.30	0.00
	1h	68330200.00	99010.80	0.14
	2h	17015600.00	39618.80	0.23
	3h	17813500.00	44936.60	0.25
	4h	15457400.00	39859.10	0.26
	5h	16906300.00	50120.90	0.30
	6h	14656000.00	79161.80	0.54
	7h	14743400.00	97503.80	0.66
	8h	13639600.00	126845.00	0.92
	24h	18282600.00	818673.00	4.29
	48h	26847000.00	4359210.00	13.97

Table S127 1 molar equivalent of [dm3-mTBDH][OAc] and 10 molar equivalent of water.

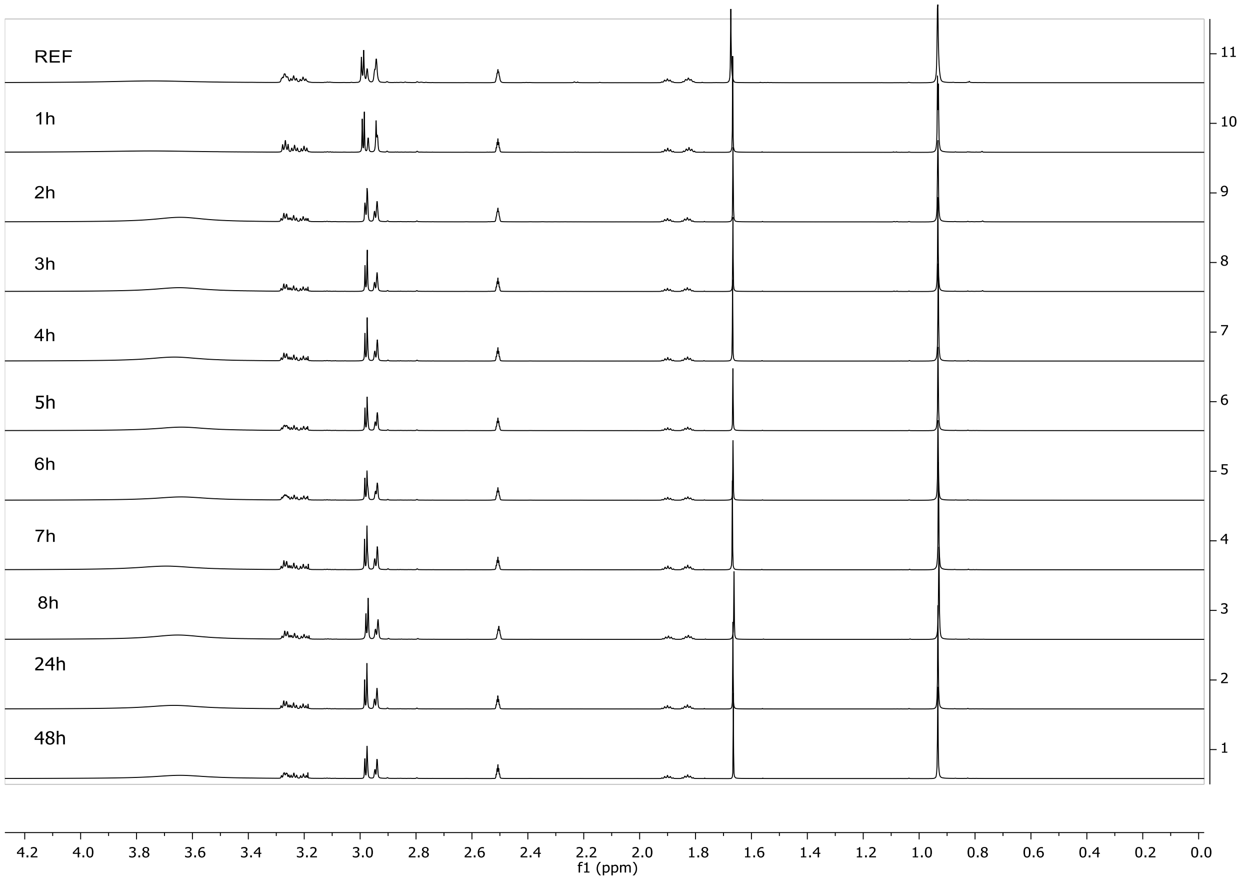

Figure S126 ^{1}H NMR 1 molar equivalent of [dm3-mTBDH][OAc] and 10 molar equivalent of water.

12. m2-mTBD + water

Structure	Time	Integral N-Methyls	Integral HP	% HP
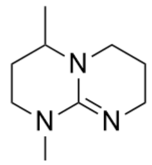	REF	175570000.00	25653.50	0.01
	1h	113922000.00	3568500.00	3.04
	2h	163310000.00	12685100.00	7.21
	3h	94630200.00	10605400.00	10.08
	4h	160835000.00	30191400.00	15.80
	5h	162374000.00	30600500.00	15.86
	6h	98907300.00	21116800.00	17.59
	7h	136984000.00	33113800.00	19.47
	8h	81642700.00	21640000.00	20.95
	24h	89501000.00	64124000.00	41.74
	48h	60679200.00	69011400.00	53.21

Table S128 1 molar equivalent of m2-mTBD and 1 molar equivalent of water.

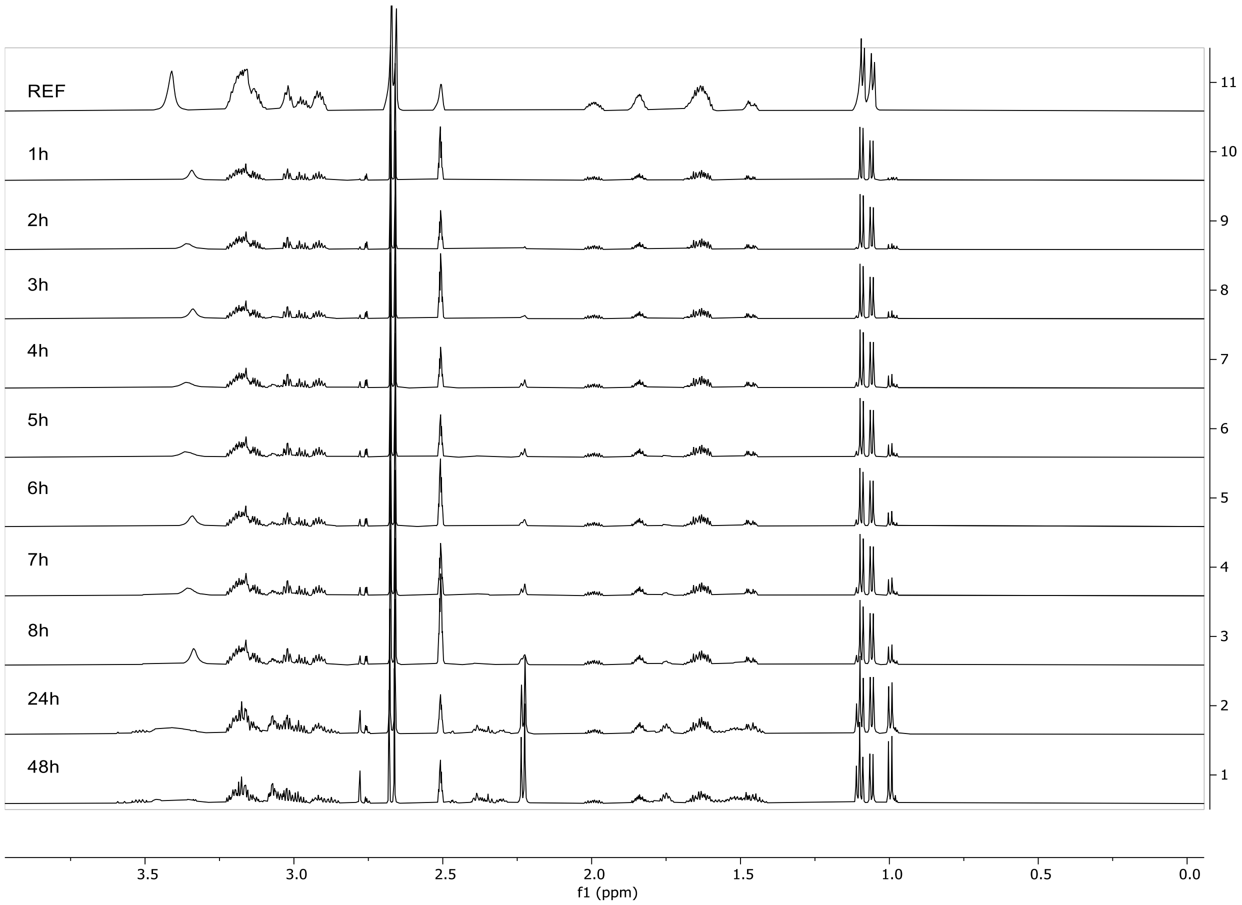

Figure S127 ^{1}H NMR 1 molar equivalent of m2-mTBD and 1 molar equivalent of water.

Structure	Time	Integral N-Methyls	integral HP	% HP
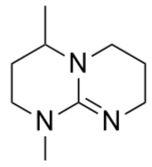	REF	119398000.00	30957.10	0.03
	1h	25754000.00	58524500.00	69.44
	2h	6463050.00	89417100.00	93.26
	3h	149593.00	101904000.00	99.85
	4h	70673.00	84192600.00	99.92
	5h	63569.10	98459600.00	99.94
	6h	115458.00	99338000.00	99.88
	7h	93672.30	94499000.00	99.90
	8h	102393.00	88188300.00	99.88
	24h	201593.00	88808200.00	99.77
	48h	180559.00	72592800.00	99.75

Table S129 1 molar equivalent of m2-mTBD and 5 molar equivalents of water.

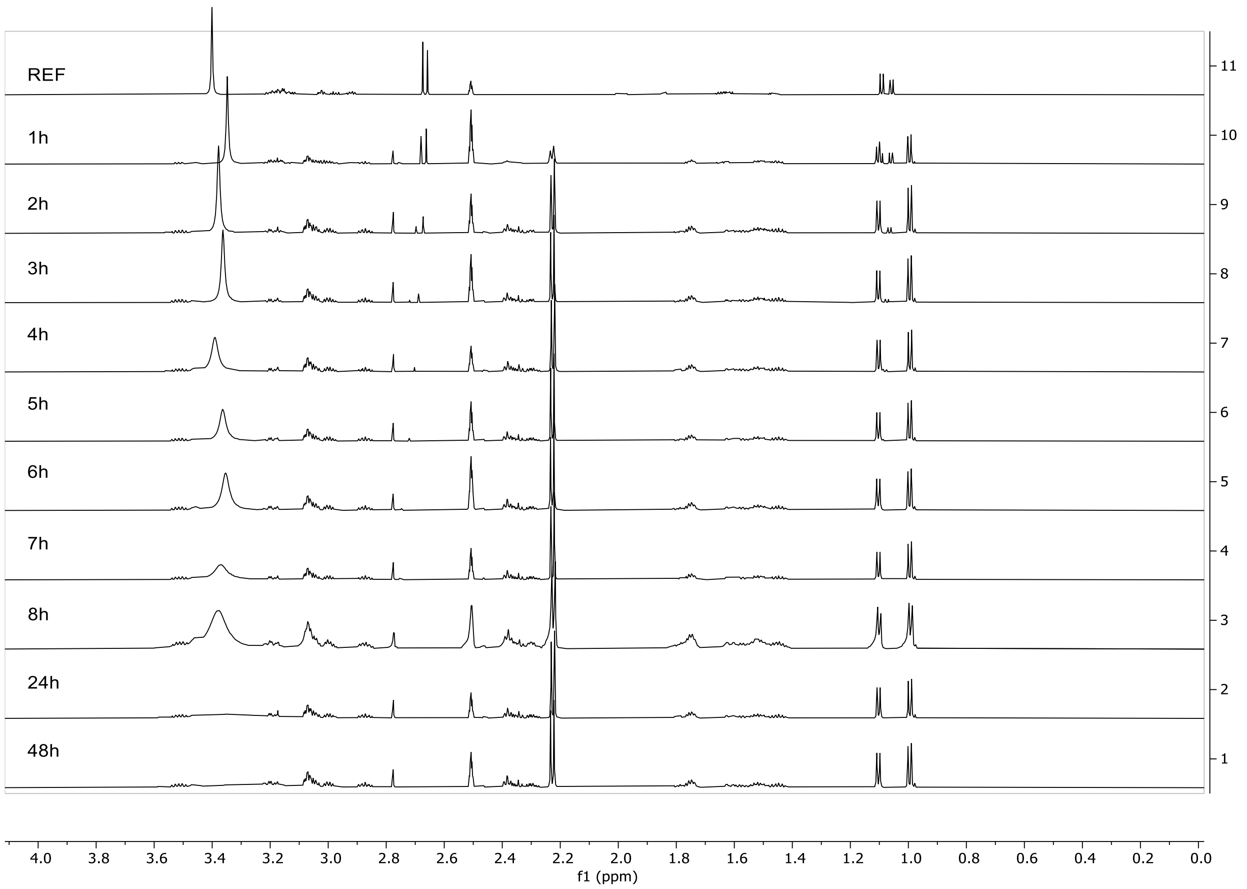

Figure S128 ^{1}H NMR 1 molar equivalent of m2-mTBD and 5 molar equivalents of water.

Structure	Time	Integral N-Methyl 1	Integral HP	% HP
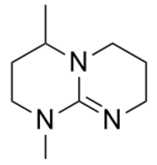	REF	81831600.00	179220.00	0.22
	1h	136563.00	56006700.00	99.76
	2h	540111.00	71540500.00	99.25
	3h	279126.00	44451300.00	99.38
	4h	579165.00	78031900.00	99.26
	5h	581767.00	80161600.00	99.28
	6h	614256.00	68265300.00	99.11
	7h	877760.00	79730800.00	98.91
	8h	577933.00	63026400.00	99.09
	24h	942660.00	90959600.00	98.97
	48h	957250.00	72830700.00	98.70

Table S130 1 molar equivalent of m2-mTBD and 10 molar equivalents of water.

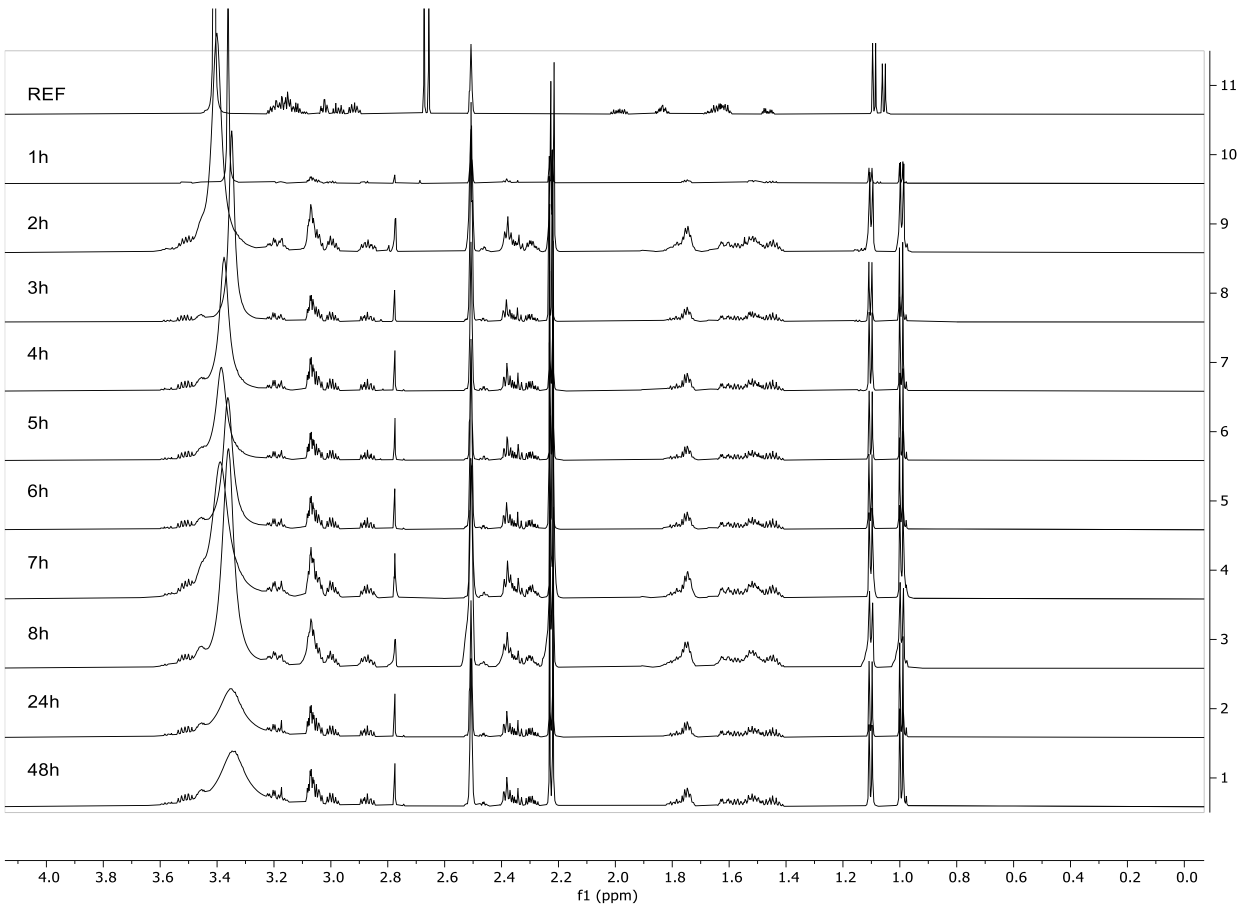

Figure S129 ^{1}H NMR 1 molar equivalent of m2-mTBD and 10 molar equivalents of water.

13. [m2-mTBDH][OAc] + water

Structure	Time	integral N-Methyls	integral HP	% HP
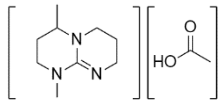	REF	121532000.00	0.00	0.00
	1h	158821000.00	0.00	0.00
	2h	130505000.00	11438.70	0.01
	3h	111235000.00	11988.70	0.01
	4h	154623000.00	70493.20	0.05
	5h	136963000.00	170471.00	0.12
	6h	127065000.00	217156.00	0.17
	24h	140220000.00	4260860.00	2.95
	48h	116144000.00	4629090.00	3.83

Table S131 1 molar equivalent of [m2-mTBDH][OAc] and 1 molar equivalent of water

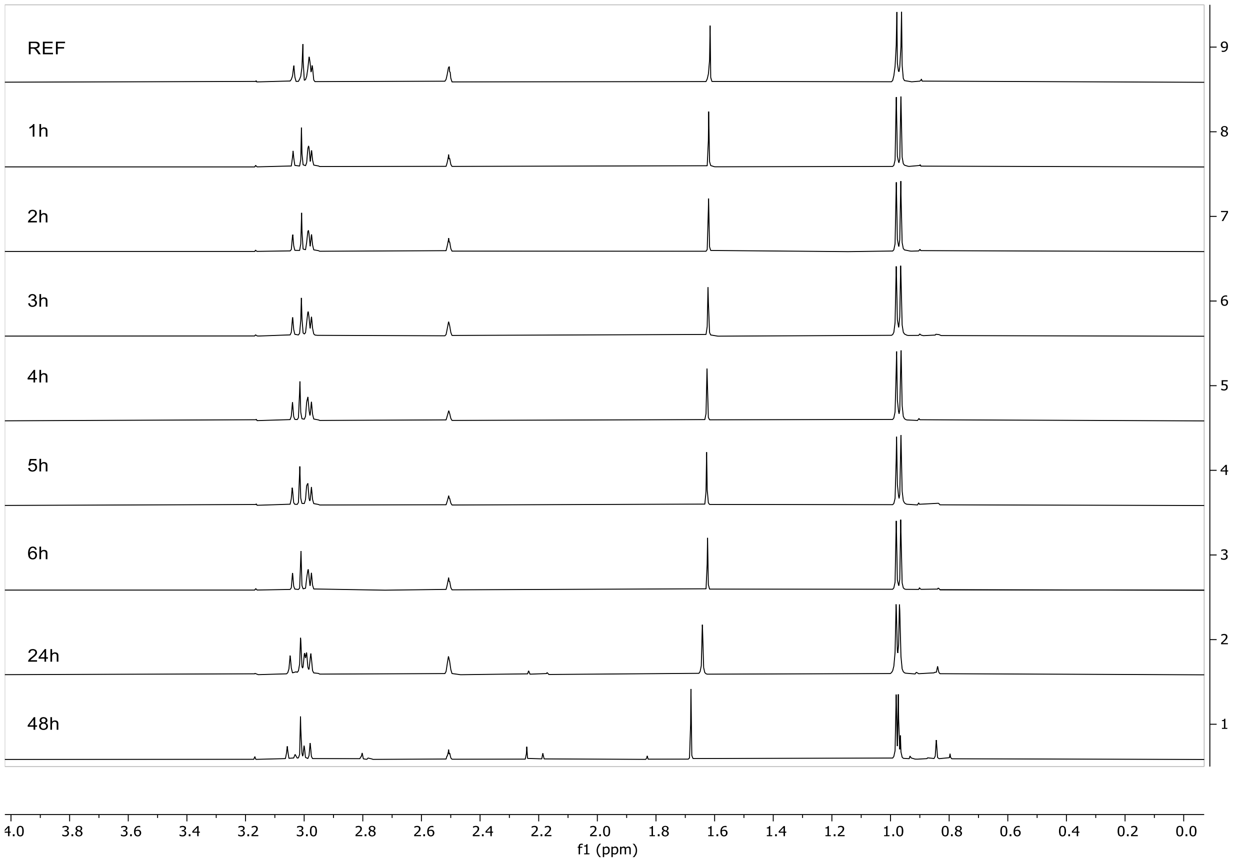

Figure S130 ^{1}H NMR 1 molar equivalent of [m2-mTBDH][OAc] and 1 molar equivalent of water

Structure	Time	Integral N-Methyls	Integral HP	% HP
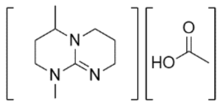	REF	91603600.00	16450.30	0.02
	1h	80429700.00	56326.30	0.07
	2h	77806800.00	73770.30	0.09
	3h	62384400.00	72907.80	0.12
	4h	80910600.00	103888.00	0.13
	5h	85839800.00	151706.00	0.18
	6h	88269700.00	354764.00	0.40
	7h	91495600.00	184078.00	0.20
	8h	86617100.00	191821.00	0.22
	24h	84997600.00	578783.00	0.68
	48h	88409800.00	759733.00	0.85

Table S132 1 molar equivalent of [m2-mTBDH][OAc] and 5 molar equivalent of water.

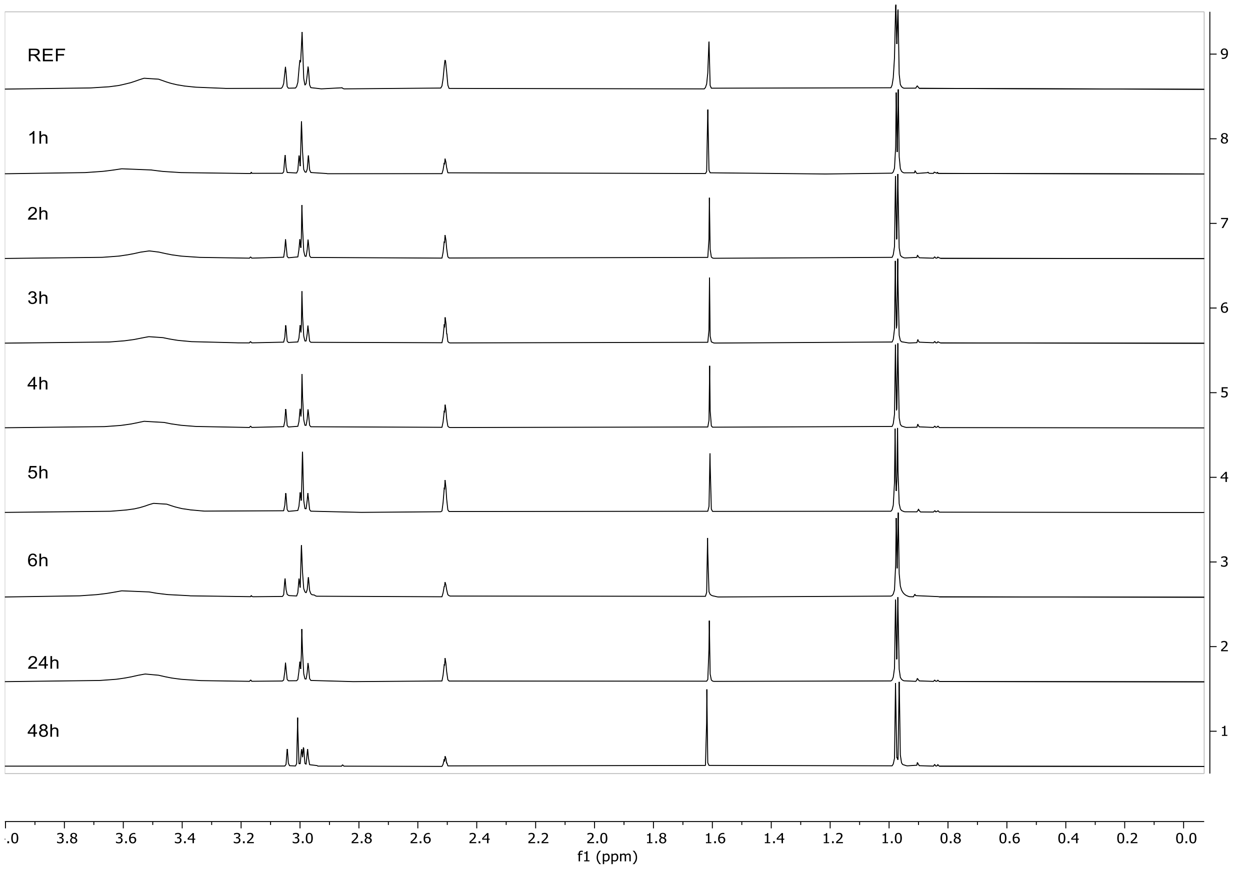

Figure S131 ^{1}H NMR 1 molar equivalent of [m2-mTBDH][OAc] and 5 molar equivalent of water.

Structure	Time	Integral N Methyls	Integral HP	% HP
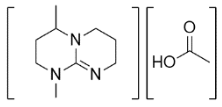	REF	6516860000000000.00	6564740000000.00	0.10
	1h	10166300000000000.00	11628000000000.00	0.11
	2h	21450100000000000.00	27307600000000.00	0.13
	3h	7138840000000000.00	9401840000000.00	0.13
	4h	10383000000000000.00	16019200000000.00	0.15
	5h	20818900000000000.00	34186800000000.00	0.16
	6h	22103600000000000.00	38744800000000.00	0.17
	7h	16392700000000000.00	27569200000000.00	0.17
	8h	25885900000000000.00	46710000000000.00	0.18
	24h	9880040000000000.00	23709800000000.00	0.24
	48h	7912300000000000.00	63436500000000.00	0.80

Table S133 1 molar equivalent of [m2-mTBDH][OAc] and 10 molar equivalent of water.

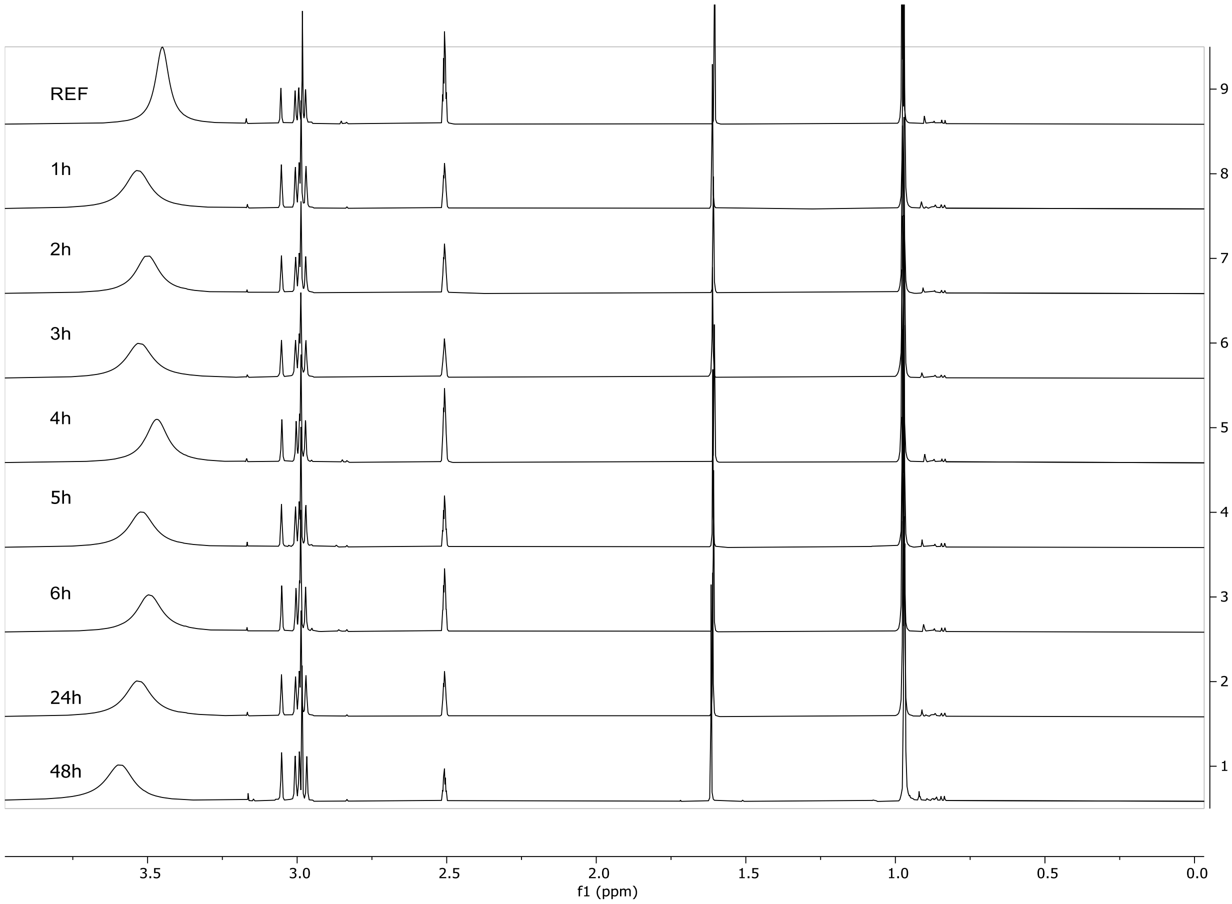

Figure S132 ^{1}H NMR 1 molar equivalent of [m2-mTBDH][OAc] and 10 molar equivalent of water.

II. Half-time calculation

When the half-time is reached during the experiment, the value is readable from the plot using the experimental data. When the half-time is not reached during the experiment, the value is calculated using the function fitting as close as possible to our data. The obtained value is an estimation and may differ from experimental data.

1. SB and their salts with pure water at 90°C

Molecule	Water equivalents	Half-time in hours
mTBD	0.2	8
mTBD	0.5	6
mTBD	1	8
mTBD	1.3	4.7
mTBD	2	1
mTBD	5	0.5
mTBD	10	0.5
[mTBDH][OAc]	0.2	Over 48h. non-predictable
[mTBDH][OAc]	0.5	Over 48h. non-predictable
[mTBDH][OAc]	1	Over 48h. non-predictable
[mTBDH][OAc]	5	Over 48h. non-predictable
[mTBDH][OAc]	10	Over 48h. non-predictable

mTBN	0.2	Over 48h. non-predictable
mTBN	0.5	Over 48h. non-predictable
mTBN	1	Over 48h. non-predictable
mTBN	1.5	Over 48h. non-predictable
mTBN	3	18.5
mTBN	5	2.3
mTBN	10	0.86
[mTBNH][OAc]	0.2	Over 48h. non-predictable
[mTBNH][OAc]	0.5	Over 48h. non-predictable
[mTBNH][OAc]	1	Over 48h. non-predictable
[mTBNH][OAc]	5	Over 48h. non-predictable
[mTBNH][OAc]	10	Over 48h. non-predictable
mTBO	0.2	Over 48h. non-predictable
mTBO	0.5	Over 48h. non-predictable
mTBO	1	Over 48h. non-predictable
mTBO	5	1.33
mTBO	10	0.76
dm39-mTBD	1	35.3
dm39-mTBD	5	0.72
dm39-mTBD	10	0.5
[dm39-mTBDH][OAc]	1	Over 48h. non-predictable
[dm39-mTBDH][OAc]	5	Over 48h. non-predictable
[dm39-mTBDH][OAc]	10	Over 48h. non-predictable
dm3-m0-mTBD	1	42
dm3-m0-mTBD	5	2.9
dm3-m0-mTBD	10	2
[dm3-m0-mTBDH][OAc]	1	Over 48h. non-predictable
[dm3-m0-mTBDH][OAc]	5	Over 48h. non-predictable
[dm3-m0-mTBDH][OAc]	10	Over 48h. non-predictable
dm3-mTBD	0.2	3.6
dm3-mTBD	0.5	3.5
dm3-mTBD	1	6
dm3-mTBD	1.5	7.6
dm3-mTBD	3	1.65
dm3-mTBD	5	1
dm3-mTBD	10	0.65
[dm3-mTBDH][OAc]	0.2	28.5
[dm3-mTBDH][OAc]	0.5	Over 48h. non-predictable
[dm3-mTBDH][OAc]	1	Over 48h. non-predictable
[dm3-mTBDH][OAc]	5	Over 48h. non-predictable
[dm3-mTBDH][OAc]	10	Over 48h. non-predictable
m2-mTBD	1	41.2
m2-mTBD	5	0.72
m2-mTBD	10	0.5
[m2-mTBDH][OAc]	1	Over 48h. non-predictable

[m2-mTBDH][OAc]	5	Over 48h. non-predictable
[m2-mTBDH][OAc]	10	Over 48h. non-predictable
[mTBDH][ClOAc]	0.2	Over 48h. non-predictable
[mTBDH][ClOAc]	0.5	Over 48h. non-predictable
[mTBDH][ClOAc]	1	Over 48h. non-predictable
[mTBDH][ClOAc]	5	Over 48h. non-predictable
[mTBDH][ClOAc]	10	Over 48h. non-predictable
[mTBDH][Cl_2OAc]	0.2	Over 48h. non-predictable
[mTBDH][Cl_2OAc]	0.5	Over 48h. non-predictable
[mTBDH][Cl_2OAc]	1	Over 48h. non-predictable
[mTBDH][Cl_2OAc]	5	Over 48h. non-predictable
[mTBDH][Cl_2OAc]	10	Over 48h. non-predictable
[mTBDH][Cl_3OAc]	0.2	Over 48h. non-predictable
[mTBDH][Cl_3OAc]	0.5	Over 48h. non-predictable
[mTBDH][Cl_3OAc]	1	Over 48h. non-predictable
[mTBDH][Cl_3OAc]	5	Over 48h. non-predictable
[mTBDH][Cl_3OAc]	10	Over 48h. non-predictable
[mTBNH][ClOAc]	0.2	Over 48h. non-predictable
[mTBNH][ClOAc]	0.5	Over 48h. non-predictable
[mTBNH][ClOAc]	1	Over 48h. non-predictable
[mTBNH][ClOAc]	5	Over 48h. non-predictable
[mTBNH][ClOAc]	10	Over 48h. non-predictable
[mTBNH][Cl_2OAc]	0.2	Over 48h. non-predictable
[mTBNH][Cl_2OAc]	0.5	Over 48h. non-predictable
[mTBNH][Cl_2OAc]	1	Over 48h. non-predictable
[mTBNH][Cl_2OAc]	5	Over 48h. non-predictable
[mTBNH][Cl_2OAc]	10	Over 48h. non-predictable
[mTBNH][Cl_3OAc]	0.2	Over 48h. non-predictable
[mTBNH][Cl_3OAc]	0.5	Over 48h. non-predictable
[mTBNH][Cl_3OAc]	1	Over 48h. non-predictable
[mTBNH][Cl_3OAc]	5	Over 48h. non-predictable
[mTBNH][Cl_3OAc]	10	Over 48h. non-predictable

Table S134 Half-life of SB and their corresponding salt.

2. Superbase and their acetate salt in presence of water and co-solvent at 90°C

Molecule	Water equivalents	Co-solvent	Co-solvent equivalents	Half-time in hours
[mTBDH][OAc]	1	DMSO	0.2	Over 48h. non-predictable
[mTBDH][OAc]	1	DMSO	0.5	Over 48h. non-predictable
[mTBDH][OAc]	1	DMSO	1	Over 48h. non-predictable
[mTBDH][OAc]	1	DMSO	2	Over 48h. non-predictable
mTBD	1	DMSO	0.2	7.5
mTBD	1	DMSO	0.5	15.1
mTBD	1	DMSO	1	26.7
mTBD	1	DMSO	2	Over 48h. non-predictable
[mTBDH][OAc]	1	Ethylene glycol	0.2	Over 48h. non-predictable
[mTBDH][OAc]	1	Ethylene glycol	0.5	Over 48h. non-predictable
[mTBDH][OAc]	1	Ethylene glycol	1	Over 48h. non-predictable
[mTBDH][OAc]	1	Ethylene glycol	2	Over 48h. non-predictable
mTBD	1	Ethylene glycol	0.2	3.5
mTBD	1	Ethylene glycol	0.5	1.3
mTBD	1	Ethylene glycol	1	0.91
mTBD	1	Ethylene glycol	2	0.88
[mTBDH][OAc]	1	Phenol	0.2	Over 48h. non-predictable
[mTBDH][OAc]	1	Phenol	0.5	Over 48h. non-predictable
[mTBDH][OAc]	1	Phenol	1	Over 48h. non-predictable
[mTBDH][OAc]	1	Phenol	2	Over 48h. non-predictable
mTBD	1	Phenol	0.2	2.98
mTBD	1	Phenol	0.5	3.12
mTBD	1	Phenol	1	8.5
mTBD	1	Phenol	2	10.76
mTBD	1	Propanol	0.2	5.8
mTBD	1	Propanol	0.5	5.36
mTBD	1	Propanol	1	5
mTBD	1	Propanol	2	5.53
[mTBDH][OAc]	1	Propanol	0.2	Over 48h. non-predictable
[mTBDH][OAc]	1	Propanol	0.5	Over 48h. non-predictable
[mTBDH][OAc]	1	Propanol	1	Over 48h. non-predictable
[mTBDH][OAc]	1	Propanol	2	Over 48h. non-predictable

Table S135 Half-life of SB and their corresponding salt in presence of co-solvent

3. SB and their acetate salts over a three days period at different temperature

Molecule	Water equivalents	Temperature	Half time
mTBD	1	70°C	0.345 days (24.84h)
mTBD	5	70°C	< 24h
mTBD	10	70°C	< 24h
[mTBDH][OAc]	0.2	70°C	8.1 days (194.4h)
[mTBDH][OAc]	0.5	70°C	Over 12 days. non-predictable

[mTBDH][OAc]	1	70°C	Over 12 days. non-predictable
mTBN	1	70°C	Over 12 days. non-predictable
mTBN	5	70°C	< 24h
mTBN	10	70°C	< 24h
[mTBNH][OAc]	1	70°C	Over 12 days. non-predictable
[mTBNH][OAc]	5	70°C	Over 12 days. non-predictable
[mTBNH][OAc]	10	70°C	Over 12 days. non-predictable

Table S136 Half-life of SB and their corresponding salt.

III. Curves of hydrolysis

In this section, we collected the hydrolysis data obtained by NMR and plotted them together on a same graph for comparison. We first did this for each superbase (individually) in different water conditions. Then we plotted the data from an individual superbase + different co-solvent (individual again) amount with a constant water amount (here 1 molar equivalent according to the superbase). After that we repeated the same plotting but for the corresponding carboxylate salt.

1. mTBD with different amounts of water

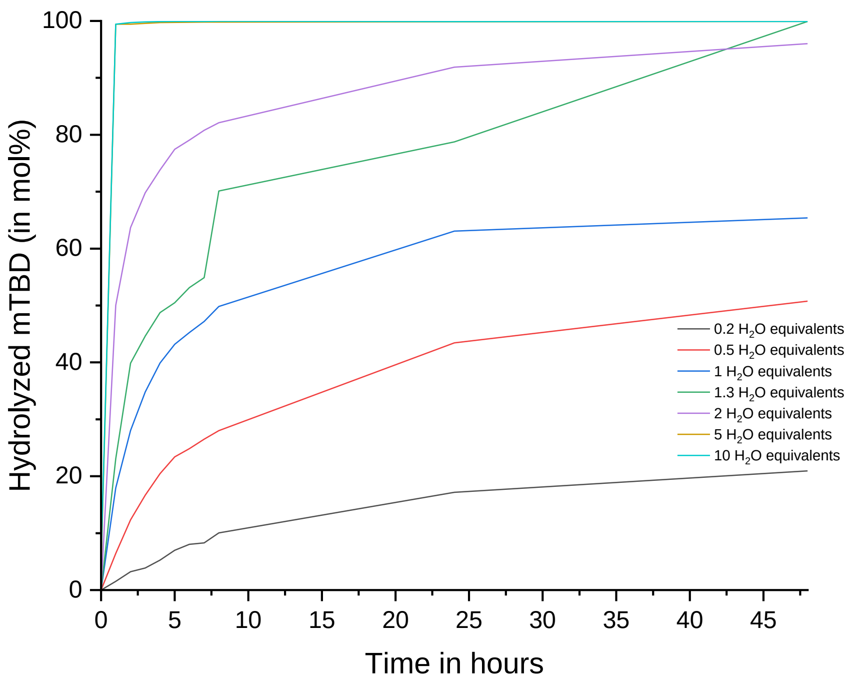

Figure S133 hydrolysis of mTBD with different water amounts.

2. mTBD with different amounts of DMSO

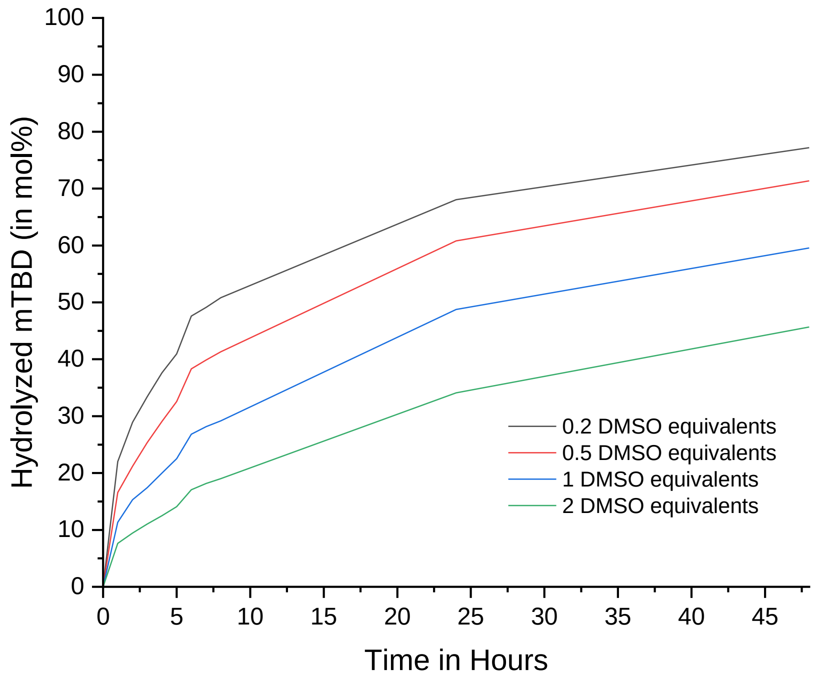

Figure S134 hydrolysis of mTBD + DMSO with different DMSO amounts.

3. mTBD with different amounts of Ethylene Glycol

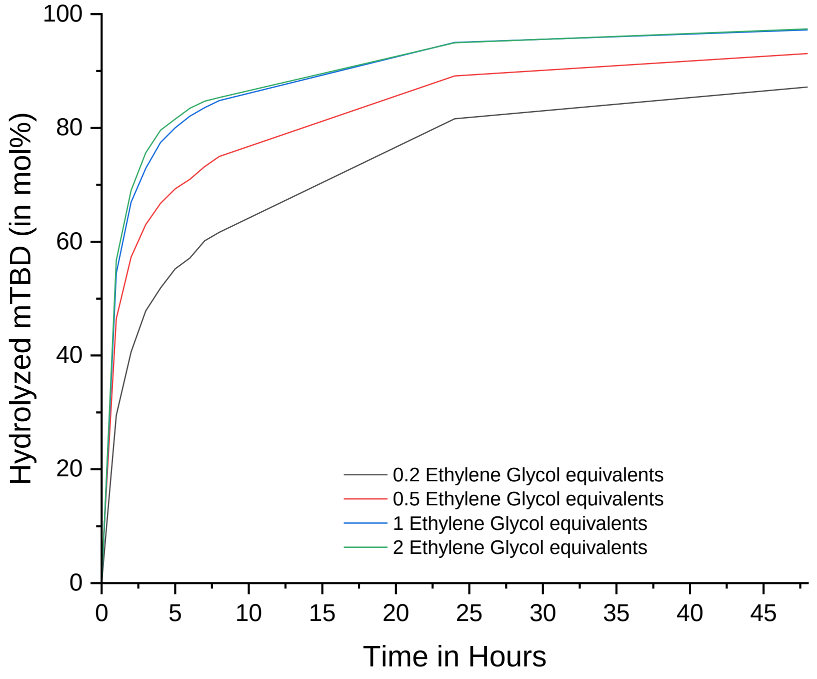

Figure S135 hydrolysis of mTBD + Ethylene Glycol with different Ethylene Glycol amounts.

4. mTBD with different amounts of Phenol

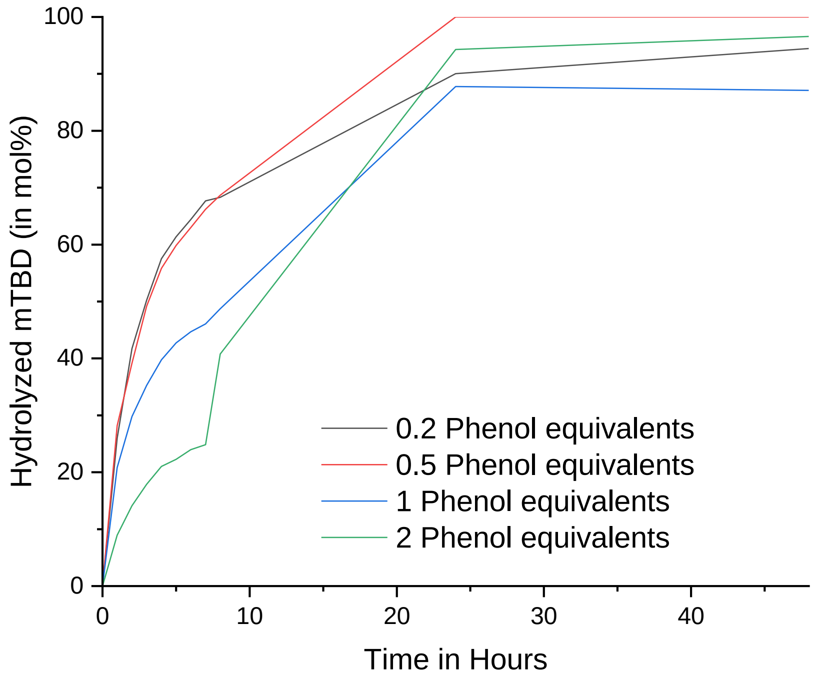

Figure S136 hydrolysis of mTBD + Phenol with different phenol amounts.

5. mTBD with different amounts of Propanol

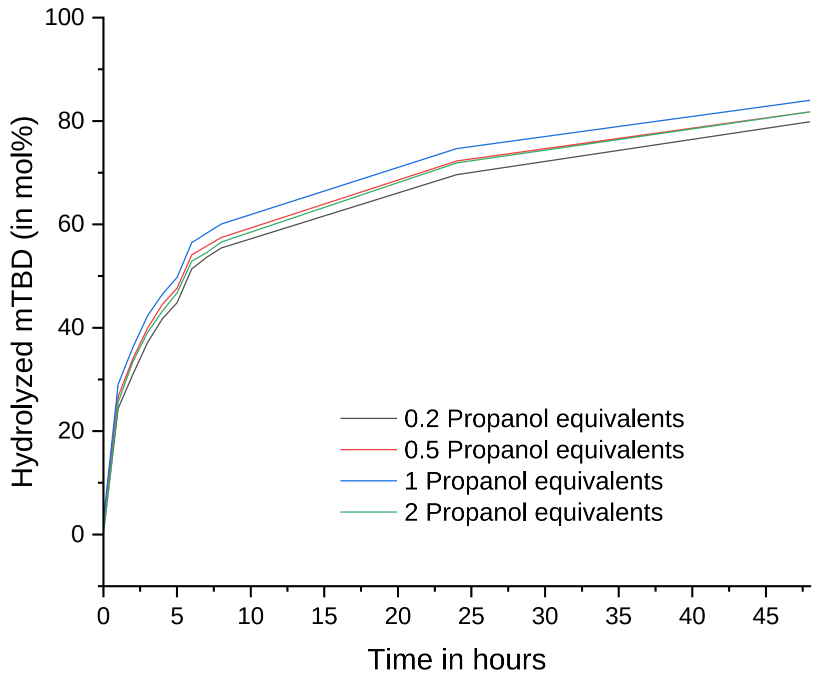

Figure S137 hydrolysis of mTBD + Propanol with different Propanol amounts.

6. mTBN with different amounts of water

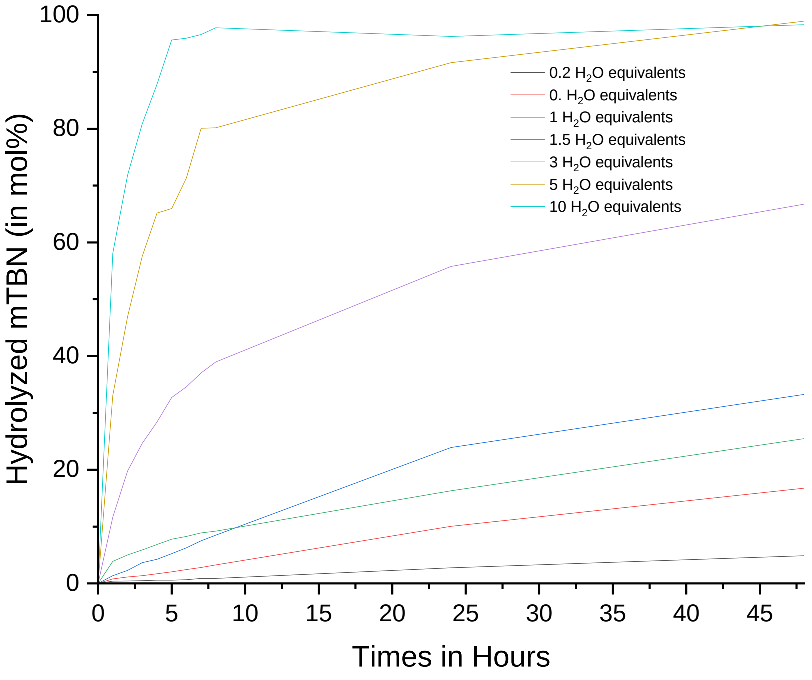

Figure S138 hydrolysis of mTBN with different water amounts.

7. dm39-mTBD with different amounts of water

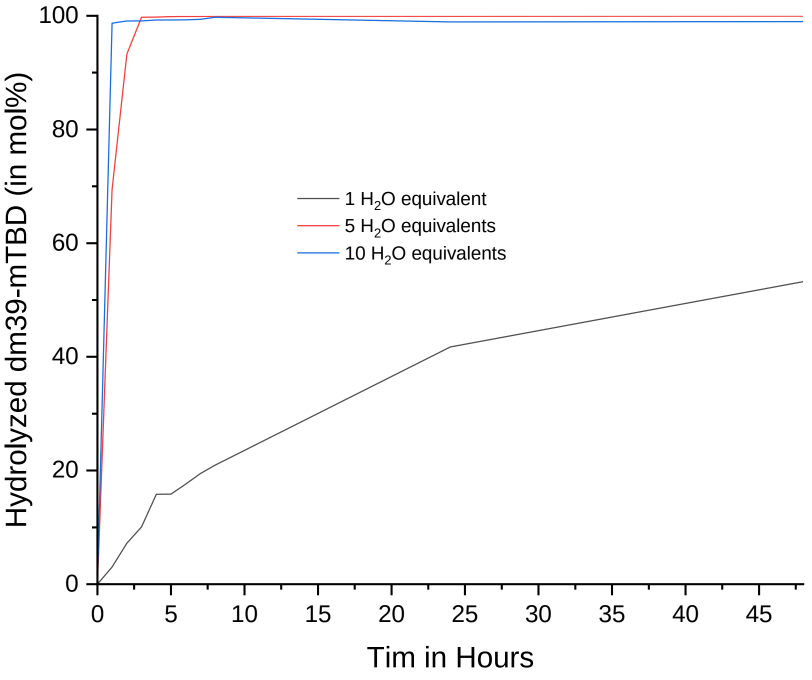

Figure S 139 hydrolysis of dm39-mTBD with different water amounts.

8. dm3-m0-mTBD with different amounts of water

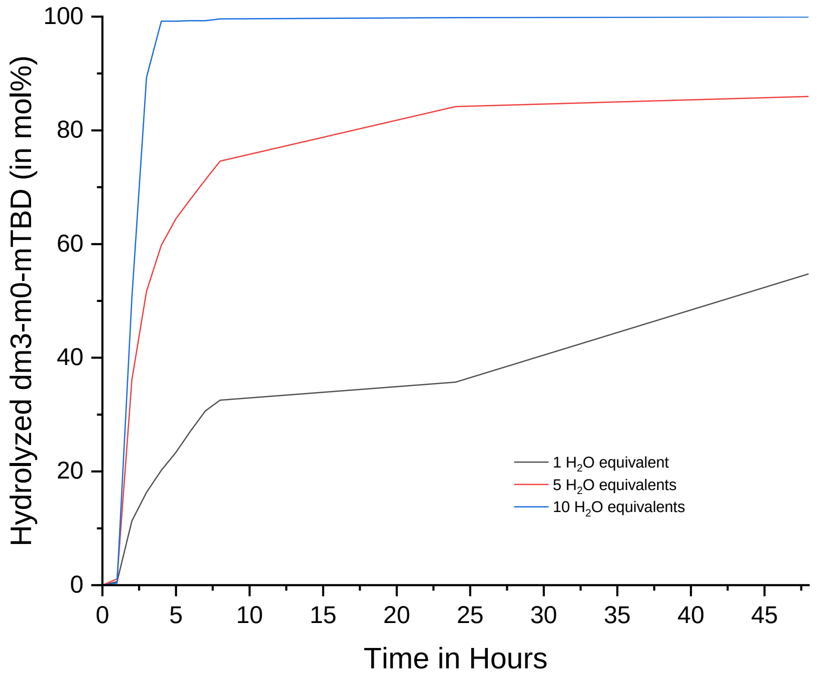

Figure S140 hydrolysis of dm3-m0-mTBD with different water amounts.

9. dm3-mTBD with different amounts of water

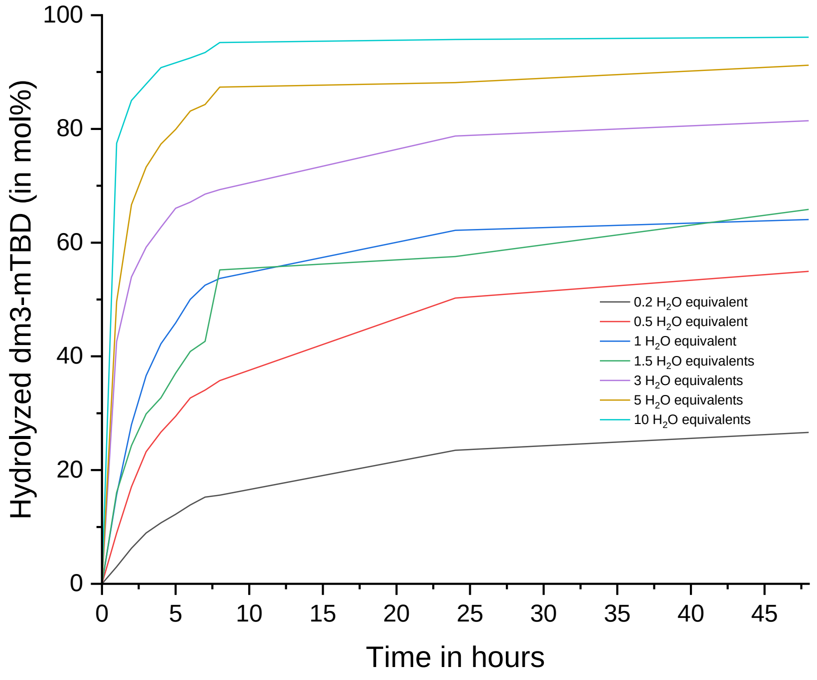

Figure S141 hydrolysis of dm3-mTBD with different water amounts.

10. m2-mTBD with different amounts of water

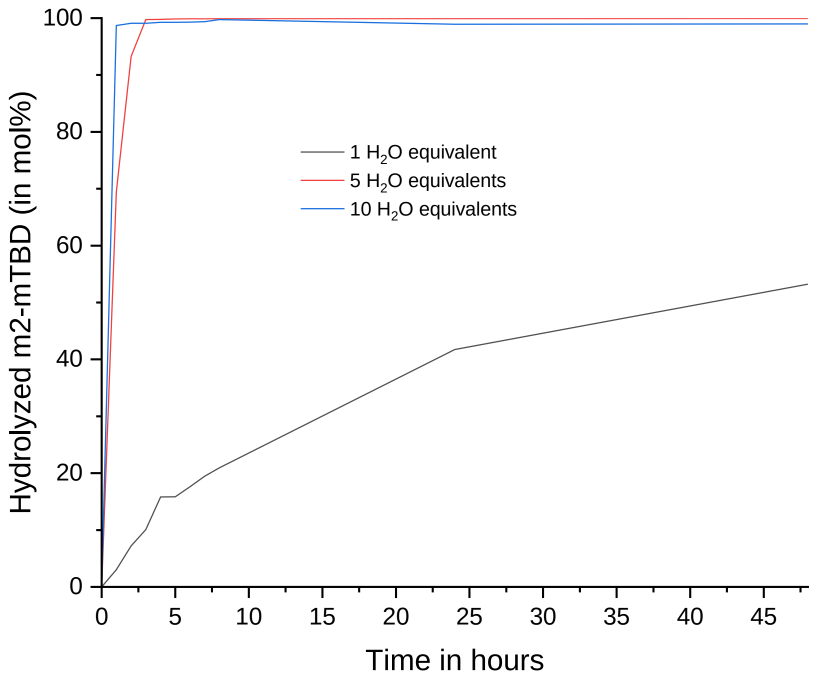

Figure S142 hydrolysis of m2-mTBD with different water amounts.

11. [mTBDH][OAc] with different amounts of water

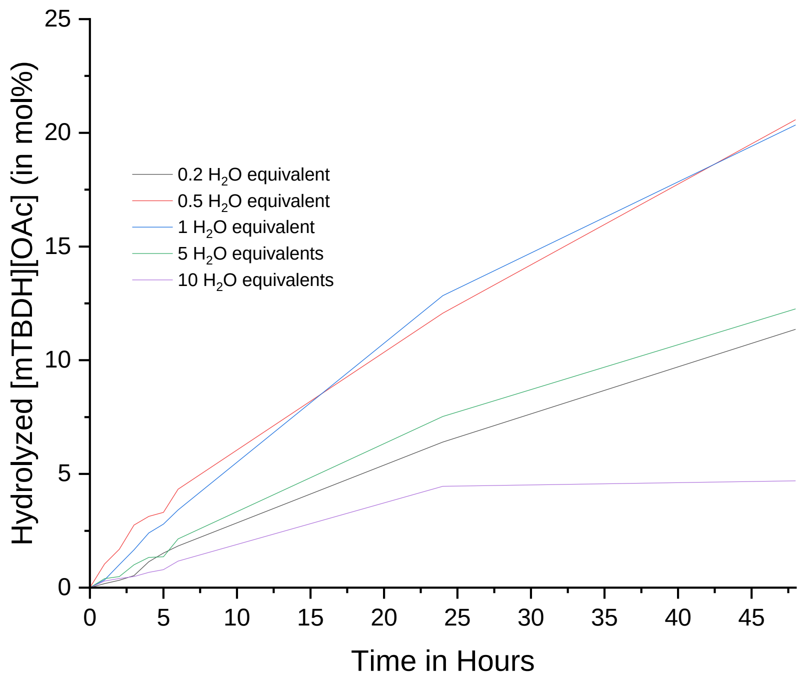

Figure S143 hydrolysis of [mTDBH][OAc] with different water amounts.

12. [mTBDH][OAc] with different amounts of DMSO

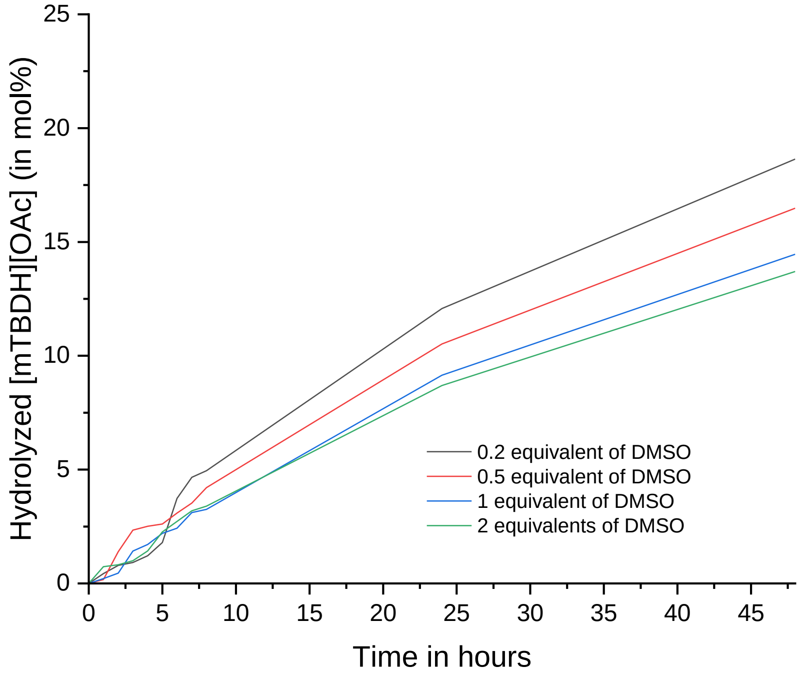

Figure S144 hydrolysis of [mTDBH][OAc] + DMSO withh different DMSO amounts.

13. [mTBDH][OAc] with different amounts of Ethylene Glycol

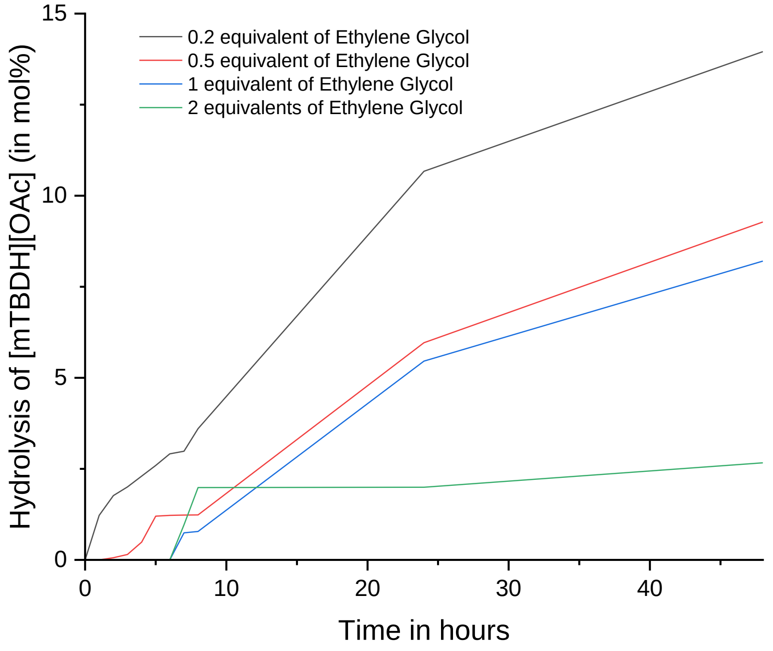

Figure S145 hydrolysis of [mTDBH][OAc] + Ethylene Glycol with different Ethylene Glycol amounts.

14. [mTBDH][OAc] with different amounts of Phenol

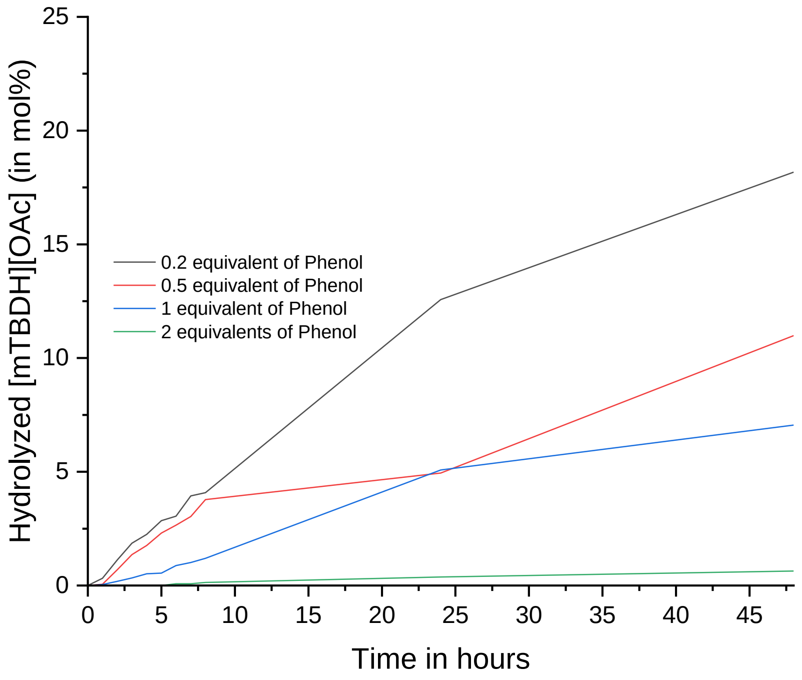

Figure S146 hydrolysis of [mTDBH][OAc] + Phenol with different phenol amounts.

15. [mTBDH][OAc] with different amounts of Propanol

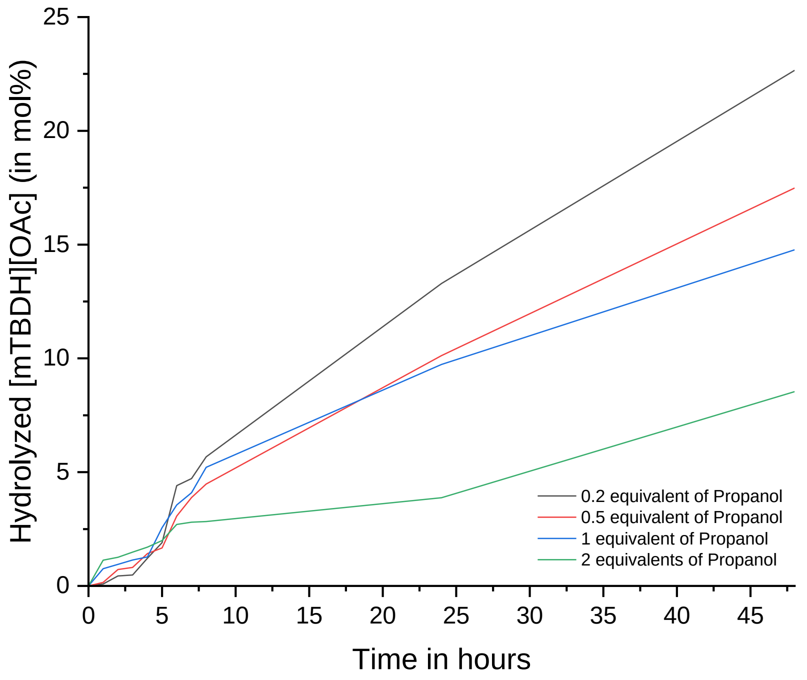

Figure S147 hydrolysis of [mTDBH][OAc] + Propanol with different propanol amount.

16. [mTBNH][OAc] with different amounts of water

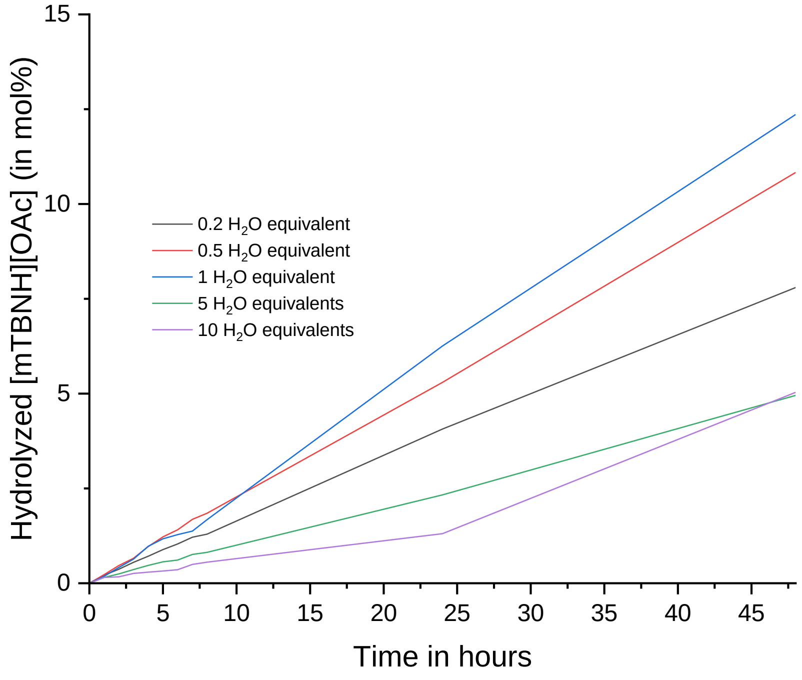

Figure S148 hydrolysis of [mTBNH][OAc] with different water amounts.

17. [dm39-mTBDH][OAc] with different amount of water

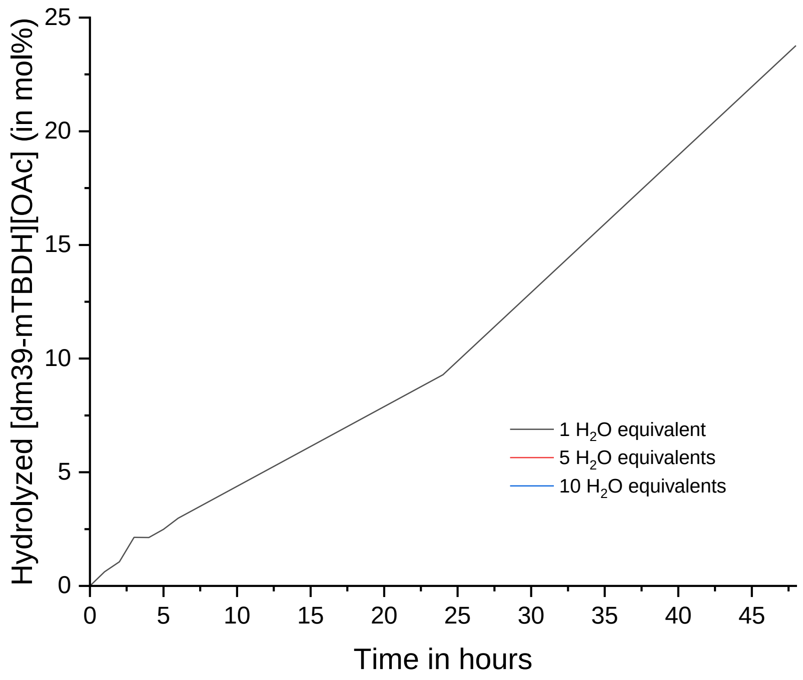

Figure S149 hydrolysis of [dm39-mTDBH][OAc] with different water amounts.

18. [dm3-m0-mTBDH][OAc] with different amount of water

In this specific case. no hydrolysis happens in 48 hours no matter the amount of water used.

19. [dm3-mTBDH][OAc] with different amount of water

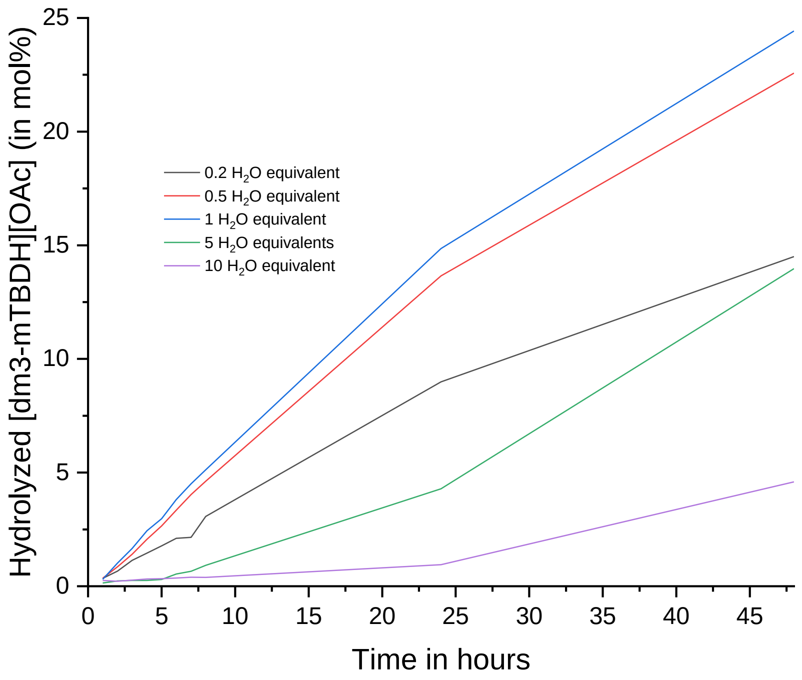

Figure S150 hydrolysis of [dm3-mTDBH][OAc] with different water amounts.

20. [m2-mTBDH][OAc] with different amount of water

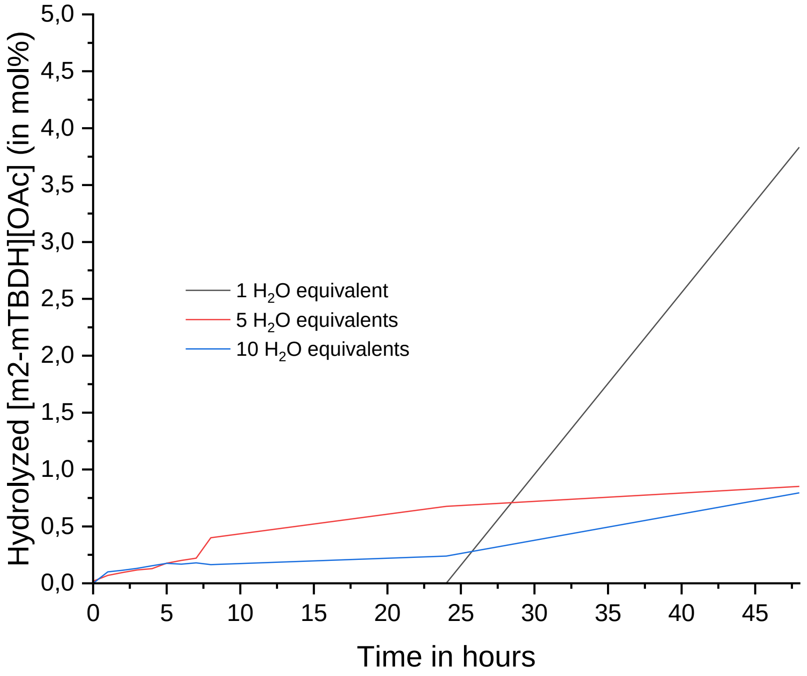

Figure S151 hydrolysis of [m2-mTDBH][OAc] with different water amounts.

IV. Conductivity.

To follow the evolution of the hydroxyl salt formation of a superbase in water, we performed a conductivity experiment by titrating the superbase with water. Simultaneously, at each water equivalents and after waiting until the conductivity value was stable. we performed a FTIR measurement.

1. General procedure:

First the conductivity of the pure water was measured, then the pure superbase. After that, in a falcon tube was loaded 15ml of a given superbase (mTBD, mTBN, mTBO or dm3-mTBD) with a small stirring magnet. Then the conductivity probe was immerged (pay attention to fully submerge the "reading window" of the probe). The water amounts were added using a calibrated mechanical pipette. N.B! the conductivity may take few minutes before stabilizing.

2. Curves and raw data.

a. Water titration of mTBD

After plotting the conductivity data, we could observe some turning points on the conductivity curves. We used a tangent tool to get an estimation of the precise location of these points. Note that variations may occur from one experiment to another due to the instrument used, the ambient temperature / moisture or the "stabilization" time used to collect the conductivity data. For clarity, in the following section a picture of the entire conductivity curve is shown followed by zooms on the turning points area.

Water amount (molar equivalent according to Superbase)	Conductivity (µs/cm)	Water amount (molar equivalent according to Superbase)	Conductivity (µs/cm)	Water amount (molar equivalent according to Superbase)	Conductivity (µs/cm)	Water amount (molar equivalent according to Superbase)	Conductivity (µs/cm)
**0**	1.1	**1.7**	35.4	**5.2**	4220	**28**	105700
**1**	2.3	**1.8**	43.9	**5.3**	4280	**30**	107000
**0.1**	1.88	**1.9**	54.1	**5.4**	4570	**32**	107000
**0.2**	2.92	**2**	65.7	**5.5**	4920	**34**	105700
**0.3**	4.35	**2.5**	191.6	**5.6**	5270	**36**	105000
**0.4**	5.8	**2.75**	257	**5.8**	6070	**38**	103600
**0.5**	7.06	**2.85**	304	**6**	6820	**40**	102300
**0.6**	8.84	**3**	361	**7**	12010		
**0.7**	10.44	**3.25**	560	**8**	17730		
**0.8**	11.54	**3.5**	785	**10**	32700		
**0.9**	12.69	**3.75**	1008	**12**	47200		
**1**	13.9	**4**	1426	**14**	60200		
**1.1**	15.15	**4.25**	1904	**16**	71700		
**1.2**	16.6	**4.35**	2100	**18**	84000		

**1.3**	17.96	**4.45**	2280	**20**	90700		
**1.4**	20.8	**4.7**	2970	**22**	95000		
**1.5**	24.7	**5**	3780	**24**	99600		
**1.6**	29.7	**5.1**	4030	**26**	104000		

Table S137 Conductivity of mTBD in water.

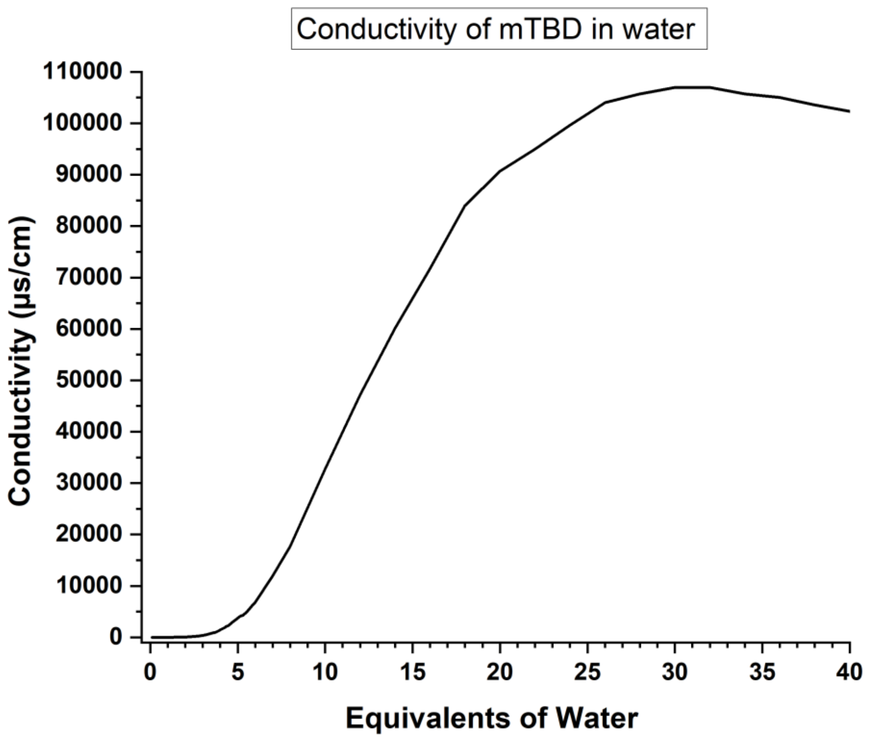

Figure S152 Conductivity data of mTBD in water.

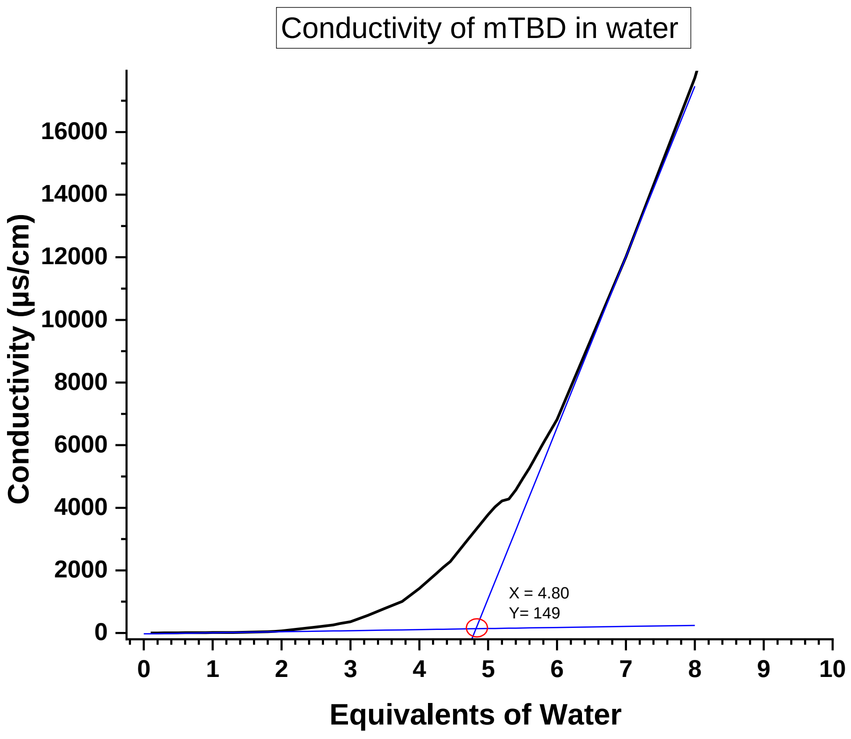

Figure S153 Conductivity data of mTBD in water.

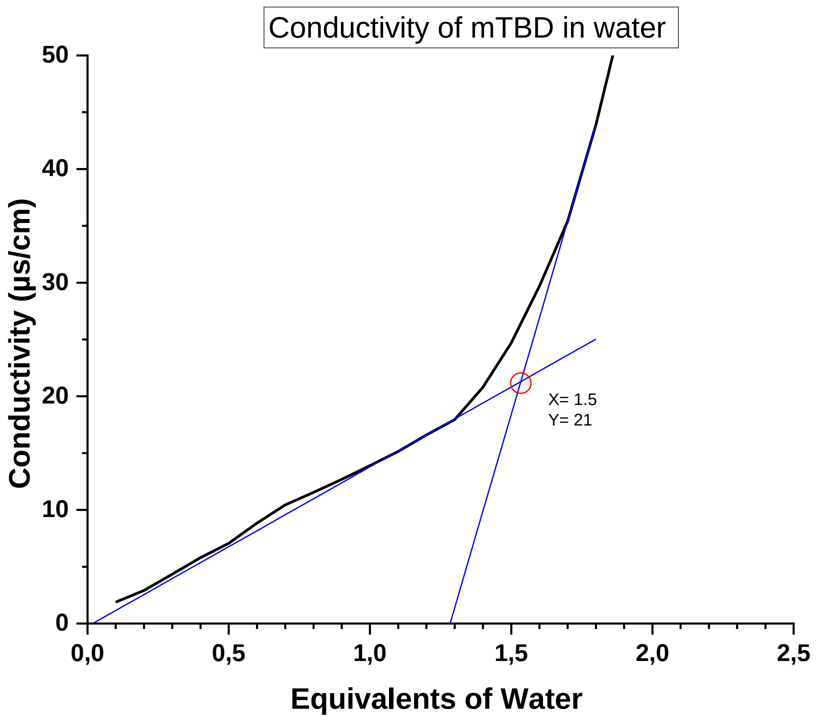

Figure S 154 Conductivity data of mTBD in water

b. water titration of mTBN

Water amount (molar equivalent according to Superbase)	Conductivity (µs/cm)	Water amount (molar equivalent according to Superbase)	Conductivity (µs/cm)	Water amount (molar equivalent according to Superbase)	Conductivity (µs/cm)
**0**	3.16	**5.2**	713	**66**	31400
**1**	4.27	**5.3**	768	**68**	31300
**0.1**	5.88	**5.5**	823	**70**	31200
**0.2**	7.85	**5.6**	877	**72**	31100
**0.3**	9.21	**5.8**	935	**74**	31000
**0.4**	10.52	**6**	1060	**76**	30800
**0.5**	11.43	**7**	1204	**78**	30700
**0.6**	12.19	**8**	2110	**80**	30600
**0.7**	12.58	**10**	3230	**82**	30500
**0.8**	13.05	**12**	5910	**84**	30400
**0.9**	13.4	**14**	8820	**86**	30300
**1**	13.52	**16**	11730	**88**	30100
**1.1**	13.92	**18**	14510	**90**	30000
**1.2**	14.2	**20**	16930		
**1.3**	14.67	**22**	19100		
**1.4**	15.17	**24**	21000		
**1.5**	15.54	**26**	22900		
**1.6**	16.35	**28**	24500		
**1.7**	17.24	**30**	25700		
**1.8**	18.66	**32**	26800		
**1.9**	20.1	**34**	27800		
**2**	22	**36**	28500		
**2.5**	41.7	**38**	29200		
**2.75**	57.9	**40**	29700		
**2.85**	65.5	**42**	30200		
**3**	89.4	**44**	30600		
**3.25**	121.7	**46**	30800		
**3.5**	162.3	**48**	31100		
**3.75**	211	**50**	31300		
**4**	272	**52**	31400		
**4.25**	343	**54**	31500		
**4.35**	362	**56**	31600		
**4.45**	393	**58**	31600		
**4.7**	488	**60**	31600		
**5**	624	**62**	31500		
**5.1**	668	**64**	31500		
**5.2**	713	**66**	31400		

Table S138 Conductivity of mTBN in water

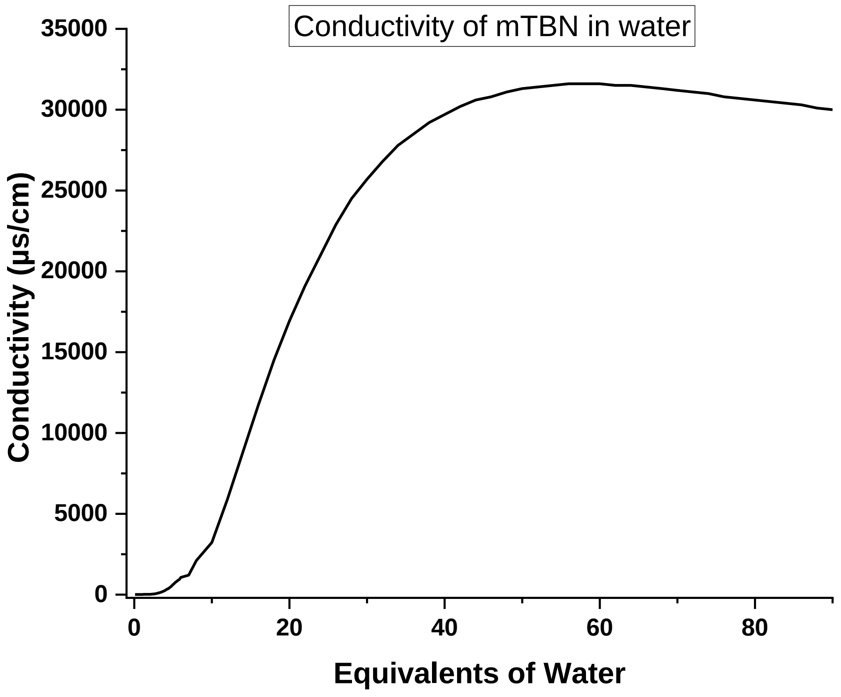

Figure S155 Conductivity data of mTBN in water.

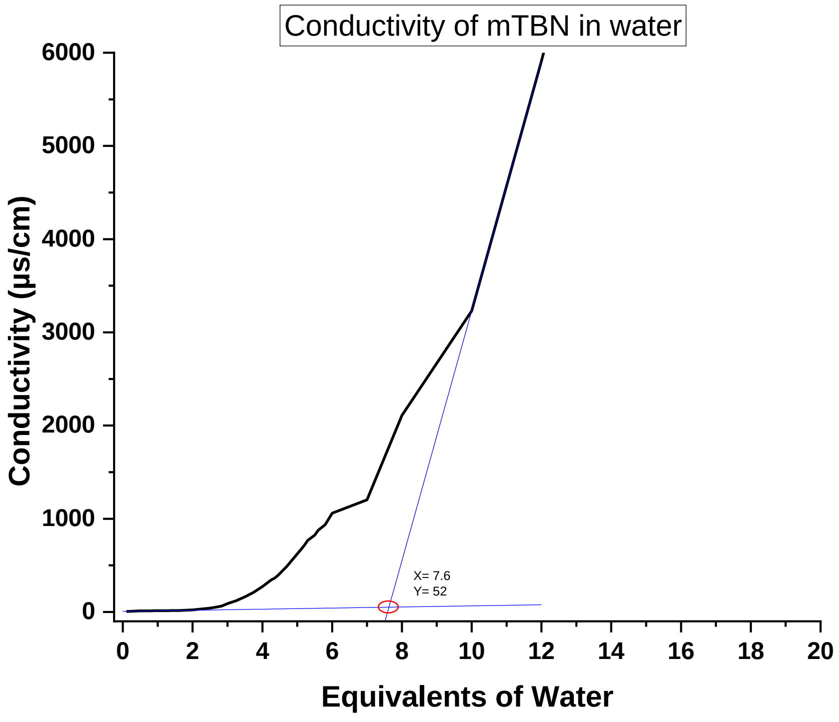

Figure S156 Conductivity data of mTBN in water.

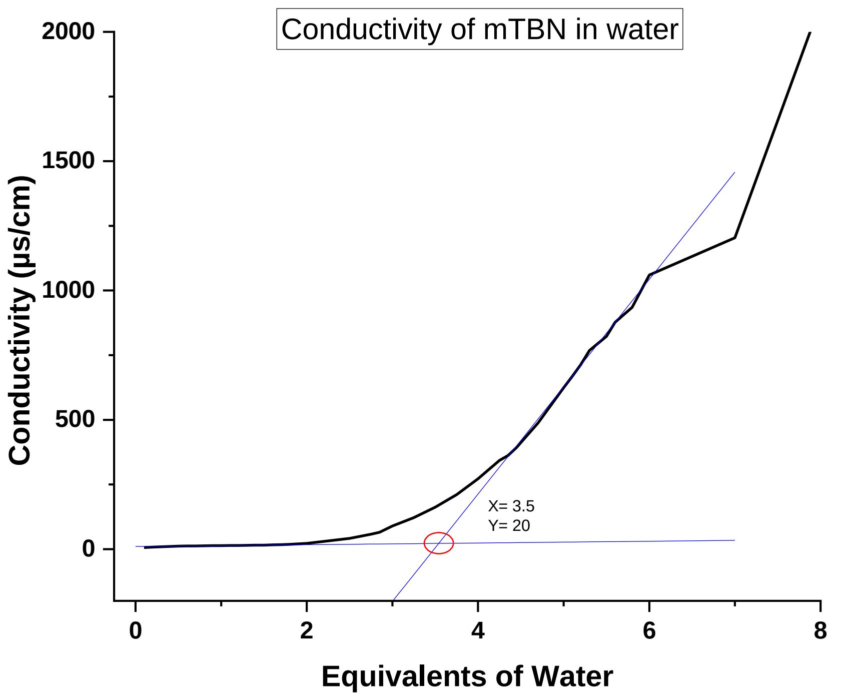

Figure S157 Conductivity data of mTBN in water.

c. *Water titration of dm3-mTBD*

Water amount (molar equivalent according to Superbase)	Conductivity (µs/cm)	Water amount (molar equivalent according to Superbase)	Conductivity (µs/cm)	Water amount (molar equivalent according to Superbase)	Conductivity (µs/cm)
0	1.16	3.5	644	28	88500
1	2.28	3.75	842	30	89400
0.1	1.94	4	1082	32	88300
0.2	3.38	4.25	1372	34	88200
0.3	5.21	4.35	1485	36	88770
0.4	7.4	4.45	1606	38	87400
0.5	10.08	4.7	2050	40	87000
0.6	13.18	5	2650	42	86600
0.7	16.59	5.1	2840		
0.8	19.63	5.2	3080		
0.9	22.8	5.3	3250		
1	25.8	5.4	3490		

1.1	29	5.5	3710		
1.2	32.9	5.6	3960		
1.3	36.1	5.8	4500		
1.4	40	6	5070		
1.5	44.1	7	8790		
1.6	49	8	13100		
1.7	55.3	10	22900		
1.8	62.2	12	33200		
1.9	70.9	14	43000		
2	82.1	16	52000		
2.5	181.3	18	62700		
2.75	238	20	68200		
2.85	273	22	76200		
3	364	24	77900		
3.25	477	26	85000		

Table S 139 Conductivity of dm3-mTBD in water.

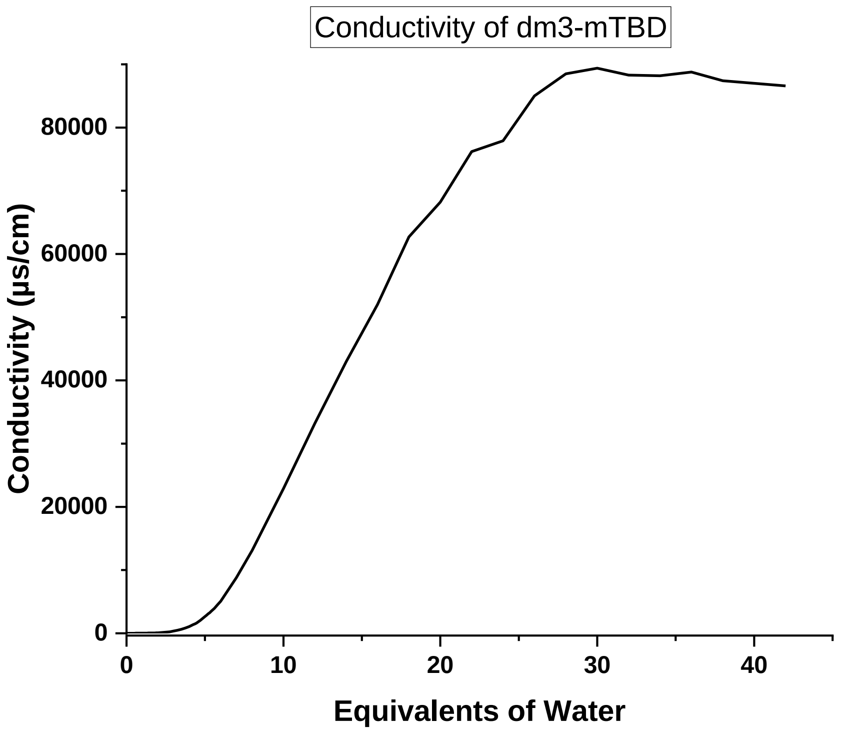

Figure S158 Conductivity data of dm3-mTBD in water.

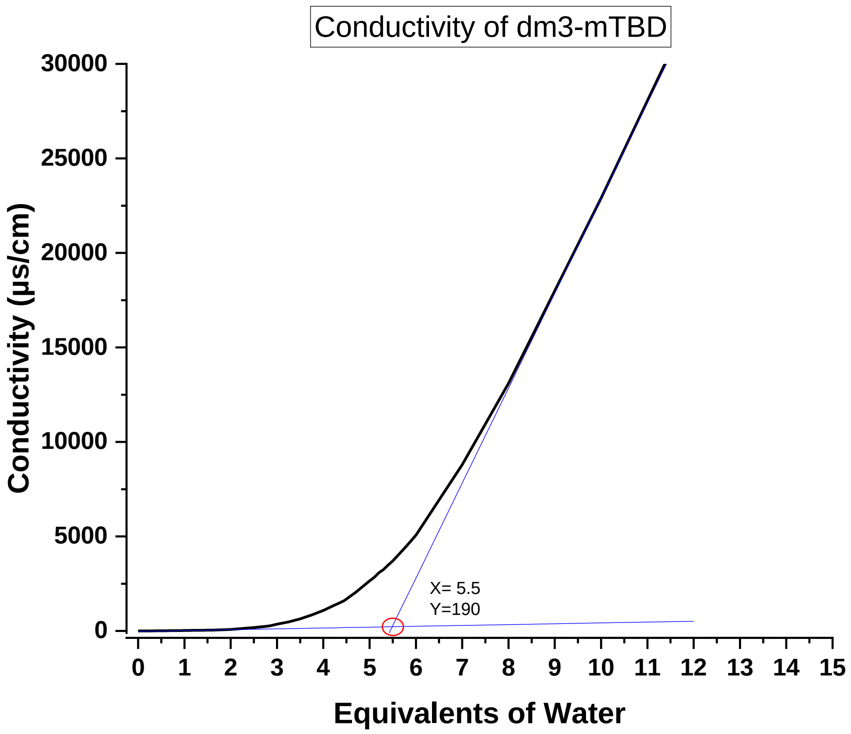

Figure S159 Conductivity data of dm3-mTBD in water.

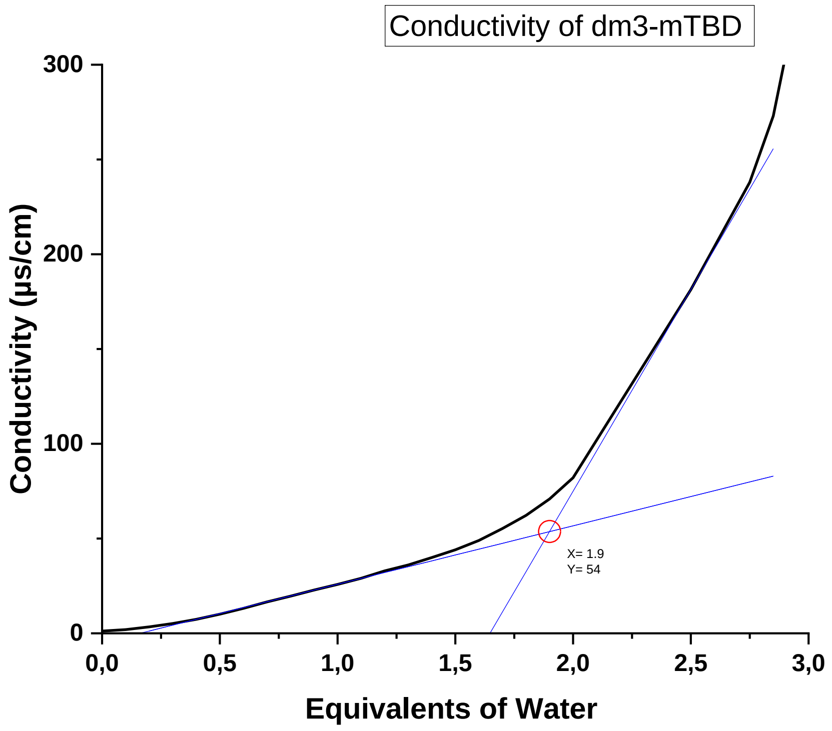

Figure S160 Conductivity data of dm3-mTBD in water.

d. Water titration of mTBO

Water amount (molar equivalent according to Superbase)	Conductivity (µs/cm)	Water amount (molar equivalent according to Superbase)	Conductivity (µs/cm)
**0**	2.57	**4.25**	244
**1**	2.45	**4.35**	267
**0.1**	3.55	**4.45**	274
**0.2**	5.4	**4.7**	291
**0.3**	8.55	**5**	317
**0.4**	12.97	**5.1**	345
**0.5**	18.9	**5.2**	350
**0.6**	35.3	**5.3**	357
**0.7**	45	**5.4**	374
**0.8**	51.1	**5.5**	386
**1**	56.5	**5.6**	400
**1.1**	65.4	**5.8**	407
**1.2**	70.6	**6**	430
**1.3**	75.5	**7**	433
**1.4**	77	**8**	581
**1.5**	93.7	**10**	730
**1.6**	97.5	**12**	1025
**1.7**	101.5	**14**	1375
**1.8**	105	**16**	1446
**1.9**	110.9	**18**	1616
**2**	118	**20**	2150
**2.5**	126.8	**22**	2370
**2.75**	161.5	**24**	2540
**2.85**	164.8	**26**	2680
**3**	167.5	**28**	2800
**3.25**	182.2	**30**	2880
**3.5**	187.7	**32**	2940
**3.75**	209	**34**	3030
**4**	226	**36**	3060
**38**	3110		

**40**	3130
**42**	3150
**44**	3150
**46**	3150
**48**	3150
**50**	3150
**52**	3150
**54**	3150
**56**	3150
**58**	3150
**60**	3150
**62**	3130
**64**	3110

Table S140 Conductivity of mTBO in water

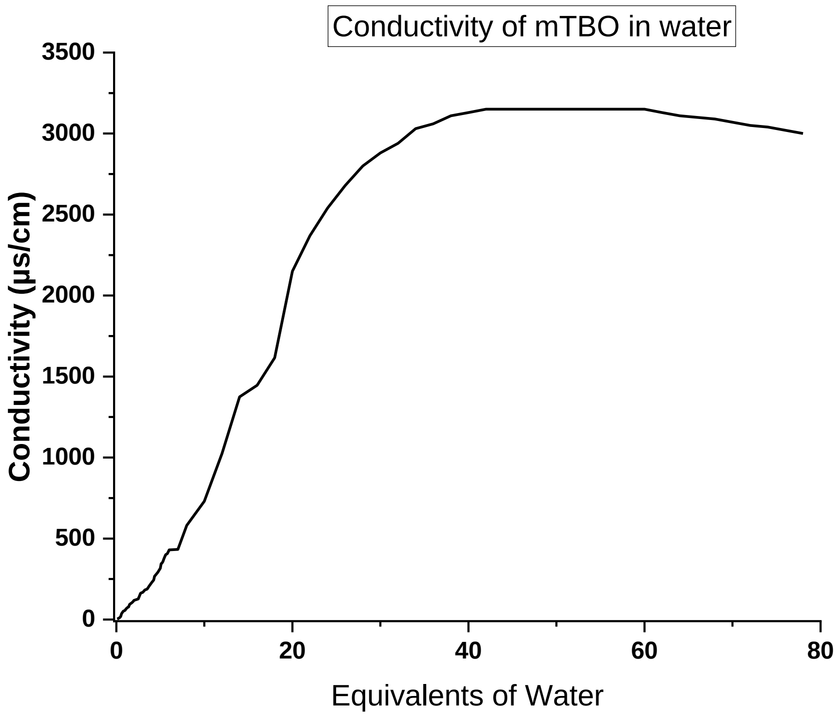

Figure S161 Conductivity data of mTBO in water.

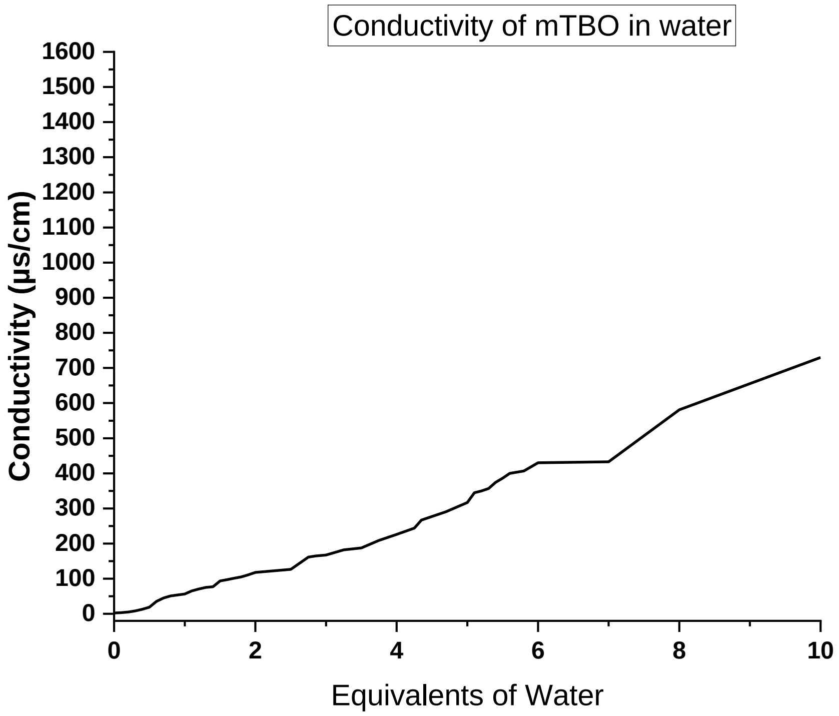

Figure S162 Conductivity data of mTBO in water.

V. PH measurements

a. KOH or mTBD in pure distilled water

A pH meter was used to follow the pH of a 100ml distilled water to which a solution of KOH or mTBD (0.1 M, 10 mL 0.056 or 0.15g respectively) was gradually added.

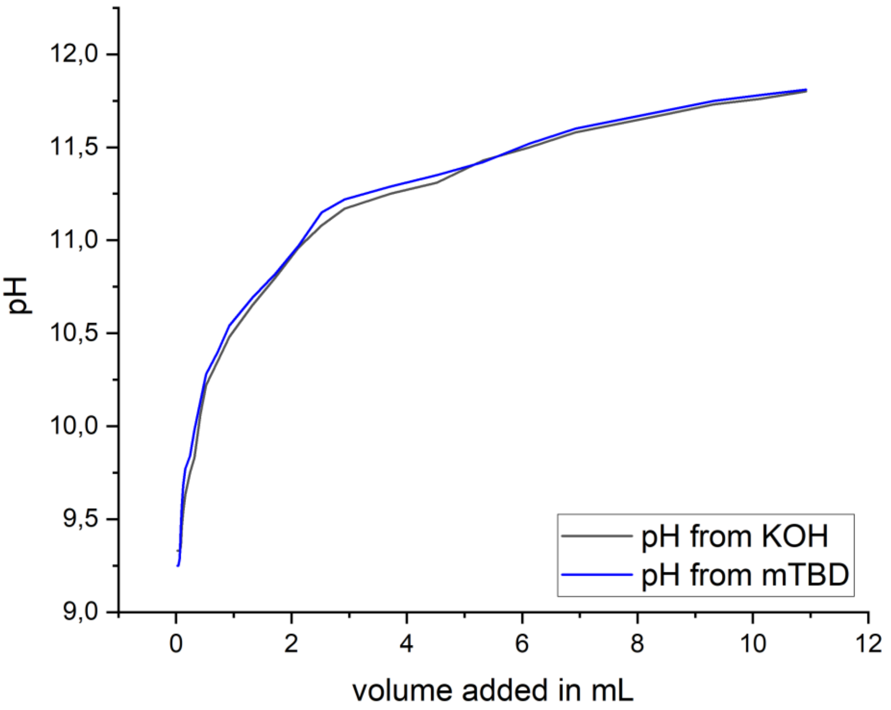

Figure S163 pH evolution of pure water in function of the addition of KOH or mTBD.

b. KOH or mTBD in distilled water with acetic acid

A pH meter was used to follow the pH of a 100ml distilled water with a 0.1 M concentration of acetic acid (0.3 g of acetic acid) to which a solution of KOH or mTBD (0.1 M, 10 mL 0.056 or 0.15g respectively) was gradually added.

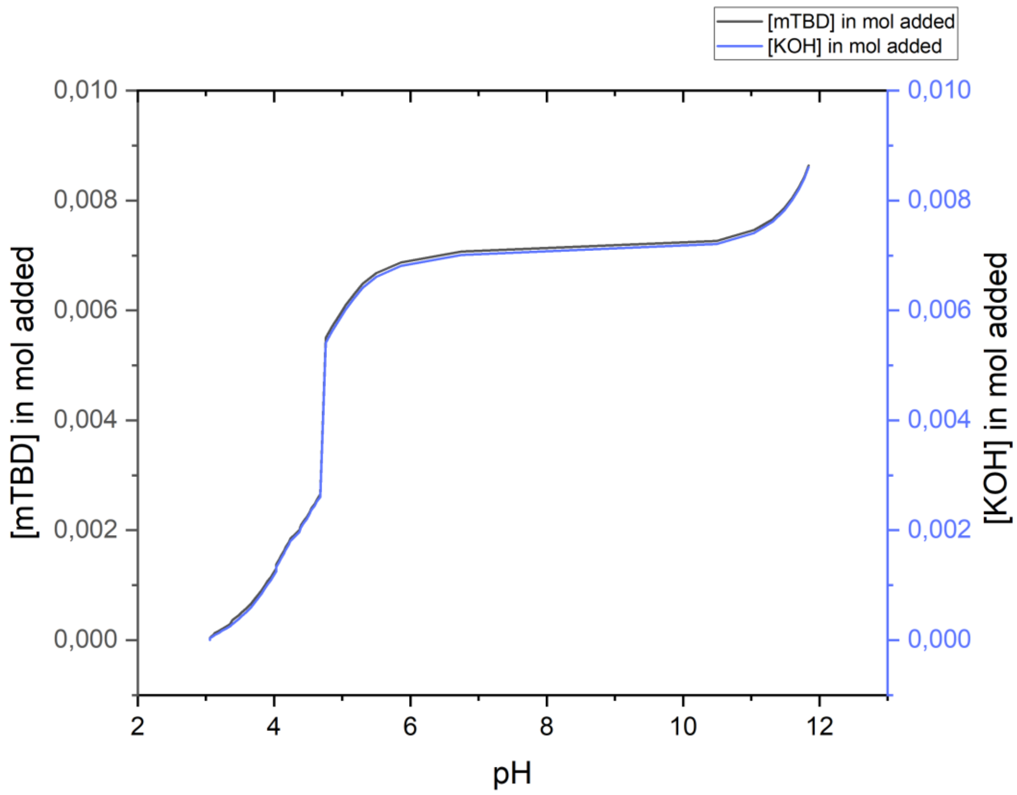

Figure S164 pH evolution of an acidic solution in function of the addition of KOH or mTBD.

c. pH of KOAc and [mTBDH][OAc] solutions

10 mL solution of 0.5 M, 1 M, 2M, 3M, 5 M and 10 M of KOAc or [mTBDH][OAc] were prepared, and their pH was measured with a pH meter.

Molar concentration	pH value	
	KOAc	[mTBDH][OAc]
0.5	7.7	6.88
1	7.92	7.0
2	8.21	8.13
3	8.66	8.5
5	9.46	10.71
10	10.90	12.3

Table S141 pH evolution of a solution of KOAc or [mTBDH][OAc] in function of their concentration.

VI. InfraRed measurements

The measurements were performed using an FTIR instrument. As previously mentioned, the sample measured by IR spectroscopy is the same sample where the conductivity was measured. In the following section, for each experiment, a figure of all the spectra combined are shown. followed by an expansion of the CH2 region (2800 – 3000 cm-^{1}) and the imine region (1400-1600cm^{-1}). The raw data are available upon request.

1. FTIR measurements of water titration

a. mTBD

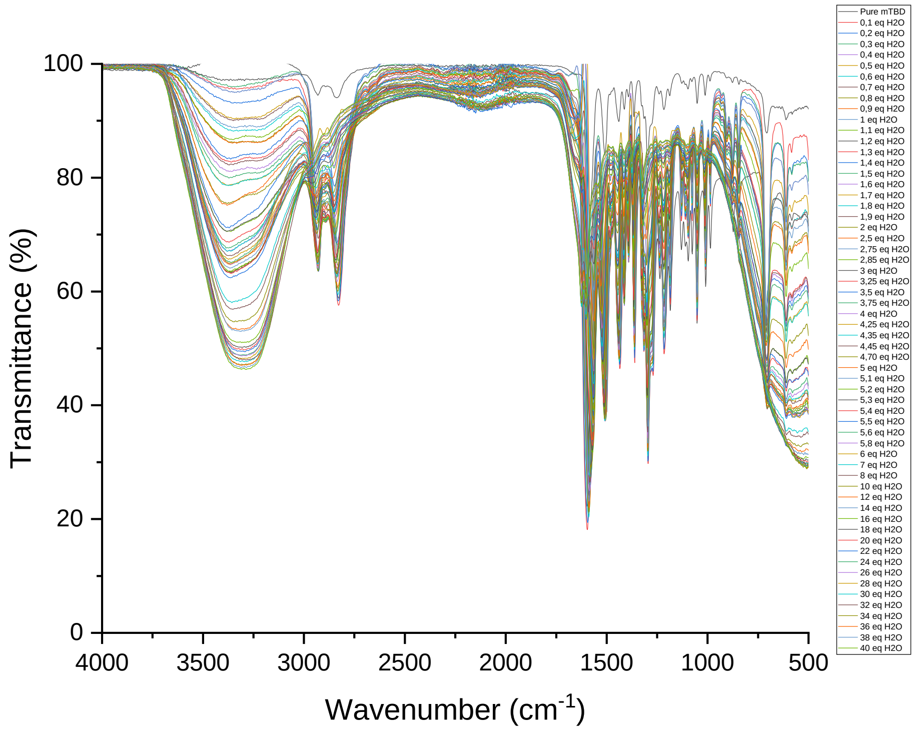

Figure S165 IR of water titration of mTBD.

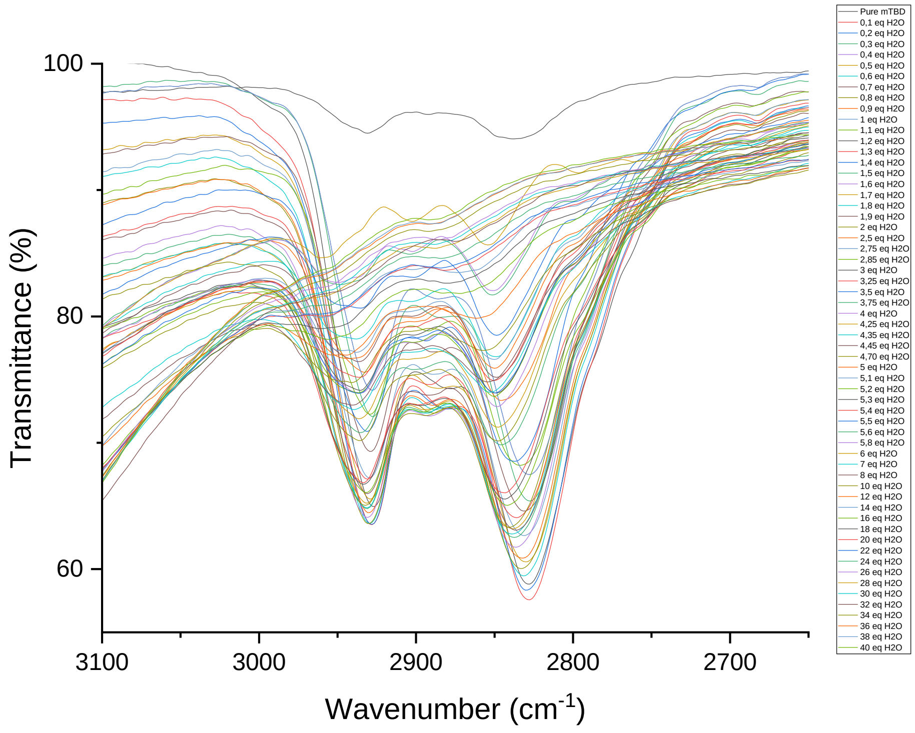

Figure S166 expansion of the mTBD CH_2 region (2800 – 3000 cm^{-1})

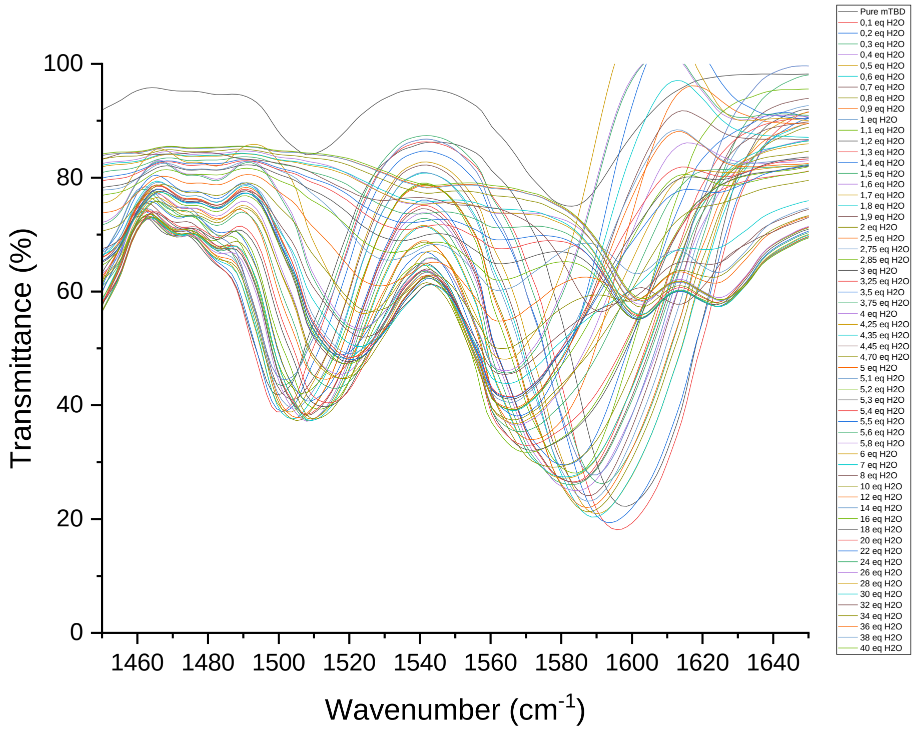

Figure S167 expansion of the mTBD imine region (1400 – 1600 cm^{-1})

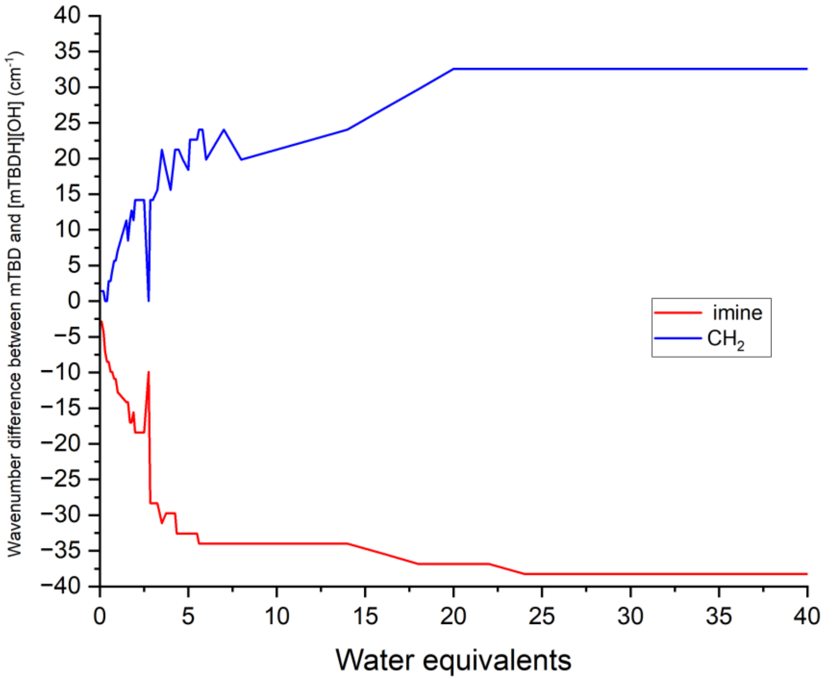

Figure S 168 IR shift of the imine and CH_2 IR signal of mTBD in function of the water equivalents.

b. mTBN

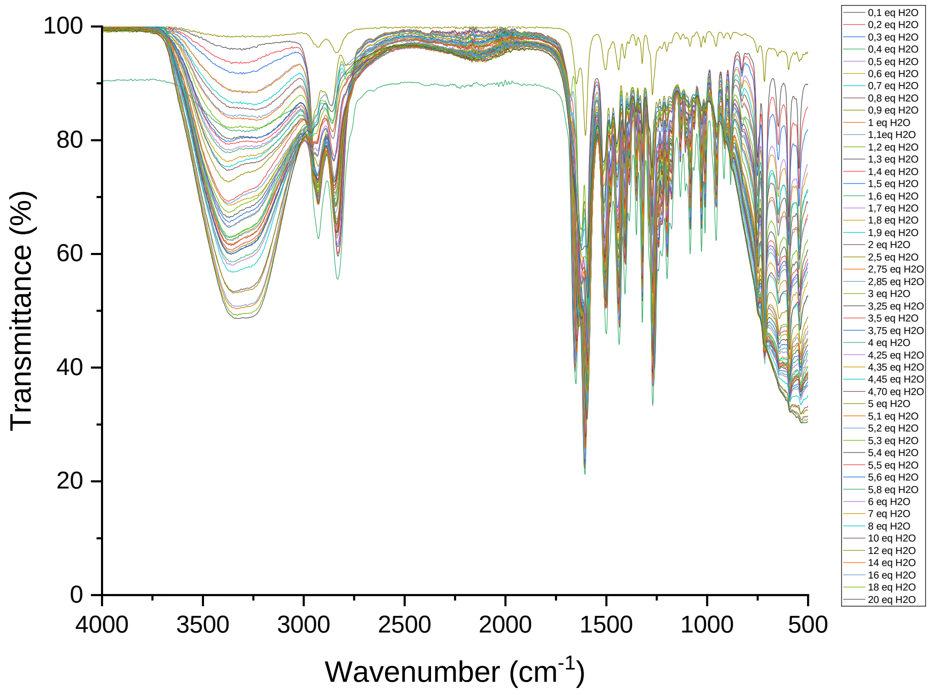

Figure S169 IR of water titration of mTBN.

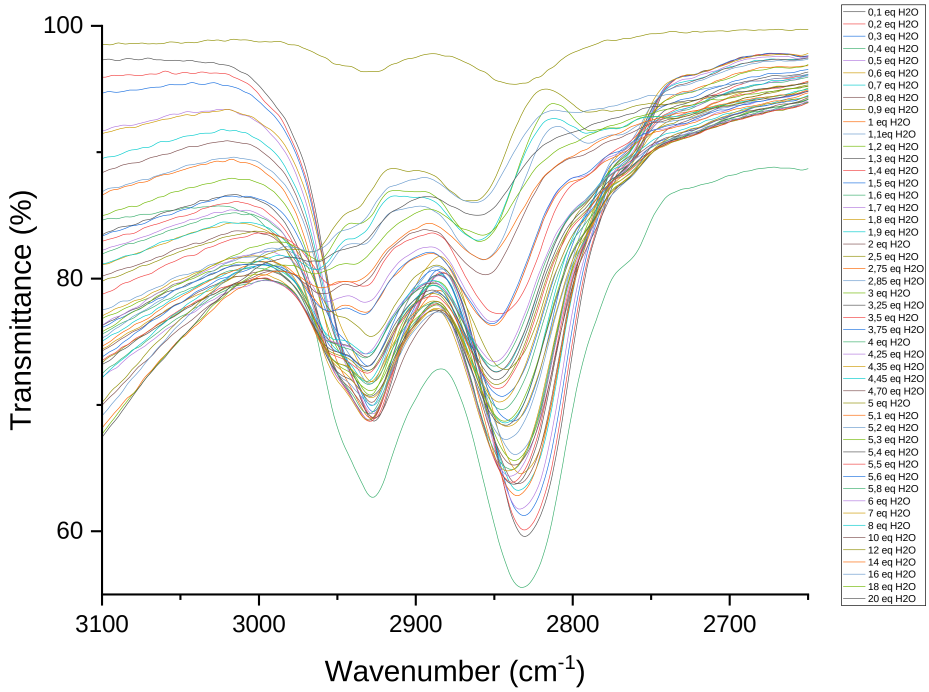

Figure S 170 expansion of the mTBN CH_2 region (2800 – 3000 cm^{-1})

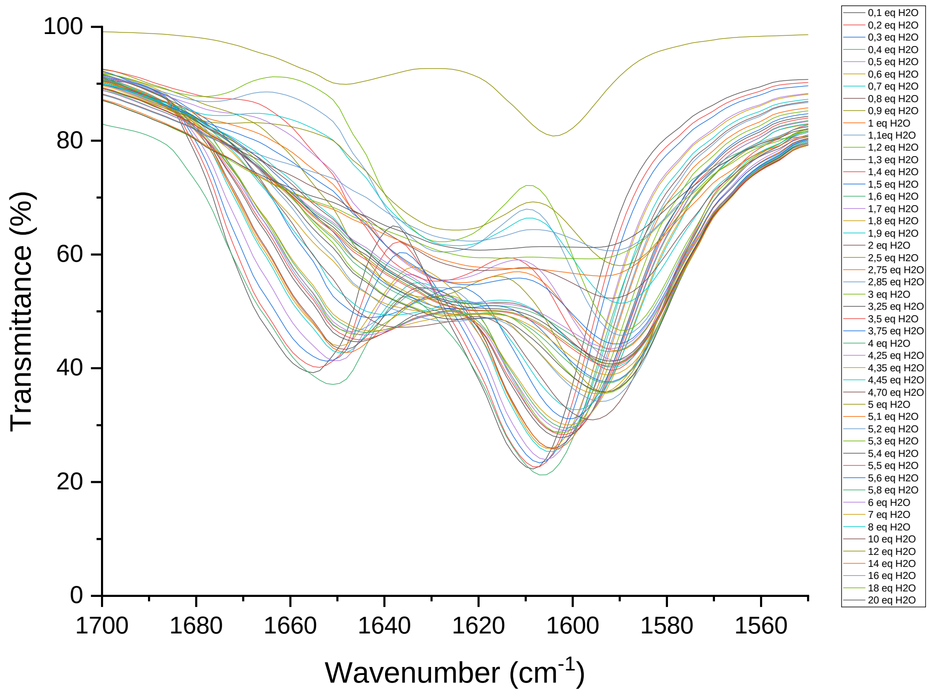

Figure S171 expansion of the mTBN imine region (1400 – 1600 cm^{-1})

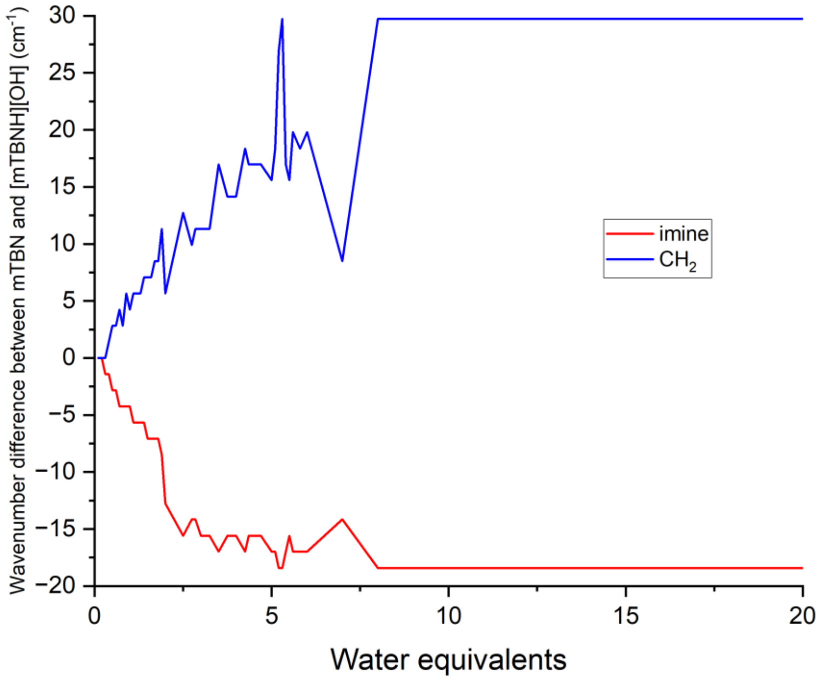

Figure S 172 IR shift of the imine and CH_2 IR signal of mTBN in function of the water equivalents.

c. dm3-mTBD

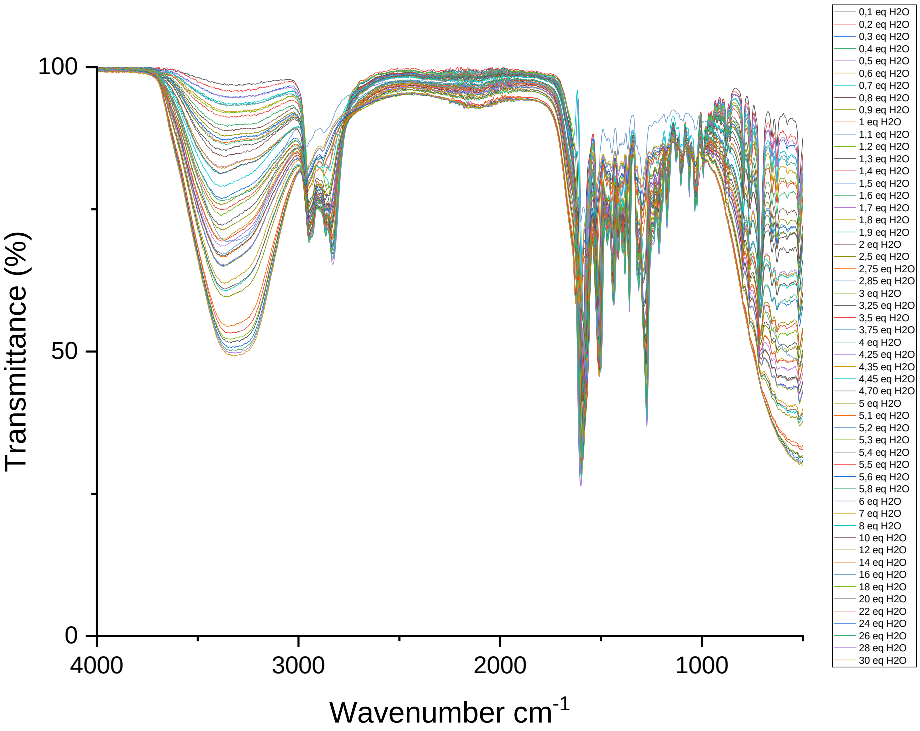

Figure S173 IR of water titration of dm3-mTBD.

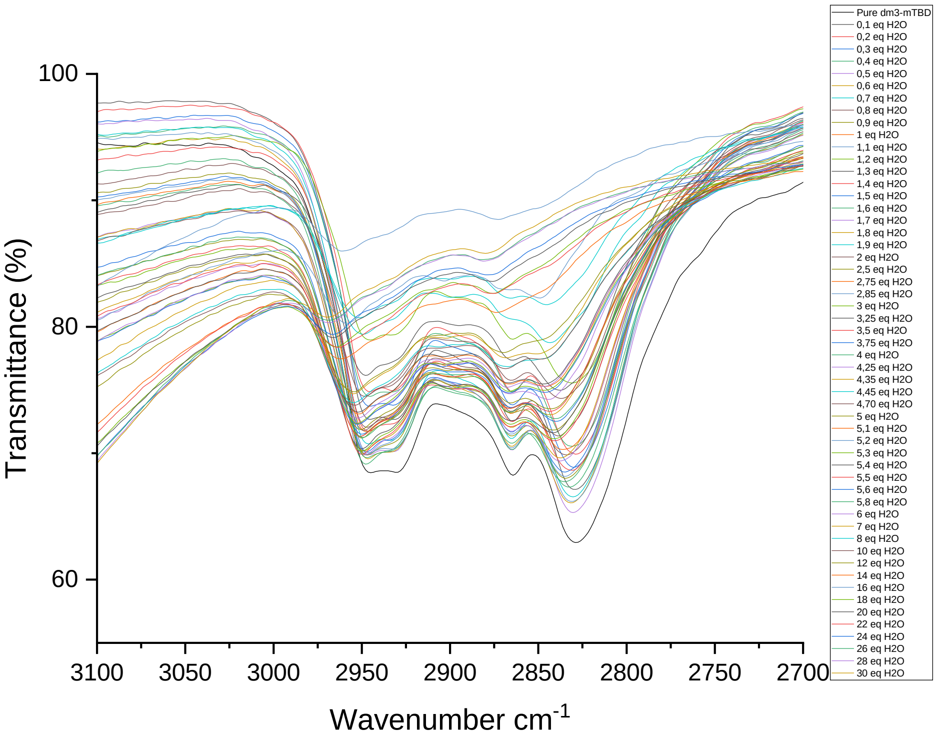

Figure S174 expansion of the dm3-mTBD CH_2 region (2800 – 3000 cm^{-1})

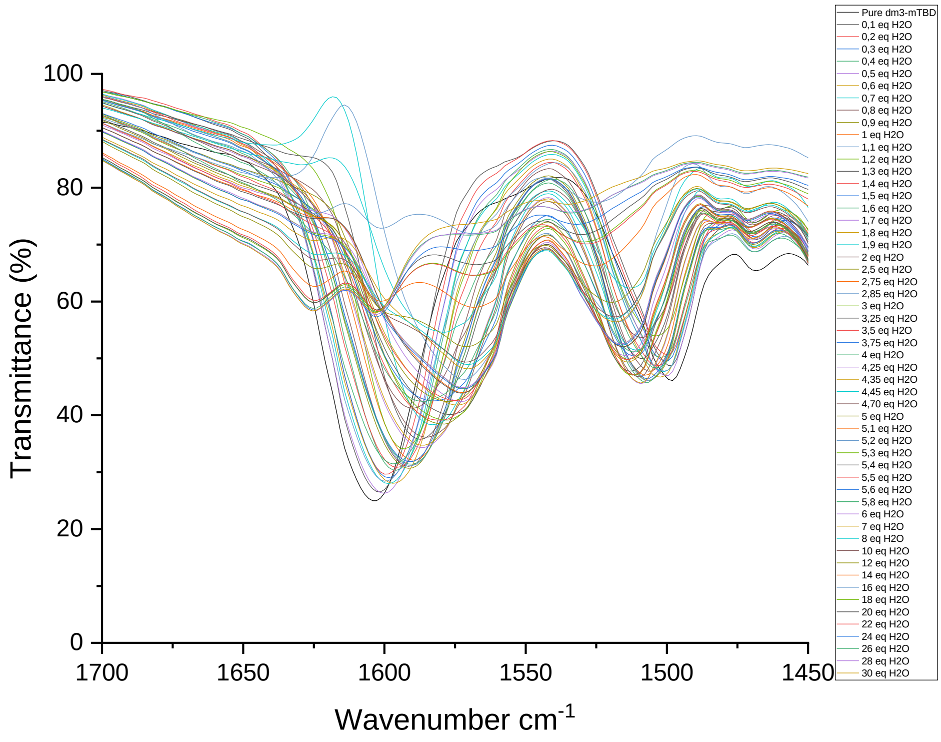

Figure S175 expansion of the dm3-mTBD imine region (1400 – 1600 cm^{-1})

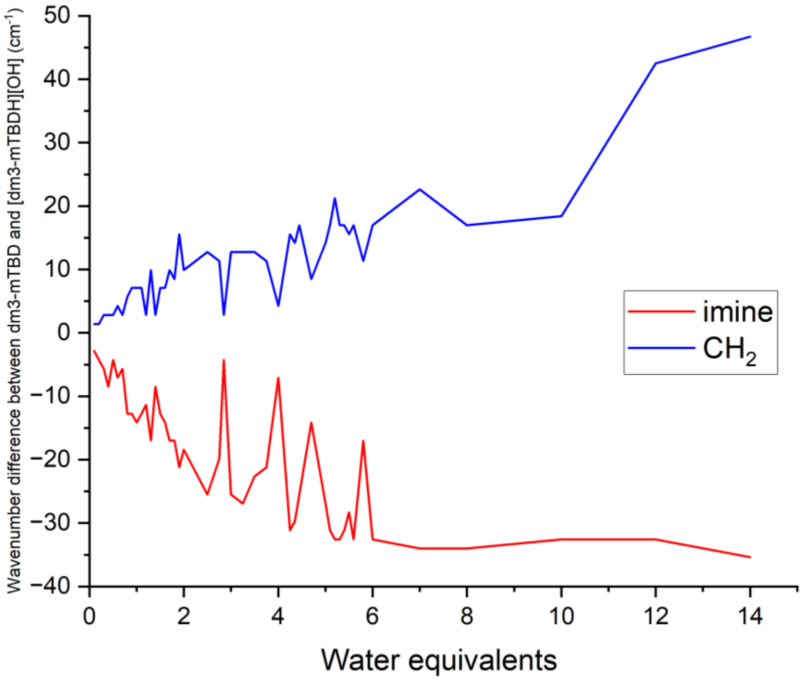

Figure S 176 IR shift of the imine and CH_2 IR signal of dm3-mTBD in function of the water equivalents.

d. mTBO

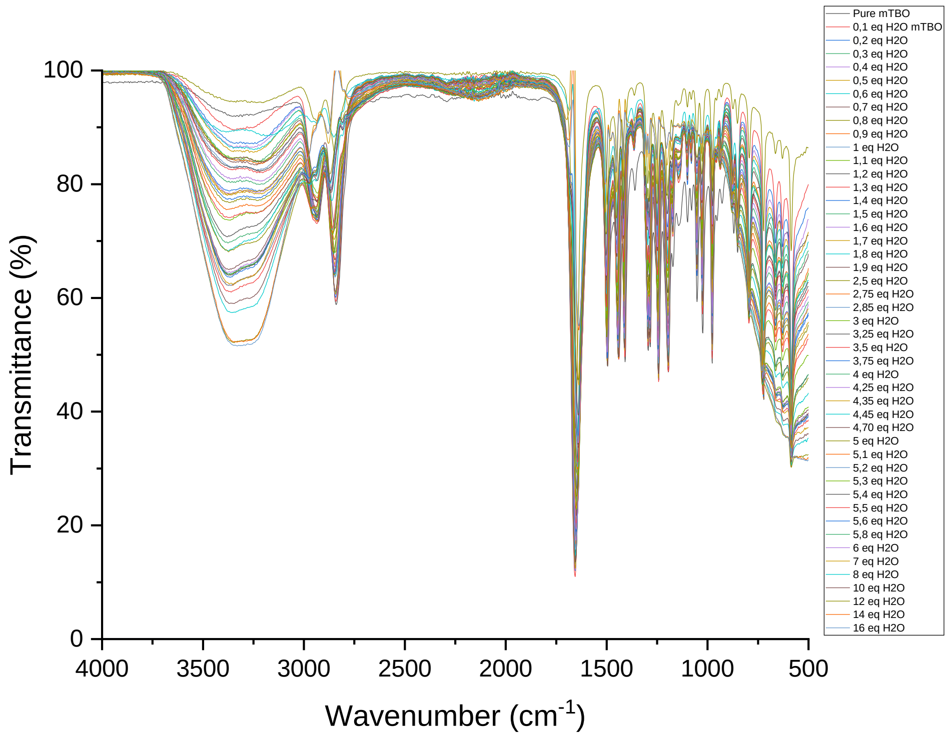

Figure S177 IR of water titration of mTBO.

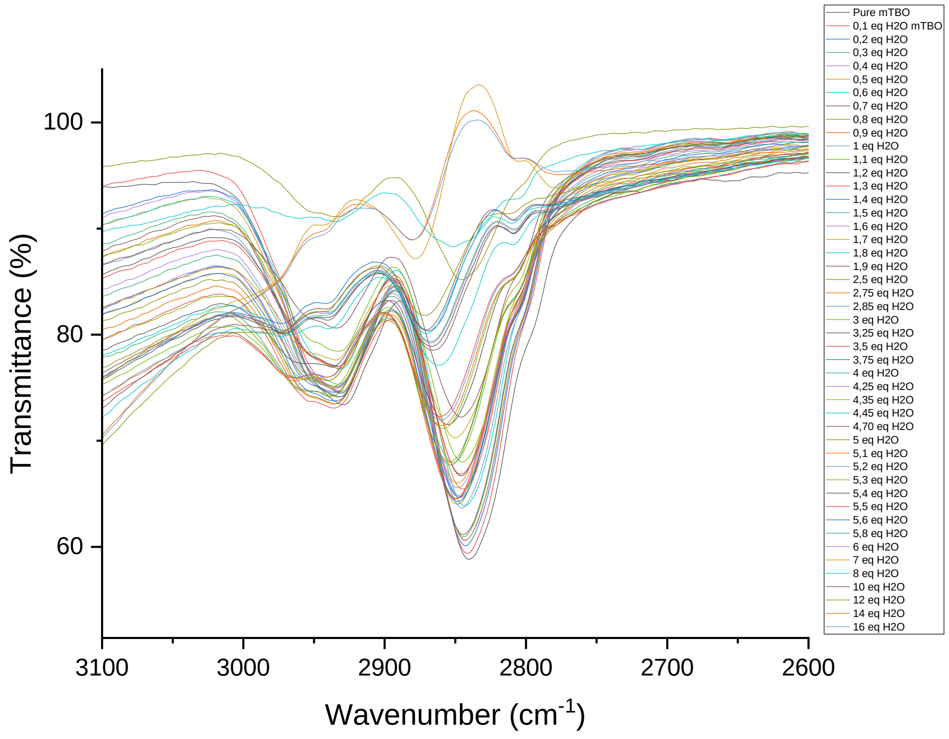

Figure S178 expansion of the mTBO CH_2 region (2800 – 3000 cm^{-1})

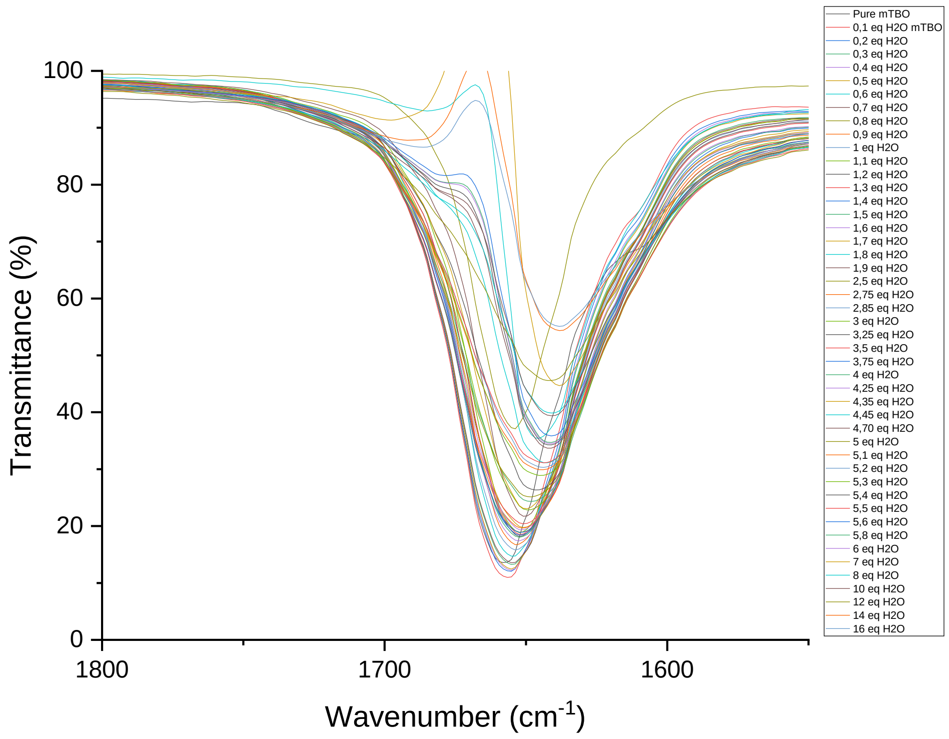

Figure S179 expansion of the dm3-mTBD imine region (1400 – 1600 cm^{-1})

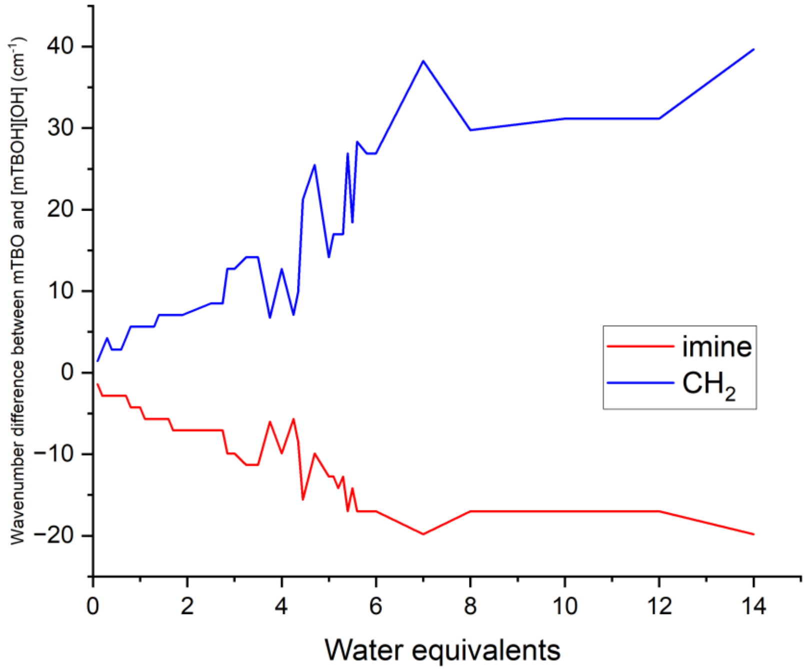

Figure S 180 IR shift of the imine and CH_2 IR signal of mTBO in function of the water equivalents.

VII. Prediction of reaction free energy barriers

The Gibbs free energy barriers ($\Delta^{\ddagger}G$) of reaction steps involved in the hydrolysis of mTBD were estimated with quantum chemical methods.

Conformational isomers of reactants, transition states and products were initially searched with Spartan'20[1] using the Merck Molecular Force Field (MMFF) method[2]. Next, the geometry of each conformer was optimized at the BP86/def-TZVP-COSMO[3-6] level of theory, using TURBOMOLE v7.8[7,8], with subsequent frequency calculations done to ensure that the structures correspond to appropriate stationary points. Intrinsic Reaction Coordinate (IRC) calculations served to characterize the minima (reactants and products) connected to each transition state.

The optimized conformer sets produced this way were used as input to COSMO*conf* v'24[9], using a custom job workflow, which included the following steps: 1) Single-point calculation at the BP86/def2-TZVPD-COSMO level of theory[3-6,10] with a smooth-radii based isosurface cavity (FINE) parameterization; 2) Removal of duplicate confromers based on geometry similarity, using COSMO*conf*'s CLUSTER_GEOCHECK algorithm; 3) Single-point calculation at the BP86/def2-TZVPD level of theory, but without COSMO.

Once the BP86/def2-TZVPD//BP86/def2-TZVP COSMO screening surface charge densities and gas-phase energies were obtained for each conformer set, the multi-conformer free energy of each species in pure water was obtained with the COnductor like Screening MOdel for Real Solvents (COSMO-RS)[11-13] implemented in COSMO*therm* v'24[14]. The reference state used was 1 bar of ideal gas and 1 mol/L for the liquid solvent, and all calculations were done at 298 K.

The modelled step-wise hydrolysis mechanism included the following reaction steps: Initial nucleophilic attack of water on the protonated mTBD, forming a protonated hemiaminal intermediate (hamH$^+$), shown in Scheme 1; Initial nucleophilic attack of OH$^-$ on the protonated mTBD, forming a neutral hemiaminal intermediate, shown in Scheme 2; Nucleophilic attack of OH$^-$ on the neutral mTBD, forming an anionic intermediate, shown in Scheme 3; Nucleophilic attack of water on the protonated mTBD catalyzed by acetate, shown in Scheme 4; Hemiaminal zwitterion formation and subsequent deamination step, shown in Scheme 5; Concerted deamination catalyzed by acetate, shown in Scheme 6.

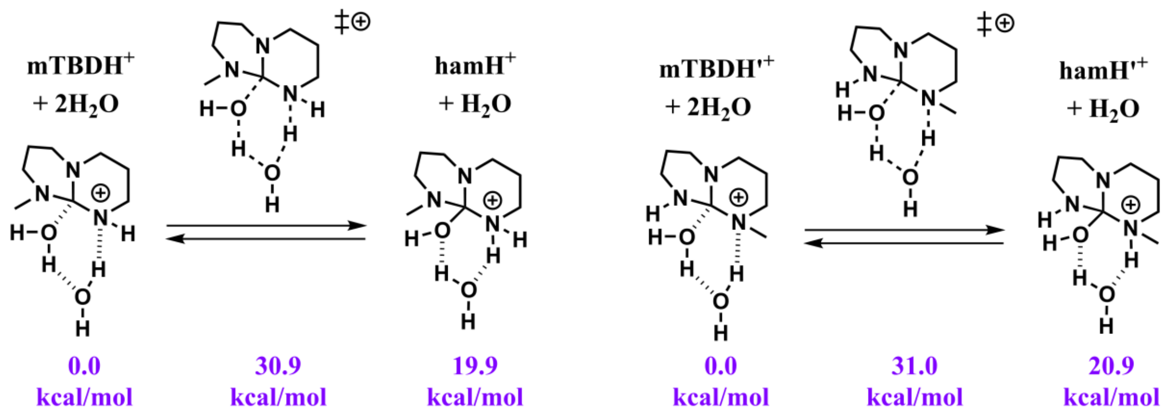

Scheme 1) Hemiaminal formation step: Water attack on protonated mTBD.

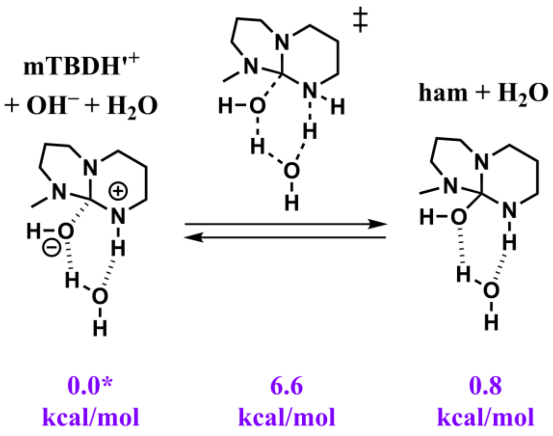

Scheme 2) Hemiaminal formation step: OH^- attack on protonated mTBD. *: reactant-side free energy calculated for the IRC end-point, without full geometry optimization.

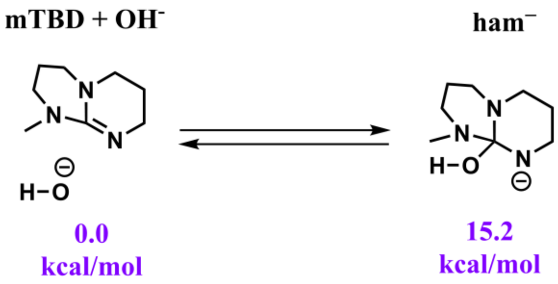

Scheme 3) OH^- attack on neutral mTBD. No transition state was found. Free energy of anionic product calculated relative to separate reactants (mTBD + OH^-).

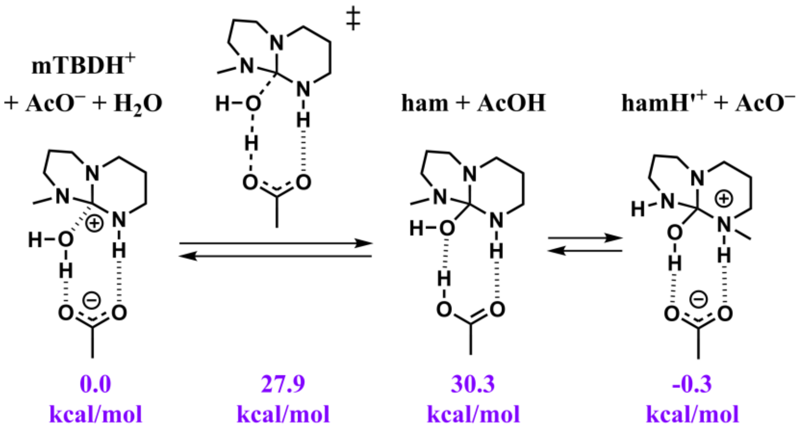

Scheme 4) Hemiaminal formation step: Water attack on protonated mTBD catalyzed by acetate.

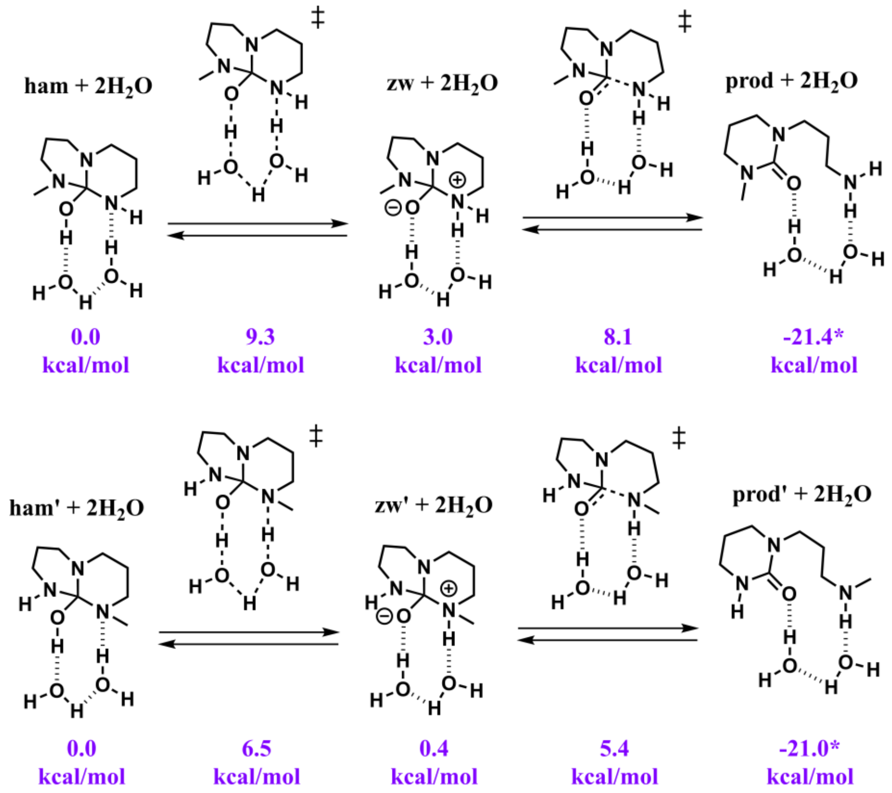

Scheme 5) Hemiaminal zwitterion formation step followed by deamination reaction step. *: summed free energy of separate products (prod + $2H_2O$).

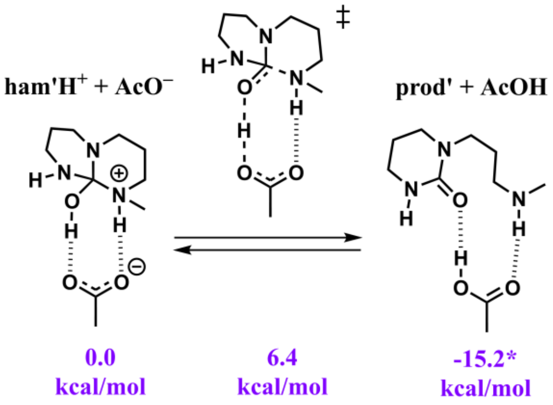

Scheme 6) Concerted deamination reaction step catalyzed by acetate. *: summed free energy of separate products (prod' + AcOH).

VIII. References:

1) E. Gazagnaire, J. Helminen, A. W. T. King, T. Golin Almeida, T. Kurten and I. Kilpeläinen, 2024, 12119–12124.
2) Spartan'16. *Wavefunct. Inc Irvine, CA* **2016**. https://doi.org/https://downloads.wavefun.com/Spartan20Manual.pdf.
3) Halgren, T. A. MMFF VII. Characterization of MMFF94, MMFF94s, and Other Widely Available Force Fields for Conformational Energies and for Intermolecular- Interaction Energies and Geometries. **2000**, *20* (7), 730–748. https://doi.org/doi.org/10.1002/(SICI)1096-987X(199905)20:7<730::AID-JCC8>3.0.CO;2-T.
4) Becke, A. . Density-Fnnctional Exchange-Energy Approximation with Correct Asymptotic Behavior. *J. Chem. Phys.* **1988**, *4* (4), 276–282. https://doi.org/10.1063/1.1749835.
5) Perdew, J. P. Density-Functional Approximation for the Correlation Energy of the Inhomogeneous Electron Gas. *Pay Perform. Heal. Care Methods Approaches* **1986**, *33* (June), 8822–8824. https://doi.org/10.3768/rtipress.2011.bk.0002.1103.12.
6) Weigend, F. Accurate Coulomb-Fitting Basis Sets for H to Rn. *Phys. Chem. Chem. Phys.* **2006**, *8* (9), 1057–1065. https://doi.org/10.1039/b515623h.
7) Klamt, A.; Schuurmann, G. *COSMO: A New Approach to Dielectric Screening in Solvents with Explicit Expressions for the Screening Energy and Its Gradient*; 1993.
8) GmbH, T. TURBOMOLE V7.7 2022. a Development of University of Karlsruhe and Forschungszentrum Karlsruhe GmbH,. TURBOMOLE GmbH, since 2007. https://www.turbomole.org.
9) Balasubramani, S. G.; Chen, G. P.; Coriani, S.; Diedenhofen, M.; Frank, M. S.; Franzke, Y. J.; Furche, F.; Grotjahn, R.; Harding, M. E.; Hättig, C.; Hellweg, A.; Helmich-Paris, B.; Holzer, C.; Huniar, U.; Kaupp, M.; Marefat Khah, A.; Karbalaei Khani, S.; Müller, T.; Mack, F.; Nguyen, B. D.; Parker, S. M.; Perlt, E.; Rappoport, D.; Reiter, K.; Roy, S.; Rückert, M.; Schmitz, G.; Sierka, M.; Tapavicza, E.; Tew, D. P.; Van Wüllen, C.; Voora, V. K.; Weigend, F.; Wodyński, A.; Yu, J. M. TURBOMOLE: Modular Program Suite for Ab Initio Quantum-Chemical and Condensed-Matter Simulations. *J. Chem. Phys.* **2020**, *152* (18), 184107. https://doi.org/10.1063/5.0004635.
10) BIOVIA COSMOconf. *Dassault Systèmes* **2024**, http://www.3ds.com.
11) Rappoport, D.; Furche, F. Property-Optimized Gaussian Basis Sets for Molecular Response Calculations. *J. Chem. Phys.* **2010**, *133* (13), 134105. https://doi.org/10.1063/1.3484283/920872.
12) Klamt, A. Conductor-like Screening Model for Real Solvents: A New Approach to the Quantitative Calculation of Solvation Phenomena. *J. Phys. Chem.* **1995**, *99* (7), 2224–2235. https://doi.org/10.1021/j100007a062.
13) Klamt, A.; Jonas, V.; Bürger, T.; Lohrenz, J. C. W. Refinement and Parametrization of COSMO-RS. *J. Phys. Chem. A* **1998**, *102* (26), 5074–5085. https://doi.org/10.1021/jp980017s.
14) Eckert, F.; Klamt, A. Fast Solvent Screening via Quantum Chemistry: COSMO-RS Approach. *AIChE J.* **2002**, *48* (2), 369–385. https://doi.org/10.1002/AIC.690480220.
15) BIOVIA COSMOtherm. Release 2019; Dassault Systèmes: San Diego 2019. http://www.3ds.com.
